# The Biokinetic Spectrum for Temperature

**DOI:** 10.1371/journal.pone.0153343

**Published:** 2016-04-18

**Authors:** Ross Corkrey, Tom A. McMeekin, John P. Bowman, David A. Ratkowsky, June Olley, Tom Ross

**Affiliations:** Tasmanian Institute of Agriculture / School of Land and Food, University of Tasmania, Hobart, Tasmania, Australia; National Cancer Institute, UNITED STATES

## Abstract

We identify and describe the distribution of temperature-dependent specific growth rates for life on Earth, which we term the biokinetic spectrum for temperature. The spectrum has the potential to provide for more robust modeling in thermal ecology since any conclusions derived from it will be based on observed data rather than using theoretical assumptions. It may also provide constraints for systems biology model predictions and provide insights in physiology. The spectrum has a Δ-shape with a sharp peak at around 42°C. At higher temperatures up to 60°C there was a gap of attenuated growth rates. We found another peak at 67°C and a steady decline in maximum rates thereafter. By using Bayesian quantile regression to summarise and explore the data we were able to conclude that the gap represented an actual biological transition between mesophiles and thermophiles that we term the Mesophile-Thermophile Gap (MTG). We have not identified any organism that grows above the maximum rate of the spectrum. We used a thermodynamic model to recover the Δ-shape, suggesting that the growth rate limits arise from a trade-off between activity and stability of proteins. The spectrum provides underpinning principles that will find utility in models concerned with the thermal responses of biological processes.

## Introduction

The cell maintains itself in a thermodynamically non-equilibrium state with respect to its environment, but its capacity to do so has a limited temperature range [1]. Within that range organisms can exhibit varying growth rates, with some growing more quickly than others, with the fastest growth being ultimately limited by cellular mechanisms, including the error-rate of replication, and accuracy and regulation of synthesis [2]. Previous work by us concerned a model of temperature-dependent growth rates of microbes that were empirical [3], but this was followed by a semi-mechanistic model based on thermodynamic properties of protein folding [4]. In this paper we examine growth rates in a much broader context: growth rates of all life, those rates that are possible at various temperatures, and how they are distributed. Others have attempted to relate the metabolic rates of organisms to larger scale ecology processes [5]. That particular model has been criticised on several grounds including its assumption of a constant activation energy [6] and the validity of its size scaling coefficient [7]. In this paper we also infer relationships between temperature-dependent growth with larger scale ecological processes and physiological processes at the organism level. We acknowledge a linkage between metabolic processes and growth rates, but our consideration here is the observed distribution of the growth rates themselves rather than a theoretical model of how they change with temperature [5]. Since the relationships we identify are based on observed data we consider that they provide a firmer foundation for further work. We call the distribution of these rates the ‘biokinetic spectrum for temperature’.

## The Spectrum

We describe the biokinetic spectrum for temperature, define various terms to describe aspects of the data, and propose a statistical method for describing the spectrum along with the fitting procedure and the results. We then explain how we use a thermodynamic model of growth to model the spectrum.

### Spectrum Characteristics

**Fig 1:**
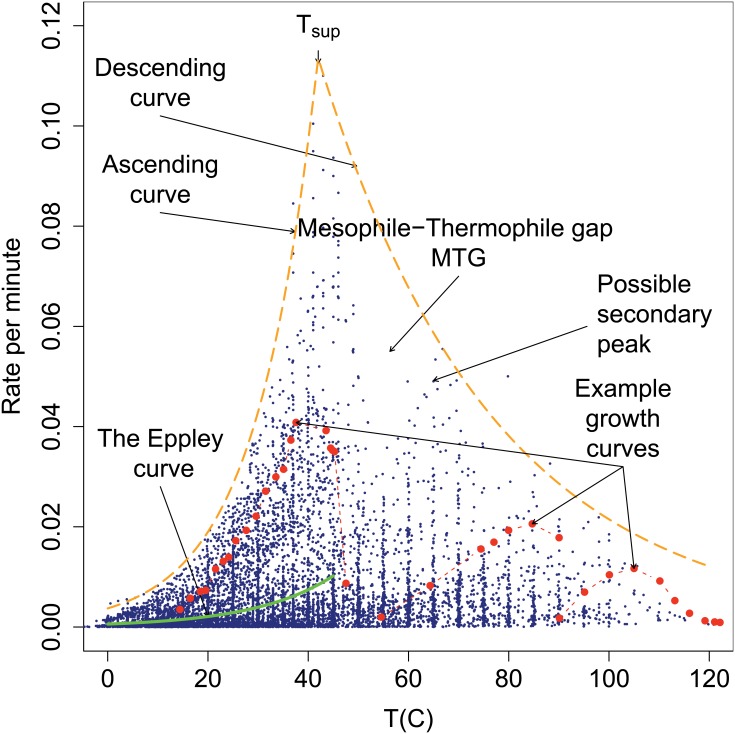
The biokinetic spectrum for temperature. The observed rate of growth for all 1627 strains versus temperature consisting of 10956 data points. We highlight as a visual indication the distribution of the data using dashed lines labeled ascending curve and descending curve. We indicate the location of the Mesophile-Thermophile Gap (MTG) described in the text and of a possible secondary peak. We also show an examples of growth curves for three strains (dashed red), and the curve described by Eppley [8] (solid green) and over the same temperature range he used. The inset shows a histogram of *T*_opt_ of the strains.

We began by collating the data shown in Fig 1 from the peer-reviewed literature. We collated the data by conducting intensive and regular searches to locate studies reporting temperature-dependent growth rates. The data consist of a collation of data sets for organisms grown or cultured at different temperatures. We use the word strain rather than species or taxa. This is because some data sets are of a single species grown under different conditions, or the same species grown by different researchers. The strains are largely unicellular organisms but there are also some multicellular organisms. There were 1627 strains represented and a total of 10956 observations. Many strains were represented by growth curves consisting of multiple points, while other strains appeared only as a single data point. We scaled all the growth rates to the same units, which was growth per minute versus temperature in Celsius. It was not our intention to obtain a data set that contained a random sample of strains since that was not, in any case, possible when considering the whole of life. Instead, we aimed to include as wide a range of strains as possible. This meant that we did not eliminate strains grown in suboptimal conditions and that we were more likely to include culturable strains of economic, veterinary, agricultural, or medical importance. Since random data were unavailable we proceeded by examining the statistical structure of the data.

Most striking was the Δ-shaped distribution manifested by the set of points representing the highest growth rate at each temperature, below which are strains growing at varying, but slower, rates. Across a range of temperatures (Fig 1), these maximum growth rates form the biokinetic spectrum for temperature. We are unaware of data covering the *whole* temperature range over which life is known to exist being presented in this manner elsewhere. The distinctiveness of the Δ-shape and the sharpness of its edge suggested that we were not likely missing very many fast-growing strains. If the data were very incomplete at the upper boundary then we would have expected a fuzzier edge. We made considerable efforts to locate data for strains that grew above the upper boundary. Since we found none we speculate that such strains do not exist.

The upper boundary exhibited a rapid curvilinear rise starting from a lower limit that began below 0°C and reached a surprisingly sharp peak at about 42°C. We defined a new cardinal temperature to refer to the maximum observed growth rate called *T*_sup_, where ‘sup’ is short for supremum. The *T*_sup_ represents the temperature at which the fastest known growth rate occurs and was largely due to a single strain of *Clostridium perfringens*. At temperatures above *T*_sup_ the boundary declined at a lesser rate to eventually approach zero above 120°C. We term the boundary for the maximum rates at temperatures below *T*_sup_ the ‘ascending curve’, and the boundary above it the ‘descending curve’. Since the maximum rate of growth of any organism declines above *T*_sup_, theories that assume that growth rates always increase with rising temperatures must be incorrect or incomplete.

The strains in Fig 1 are represented by one or more points. The figure shows examples of three strains with growth curves consisting of multiple data points. Also shown is the envelope identified by Eppley [8] for phytoplankton growth rates, which is discussed later.

We indicate in the figure that at about 50°C there appears to be a gap that interrupts the descending curve. We refer to this as the Mesophile-Thermophile Gap (MTG) since it lies on the boundary between these thermal groups. We made considerable efforts without success to locate data sets within the MTG. It was unclear from examining the figure alone if the gap might have resulted from (*a*) sampling insufficiency, (*b*) attenuation of growth rate, (*c*) rarity of suitable environments, or (*d*) difficulty in culturing strains. In the inset to Fig 1 we calculated a histogram of strain *T*_opt_, which also showed a reduced frequency at about 50°C. In summary, there was both an under-representation of strains and a reduction in the maximum rates at about 50°C. If the MTG were to be regarded as real, and not a statistical anomaly, then growth rates must increase at temperatures above it. This increase would continue then until a secondary peak located at about 67°C is reached. At still higher temperatures the maximum rates of growth decline further, eventually reaching detection limits.

### Motivating the Model

Previously we have shown that based on the assumption that temperature-dependent growth rates of cells were limited by a single rate-limiting Master Reaction System (MRS) we could accurately model the relative growth rates of strains in all three Domains (Bacteria, Archaea, Eukarya) [9]. The MRS maintains that there is a single, rate-limiting, enzyme-catalyzed reaction that limits growth rate. The same model also well described relative growth rates for multicellular poikilothermic organisms and obtained relationships between thermodynamic parameters that were consistent with expectations from biochemistry [10]. That work bridged biochemistry and whole organism biology and provided strong support for a MRS common to all life.

Although the growth rates of individual strains were modeled well by the thermodynamic model, the Δ-shape distribution of the data did not appear to be implicit in the model. Nevertheless, if there were a single rate-limiting reaction common to all life then its upper rate limit would also be universal. This suggests that it should be possible to obtain the Δ-shaped boundary from the thermodynamic model by imposing additional assumptions. We hoped that a mathematical description of the Δ-shaped limit might result in insight into the nature of the MRS.

However, it would have been a mistake to have derived a mathematical description of the edge that formed the Δ-shaped limit based simply on the outlying points since this would have amounted to ‘cherry-picking’ data. Instead, we developed a statistical approach using quantile regression and a modeling assumption, which was that the quantiles depended on a function of temperature. A quantile is a quantity below which a specific proportion of data fall. The objective of the quantile regression model was to summarise the data and explore any structure that exists with them. Once we obtained the parameters of this function we hoped to relate them to the parameters of the thermodynamic model. This would allow us to connect the thermodynamics of cell processes to the distribution of rate of growth as well as the limiting growth rate of life on Earth. These are the objectives of this paper.

### Modeling the Spectrum

To describe the distribution of such rates we used Bayesian quantile regression (see Methods). A quantile regression obtains a fitted line below which a particular proportion of the data is to be found. The quantiles could be thought of as describing the distribution of rates below the boundary and given sufficient data, could come as close as desired to the upper limit of growth. Quantile regression has been used elsewhere to describe plankton specific growth rates [11] but that study made use of a frequentist approach and only described an ‘ascending curve’. We report here, we believe for the first time, a description of a much wider range of data that also shows a descending curve and investigates the distribution of rates beneath the ascending and descending curves.

Eppley [8] calculated a power curve to fit an envelope to the upper limit of temperature-dependent growth rates of mixed phytoplankton assemblages. We chose to follow this approach and also fitted a power curve to the ascending curve. From examination of the data in Fig 1 we considered that another power curve might well describe the descending curve. We do not maintain that this is the only function that might be used nor that it has any biological significance, but it has the merits of simplicity and symmetry. The ascending and descending curves have the same form so their parameters have equivalent interpretations. The function is given by Eq 1 in which *r* is the rate of growth (per minute), *T* is the temperature (°C), *a*, *b*, *c*, *d* are parameters to be estimated, and *T*_sup_ can be derived from the parameters (see Methods). The rate of growth, *r*, is proportional to the reciprocal of the generation time. The parameters *b* and *d* control the rate of ascent and descent, respectively. A larger positive *b* value corresponds to a steeper response to temperature change whereas a lower positive value indicates a shallower response. A more negative *d* corresponds to a steeper decline in growth rate with increasing temperature and a smaller negative *d* indicates a lesser decline. We refer to this as the quantile curve model and the fitted curves as quantile curves.

$$r=\left\{\begin{array}{cc} \exp(a+b\times T) & T\leq T_{\text{sup}}\text{,}\;b>0\\ \exp(c+d\times T) & T>T_{\text{sup}}\text{,}\;d<0 \end{array}\right.$$ (1)

## Results

We proceeded in several steps. First we fitted the quantile curves to the entire data set, then investigated whether there were subgroups of organisms with different quantiles curves at each temperature, and whether the curves depended on trophic or metabolic status. Having modeled the spectrum we then attempted to predict the form of the spectrum from our thermodynamic model.

### Quantile curves

We fitted the quantile curves within a Bayesian framework so that the results could be integrated with our previously developed Bayesian thermodynamic model for temperature-dependent growth rates [10]. We fitted curves for quantiles 50, 60, 70, 80, 90, 92.5, 95, 97.5%. We did not select higher quantiles since these become more subject to error as 100% was approached. We also did not select lower quantiles since we were interested primarily in growth rates that defined the shape of the spectrum.

**Fig 2:**
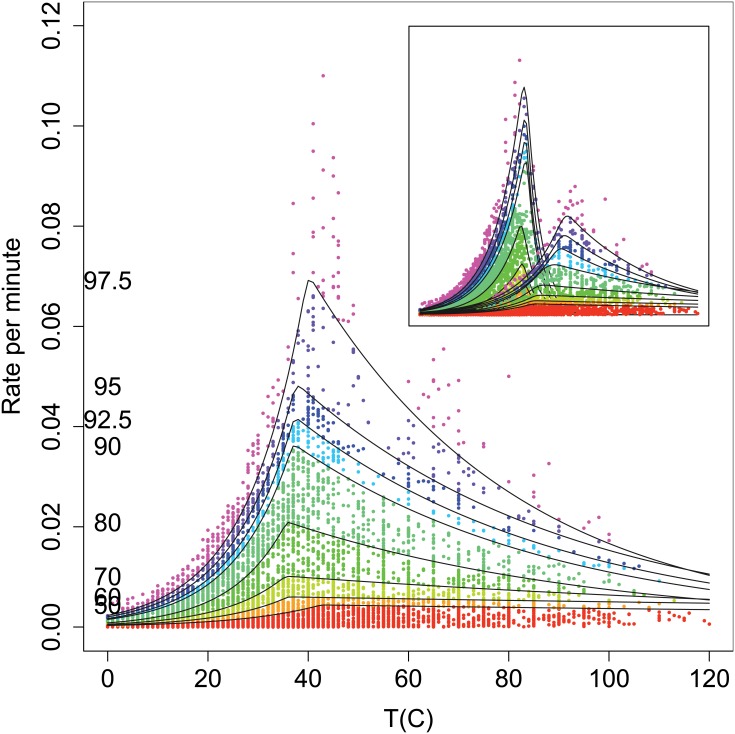
Fitted quantile curves. The observed rate of growth for the 10956 data points strains versus temperature and posterior mean fits for the quantiles. The percentile values per curve are shown on the left hand side. We show the fitted quantile curves as black lines and the data as points coloured according to the quantile in which they appeared. The inset shows the observed rate of growth for those strains with *T*_opt_ ≤ 50°C (left) *T*_opt_ > 50°C (right) with fitted quantile curves for each case.

We show in Fig 2 the fitted quantile curves and give the coefficients in Table 1. Examination of the quantile curves indicated that they conformed to the data on the ascending curve up to *T*_sup_. The temperatures at which the quantile curves peaked did not deviate greatly from the *T*_sup_ observed from the data. This meant that even if the fastest growing strains such as *Clostridium perfringens* were deleted the resulting quantiles would still have peaks at about the same temperature. This agreement of the peaks in successive quantiles suggests that a transition of some nature occurs at about this temperature and which is common to all life, or perhaps, speculatively, involves an environmental influence, such as the structure of water. We also noted that the Eppley curve [8] closely corresponded to the 60% quantile curve (not shown). Although not as visually impressive as the ascending curves, the descending quantile curves conformed to the data and served to emphasize the MTG. This suggested that if the MTG was real then there were actually two groups of strains, one predominantly below approximately 50°C and another above. Therefore, we also fitted another set of quantile curves for strains with *T*_opt_ ≤ 50°C and *T*_opt_ > 50°C. The *T*_opt_ is the temperature at which an individual strain grows most rapidly. We show the resulting quantiles in the inset in Fig 2 and the coefficients in Table 2. The conformation of these curves appeared to be a better visual match to the observations. However, the quantile curves also appeared to differ radically in shape between the two groups.

**Table 1:** Quantile curve parameters for the whole data set.

Quantile (%)	a	sd(a)	b	sd(b)	c	sd(c)	d	sd(d)
50	-8.085	0.012	0.062	0.0005	-5.293	0.046	-0.003	0.001
60	-7.846	0.015	0.076	0.0008	-5.012	0.034	-0.003	0.001
70	-7.484	0.024	0.082	0.0012	-4.331	0.079	-0.007	0.001
80	-7.095	0.027	0.090	0.0012	-3.288	0.054	-0.016	0.001
90	-6.492	0.020	0.086	0.0010	-2.600	0.071	-0.019	0.001
92.5	-6.378	0.022	0.086	0.0010	-2.465	0.071	-0.019	0.001
95	-6.194	0.024	0.085	0.0011	-2.324	0.111	-0.019	0.002
97.5	-6.065	0.011	0.085	0.0006	-1.692	0.121	-0.024	0.002

**Table 2:** Quantile curve parameters for strains with *T*_opt_ ≤ 50 or *T*_opt_ > 50.

Quantile (%)	a	sd(a)	b	sd(b)	c	sd(c)	d	sd(d)
*T* ≤ 50: 50	-8.100	0.012	0.064	0.0005	3.394	1.297	-0.181	0.026
*T* ≤ 50: 60	-7.827	0.013	0.075	0.0006	5.248	1.196	-0.210	0.024
*T* ≤ 50: 70	-7.517	0.024	0.085	0.0012	5.131	1.614	-0.199	0.031
*T* ≤ 50: 80	-7.037	0.034	0.088	0.0015	4.925	1.769	-0.183	0.034
*T* ≤ 50: 90	-6.441	0.017	0.083	0.0007	7.453	2.006	-0.220	0.039
*T* ≤ 50: 92.5	-6.327	0.027	0.083	0.0011	6.997	1.713	-0.208	0.034
*T* ≤ 50: 95	-6.153	0.021	0.082	0.0008	6.454	2.428	-0.193	0.048
*T* ≤ 50: 97.5	-6.057	0.010	0.085	0.0006	5.517	2.857	-0.170	0.056
*T* > 50: 50	-8.438	0.239	0.064	0.0063	-5.104	0.111	-0.005	0.002
*T* > 50: 60	-8.316	0.299	0.067	0.0078	-4.899	0.095	-0.004	0.001
*T* > 50: 70	-8.021	0.283	0.065	0.0073	-4.529	0.116	-0.005	0.002
*T* > 50: 80	-7.905	0.319	0.070	0.0085	-3.948	0.206	-0.007	0.003
*T* > 50: 90	-7.446	0.378	0.067	0.0091	-3.059	0.398	-0.013	0.005
*T* > 50: 92.5	-7.130	0.278	0.061	0.0062	-2.323	0.412	-0.020	0.005
*T* > 50: 95	-6.947	0.227	0.059	0.0048	-1.954	0.465	-0.022	0.006
*T* > 50: 97.5	-6.821	0.229	0.059	0.0051	-1.512	0.541	-0.025	0.007

To quantify the apparent visual difference in shapes for the two groups we compared the values of the ascending and descending parameters (*b* and *d*) for strains with *T*_opt_ ≤ 50°C to those strains with *T*_opt_ > 50°C. The 99% CIs for their differences in Table 3 indicated that the *b* parameters generally did not differ between the two temperature groups for lower quantiles (below 92.5%), but, by contrast, all but one of the *d* parameters differed between the two temperature groups. This meant that the maximum rate of increase for biological growth had a fixed value for all quantiles below the 95% quantile limit at all temperatures. The difference in the *d* parameters suggests a limiting mechanism that differed between the two groups. Since we observed a difference in the two groups we suspected that the MTG was not in fact a sampling insufficiency or where strains were less likely to be found, but actually was a region where growth was attenuated. We considered it to be a transition region that separated two groups of organisms, one group with *T*_opt_ ≤ 50°C and another with *T*_opt_ > 50°C.

**Table 3:** CIs for differences of *b* and *d* for *T*_opt_ ≤ 50 and *T*_opt_ > 50. Table of 99% credible intervals (CIs) for differences of *b* and *d* between strains with *T*_opt_ ≤ 50 and *T*_opt_ > 50. A 99% CI that includes zero indicates that there is a 99% probability that the two parameters do not differ.

Quantile (%)	$b_{T_{\text{opt}}}\;\leq \;50\;-\;b_{T_{\text{opt}}}\;>\;50$		$d_{T_{\text{opt}}}\;\leq \;50\;-\;d_{T_{\text{opt}}}\;>\;50$	
50	-0.02	0.01	-0.24	-0.12
60	-0.02	0.02	-0.26	-0.13
70	-0.00	0.03	-0.27	-0.13
80	-0.01	0.03	-0.26	-0.11
90	-0.01	0.03	-0.32	-0.08
92.5	0.00	0.03	-0.27	-0.08
95	0.00	0.03	-0.29	-0.06
97.5	0.01	0.03	-0.27	0.01

**Fig 3:**
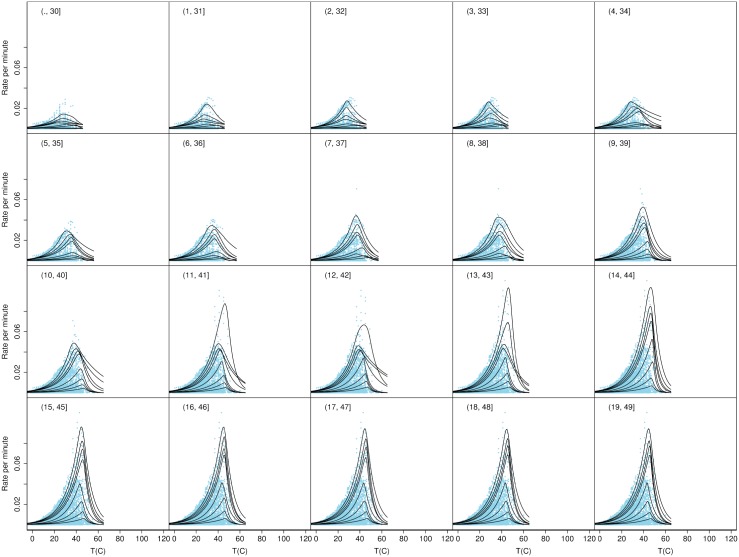
Quantile curves for temperature bins ≤30, …, 19–49°. Shown are the observed rates for all strains plotted as separate overlapping bins based on the observed strain *T*_opt_. The figure also shows the fitted quantile curves for temperature bins ≤30, …, 19–49°.

**Fig 4:**
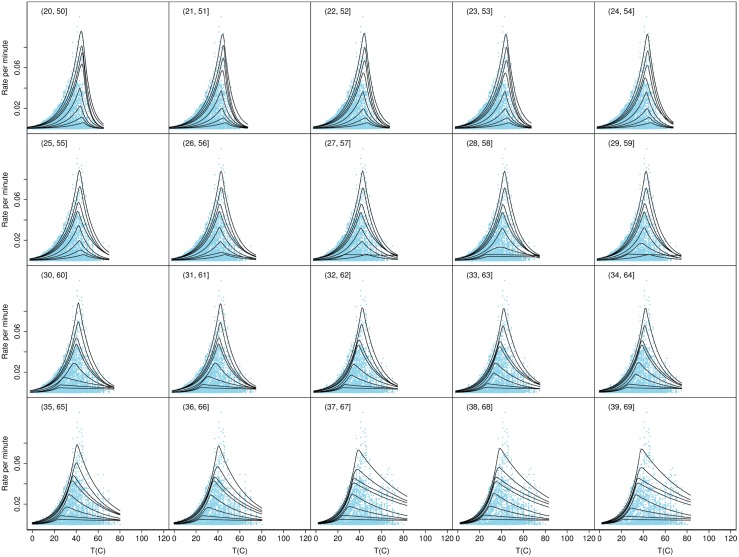
Quantile curves for temperature bins 20–50, …, 39–69°. Shown are the observed rates for all strains plotted as separate overlapping bins based on the observed strain *T*_opt_. The figure also shows the fitted quantile curves for temperature bins 20–50, …, 39–69°.

**Fig 5:**
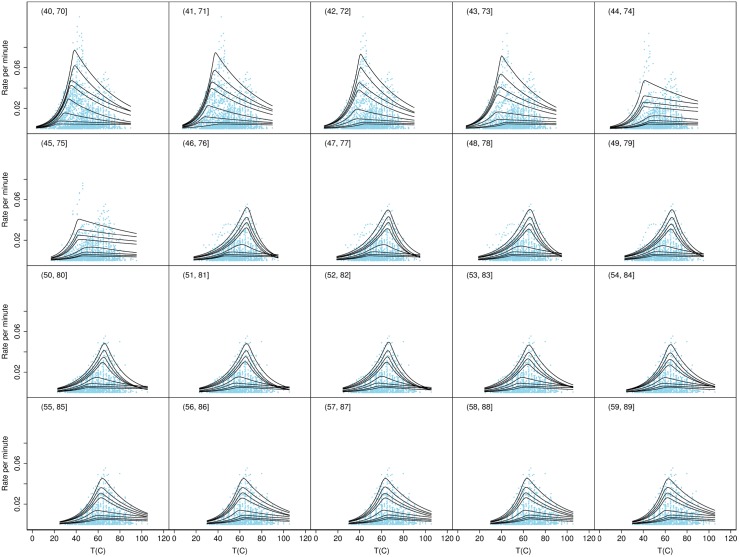
Quantile curves for temperature bins 40–70, …, 59–89°. Shown are the observed rates for all strains plotted as separate overlapping bins based on the observed strain *T*_opt_. The figure also shows the fitted quantile curves for temperature bins 40–70, …, 59–89°.

**Fig 6:**
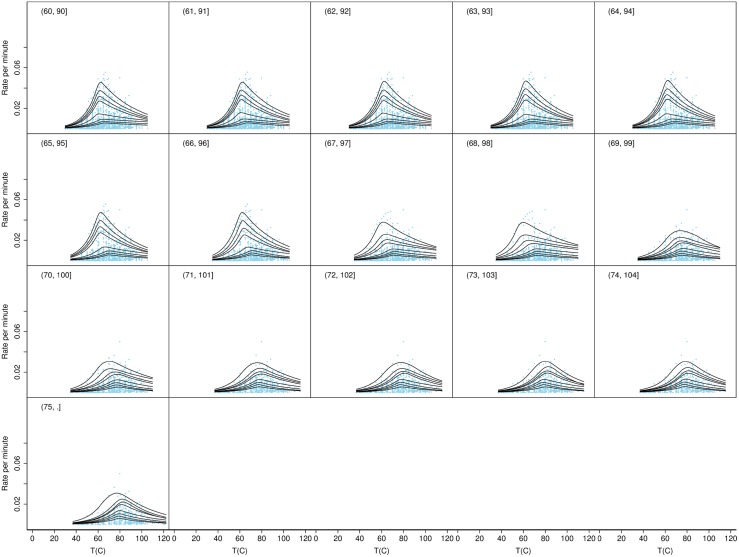
Quantile curves for temperature bins 60–90, …, >75°. Shown are the observed rates for all strains plotted as separate overlapping bins based on the observed strain *T*_opt_. The figure also shows the fitted quantile curves for temperature bins 60–90, …, >75°.

To test if there may have been additional groups present with growth rates that varied on narrower temperature ranges we calculated the quantiles for a series of overlapping temperature ranges each of width 30 degrees and each displaced by 1 degree above the previous one, as shown in Figs 3, 4, 5 and 6. These figures show the consistency of the fitted quantile curves over small temperature intervals on each side of the MTG. A width of 30 degrees ensured a range of strains would be included in each. The quantile curves conformed well to the data up to the lower bound of 37°C. Thereafter the secondary peak intruded which caused the quantiles to stretch out. From 46°C onwards the quantiles again more naturally fitted the data. Trends in the quantile curve parameters indicated the effect of the MTG as it was gradually included in bins and then excluded again, suggesting that the MTG represented an actual biological transition rather than a statistical anomaly.

**Fig 7:**
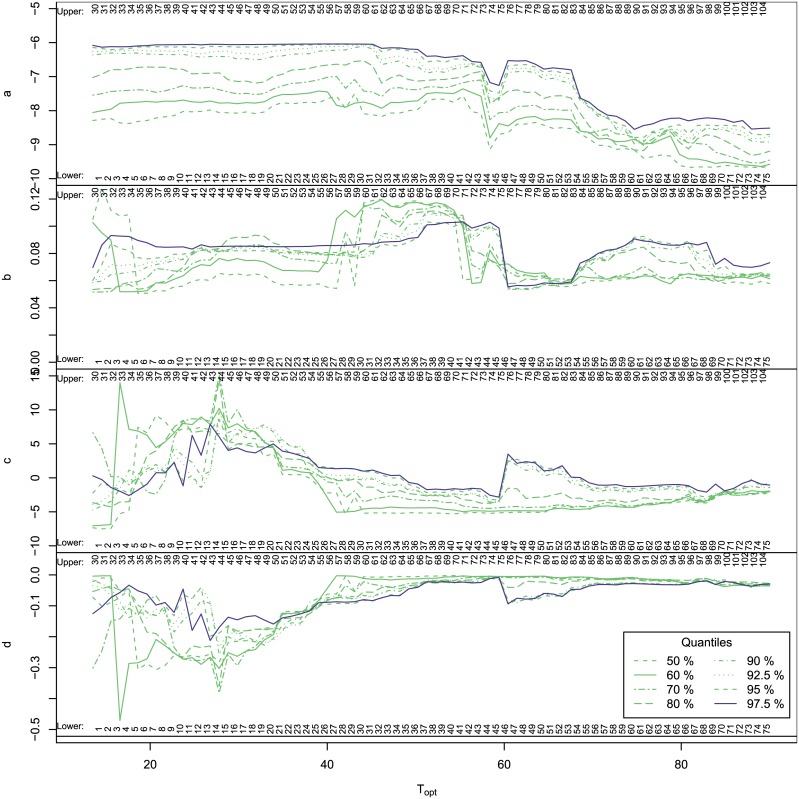
Thermodynamic parameter trends. The parameter values for quantile curves versus the midpoint of the temperature bin. The posterior mean values are shown according to their respective quantiles. The numbers at the top and bottom of each plot are the temperature bins for the plots shown in Figs 3–6. For simplicity we concentrate on the 97.5% quantile. The 97.5% quantile for the *a* parameter remains steady until the upper bound reaches 60°C above which it declines until the lower bound reached 60°C; *b* remains steady until the upper bound reaches 75°C (lower bound 45°C) above which it declines until the lower bound reaches 60°C; *c* declines gradually until the upper bound reaches 75°C (lower bound 45°C) above which it rose, then dips between 60°C and above; while *d* rises to a peak at 33°C (lower bound 7.5°C) then dips until 42.5°C (lower bound 12.5°C) above which it rises, then remains steady after 60°C (lower bound 30°C).

We plotted the quantile curve parameters for the same ranges in Fig 7. Although complex, examination of their trends proved to be of interest. Below, the *b* parameter can be related to the how fast the maximum growth rates increased with increase in temperature, while the *d* parameter corresponded to how rapidly the maximum growth rates reduced with increasing temperature. To simplify the interpretation we just discuss here the 97.5% quantile. The 97.5% quantile for the *a* parameter remained steady until the upper bound reached 60°C above which it declined until the lower bound reached 60°C; *b* remained steady until the upper bound reached 75°C (lower bound 45°C) above which it declined until the lower bound reaches 60°C; *c* declined gradually until the upper bound reached 75°C (lower bound 45°C) above which it rose, then dipped between 60°C and above; while *d* rose to a peak at 33°C (lower bound 7.5°C) then dipped until 42.5°C (lower bound 12.5°C) above which it rose, then remaining steady after 60°C (lower bound 30°C). These patterns indicated the effect of the MTG as it was gradually included in bins and then excluded again and suggested that the MTG indeed represented an actual biological transition. The MTG is a region that separates psychrophile-mesophile and thermophile-hyperthermophile strains, each of which groups is internally consistent but differed one from another.

**Fig 8:**
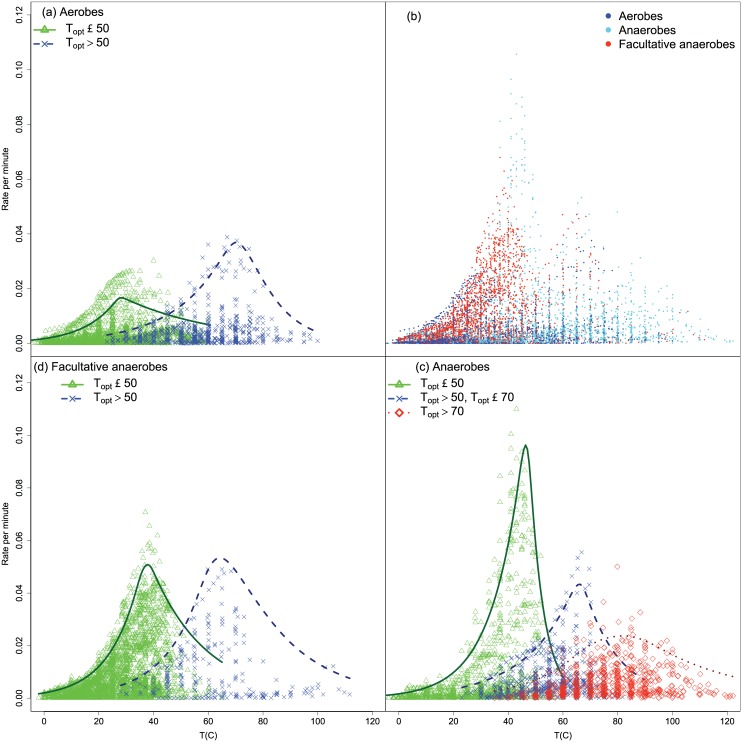
Fitted quantile curves for strains by respiration status. Fitted quantile curves for strains by respiration status. Shown are the observed data and 95% quantile curves for each group.

We then examined whether the results might vary by metabolic status or trophic status (Fig 8). After grouping the strains by *T*_opt_ ≤ 50 and *T*_opt_ > 50 the descending curve showed two minor peaks with one consisting almost entirely of anaerobes so we also divided the anaerobes into two further groups. Examination of the *b* and *d* parameters (Table 4) showed that, after disregarding differences between the ≤ 50 and > 50 groups, that *b* parameter had very few significant differences apart from the ≤ 50 group between the aerobes having a slightly lower maximum rate (mean *b* = 0.0833) than facultative anaerobes (mean *b* = 0.0897). For the *d* parameter there was only one significant difference. In summary, we could not distinguish strains on the basis of respiration.

**Table 4:** Probabilities that *b* or *d* are equal by respiration status. Shown is a symmetric matrix of the probabilities that *b* or *d* are equal for respiration statuses for the 95% quantile. Those probabilities less than 0.01 are bolded.

	Respiration group						
	Aerobes		Anaerobes			Facultative Aerobes	
	*T*_opt_ ≤ 50	*T*_opt_ > 50	*T*_opt_ ≤ 50	50 < *T*_opt_ ≤ 70	*T*_opt_ > 70	*T*_opt_ ≤ 50	*T*_opt_ > 50
Parameter *b*:							
${\text{A}}_{T_{\text{opt}}}\;\leq \;50$	1	**0.004**	**0**	**0**	0.196	0.076	0.016
${\text{A}}_{T_{\text{opt}}}\;>\;50$	**0.004**	1	0.016	0.602	0.524	**0.008**	0.184
${\text{An}}_{T_{\text{opt}}}\;\leq \;50$	**0**	0.016	1	**0.002**	0.748	0.084	0.150
${\text{An}}_{50}\;<\;T_{\text{opt}}\;\leq \;70$	**0**	0.602	**0.002**	1	0.431	**0**	0.102
${\text{An}}_{T_{\text{opt}}}\;>\;70$	0.196	0.524	0.748	0.431	1.000	0.283	0.664
${\text{FA}}_{T_{\text{opt}}}\;\leq \;50$	0.076	**0.008**	0.084	**0**	0.283	1	0.102
${\text{FA}}_{T_{\text{opt}}}\;>\;50$	0.016	0.184	0.15	0.102	0.664	0.102	1.000
Parameter *d*:							
${\text{A}}_{T_{\text{opt}}}\;\leq \;50$	1	0.096	**0.006**	0.116	0.618	0.427	0.395
${\text{A}}_{T_{\text{opt}}}\;>\;50$	0.096	1.000	0.407	0.698	0.162	0.502	0.471
${\text{An}}_{T_{\text{opt}}}\;\leq \;50$	**0.006**	0.407	1	0.293	0.028	0.283	0.194
${\text{An}}_{50}\;<\;T_{\text{opt}}\;\leq \;70$	0.116	0.698	0.293	1.000	0.220	0.602	0.572
${\text{An}}_{T_{\text{opt}}}\;>\;70$	0.618	0.162	0.028	0.220	1.000	0.590	0.389
${\text{FA}}_{T_{\text{opt}}}\;\leq \;50$	0.427	0.502	0.283	0.602	0.590	1.000	0.751
${\text{FA}}_{T_{\text{opt}}}\;>\;50$	0.395	0.471	0.194	0.572	0.389	0.751	1.000

**Fig 9:**
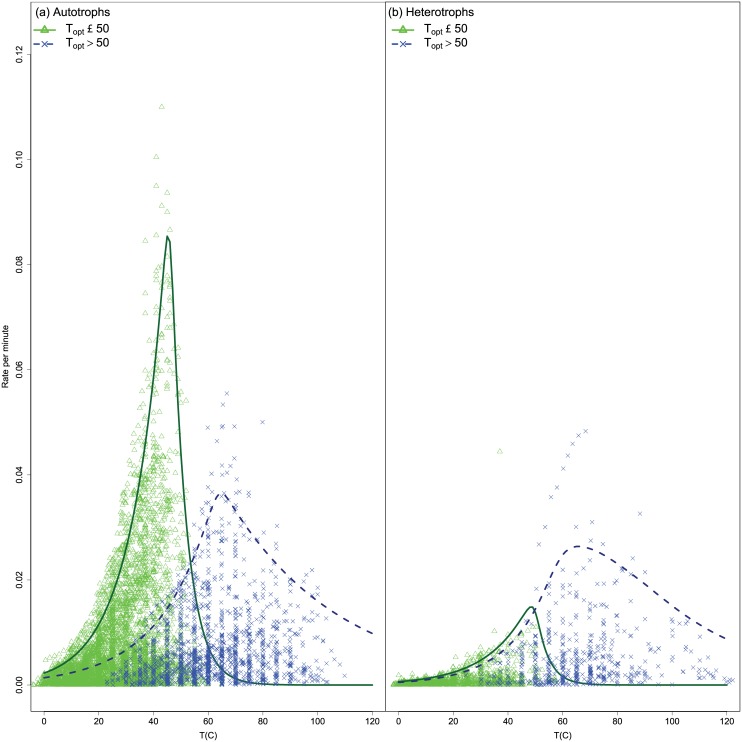
Fitted quantile curves for strains by trophic status. Shown are the observed data and the 95% quantile curves.

In Fig 9 we show the quantiles for autotrophs and heterotrophs. As shown in Table 5 the *b* parameter differs significantly between autotrophs (mean *b* = 0.0669) and heterotrophs (mean *b* = 0.0812) for the lower temperature group. This means that the upper maximal response is slightly greater in heterotrophs than autotrophs, but since the *d* parameter does not differ they share the same upper limit in the declining phase. Therefore we could distinguish strains on the basis of trophic status, which implied that the limiting mechanism for the descending curve might differ between the trophic groups.

**Table 5:** Probabilities that *b* or *d* are equal by trophic status. Probabilities that *b* or *d* are equal by trophic status for the 95% quantile. Those probabilities less than 0.01 are bolded.

	Trophic group			
	Autotrophs		Heterotrophs	
	*T*_opt_ ≤ 50	*T*_opt_ > 50	*T*_opt_ ≤ 50	*T*_opt_ > 50
Parameter *b*:				
${\text{A}}_{T_{\text{opt}}}\;\leq \;50$	1	0.267	**0**	0.068
${\text{A}}_{T_{\text{opt}}}\;>\;50$	0.267	1.000	0.487	0.076
${\text{H}}_{T_{\text{opt}}}\;\leq \;50$	**0**	0.487	1	**0.006**
${\text{H}}_{T_{\text{opt}}}\;>\;50$	0.068	0.076	**0.006**	1
Parameter *d*:				
${\text{A}}_{T_{\text{opt}}}\;\leq \;50$	1.000	0.088	0.443	0.014
${\text{A}}_{T_{\text{opt}}}\;>\;50$	0.088	1.000	0.050	0.710
${\text{H}}_{T_{\text{opt}}}\;\leq \;50$	0.443	0.050	1.000	0.024
${\text{H}}_{T_{\text{opt}}}\;>\;50$	0.014	0.710	0.024	1.000

**Fig 10:**
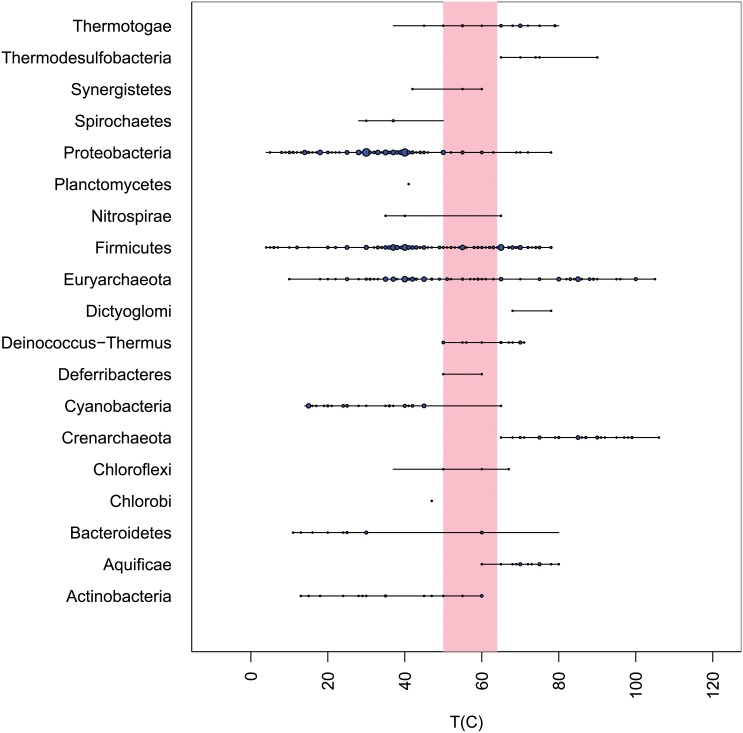
Shown are the occurrences of microbial phyla by *T*_opt_. The horizontal lines indicate the temperature span of each phylum. The circles indicate the occurrence of strains at each temperature and their diameters are proportional to the square root of their prevalence. The vertical block indicates the approximate position of the MTG.

We show in Fig 10 the prevalence of microbial strains within phyla at each temperature along with the approximate location of the MTG. There were no obvious association of microbial phyla with the MTG (Fig 10). The spans of several phlya terminated within the MTG but only one was confined to it (Deferribacteres). The most populous within the MTG are Firmicutes and Euryarchaeota.

From the above we concluded that we could describe the biokinetic spectrum using quantile curves, that the MTG was an actual biological transition that separated two groups, and that the two groups differed according to trophic status. However, in order to recover the Δ-shape from the thermodynamical MRS model we chose to treat the spectrum as a whole rather than in two parts. We describe this in the next section.

### Relation to Thermodynamic Properties

The MRS assumption, that growth rates are limited by a single reaction system common to all life, made in our earlier studies [9, 10], might appear to be excessively strong. However, Occam’s razor requires us to choose a simpler model over a complex alternative where each is equally effective. Our thermodynamic model only contains eight distinct parameters of which four are global and four parameters are assigned to each strain, one of which is a scaling parameter. With these we have previously well described growth curves for 230 strains [10]. Despite the obvious complexity of cellular processes our relatively simple model could successfully describe growth rates. This might have been because: (*a*) the MRS assumption is correct; (*b*) some coordination occurs within metabolic systems, such as an adaptation for similar temperature dependencies of constituent enzymes [12–14]; (*c*) a metabolic-controlling mechanism acts to regulate steps in metabolic systems [15]; or (*d*) a sequence of reactions fails one by one at increasing temperatures, the collective result of which appears as a single reaction. If we could obtain the overall Δ-shape from the thermodynamic model we might throw light on such alternatives.

**Fig 11:**
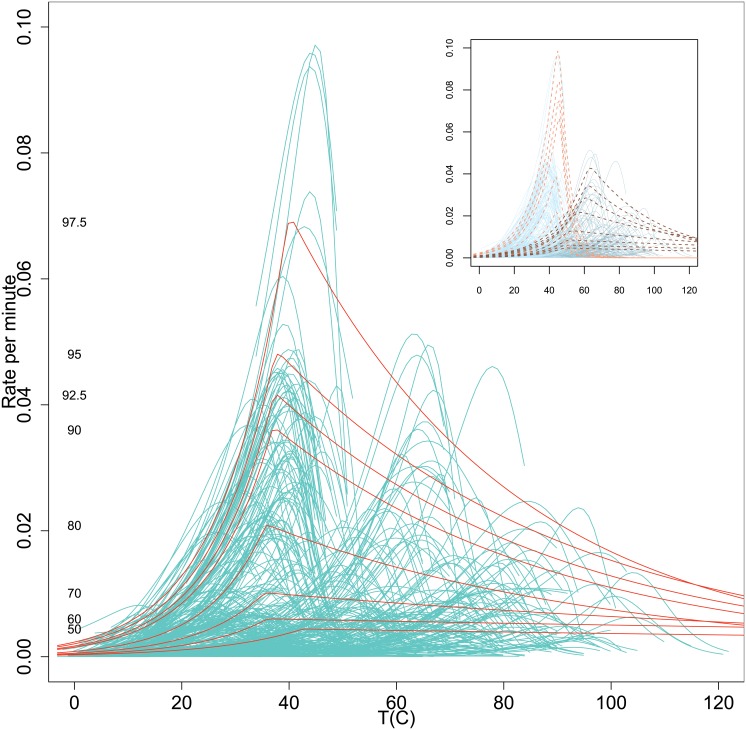
Over-plotted fitted growth curves. Over-plotted fitted growth curves from the thermodynamic model for the 694 strains that had at least 5 data points and well defined peaks and with quantile curves for all the 10956 data points. The quantiles are the same as in Fig 2 and are calculated from the observed data. The inset shows the same fitted thermodynamic curves coloured differently for those strains with *T*_opt_ ≤ 50 or *T*_opt_ > 50 along with the corresponding quantile curves for each group.

**Fig 12:**
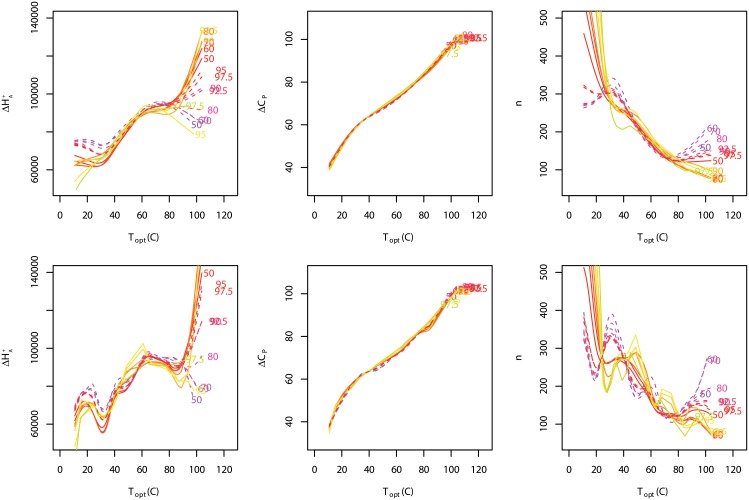
Thermodynamic parameters trends. Trends in the mean thermodynamic parameters ($\Delta H_{A}^{‡}$, Δ*C*_*P*_ and *n*) for strains above (solid lines) and below (dashed lines) quantile curves versus temperature. Curves are cubic-spline smoothed with df = 5 on the top row and df = 10 on the bottom row.

**Fig 13:**
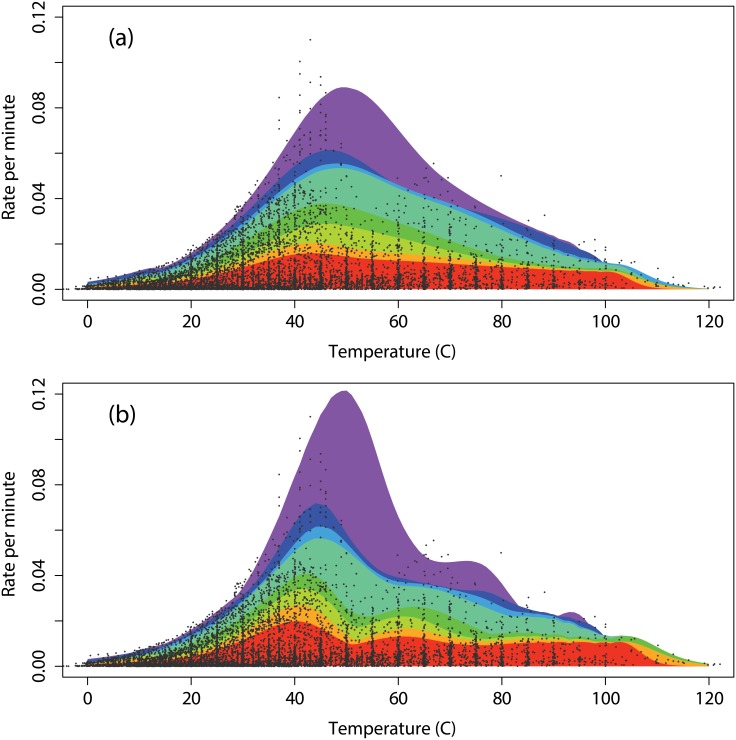
Predicted spectra and observed data. Shaded areas are the predicted limits for the quantiles 50, 60, 70, 80, 90, 92.5, 95, 97.5 generated by the thermodynamic model and assuming smooth trends in the thermodynamic parameters. The observed data are indicated by black dots. The upper plot (a) is for smoothed parameters with df = 5 and the lower plot (b) is for df = 10.

To see if we could obtain the overall Δ-shape from the thermodynamic model we began by using it to fit growth curves for all suitable strains. Of the eight distinct thermodynamic parameters four are the main focus of this communication: *c*, a scaling constant; $\Delta H_{A}^{‡}$, the enthalpy of activation (J/mol) of the ‘master reaction’; Δ*C*_*P*_, the heat capacity change on denaturation (J/K mol-amino acid residue) of the rate-controlling enzyme; and *n*, the number of amino acid residues. Another parameter that canbecalculated from the model is *T*_mes_, the temperature of maximum enzyme stability, at which the putative enzyme in the MRS is least likely to be denatured. We simultaneously fitted growth curves for all suitable strains (Figures 1–44 in S1 File) and calculated their thermodynamic parameters (S1 Table). We show the over-plotted growth curves in Fig 11.

The quantile curves were calculated from the complete data set while the individual growth curves were calculated for strains with at least 5 data points and which had distinct peaks. It was clear that the fitted curves for some strains exceeded some quantile curves while others did not. In order to simplify the results below we define two terms. We refer to strains that exceed the quantile curves as ‘exceedance strains’ and the others as ‘non-exceedance strains’. The exceedance strains could be thought as exhibiting relatively faster growth compared to non-exceedance strains. We calculated the mean thermodynamic parameters of the exceedance and non-exceedance strains for each quantile for a series of narrow temperature bins. We show the posterior means of $\Delta H_{A}^{‡}$, Δ*C*_*P*_ and *n* for exceedance and non-exceedance strains in Fig 12. We omit the *C* parameter in the figure since it is simply a scaling parameter. We do not consider those parameters to be independent, either in a biological or a statistical sense. Since there were only a few growth curves that exceeded the upper quantiles we smoothed the trends to highlight the posterior trends with temperature. The plots show the mean trends and not their variabilities, which, in any case, are considerable, as is visually evident from the sensitivity of the curves to smoothing.

Since the trends shown in Fig 12 have been previously reported by us [10] we do not describe them in detail here. Our purpose was not to compare the thermodynamic model to experimental data on isolated proteins, but rather, to show the observed trends of the model parameters that were obtained from growth curves fitted using the thermodynamic model. Notwithstanding the above point, a comment on *n* is warranted. There is no doubt that there was a trend when *n* was plotted against temperature and that *n* decreased with increasing thermophilicity. The declining trends of *n* are consistent with negative correlations of protein length with temperature observed by others [16–20]. However, there was great deal of variability of *n* at lower temperatures. This was partly a result of attempting estimation of model strains from the highest quantiles for which there were the least amount of data available, but was also evident in previous estimates using all the data [10]. There were surprisingly low values at high temperatures. There is no reason why these particular values for *n* should match the mean lengths for proteins in cells. Our results refer to a particular putative protein in the MRS rather than the average length of a protein in a cell. Protein lengths in cells are typically right-skewed [21], which will result in their mean being located somewhat to right of the median. Therefore, one would expect the average protein length to be higher than that of a randomly chosen protein. Since we observe this parameter through its putative effect on cell division rate, it is possible that *n* represents something more complicated then the number of amino acids in a protein within the MRS. It may be sensitive to other characteristics, such as the number of local or global entropic configurations, the number of amino acids involved in the reaction centre, or the number of nonpolar amino acids.

The posterior mean $\Delta H_{A}^{‡}$ for exceedance and non-exceedance strains generally rose with temperature (Fig 12), but, with the exception of the 97.5 and 99% curves, at about 40°C the mean $\Delta H_{A}^{‡}$ for exceedance strains rose above the non-exceedance strains. It should be noted that 40°C corresponded to the approximate location of *T*_sup_. The Δ*C*_*P*_ increased smoothly with temperature and there was no differentiation between quantiles or between exceedance strains and non-exceedance strains. However, *n* displayed the opposite pattern of quantiles to that of $\Delta H_{A}^{‡}$, although the ordering was more confused. We used the smoothed posterior exceedance group parameters and the thermodynamic model to predict growth curves for the temperature bins. We then calculated the envelopes that would enclose the ensemble of these fitted growth curves (Fig 13). The envelopes produced an obvious Δ-shaped appearance, and in the less smoothed case the MTG also appeared in the lower quantiles.

Without knowledge of the trends in the parameters we could not use the thermodynamic model, which describes the growth rate of single organisms, to reproduce the Δ-shape of the biokinetic spectrum that represents a very great number of organisms. However, we could achieve this extrapolation when provided with additional information. This we did by using smoothed trends of the thermodynamic parameters. The binning and smoothing are simply means of coping with the variability introduced when dealing with the necessarily small numbers of fast-growing organisms. In short, while the thermodynamic model could not of itself predict the maximum rate, by using the MRS and the smoothed thermodynamic parameter trends, we could recover the biokinetic spectrum.

## Discussion and Summary

We need to distinguish between rate limits and temperature limits. Discussions on limits of growth usually concern temperature extremes. The nature of the upper temperature limit for growth may be related to protein denaturation and thermophilic protein adaptations, membrane structure, or hydrolysis [22]. Growth rate may be limited by reduced availability of substrate but if the substrate is not limiting then growth rate may still be moderated by the capacity for substrate affinity [23]. These are separate considerations: a mechanism that determines the upper and lower temperature limits might not necessarily be a determinant of the upper rate limit. Clearly, temperature limits is a topic that could be informed by our data, but this study concerned the upper growth rates possible for any strain and without limitation, and the Δ-shape that revealed it. Importantly, we were able to reproduce the Δ-shape of the spectrum using a thermodynamic model based on the MRS.

In the thermodynamic model the parameters are characteristics of the putative rate-limiting enzyme assumed in the MRS. Considering the model parameters, the $\Delta H_{A}^{‡}$ can be viewed as the energy barrier that limits reaction rates, Δ*C*_*P*_ positively correlates with thermostability, and *n* negatively correlates with the size of the enzyme. As we have shown previously [9, 10] the protein thermostability temperature range of the MRS increases with temperature, which is consistent with observations by others [24]. While we report a positive trend of heat capacity change in the current manuscript we also show a decreasing trend with n. The joint effect of these parameters is to broaden the thermostability of the MRS. However, there are numerous possible mechanisms to promote protein thermostability [24]. The smoothed trends of these parameters are interpretable as an evolutionary trade-off between stability at higher temperatures and activity at lower temperatures, but interestingly, we can now see that the switchover of priority between protein activity and stability occurs at about *T*_sup_. We do not imply that proteins that are optimally active at high temperatures are necessarily more thermostable and active than proteins that are optimally active at lower temperatures, but that evolutionary pressure has resulted in a trade-off between thermostability and activity: while it is possible for a protein to be engineered to be active and stable, organisms need to have proteins that they are able to degrade [24]. In short, the form of the biokinetic spectrum, identified and defined herein, appears to be consistent with an evolutionary trade-off between activity and stability with changing temperature. However, we are still left with the MTG, which appears to require finely tuned variations in thermodynamic trends and these remain unexplained.

We do not claim that the biokinetic spectrum necessarily arises from the thermodynamic model, but that the form of the spectrum is not inconsistent with it. This means that the biokinetic spectrum is potentially understandable on the basis of cell physiology relating to protein denaturation, such as would occur within the putative MRS. Whether this particular hypothesis is valid is a matter still to be determined.

Falsity of the Δ-shape of the spectrum could be established by detecting a strain with a growth rate in excess of its upper bound. Some workers have speculated that life began on Earth more than once [25]. If still extant, detection of such life would be confounded by rarity and lack of knowledge of its metabolism unless they utilised resources conventional organisms cannot use. Since we lack knowledge of those resources we would have difficulty in detecting such life. A strain that exceeded the spectrum limits would have to possess a very unusual metabolism or adaptations. Our results here suggest a speculative possibility, which is that if an organism is found in exceedance of the biokinetic spectrum it may be a candidate for an alternative biogenesis. In a more traditional vein, we expect that this work will be of aid to systems biologists [26]. To be realistic their models must predict feasible growth rates, not just for single organisms, but for all life. The biokinetic spectrum provides a limiting constraint on the possible range of model predictions of growth at any temperature and may perhaps allay some reasonable doubts that have been expressed on the subject [27].

Since we show that the upper growth rate possible for all life has a distinct limit we suggest that this observation may have particular implications to ecology, physiology and climate change studies. For example, biological processes are often assumed to have an exponential relationship with temperature that continues without moderation [28]. The form of the spectrum suggests that biodiversity, physiology, and other temperature-dependent biological phenomena may be more complex than such models assume, or at least that the existence of an upper limit may moderate prediction of such relationships. We expect that the spectrum will provide underpinning principles for many models looking at the thermal responses of biota.

The similarity of temperatures at which the quantile curves peaked was remarkable, suggesting some underlying phenomenon common to all life occurs in the vicinity of *T*_sup_. Such a phenomenon would be expected to have impact on the temperature sensitivity of many biological systems. We might expect that, as an example for further investigation, there may be an asymmetry in the robustness of ecosystems immediately above and below *T*_sup_.

This paper has generated a number of novel results. We:

1. define a Δ-shaped biokinetic spectrum for temperature;
2. describe and identify the MTG;
3. provide evidence that the MTG represents an actual biological transition between mesophiles and thermophiles;
4. use Bayesian quantile regression to describe the biokinetic spectrum;
5. explore variation of the quantile curve parameters with temperature and within subgroups;
6. replicate the Δ-shape of the biokinetic spectrum using smoothed parameter values for the thermodynamic model.

Having described the existence and nature of the biokinetic spectrum, we expect further work will concentrate on evolutionary implications, the origin of endothermy, application of the spectrum to biological models, elucidation of causative mechanisms, transition phenomena at *T*_sup_, and, of course, the nature and existence of the MTG. These are matters of wide interest, especially to physiologists, microbiologists, system biologists, and astrobiologists.

## Methods

### Data

Some of the data used in the analyses were generated by ourselves or by colleagues. These have been previously reported in an earlier publication [10]. The remaining data were obtained by searching the scholarly literature. We attempted to broaden the search as far as possible rather than limit it to particular taxa. We use the word strain rather than species or taxa to refer to organisms grown under different conditions or by different researchers. The overall aim was to locate the fastest observed rates possible as well as the spread of rates below them. For this reason we included multicellular organisms, such as insects and mites, as well as a single endotherm data set consisting of cultured mammalian cells. We included growth rates for strains grown under multiple conditions but regarded them as separate strains. The overall aim was to locate the fastest observed rates possible as well as the spread of rates below them. We did not exclude any data based on the growth rate or by publication date. Our strategy to locate potentially useful data consisted of searching for combinations of the search strings: “growth rate”, “generation time”, “doubling time”, “intrinsic growth”, “specific growth”, “nov gen”, “nov sp”, “isolate”, and “novel” using Google Scholar. Searches were conducted between 17 June 2014 and 18 September 2015. Some papers were also provided by colleagues. The reference lists of the publications we located were searched for further potential publications. Publications that described measurements of growth rates or generation times of cellular organisms at specific temperatures were retained. Publications that only contained relative rates from which absolute rates were not calculable, only reported growth-no growth status, or dealt with non-cellular organisms such as viruses, were discarded. Most sources identified were peer-reviewed journals, but a small number were textbooks, conference proceedings, technical notes, and academic theses. All retained publications, or relevant chapters, were saved in PDF format. Tabular listings of growth rates or generation times in publications were transcribed manually into text files. Legible figures displaying growth rates or generation times were digitized using the open source program g3data, available from https://github.com/pn2200/g3data. Both tabular and digitised data were saved in the original units used within the publication. An R program was written to process the data, which consisted of converting generation times to rates (*r* = ln(2)/*g*), expressing the data as rate per minute, and generating plots of the data for visual comparison with the original figures. Codes were assigned manually that uniquely identified strains within publications. The data were then collated into a single file for analysis. The sources for the collated data, summarized in S2 Table, included 1627 strains and comprised 10956 records of growth rates (or rates of metabolism in some cases). They covered a temperature range of -10–122°C. The raw data used in the analyses are to be found in S1 Data.

We noted above that the data were not randomly selected, since this was not possible. This means that quantiles calculated herein depended on occurrences of slower-growing strains within the data. Life on Earth contains many very slow-growing species that are largely multicellular. However, only a small proportion of unicellular organisms are likely to have been described [29]. Of greater concern is the number of non-culturable strains, since these may also be slow-growing, but at least their prevalence may be assessable by nucleic acid probes [30]. Their inclusion here would be to shift the quantiles upwards. However, this can be accommodated quite simply by examining higher quantiles, such as the 99% or higher.

The complete set of growth rates was used to calculate quantile curves. To fit the thermodynamic model we restricted the analysis to the 694 strains that had growth curves consisting of at least 5 data points and had a distinct peak.

### Quantile Bayesian model

We used quantile regression to obtain fitted curves below which a specified proportion of the observation fell. We chose to work within a Bayesian framework so that the approach was compatible with a previously developed Bayesian thermodynamic model. As noted in the text we simultaneously fitted an ascending quantile curve and a descending quantile curve. These are defined as:

$$r=\left\{\begin{array}{cc} \exp\left(a+b\times T\right) & T\leq T_{\text{sup}}\\ \exp\left(c+d\times T\right) & T>T_{\text{sup}} \end{array}\right.$$

where *T* is temperature (°C), *r* is the predicted quantile, *T*_sup_ = (*c* − *a*)/(*d* − *b*) and *a*, *b*, *c*, *d* are parameters to be estimated.

After exploratory work we choose the following priors for the parameters: *a* ∼ N(−6.5, 0.01), *b* ∼ N(0.1, 0.01), *c* ∼ N(1.0, 0.01), and *d* ∼ N(−0.1, 0.01). Inference was obtained in the form of posterior means and variances using Markov Chain Monte Carlo (MCMC) simulation [31]. The details of the fitting procedure are given elsewhere [32]. We also found it necessary to restrict the proposals for *a*, *b*, *c*, *d* so as to maintain *T*_sup_ within the range of the observed data and which resulted in *b* > 0 and *d* < 0. The above procedure was separately implemented for various subsets of the data, such as trophic status groups. This was done simultaneously in a single run for all quantiles.

To obtain trends of thermodynamic parameters with the temperature we first fitted the quantile Bayesian model to overlapping subsets of data. The subsets consisted of growth data of strains with *T*_opt_ in the temperature ranges ≤ 30, (1, 31], (2, 32], …, > 75. Then we compared the fitted curves from the thermodynamic model for those strains with *T*_opt_ that fell within each interval to the quantile curves. In each interval, we tabulated the strains whose thermodynamic growth curves rose above the quantile curve within the strain’s observed temperature range (exceedance strains). Since there were only a few growth curves that exceeded the upper quantiles we smoothed the trends to highlight the posterior trends with temperature using cubic-spline smoothing with either df = 5 or df = 10. We averaged the thermodynamic parameters (*c*, $\Delta H_{A}^{‡}$, *n*, Δ*C*_*P*_) of the exceedance strains across the MCMC iterations. Finally, we calculated predicted limits of growth rates using the thermodynamic parameters of the exceedance strains using Eq 2.

### Thermodynamic model

Below, we refer to the observed growth rate as *r* and the modeled growth rate as *F*. The model shown in Eq 2 below assumes that the growth rate is governed by a single, enzyme-catalyzed reaction system that is limiting under all conditions. In the equation the quantity *F* is the predicted rate given the temperature and the values of the parameters. The numerator $\left(T\text{ exp}(c-\Delta H_{A}^{‡}/RT)\right)$ is essentially an Arrhenius model that describes the rate of the putative enzyme-catalyzed rate-controlling reaction as a function of temperature while the denominator models the change in expected rate due to the effects of temperature on the conformation and, hence, catalytic activity of the putative enzyme catalyzing that reaction. The model assumes that hydrophobic interactions are the larger contributers to denaturation [33].

$$F=\frac{T\exp\left(C-\frac{\Delta H_{A}^{‡}}{RT}\right)}{1+\exp\left(-n\frac{\Delta H^{⋆}-T\Delta S^{⋆}+\Delta C_{P}\left(T-T_{H}^{⋆}-T\log\left(\frac{T}{T_{S}^{⋆}}\right)\right)}{RT}\right)}$$ (2)

In Eq 2: *R* is the gas constant (8.314J/K mol); *c* is a scaling constant that also incorporates the Boltzmann and Planck constants; $\Delta H_{A}^{‡}$ is the enthalpy of activation (J/mol); *T* is the temperature in degrees Kelvin; Δ*C*_*P*_ is the heat capacity change (J/K mol-amino acid residue) upon denaturation of the rate-controlling reaction; n is the number of amino acid residues; ΔH* is the enthalpy change (J/mol amino acid residue) at $T_{H}^{*}$ the convergence temperature for enthalpy (K) of protein unfolding; ΔS* is the entropy change (J/K) at $T_{S}^{*}$ the convergence temperature for entropy (K) of protein unfolding.

The thermodynamic model has been described in previous publications [4, 34, 35]. Our starred notation follows that of Murphy et al (1990) [36] except that ΔH*, ΔS*, and Δ*C*_*P*_ are expressed per mole of amino acid residue [4] and we introduce the *n* parameter. The parameters, ΔH* and ΔS*, which are discussed elsewhere with a change in notation (i.e. Δ*H*^*u*^ and Δ*S*^*u*^) [37, 38], arise in the context of enthalpy-entropy compensation [39]. We follow [40] in assuming ΔH* and ΔS* to be constants for hydrocarbons and also follow [39] in assuming $T_{H}^{*}$ and $T_{S}^{*}$ are universal to protein, although they use a different notation.

We allowed four parameters to have values specific to each strain: (*c*, $\Delta H_{A}^{‡}$, *n*, Δ*C*_*P*_). We assumed these strain parameters to be Gaussian distributed with means specific to their thermal group. Strains were grouped into one of 60 alternative thermal groups selected by examination of the percentiles of the observed *T*_opt_. The temperature at which denaturation is minimized, *T*_mes_, is calculated by Eq 3 [4].

$$T_{\text{mes}}=T_{H}^{⋆}-\Delta H^{⋆}/\Delta C_{P}$$ (3)

To control the variance homogeneity we worked on the square root scale. We assumed that the square root of the observed growth rate had a Gaussian distribution with a mean given by the square root of the modeled value, $\sqrt{F}$, and with an unknown precision (reciprocal variance), $\sqrt{r}\sim N(\sqrt{F},\psi )$.

We used a Bayesian approach to allow for uncertainty in measurement and parameters to be incorporated in a natural way through the appropriate prior specification. For *c*, $\Delta H_{A}^{‡}$ and *n* we assigned normal priors to the strain parameters in which the means were specific to the thermal group. In the case of Δ*C*_*P*_ we used a simple prior since this parameter was always well supported by data and did not benefit from ‘borrowing of strength’ between strains. The universal and thermal group group parameters were each assigned uniform priors. Prior specifications are given in Table 6. Finally, the observational precision was assigned a gamma distribution, *ψ* ~ Γ(0.001, 0.001). This was done simultaneously in a single run for all strains. Inference was obtained in the form of posterior means and variances using MCMC. We chose to update the parameters of each strain as a block using Haario updates [41]. We also used Haario updates for each set of thermal group mean parameters and the strain parameter precisions. For the universal parameters we used adaptive direction sampling [42] combined with a low probability stepping-stone proposal [43].

**Table 6:** Priors for thermodynamic model parameters.

*Parameter*	*Priors*
Scaling constant	$\begin{array}{ccc} C_{j} & \sim & \text{N}(c_{d(j)},\tau_{C_{d(j)}})\\ C_{d} & \sim & \text{Unif}(-20,\;150)\\ \tau_{C_{d(j)}} & \sim & \Gamma \left(10^{-3},10^{-3}\right) \end{array}$
Enthalpy of activation	$\begin{array}{ccc} \Delta H_{Aj}^{‡} & \sim & N(\Delta H_{Ad(j)}^{‡},\;\tau_{\Delta H_{A}^{‡}{}_{d(j)}}\times 10^{-8})\\ \Delta H_{Ad}^{‡} & \sim & \text{Unif}(0.01,\;500000)\\ \tau_{\Delta H_{A}^{‡}{}_{d(j)}} & \sim & \Gamma (10^{\text{-3}}{,10}^{\text{-3}}\text{)} \end{array}$
Heat capacity change	Δ*C*_*Pj*_ ~ N(65, 0.0001)
Number of amino acid residues	$\begin{array}{lll} n_{j} & \sim & \text{N}(n_{d(j)},\;\tau_{n_{d(j)}}\times 10^{-6})\\ n_{d} & \sim & \text{Unif}(1,\;2000)\\ \tau_{N_{d(j)}} & \sim & \Gamma \left(10^{-3},10^{-3}\right) \end{array}$
Enthalpy change at convergence temperature	ΔH* ~ Unif(3000, 7000)
Entropy change at convergence temperature	ΔS* ~ Unif(10, 30)
Convergence temperature for enthalpy	$T_{H}^{*}\;\sim \text{ Unif}(320,\;420)$
Convergence temperature for entropy	$T_{S}^{*}\;\sim \text{ Unif}(320,\;420)$

Shown are the prior distributions which are either Gaussian, gamma or uniform distributions. The parameters of the Gaussian distributions are their means and precisions (reciprocal variances). Strain level parameters are subscripted by *j*, thermal group parameters by *d*, and membership of strain *j* in thermal group *d* by *d*(*j*).

## Supporting Information

S1 File Fitted curves for the thermodynamic model. (PDF)

S1 TablePosteriors for the thermodynamic model parameters.The strain posterior parameter estimates are shown first, followed by universal posterior parameter estimates. For the strains are shown the strain code, strain name, *c* (scaling constant), $\Delta H_{A}^{‡}$ (enthalpy of activation, J/mol) Δ*C*_*P*_ (heat capacity change, J/K mol-amino acid-residue), and *n* (number of amino acid residues). At the bottom of the table are shown the universal posterior parameter estimates consisting of ΔH* (enthalpy change, J/mol amino acid residue), ΔS* (entropy change, J/K), $T_{H}^{*}$ (convergence temperature for enthalpy, K), and $T_{S}^{*}$ (convergence temperature for entropy, K). Strains are sorted by *T*_opt_.(PDF)

S2 Table Sources of data. Shown are the strain code, strain name, Aero. (aerobic status: A = aerobe, AN = anerobe, FA = facultative anaerobe, microA = microaerobe, U = unknown), Troph. (trophic status: A = autotroph, H = heterotroph, M = mixotroph, U = unknown), Smp. (size of data set), *T*_min_ (minimum temperature for observed growth, C), *T*_opt_ (temperature of maximal observed growth, C), *T*_max_ (maximum temperature for observed growth, C), and Lit. (literature source).
(PDF)

S1 Data Collated data. The file contains the collated data used in this paper.
(CSV)
