# Correction: The Biokinetic Spectrum for Temperature

**DOI:** 10.1371/journal.pone.0157804

**Published:** 2016-06-13

**Authors:** Ross Corkrey, Tom A. McMeekin, John P. Bowman, David A. Ratkowsky, June Olley, Tom Ross

Fig 1 appears incorrectly in the published article. Please see the correct Fig 1 and its legend here.

**Fig 1:**
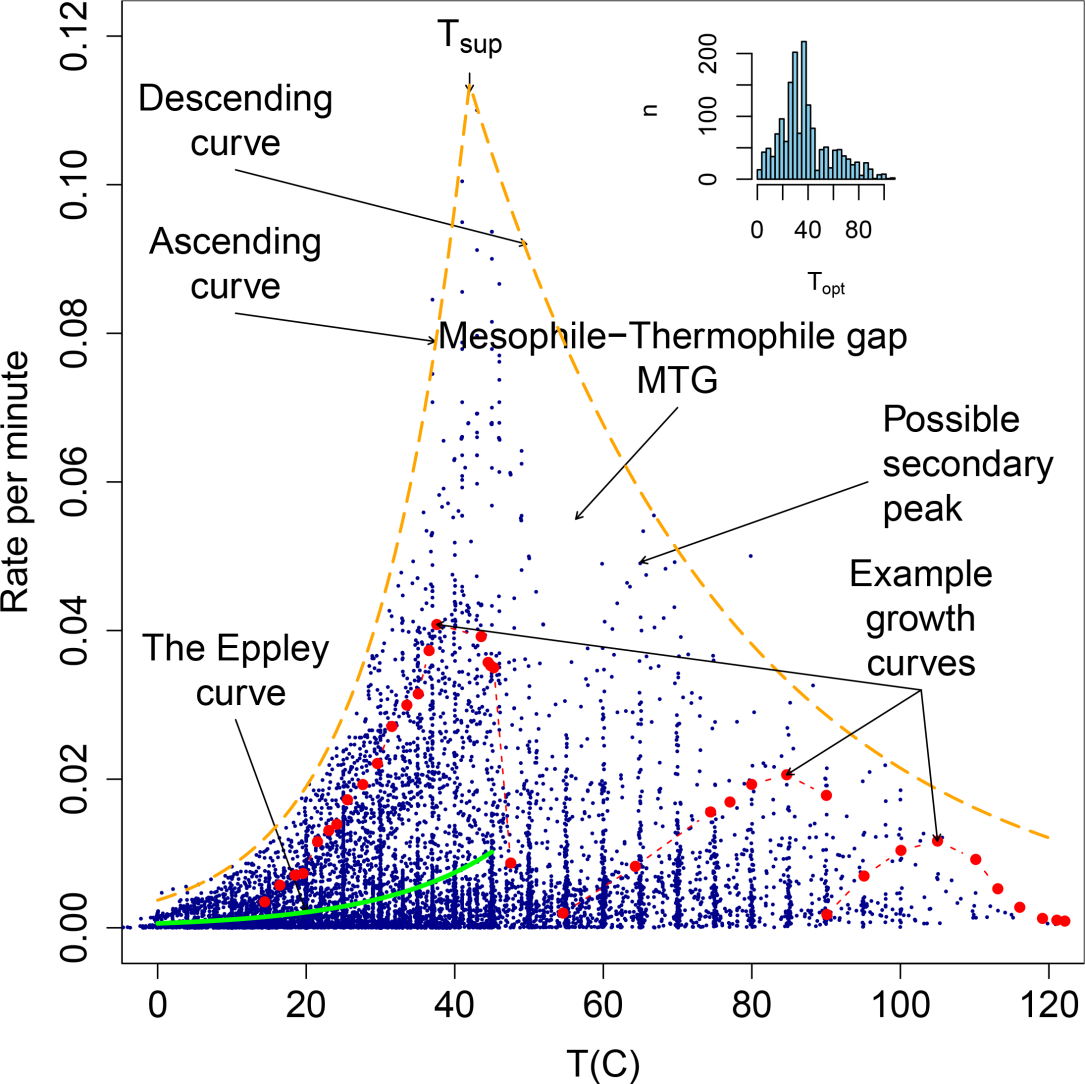
The biokinetic spectrum for temperature. The observed rate of growth for all 1627 strains versus temperature consisting of 10956 data points. We highlight as a visual indication the distribution of the data using dashed lines labeled ascending curve and descending curve. We indicate the location of the Mesophile-Thermophile Gap (MTG) described in the text and of a possible secondary peak. We also show an examples of growth curves for three strains (dashed red), and the curve described by Eppley [8] (solid green) and over the same temperature range he used. The inset shows a histogram of Topt of the strains.
