# Supplementary material for: The Biokinetic Spectrum for Temperature
Source: PLoS One. 2016 Apr 18;11(4):e0153343. doi: 10.1371/journal.pone.0153343 (PMC4835062; doi:10.1371/journal.pone.0153343)
Supplement: S1 File — (PDF) [file pone.0153343.s001.pdf]

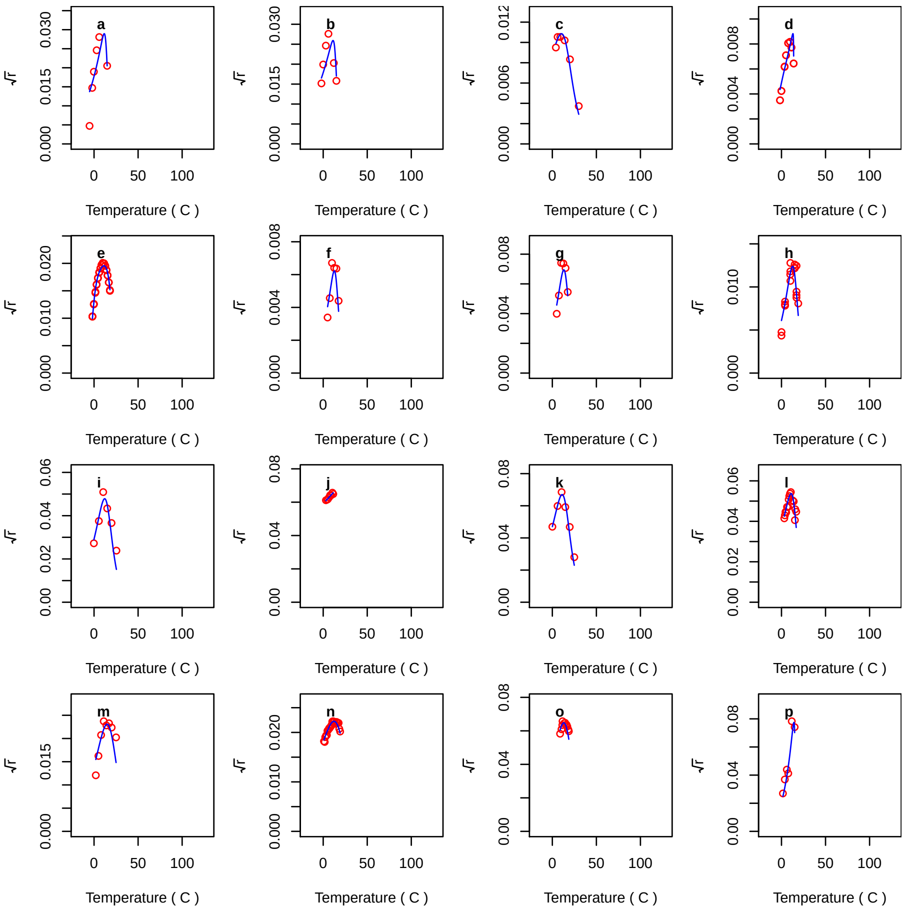

Figure 1. Fitted curve for strains. Fitted curve for strains: (a) *Clostridium sp.* (strain 876) [12], (b) *Clostridium algoriphilium* (strain 877) [13], (c) *Stichococcus sp.* (strain 1444) [5], (d) *Desulfofrigus marinus* (strain 178) [15], (e) *Desulfotalea psychrophila* (strain 180) [15], (f) *Synura sphagnicola* (strain 1466) [21], (g) *Synura sphagnicola* (strain 1467) [21], (h) *Methanogenium frigidum* (strain 859) [24], (i) *Arcobacter sp.* (strain 1359) [11], (j) *Clostridium lacusfryxellense* (strain 1366) [6], (k) *Pseudomonas sp.* (strain 1355) [11], (l) *Colwellia hornerae* (strain 879) [25], (m) *Cryptomonas marssonii* (strain 1127) [27], (n) *Psychroflexus torquis* (strain 1051) [28], (o) *Clostridium bowmanii* (strain 1363) [6], (p) *Brochothrix thermosphacta* (strain 1414) [29]. Shown for each is the mean posterior predicted curve and the observed data using circles. Strain codes in parentheses are listed in Table S2.

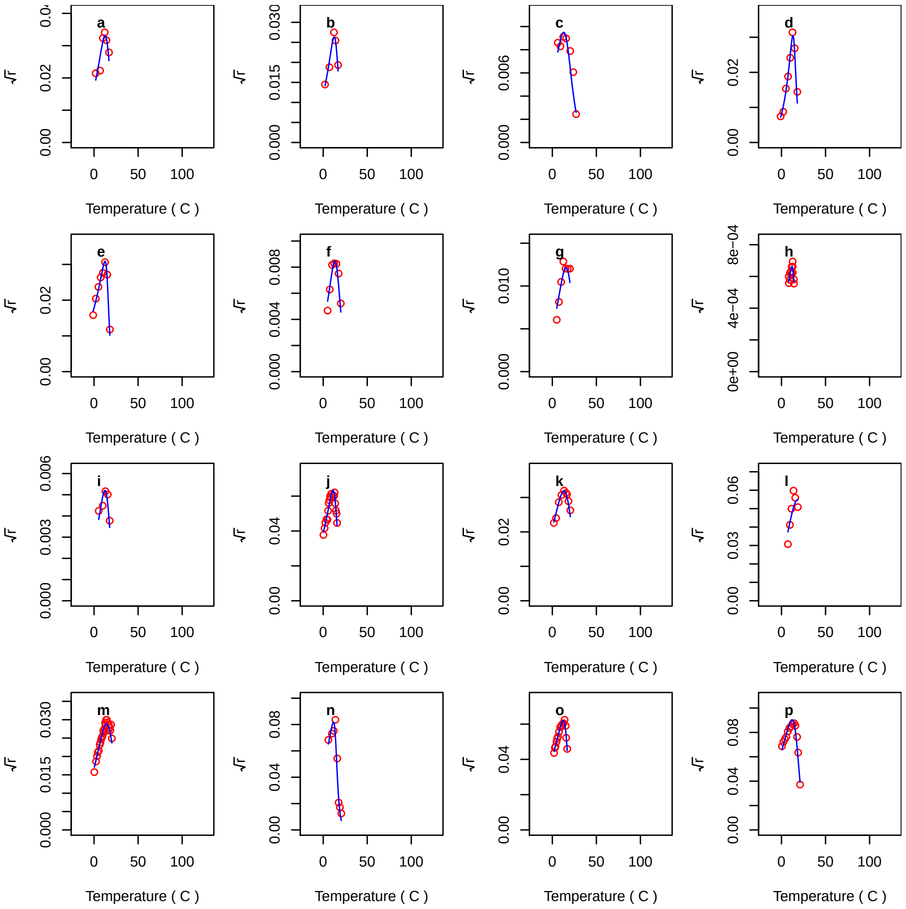

Figure 2. Fitted curve for strains. Fitted curve for strains: (a) *Psychromonas antarcticus* (strain 996) [31], (b) *Clostridium vincentii* (strain 1005) [33], (c) *Chlamydomonas raudensis* (strain 1543) [32], (d) *Chlamydomonas alpina* (strain 1440) [35], (e) *Chlamydomonas subcaudata* (strain 1443) [35], (f) *Synura sphagnicola* (strain 1468) [21], (g) *Synura sphagnicola* (strain 1470) [21], (h) *Candida curiosa* (strain 819) [36], (i) *Flavobacterium segetis* (strain 1342) [37], (j) *Colwellia demingiae* (strain 878) [25], (k) *Arthrobacter glacialis* (strain 1381) [38], (l) *Vibrio marinus* (strain 200) [39], (m) *Glaciecola punicea* (strain 522) [40], (n) *Photobacterium frigidiphilum* (strain 861) [41], (o) *Colwellia psychotropica* (strain 880) [25], (p) *Psychrobacter glacincola* (strain 396) [45]. Shown for each is the mean posterior predicted curve and the observed data using circles. Strain codes in parentheses are listed in Table S2.

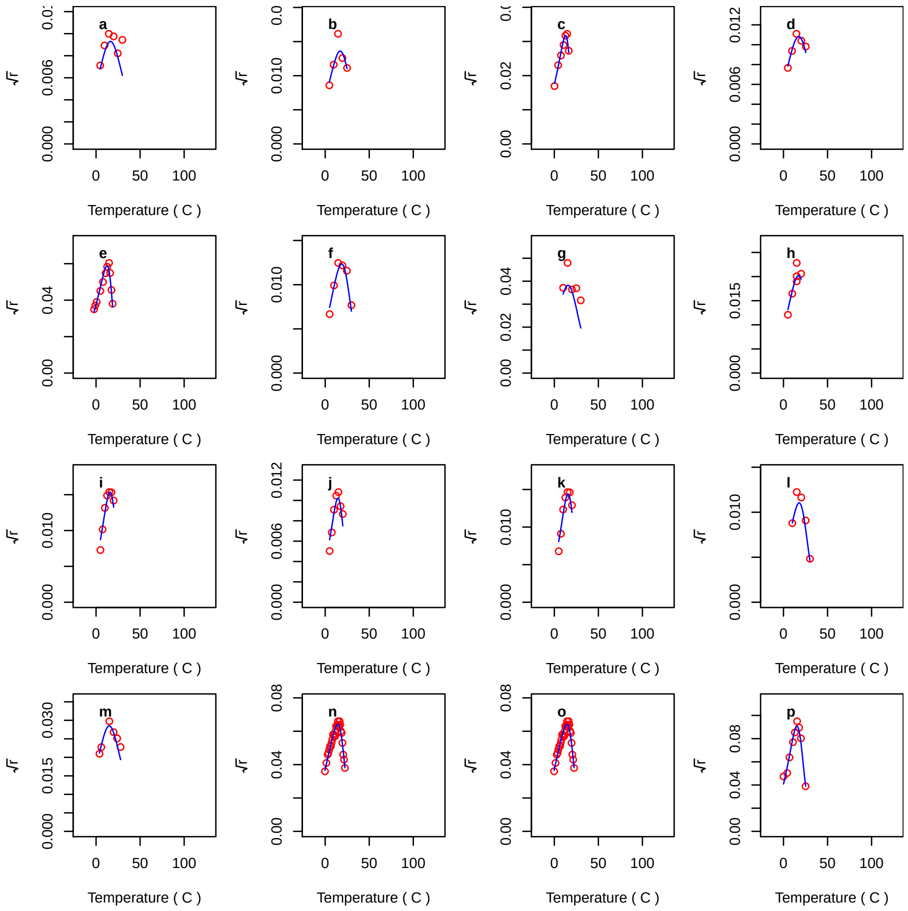

Figure 3. Fitted curve for strains. Fitted curve for strains: (a) *Cyanobacterial str.* (strain 92) [42], (b) *Cyanobacterial str.* (strain 97) [42], (c) *Arthrobacter glacialis* (strain 1380) [38], (d) *Cyanobacterial str.* (strain 82) [42], (e) *Pseudomonas sp.* (strain 502) [46], (f) *Cyanobacterial str.* (strain 83) [42], (g) *Mucor racemosus* (strain 184) [47], (h) *Gonyaulax tamarensis* (strain 1194) [50], (i) *Synura sphagnicola* (strain 1464) [21], (j) *Synura sphagnicola* (strain 1469) [21], (k) *Synura sphagnicola* (strain 1471) [21], (l) *Cyanobacterial str.* (strain 85) [42], (m) *Clostridium tagluense* (strain 265) [53], (n) *Shewanella gelidimarina* (strain 523) [55], (o) *Shewanella gelidimarina* (strain 525) [55], (p) *Shewanella sp.* (strain 1357) [11]. Shown for each is the mean posterior predicted curve and the observed data using circles. Strain codes in parentheses are listed in Table S2.

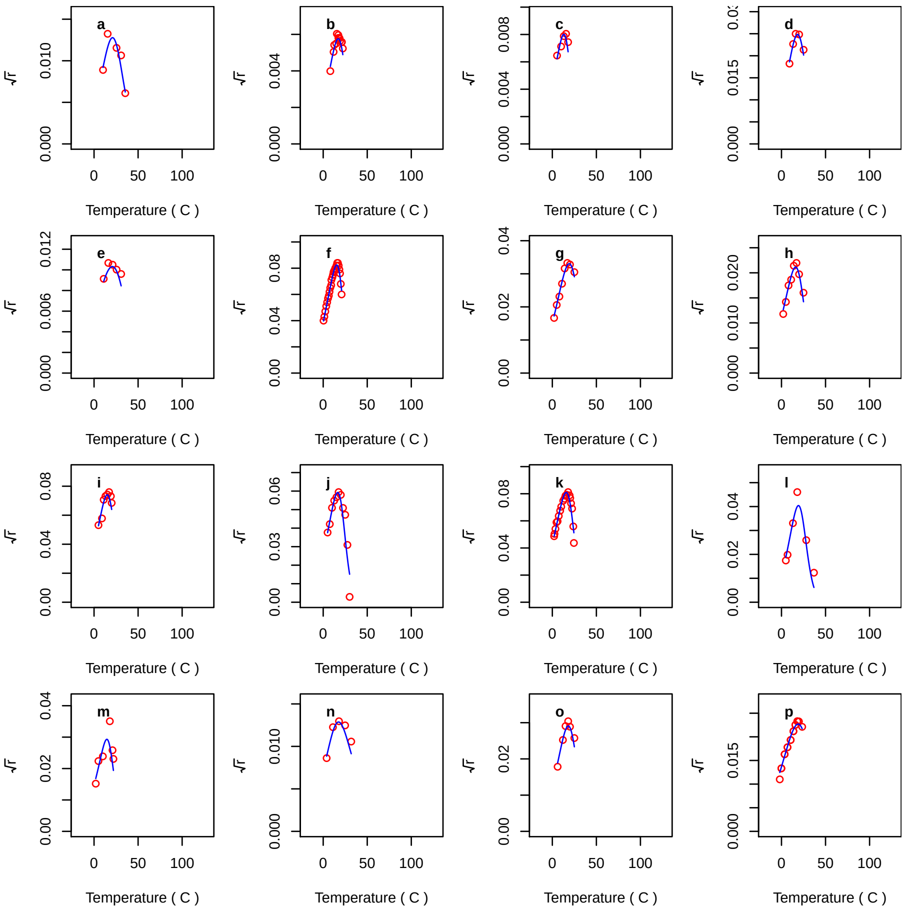

Figure 4. Fitted curve for strains. Fitted curve for strains: (a) *Cyanobacterial str.* (strain 94) [42], (b) *Salmo gairdneri* (strain 1368) [56], (c) *Flavobacterium weaverense* (strain 1343) [37], (d) *Rhodomonas salina* (strain 1378) [44], (e) *Cyanobacterial str.* (strain 86) [42], (f) *Shewanella gelidimarina* (strain 524) [55], (g) *Asterionella formosa* (strain 1124) [27], (h) *Dinobryon divergens* (strain 1128) [27], (i) *Shewanella donghaensis* (strain 1029) [61], (j) *Pseudoalteromonas antarctica* (strain 370) [62], (k) *Colwellia psychrerythraea* (strain 881) [25], (l) *Psychrobacter muriicola* (strain 1362) [63], (m) *Rhodoglobus vestalii* (strain 1052) [64], (n) *Chlorella sp.* (strain 1452) [26], (o) *Hantzschia amphyoxis* (strain 1456) [65], (p) *Desulfofrigus fragile* (strain 177) [15]. Shown for each is the mean posterior predicted curve and the observed data using circles. Strain codes in parentheses are listed in Table S2.

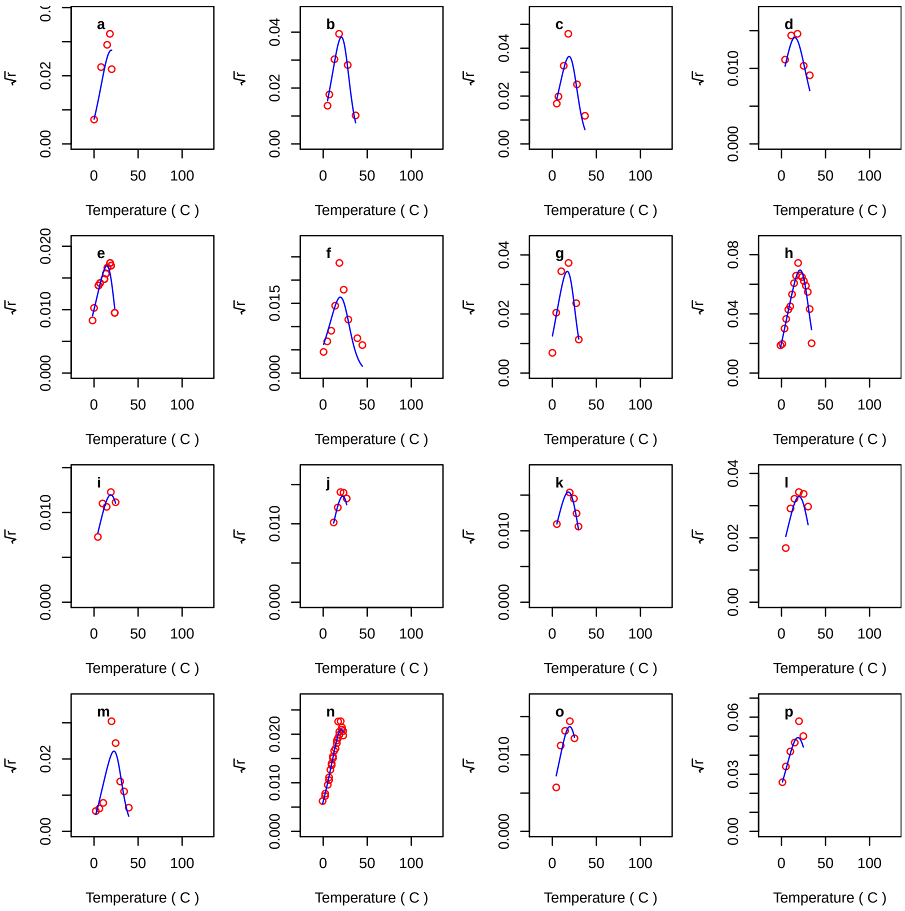

Figure 5. Fitted curve for strains. Fitted curve for strains: (a) *Methanolobus psychrophilus* (strain 1001) [66], (b) *Psychrobacter sp.* (strain 874) [12], (c) *Psychrobacter sp.* (strain 875) [12], (d) *Chlamydomonas sp.* (strain 1450) [26], (e) *Desulfotalea arctica* (strain 179) [15], (f) *Aeromonas hydrophila* (strain 1418) [67], (g) *Thiobacillus sp.* (strain 1352) [68], (h) *Bacterial str.* (strain 1361) [69], (i) *Cyanobacterial str.* (strain 88) [42], (j) *Acyrthosiphon pisum* (strain 425) [70], (k) *Aphis gossypii* (strain 599) [71], (l) *Thalassiosira rotula* (strain 1207) [54], (m) *Aeromonas hydrophila* (strain 1420) [67], (n) *Desulfobacter curvatus* (strain 586) [73], (o) *Cyanobacterial str.* (strain 80) [42], (p) *Acetobacterium paludosum* (strain 994) [75]. Shown for each is the mean posterior predicted curve and the observed data using circles. Strain codes in parentheses are listed in Table S2.

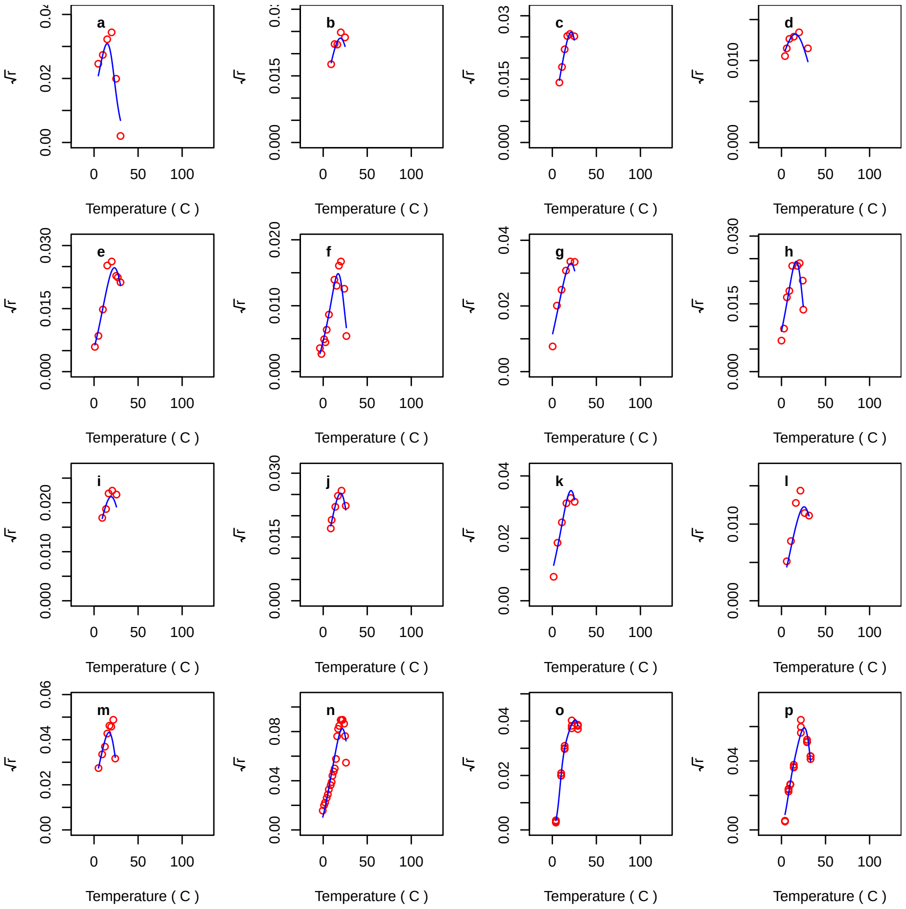

Figure 6. Fitted curve for strains. Fitted curve for strains: (a) *Phaeodactylum tricornutum* (strain 1134) [76], (b) *Skeletonema costatum* (strain 1374) [44], (c) *Tychonema bourrellyi* (strain 1126) [27], (d) *Chlorella sp.* (strain 1447) [5], (e) *Acetobacterium tundrae* (strain 1026) [89], (f) *Desulfobacter psychrotolerans* (strain 1032) [90], (g) *Thalassiosira rotula* (strain 1200) [54], (h) *Rhodoferax antarcticus* (strain 858) [92], (i) *Ditylum brightwellii* (strain 1372) [44], (j) *Phaeodactylum tricornutum* (strain 1373) [44], (k) *Thalassiosira rotula* (strain 1206) [54], (l) *Cyanobacterial str.* (strain 89) [42], (m) *Flavobacterium antarcticum* (strain 1614) [93], (n) *Clostridium gasigenes* (strain 1053) [95], (o) *Candida stellata* (strain 556) [97], (p) *Hanseniaspora uvarum* (strain 557) [97]. Shown for each is the mean posterior predicted curve and the observed data using circles. Strain codes in parentheses are listed in Table S2.

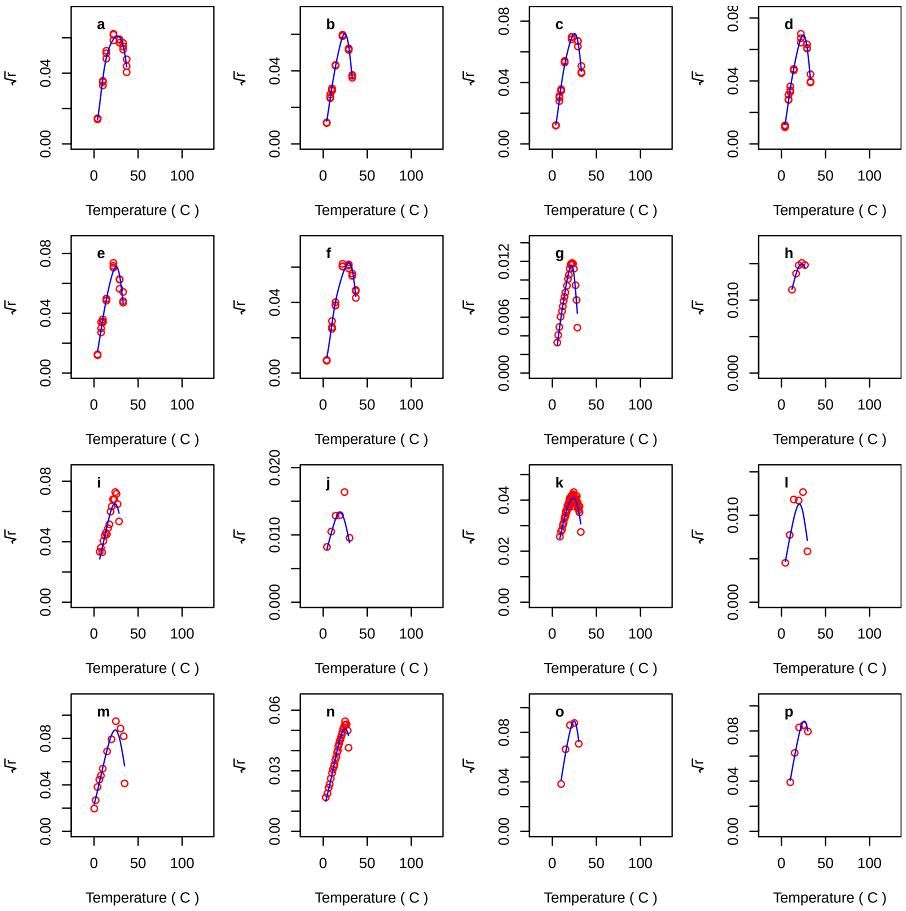

Figure 7. Fitted curve for strains. Fitted curve for strains: (a) *Pichia fermentans* (strain 559) [97], (b) *Saccharomyces kudriavzevii* (strain 574) [97], (c) *Saccharomyces kudriavzevii* (strain 575) [97], (d) *Saccharomyces kudriavzevii* (strain 576) [97], (e) *Saccharomyces kudriavzevii* (strain 577) [97], (f) *Torulaspora delbrueckii* (strain 582) [97], (g) *Methanococcoides burtonii* (strain 501) [100], (h) *Acyrthosiphon pisum* (strain 424) [70], (i) *Cellulomonas sp.* (strain 172) [110], (j) *Cyanobacterial str.* (strain 84) [42], (k) *Gelidibacter sp.* (strain 521) [40], (l) *Cyanobacterial str.* (strain 100) [42], (m) *Bacillus circulans* (strain 1385) [113], (n) *Flavobacterium hibernum* (strain 1095) [115], (o) *Mucor racemosus* (strain 181) [47], (p) *Mucor racemosus* (strain 182) [47]. Shown for each is the mean posterior predicted curve and the observed data using circles. Strain codes in parentheses are listed in Table S2.

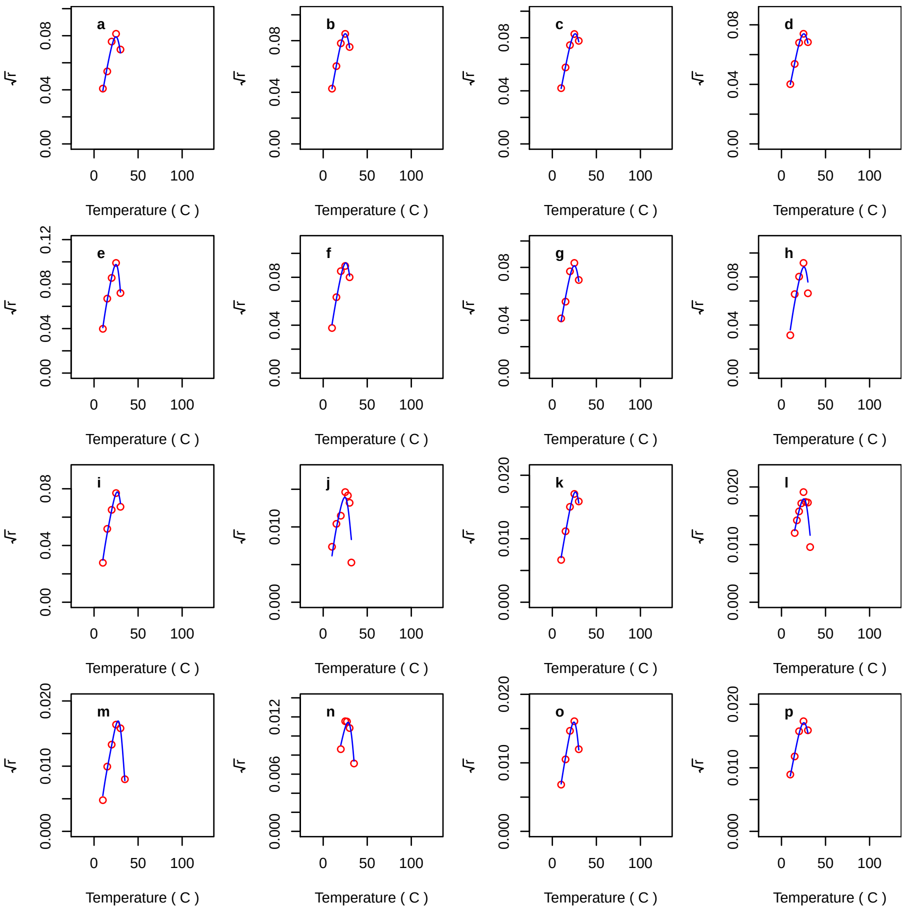

Figure 8. Fitted curve for strains. Fitted curve for strains: (a) *Mucor racemosus* (strain 183) [47], (b) *Mucor racemosus* (strain 185) [47], (c) *Mucor racemosus* (strain 186) [47], (d) *Mucor racemosus* (strain 187) [47], (e) *Mucor racemosus* (strain 189) [47], (f) *Mucor racemosus* (strain 190) [47], (g) *Mucor racemosus* (strain 191) [47], (h) *Mucor racemosus* (strain 193) [47], (i) *Mucor racemosus* (strain 195) [47], (j) *Aphis spiraecola* (strain 415) [124], (k) *Aphis gossypii* (strain 439) [126], (l) *Aphis gossypii* (strain 446) [128], (m) *Aphis gossypii* (strain 602) [135], (n) *Bemisia argentifolii* (strain 608) [136], (o) *Hyadaphis pseudobrassicae* (strain 610) [80], (p) *Rhopalosiphum rufiabdominalis* (strain 665) [140]. Shown for each is the mean posterior predicted curve and the observed data using circles. Strain codes in parentheses are listed in Table S2.

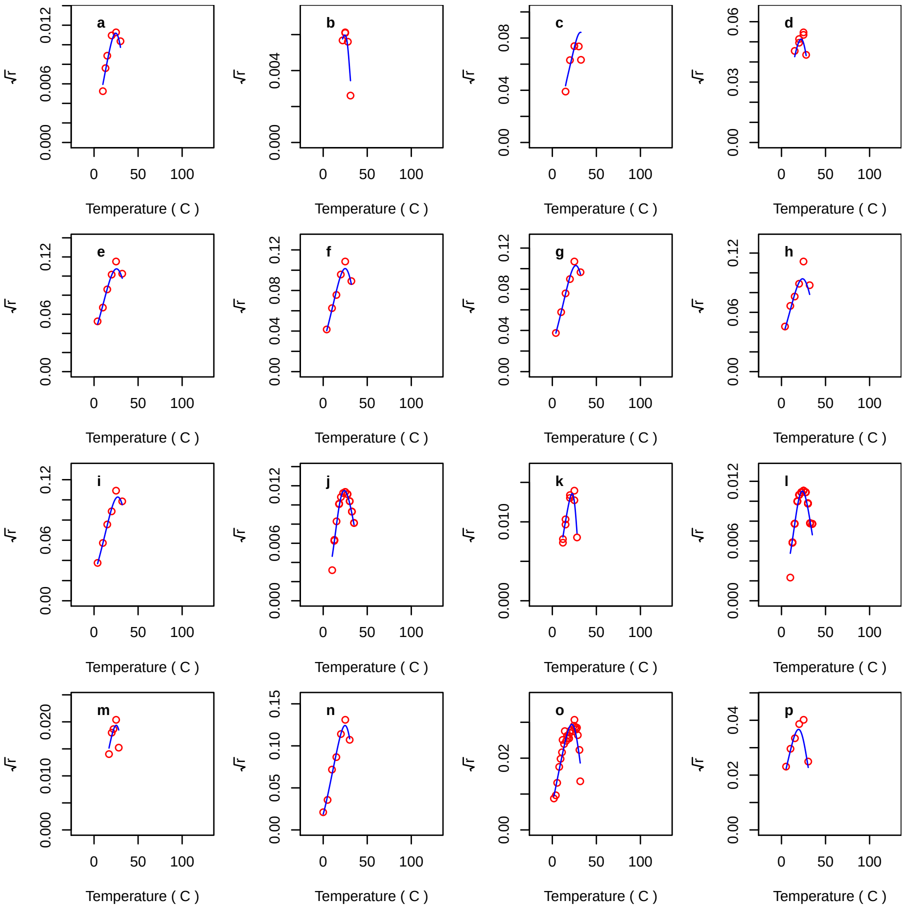

Figure 9. Fitted curve for strains. Fitted curve for strains: (a) *Eriosoma lanigerum* (strain 669) [141], (b) *Diatraea lineolata* (strain 670) [142], (c) *Trichogrammatoidea bactrae* (strain 679) [144], (d) *Methanococcoides alaskense* (strain 1392) [87], (e) *Pseudomonas fluorescens* (strain 1457) [151], (f) *Pseudomonas fluorescens* (strain 1459) [151], (g) *Pseudomonas fluorescens* (strain 1460) [151], (h) *Pseudomonas fluorescens* (strain 1461) [151], (i) *Pseudomonas fluorescens* (strain 1462) [151], (j) *Acarus siro* (strain 161) [153], (k) *Sitobion miscanthi* (strain 660) [77], (l) *Aleuroglyphus ovatus* (strain 162) [153], (m) *Paronychiurus kimi* (strain 459) [157], (n) *Brochothrix thermosphacta* (strain 924) [116], (o) *Bacillus sp.* (strain 1399) [158], (p) *Thalassiosira rotula* (strain 1203) [54]. Shown for each is the mean posterior predicted curve and the observed data using circles. Strain codes in parentheses are listed in Table S2.

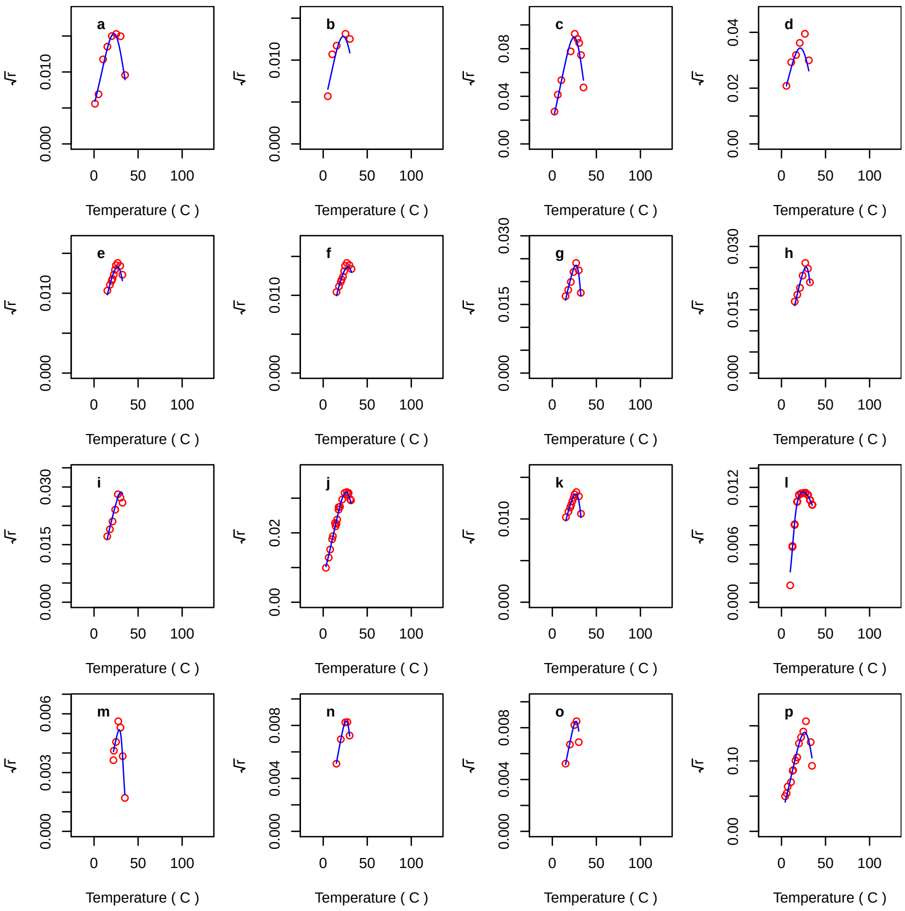

Figure 10. Fitted curve for strains. Fitted curve for strains: (a) *Methanosarcina lacustris* (strain 997) [159], (b) *Cyanobacterial str.* (strain 81) [42], (c) *Pseudomonas sp.* (strain 1384) [38], (d) *Thalassiosira rotula* (strain 1205) [54], (e) *Sitophilus oryzae* (strain 644) [102], (f) *Sitophilus oryzae* (strain 645) [102], (g) *Sitophilus oryzae* (strain 676) [171], (h) *Sitophilus oryzae* (strain 677) [171], (i) *Sitophilus oryzae* (strain 678) [171], (j) *Gram-negative str.* (strain 1400) [158], (k) *Sitophilus oryzae* (strain 643) [102], (l) *Tyrophagus putrescentiae* (strain 163) [153], (m) *Liposcelis badia* (strain 461) [175], (n) *Macrolophus pygmaeus* (strain 627) [176], (o) *Macrolophus pygmaeus* (strain 628) [176], (p) *Pseudomonas sp.* (strain 148) [177]. Shown for each is the mean posterior predicted curve and the observed data using circles. Strain codes in parentheses are listed in Table S2.

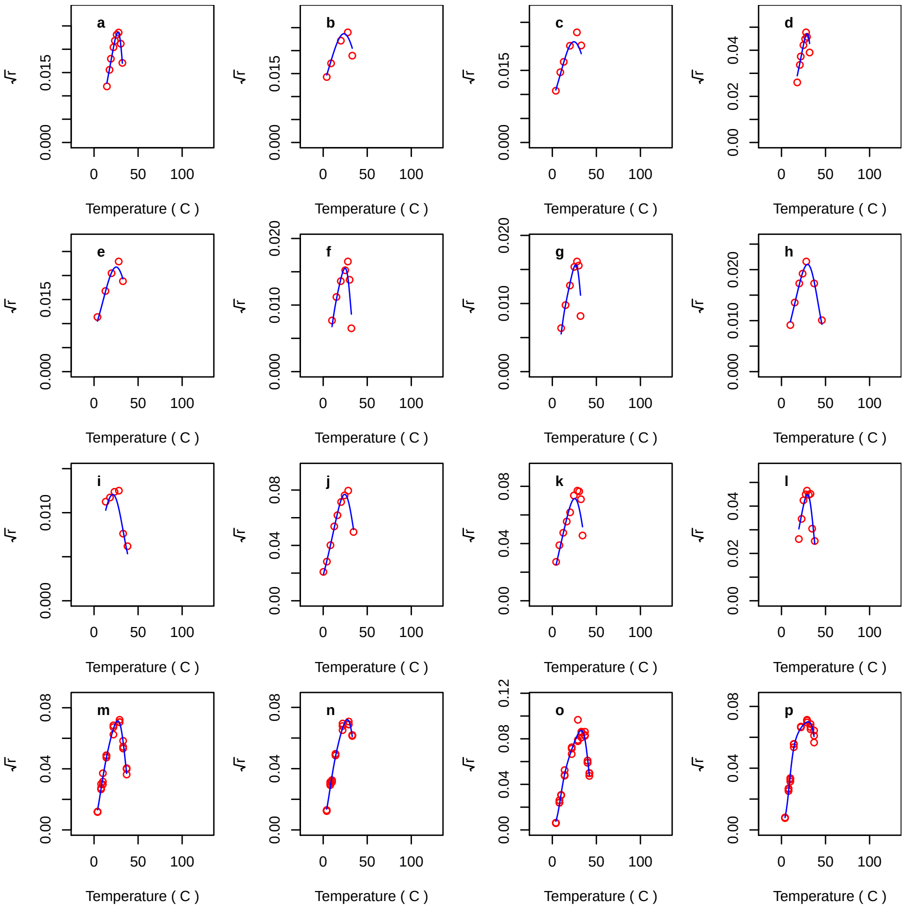

Figure 11. Fitted curve for strains. Fitted curve for strains: (a) *Synechococcus sp.* (strain 210) [104], (b) *Desulfovibrio litoralis* (strain 64) [178], (c) *Desulfovibrio cuneatus* (strain 62) [178], (d) *Xylella fastidiosa* (strain 150) [179], (e) *Desulfovibrio cuneatus* (strain 63) [178], (f) *Toxoptera aurantii* (strain 442) [186], (g) *Toxoptera citricida* (strain 458) [188], (h) *Methanobacterium Bryantii* (strain 1266) [190], (i) *Chlamydomonas sp.* (strain 1451) [26], (j) *Arthrobacter sp.* (strain 1382) [38], (k) *Arthrobacter sp.* (strain 1383) [38], (l) *Acidithiobacillus ferridurans* (strain 315) [192], (m) *Saccharomyces bayanus* (strain 561) [97], (n) *Saccharomyces bayanus* (strain 562) [97], (o) *Saccharomyces cerevisiae* (strain 571) [97], (p) *Saccharomyces mikatae* (strain 578) [97]. Shown for each is the mean posterior predicted curve and the observed data using circles. Strain codes in parentheses are listed in Table S2.

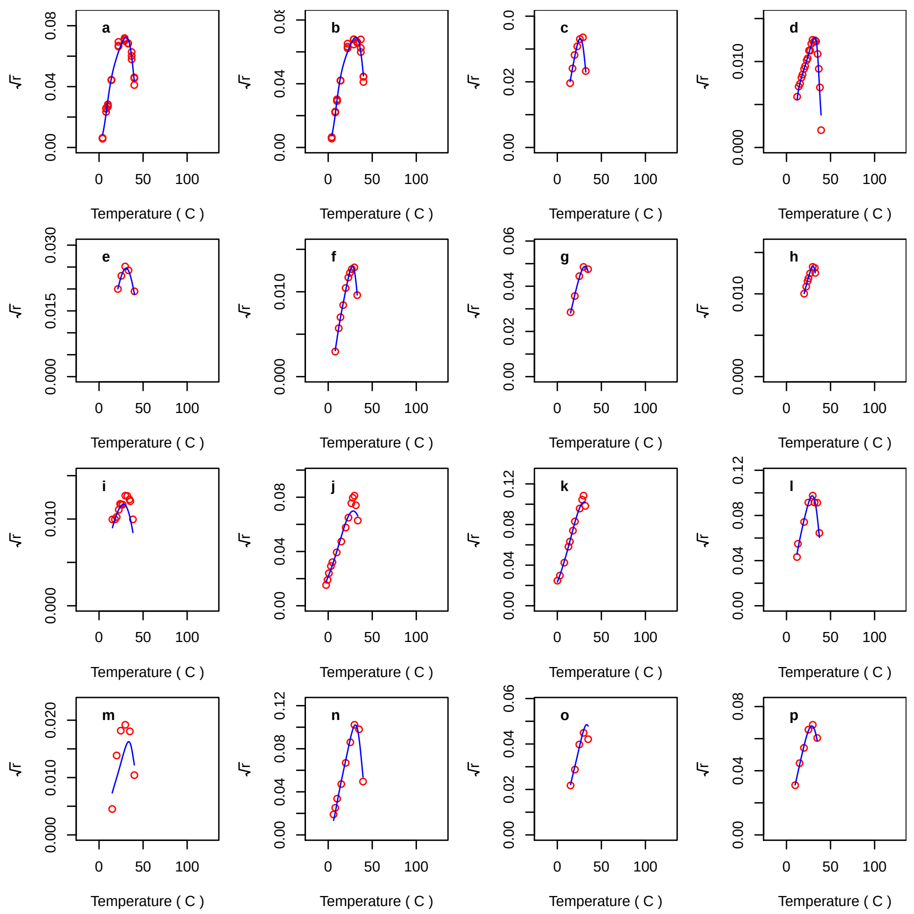

Figure 12. Fitted curve for strains. Fitted curve for strains: (a) *Saccharomyces paradoxus* (strain 580) [97], (b) *Saccharomyces paradoxus* (strain 581) [97], (c) *Nannochloropsis oceanica* (strain 1130) [200], (d) *Acidithiobacillus thiooxidans* (strain 500) [100], (e) *Methanocorpusculum sinsese* (strain 1272) [202], (f) *Plutella xylostella* (strain 410) [203], (g) *Chimaereicella alkaliphila* (strain 1339) [204], (h) *Oryzaephilus surinamensis* (strain 634) [102], (i) *Rhyzopertha dominica* (strain 638) [102], (j) *Spirillum sp.* (strain 503) [46], (k) *Pseudomonas fluorescens* (strain 1417) [205], (l) *Aspergillus candidus* (strain 817) [206], (m) *Acaryochloris marina* (strain 1249) [207], (n) *Bacillus coagulans* (strain 1386) [113], (o) *Chimaereicella alkaliphila* (strain 1340) [204], (p) *Xanthomonas campestris* (strain 149) [179]. Shown for each is the mean posterior predicted curve and the observed data using circles. Strain codes in parentheses are listed in Table S2.

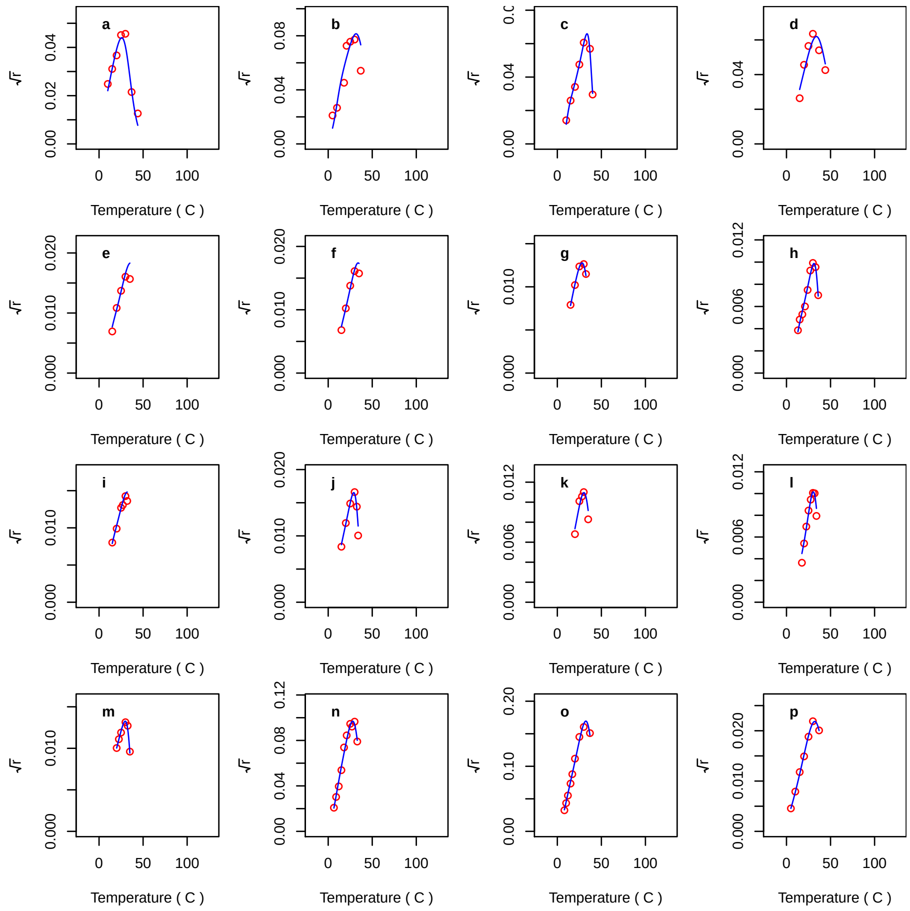

Figure 13. Fitted curve for strains. Fitted curve for strains: (a) *Haliea salexigens* (strain 411) [209], (b) *Methanospirillum stamsii* (strain 1341) [210], (c) *Balneola vulgaris* (strain 687) [211], (d) *Haliea rubra* (strain 409) [212], (e) *Tetranychus evansi* (strain 341) [222], (f) *Tetranychus evansi* (strain 343) [222], (g) *Iphiseius degenerans* (strain 416) [226], (h) *Elasmopalpus lignosellus* (strain 417) [227], (i) *Euseius finlandicus* (strain 449) [230], (j) *Tyrophagus putrescentiae* (strain 450) [231], (k) *Bemisia argentifolii* (strain 467) [235], (l) *Dactylopius austrinus* (strain 626) [241], (m) *Oryzaephilus surinamensis* (strain 631) [242], (n) *Erwinia amylovora* (strain 761) [246], (o) *Bacillus cereus* (strain 901) [248], (p) *Pseudomonas sp.* (strain 1479) [10]. Shown for each is the mean posterior predicted curve and the observed data using circles. Strain codes in parentheses are listed in Table S2.

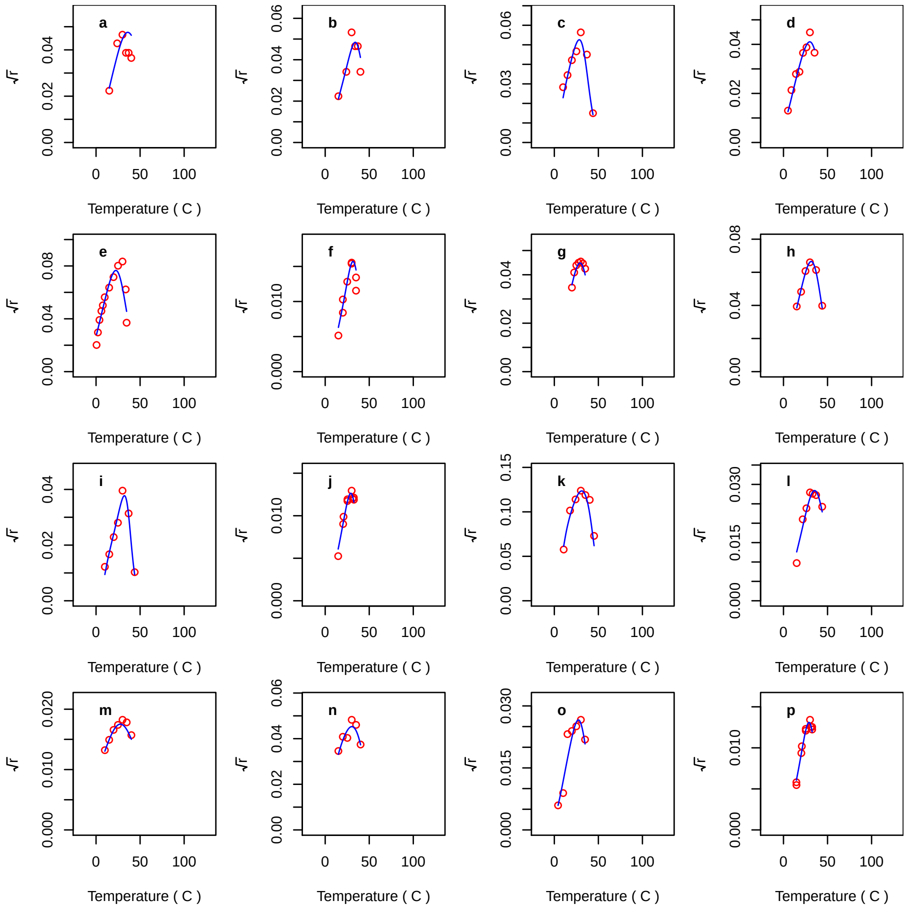

Figure 14. Fitted curve for strains. Fitted curve for strains: (a) *Alkalibacterium iburiense* (strain 1599) [250], (b) *Alkalibacterium iburiense* (strain 1600) [250], (c) *Thalassobaculum salexigens* (strain 785) [256], (d) *Trichococcus patagoniensis* (strain 1036) [257], (e) *Bacillus coagulans* (strain 1387) [113], (f) *Urolepis rufipes* (strain 432) [258], (g) *Acidithiobacillus ferrivorans* (strain 381) [259], (h) *Nisaea denitrificans* (strain 1102) [261], (i) *Melitea salexigens* (strain 768) [263], (j) *Muscidifurax raptor* (strain 430) [264], (k) *Marinobacter alkaliphilus* (strain 77) [265], (l) *Spirochaeta africana* (strain 1033) [266], (m) *Chlorella vulgaris* (strain 1415) [267], (n) *Microcella putealis* (strain 1335) [268], (o) *Sulfurimonas paralvinellae* (strain 954) [269], (p) *Muscidifurax raptor* (strain 431) [264]. Shown for each is the mean posterior predicted curve and the observed data using circles. Strain codes in parentheses are listed in Table S2.

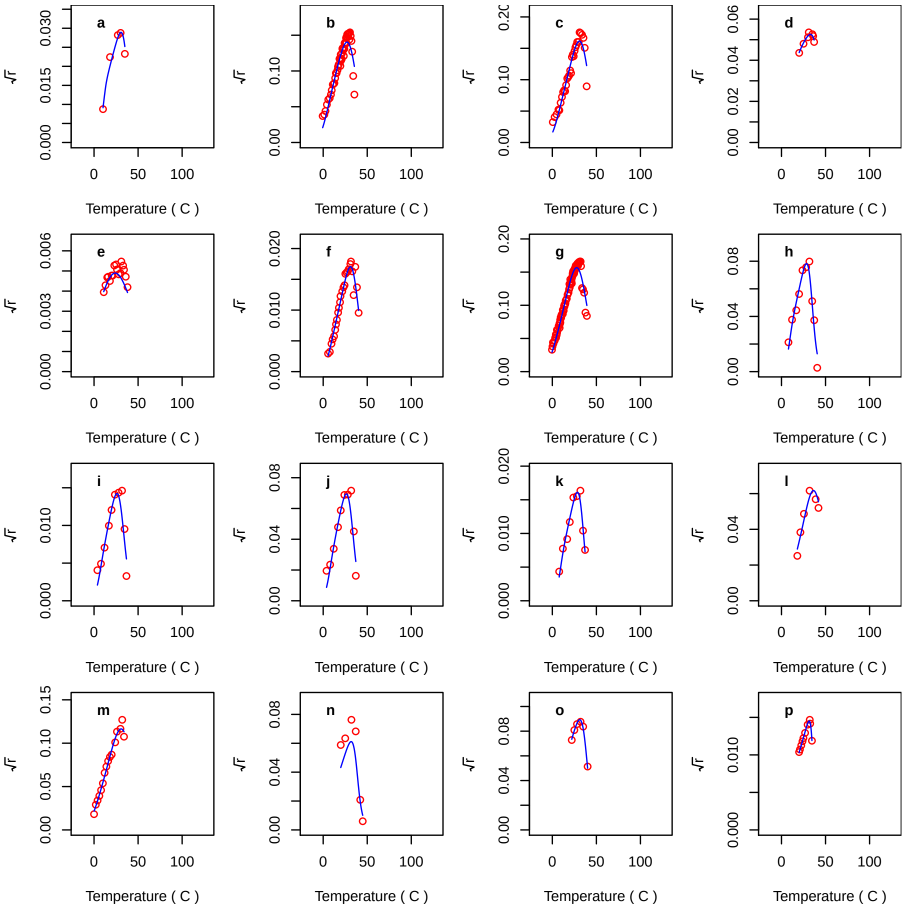

Figure 15. Fitted curve for strains. Fitted curve for strains: (a) *Thiobacillus thioparus* (strain 1353) [68], (b) *Pseudomonas fluorescens* (strain 519) [270], (c) *Aeromonas hydrophila* (strain 504) [271], (d) *Methanosarcina semesiae* (strain 1297) [273], (e) *Acidithiobacillus ferrooxidans* (strain 499) [100], (f) *Halorubrum lacusprofundi* (strain 516) [278], (g) *Pseudomonas putida* (strain 520) [270], (h) *Kluyveromyces battae* (strain 69) [279], (i) *Candida sphaerica* (strain 65) [279], (j) *Candia sphaerica* (strain 68) [279], (k) *Kluyveromyces blattae* (strain 66) [279], (l) *Chlorella pyrenoidosa* (strain 617) [162], (m) *Pseudomonas fluorescens* (strain 143) [182], (n) *Thioreductor micantisoli* (strain 1096) [281], (o) *Saccharomyces rouxii* (strain 263) [180], (p) *Oryzaephilus surinamensis* (strain 636) [102]. Shown for each is the mean posterior predicted curve and the observed data using circles. Strain codes in parentheses are listed in Table S2.

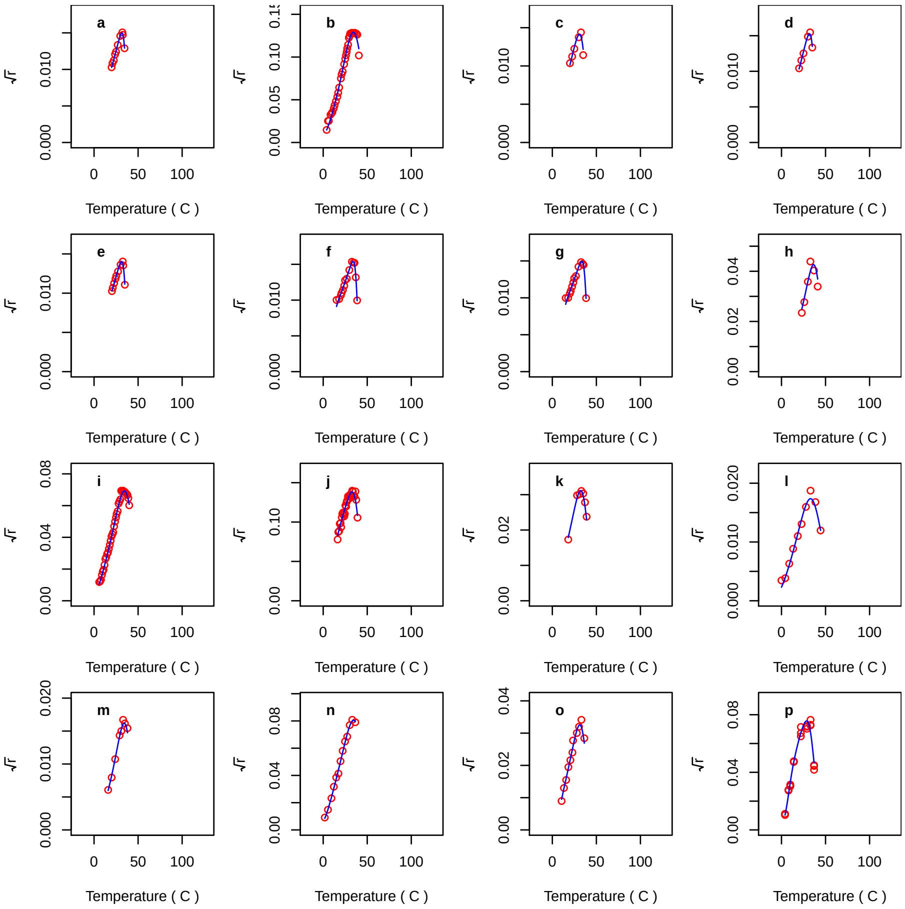

Figure 16. Fitted curve for strains. Fitted curve for strains: (a) *Oryzaephilus surinamensis* (strain 637) [102], (b) *Clostridiurn frigidicarnis* (strain 434) [282], (c) *Oryzaephilus surinamensis* (strain 632) [242], (d) *Oryzaephilus surinamensis* (strain 633) [242], (e) *Oryzaephilus surinamensis* (strain 635) [102], (f) *Rhyzopertha dominica* (strain 641) [102], (g) *Rhyzopertha dominica* (strain 640) [102], (h) *Thiobacillus prosperus* (strain 1274) [288], (i) *Lactococcus paracasei* (strain 32) [289], (j) *Acinetobacter calcoaceticus* (strain 1286) [290], (k) *Methanosarcina semesiae* (strain 1296) [273], (l) *Aeromonas shigelloides* (strain 1423) [67], (m) *Amblyseius womersleyi* (strain 419) [291], (n) *Paracoccus halodenitrificans* (strain 508) [292], (o) *Paracoccus halodenitrificans* (strain 510) [292], (p) *Saccharomyces arboricolus* (strain 560) [97]. Shown for each is the mean posterior predicted curve and the observed data using circles. Strain codes in parentheses are listed in Table S2.

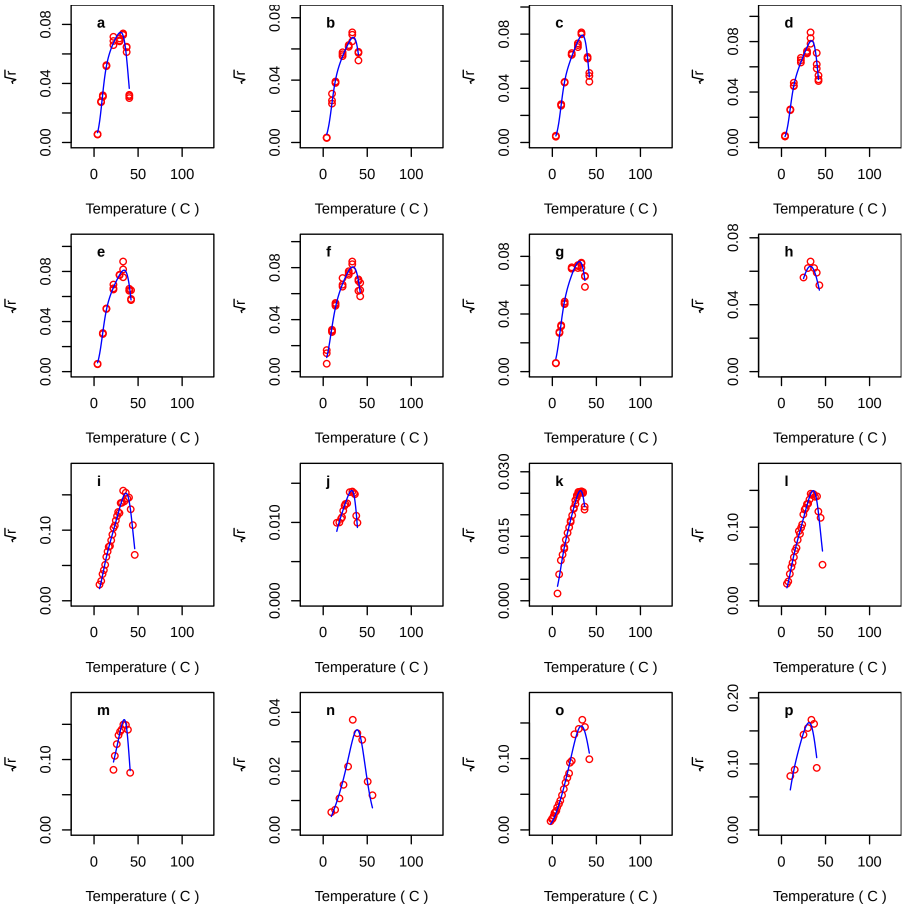

Figure 17. Fitted curve for strains. Fitted curve for strains: (a) *Saccharomyces cariocanus* (strain 563) [97], (b) *Saccharomyces cerevisiae* (strain 566) [97], (c) *Saccharomyces cerevisiae* (strain 567) [97], (d) *Saccharomyces cerevisiae* (strain 569) [97], (e) *Saccharomyces cerevisiae* (strain 570) [97], (f) *Saccharomyces cerevisiae* (strain 573) [97], (g) *Saccharomyces paradoxus* (strain 579) [97], (h) *Saccharomyces uvarum* (strain 818) [293], (i) *Listeria monocytogenes* (strain 528) [295], (j) *Rhyzopertha dominica* (strain 639) [102], (k) *Desulforhopalus species* (strain 587) [73], (l) *Listeria monocytogenes* (strain 527) [295], (m) *Serratia marcescens* (strain 199) [39], (n) *Aeromonas shigelloides* (strain 1422) [67], (o) *Listeria monocytogenes* (strain 1360) [299], (p) *Alkalibacterium psychrotolerans* (strain 1603) [300]. Shown for each is the mean posterior predicted curve and the observed data using circles. Strain codes in parentheses are listed in Table S2.

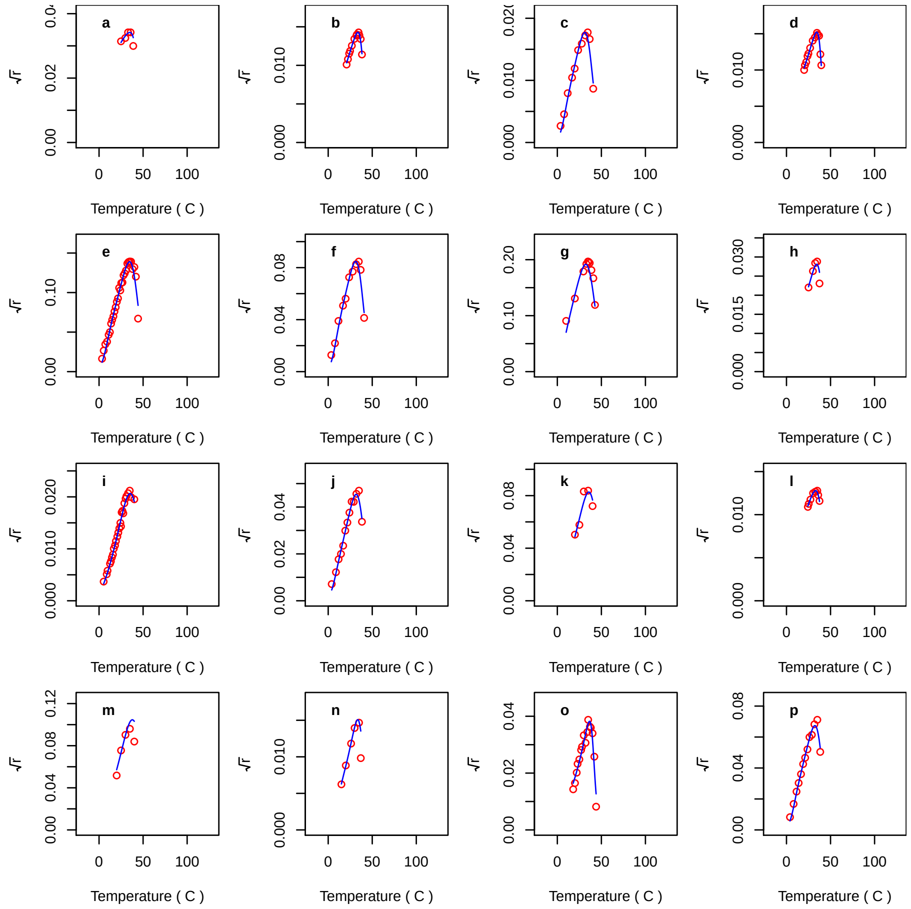

Figure 18. Fitted curve for strains. Fitted curve for strains: (a) *Sporomusa acidovorans* (strain 1542) [302], (b) *Tribolium castaneum* (strain 647) [102], (c) *Kluyveromyces thermotolerans* (strain 67) [279], (d) *Tribolium castaneum* (strain 648) [102], (e) *Listeria monocytogenes* (strain 530) [295], (f) *Kluyveromyces thermotolerans* (strain 70) [279], (g) *Escherichia coli* (strain 1138) [304], (h) *Anabaena variabilis* (strain 1316) [283], (i) *Halorubrum lacusprofundi* (strain 517) [278], (j) *Paracoccus halodenitrificans* (strain 509) [292], (k) *Salinisphaera hydrothermalis* (strain 413) [306], (l) *Tribolium castaneum* (strain 646) [102], (m) *Monascus ruber* (strain 225) [216], (n) *Scolothrips longicornis* (strain 418) [307], (o) *Leptospirillum ferrooxidans* (strain 497) [201], (p) *Paracoccus halodenitrificans* (strain 511) [292]. Shown for each is the mean posterior predicted curve and the observed data using circles. Strain codes in parentheses are listed in Table S2.

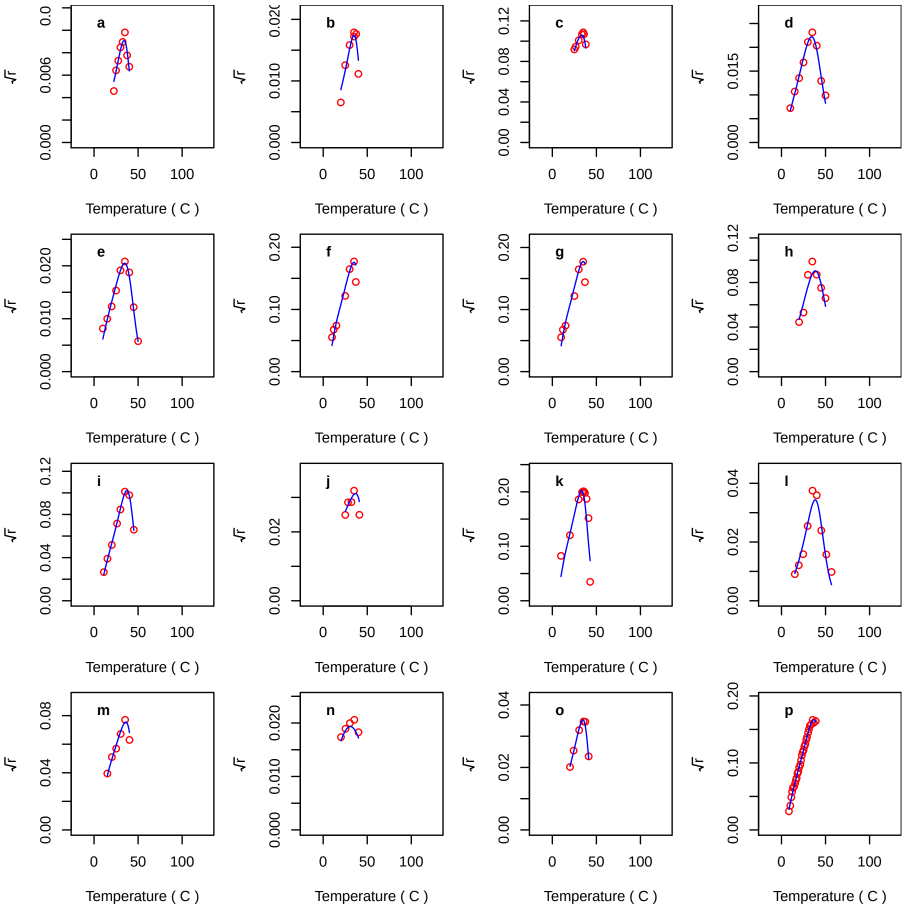

Figure 19. Fitted curve for strains. Fitted curve for strains: (a) *Cryptolestes ferrugineus* (strain 624) [240], (b) *Callosobruchus maculatus* (strain 668) [310], (c) *Saccharomyces cerevisiae* (strain 773) [247], (d) *Methanobacterium flexile* (strain 848) [313], (e) *Methanobacterium movens* (strain 849) [313], (f) *Bacillus cereus* (strain 902) [248], (g) *Bacillus cereus* (strain 903) [248], (h) *Galenea microaerophila* (strain 1554) [316], (i) *Thiobacillus hydrothermalis* (strain 998) [319], (j) *Anoxynatronum sibiricum* (strain 1003) [320], (k) *Salmonella enterica* (strain 1140) [304], (l) *Aeromonas hydrophila* (strain 1419) [67], (m) *Microcella putealis* (strain 1337) [268], (n) *Methanobacterium espanolae* (strain 1288) [323], (o) *Methanohalophilus oregonense* (strain 1290) [324], (p) *Listeria monocytogenes* (strain 545) [325]. Shown for each is the mean posterior predicted curve and the observed data using circles. Strain codes in parentheses are listed in Table S2.

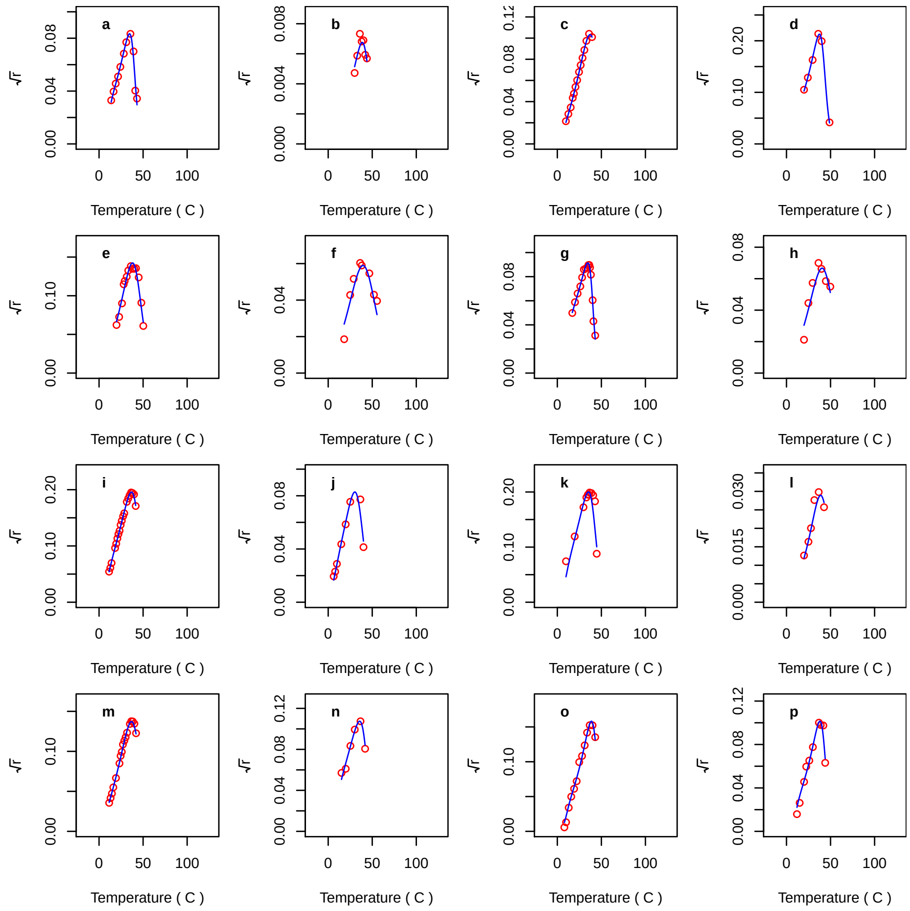

Figure 20. Fitted curve for strains. Fitted curve for strains: (a) *Staphylococcus xylosus* (strain 283) [326], (b) *Synechocystis sp.* (strain 1488) [327], (c) *Halomonas elongata* (strain 514) [292], (d) *Vibrio alginolyticus* (strain 852) [330], (e) *Bacillus subtilis* (strain 205) [39], (f) *Amphibacillus fermentum* (strain 999) [331], (g) *Candida valida* (strain 774) [332], (h) *Haloanaerobium alcaliphilum* (strain 1404) [333], (i) *Staphylococcus xylosus* (strain 287) [326], (j) *Bacillus laterosporus* (strain 1388) [113], (k) *Escherichia coli* (strain 1137) [304], (l) *Methanofollis aquaemaris* (strain 72) [334], (m) *Staphylococcus xylosus* (strain 285) [326], (n) *Clostridium sp.* (strain 856) [335], (o) *Escherichia coli* (strain 783) [336], (p) *Escherichia coli* (strain 407) [338]. Shown for each is the mean posterior predicted curve and the observed data using circles. Strain codes in parentheses are listed in Table S2.

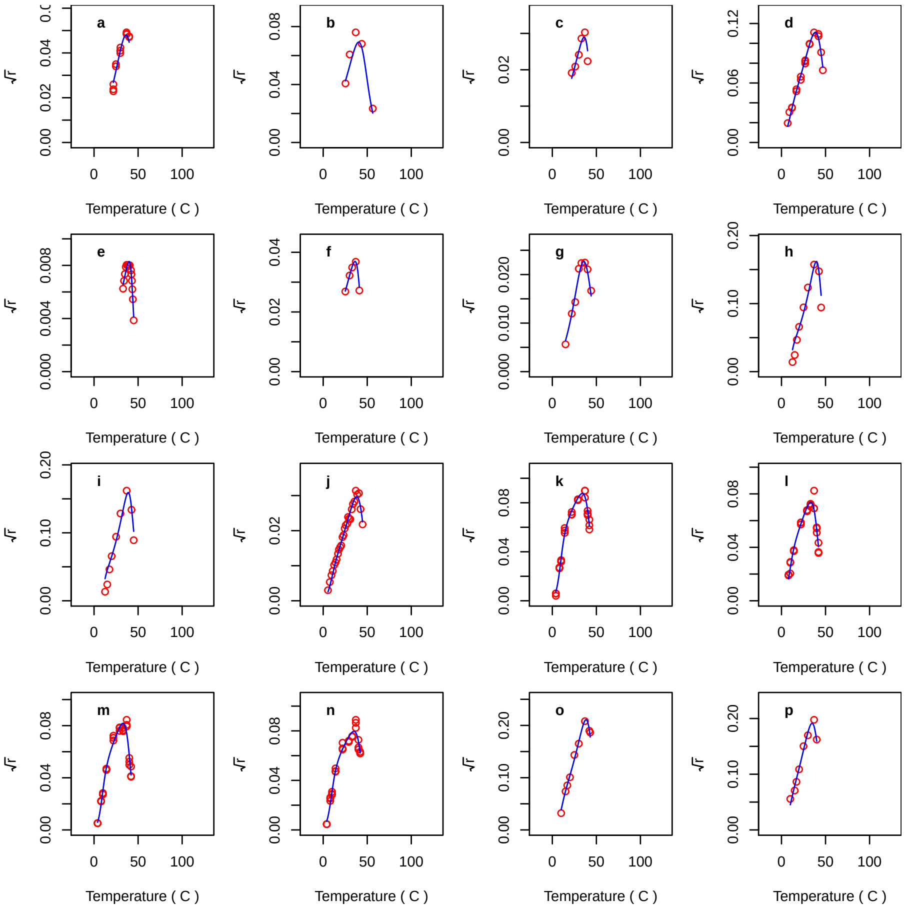

Figure 21. Fitted curve for strains. Fitted curve for strains: (a) *Methanolobus bombayensis* (strain 990) [339], (b) *Methanococcus deltae* (strain 137) [342], (c) *Spirochaeta asiatica* (strain 1035) [266], (d) *Kluyveromyces marxianus* (strain 801) [344], (e) *Synechocystis sp.* (strain 1493) [327], (f) *Thiobacillus prosperus* (strain 1273) [288], (g) *Spirochaeta alkalica* (strain 1034) [266], (h) *Clostridium botulinum* (strain 174) [357], (i) *Clostridium botulinum* (strain 175) [357], (j) *Klebsiella oxytoca* (strain 518) [368], (k) *Saccharomyces cerevisiae* (strain 564) [97], (l) *Saccharomyces cerevisiae* (strain 565) [97], (m) *Saccharomyces cerevisiae* (strain 568) [97], (n) *Saccharomyces cerevisiae* (strain 572) [97], (o) *Bacillus cereus* (strain 898) [248], (p) *Bacillus cereus* (strain 904) [248]. Shown for each is the mean posterior predicted curve and the observed data using circles. Strain codes in parentheses are listed in Table S2.

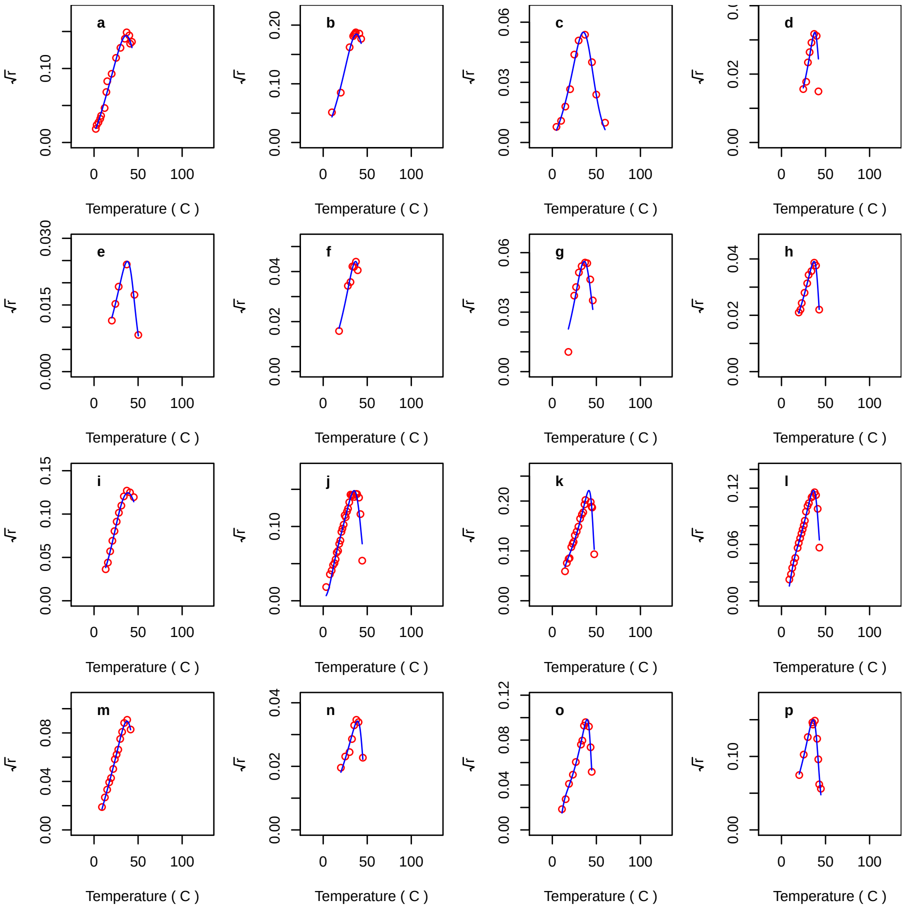

Figure 22. Fitted curve for strains. Fitted curve for strains: (a) *Listeria monocytogenes* (strain 908) [248], (b) *Salmonella enterica* (strain 1139) [304], (c) *Haloanaerobium praevalens* (strain 221) [390], (d) *Methanocalculus taiwanensis* (strain 1413) [387], (e) *Methanobacterium veterum* (strain 1267) [190], (f) *Methanosarcina semesiae* (strain 1298) [273], (g) *Methanococcus maripaludis* (strain 1333) [392], (h) *Staphylococcus xylosus* (strain 282) [326], (i) *Escherichia coli* (strain 507) [292], (j) *Listeria monocytogenes* (strain 529) [295], (k) *Escherichia coli* (strain 6) [395], (l) *Staphylococcus xylosus* (strain 284) [326], (m) *Halomonas elongata* (strain 515) [292], (n) *Clostridium termitidis* (strain 58) [396], (o) *Bacillus halodenitrificans* (strain 1295) [397], (p) *Klebsiella pneumoniae* (strain 946) [398]. Shown for each is the mean posterior predicted curve and the observed data using circles. Strain codes in parentheses are listed in Table S2.

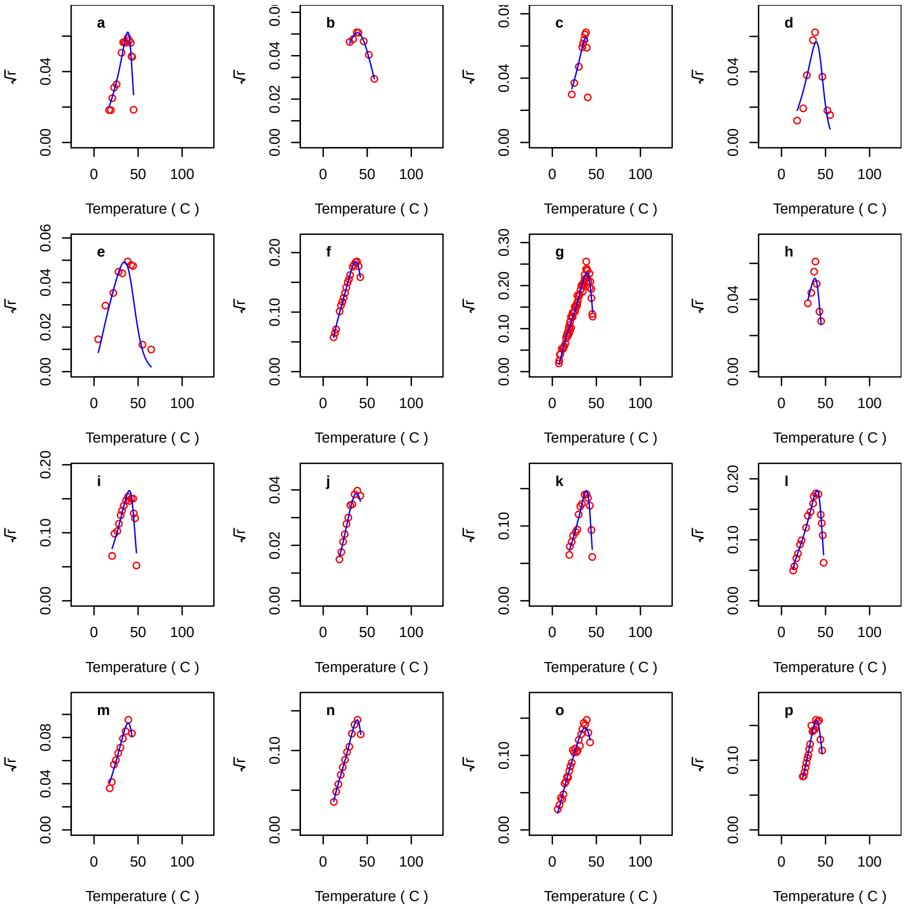

Figure 23. Fitted curve for strains. Fitted curve for strains: (a) *Desulfitobacterium dehalogenans* (strain 25) [399], (b) *Alkalithermophilic Bacteria* (strain 364) [400], (c) *Citrobacter intermedius* (strain 151) [401], (d) *Amphibacillus tropicus* (strain 1000) [331], (e) *Bacillus beveridgei* (strain 1246) [402], (f) *Staphylococcus xylosus* (strain 286) [326], (g) *Escherichia coli* (strain 547) [403], (h) *Sulfobacillus benefaciens* (strain 748) [406], (i) *Escherichia coli* (strain 208) [104], (j) *Halomonas elongata* (strain 513) [292], (k) *Pseudomonas fluorescens* (strain 198) [39], (l) *Escherichia coli* (strain 944) [407], (m) *Escherichia coli* (strain 505) [292], (n) *Escherichia coli* (strain 506) [292], (o) *Listeria monocytogenes* (strain 526) [295], (p) *Streptococcus thermophilus* (strain 485) [412]. Shown for each is the mean posterior predicted curve and the observed data using circles. Strain codes in parentheses are listed in Table S2.

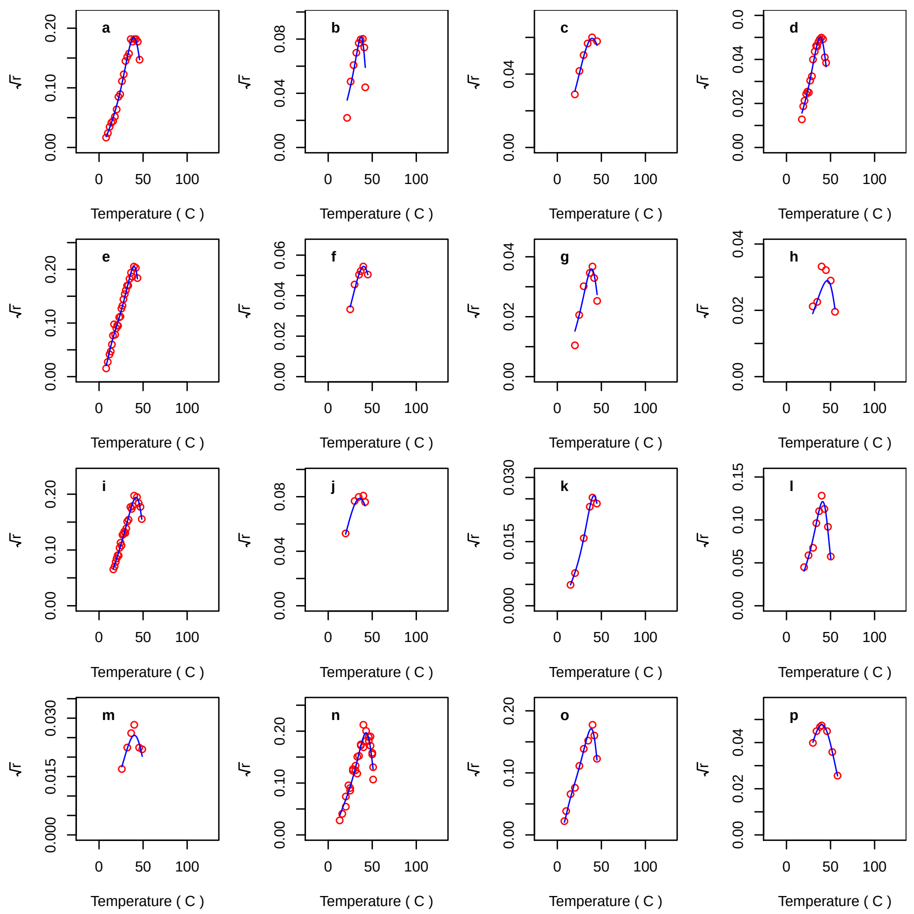

Figure 24. Fitted curve for strains. Fitted curve for strains: (a) *Escherichia coli* (strain 141) [182], (b) *Chlorella pyrenoidosa* (strain 616) [162], (c) *Desulfovibrio capillatus* (strain 1391) [413], (d) *Leptospirillum ferriphilum* (strain 496) [201], (e) *Escherichia coli* (strain 550) [403], (f) *Methanoculleus marisnigri* (strain 1072) [415], (g) *Methanosarcina mazel* (strain 1064) [272], (h) *Nautilia profundicola* (strain 718) [417], (i) *Streptococcus thermophilus* (strain 480) [412], (j) *Magnetospirillum bellicus* (strain 1327) [419], (k) *Methanobacterium kanagiense* (strain 1069) [420], (l) *Alkaliphilus transvaalensis* (strain 1031) [421], (m) *Methanoculleus palmolei* (strain 76) [422], (n) *Clostridium perfringens* (strain 45) [423], (o) *Pseudomonas aeruginosa* (strain 142) [182], (p) *Alkalithermophilic Bacteria* (strain 365) [400]. Shown for each is the mean posterior predicted curve and the observed data using circles. Strain codes in parentheses are listed in Table S2.

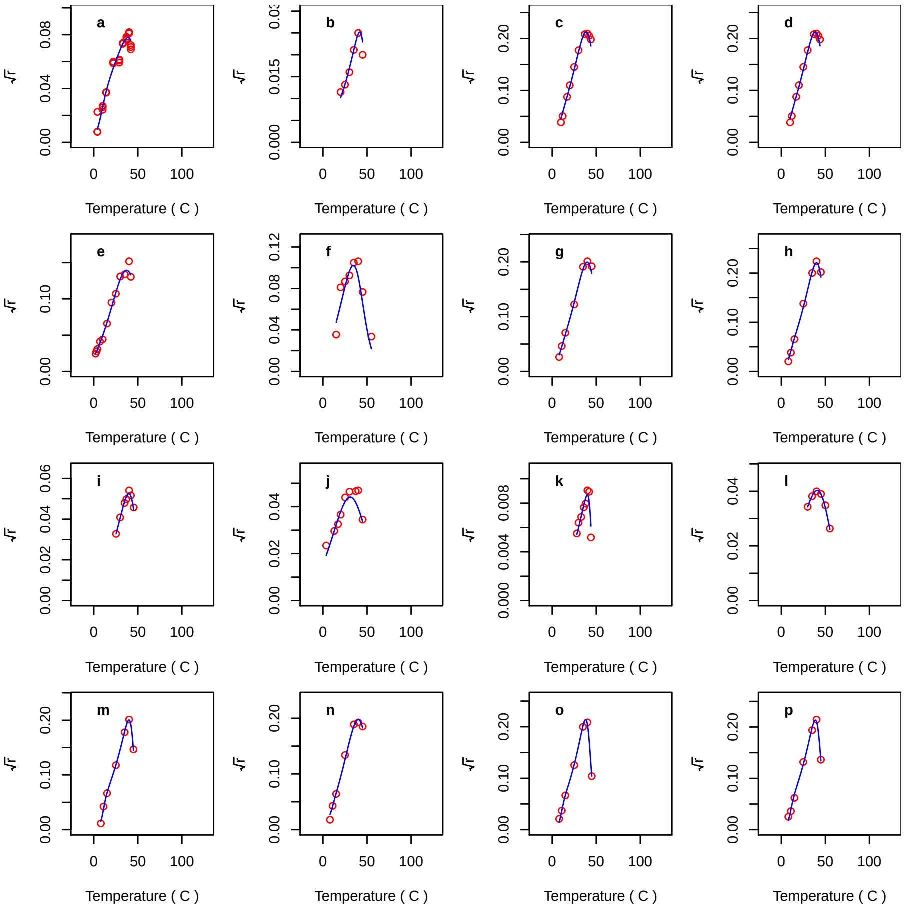

Figure 25. Fitted curve for strains. Fitted curve for strains: (a) *Kluyveromyces marxianus* (strain 558) [97], (b) *Methanobacterium ferruginis* (strain 701) [311], (c) *Bacillus cereus* (strain 899) [248], (d) *Bacillus cereus* (strain 900) [248], (e) *Listeria monocytogenes* (strain 907) [248], (f) *Thiomicrospira thermophila* (strain 1268) [428], (g) *Escherichia coli* (strain 909) [248], (h) *Escherichia coli* (strain 915) [248], (i) *Haloanaerobium lacusroseus* (strain 1405) [429], (j) *Methanobacterium subterraneum* (strain 766) [430], (k) *Synechocystis sp.* (strain 1487) [327], (l) *Alkalithermophilic Bacteria* (strain 369) [400], (m) *Escherichia coli* (strain 910) [248], (n) *Escherichia coli* (strain 911) [248], (o) *Escherichia coli* (strain 912) [248], (p) *Escherichia coli* (strain 913) [248]. Shown for each is the mean posterior predicted curve and the observed data using circles. Strain codes in parentheses are listed in Table S2.

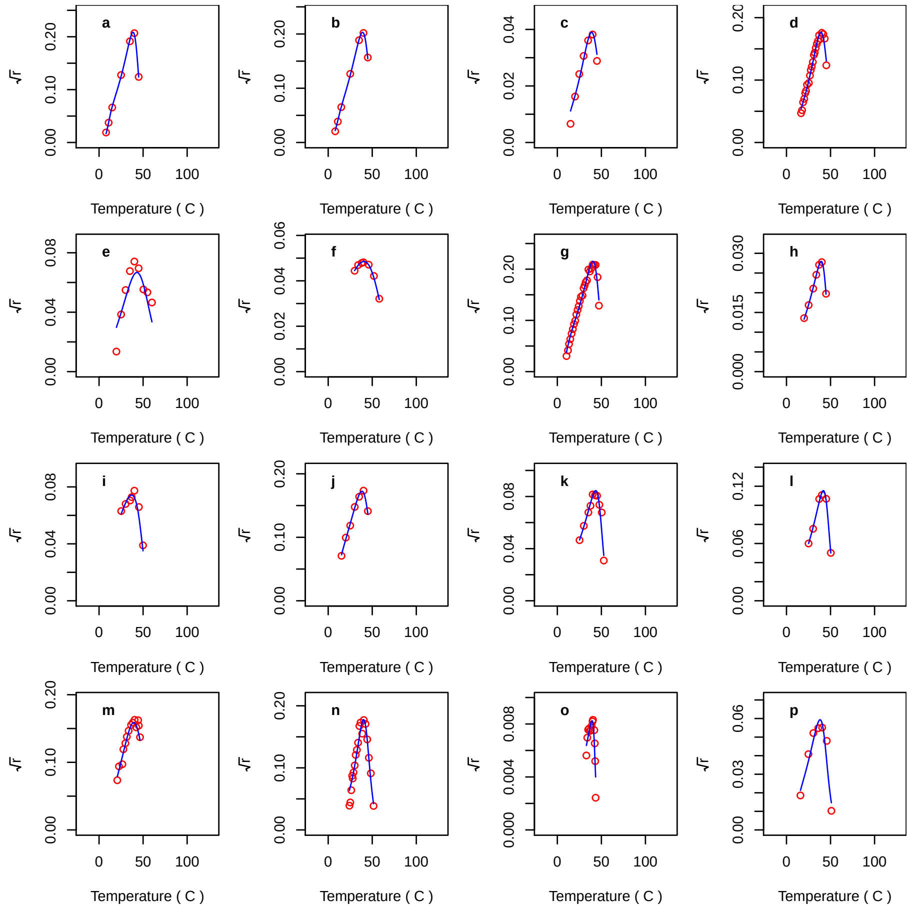

Figure 26. Fitted curve for strains. Fitted curve for strains: (a) *Escherichia coli* (strain 914) [248], (b) *Escherichia coli* (strain 916) [248], (c) *Methanosarcina acetivorans* (strain 978) [431], (d) *Streptococcus thermophilus* (strain 484) [412], (e) *Sulfobacillus thermotolerans* (strain 1312) [433], (f) *Alkalithermophilic Bacteria* (strain 362) [400], (g) *Escherichia coli* (strain 549) [403], (h) *Methanosarcina barkeri* (strain 1061) [272], (i) *Sporanaerobacter acetigenes* (strain 471) [435], (j) *Vibrio diabolicus* (strain 1047) [436], (k) *Porphyrobacter cryptus* (strain 866) [437], (l) *Pseudomonas thermotolerans* (strain 1258) [439], (m) *Bacillus megaterium* (strain 204) [39], (n) *Streptococcus thermophilus* (strain 488) [412], (o) *Synechocystis sp.* (strain 1494) [327], (p) *Halomonas mongoliensis* (strain 74) [440]. Shown for each is the mean posterior predicted curve and the observed data using circles. Strain codes in parentheses are listed in Table S2.

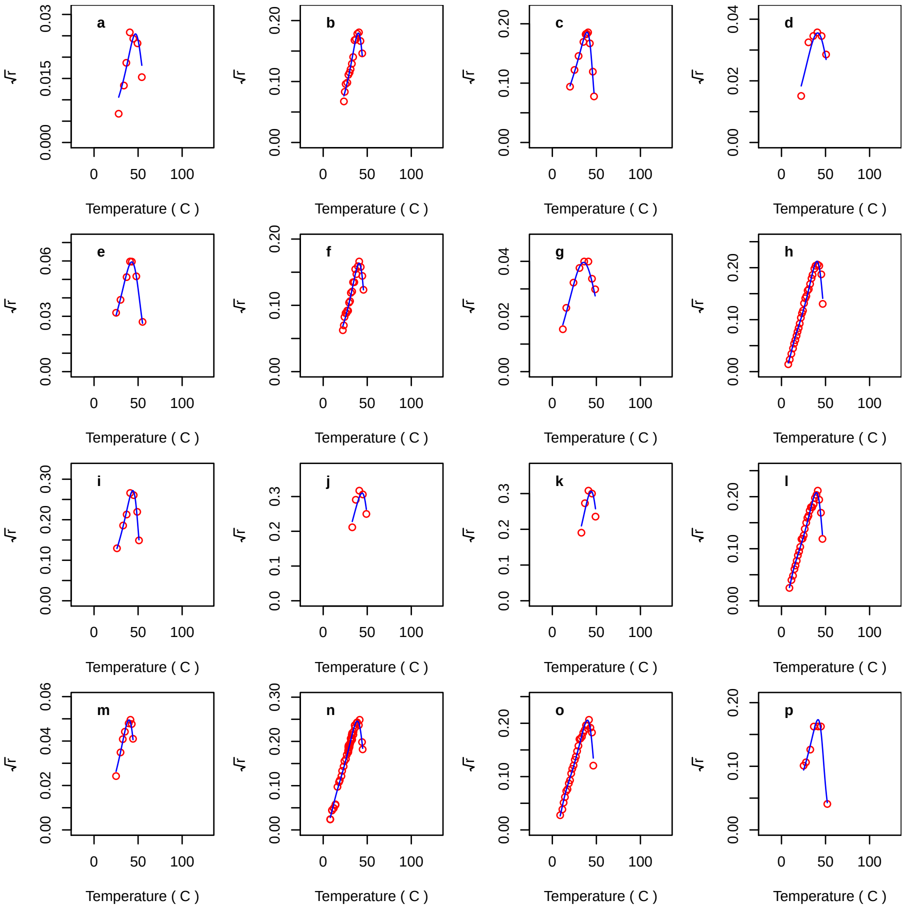

Figure 27. Fitted curve for strains. Fitted curve for strains: (a) *Isosphaera pallida* (strain 830) [441], (b) *Streptococcus thermophilus* (strain 487) [412], (c) *Klebsiella pneumoniae* (strain 945) [398], (d) *Haloanaerobiurn salsugo* (strain 406) [442], (e) *Methanoculleus marisnigri* (strain 1071) [415], (f) *Streptococcus thermophilus* (strain 481) [412], (g) *Halomonas kenyensis* (strain 73) [440], (h) *Escherichia coli* (strain 546) [403], (i) *Clostridium perfringens* (strain 1021) [444], (j) *Clostridium perfringens* (strain 1022) [444], (k) *Clostridium perfringens* (strain 1023) [444], (l) *Escherichia coli* (strain 555) [403], (m) *Anacystis nidulans* (strain 1315) [283], (n) *Escherichia coli* (strain 551) [403], (o) *Escherichia coli* (strain 552) [403], (p) *Sporohalobacter marismortui* (strain 152) [445]. Shown for each is the mean posterior predicted curve and the observed data using circles. Strain codes in parentheses are listed in Table S2.

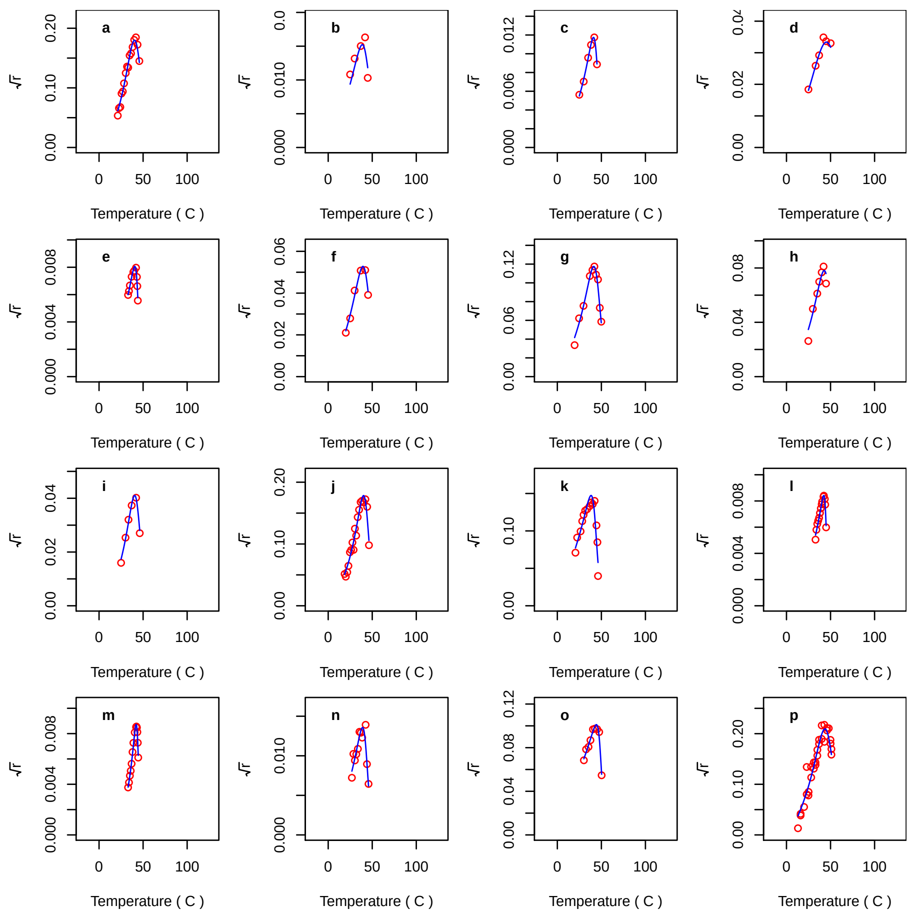

Figure 28. Fitted curve for strains. Fitted curve for strains: (a) *Streptococcus thermophilus* (strain 479) [412], (b) *Methanosarcina mazel* (strain 1062) [272], (c) *Methanosarcina barkeri* (strain 1059) [272], (d) *Methanolobus zinderi* (strain 59) [446], (e) *Synechocystis sp.* (strain 1489) [327], (f) *Methanosarcina mazel* (strain 1063) [272], (g) *Lactobacillus thermotolerans* (strain 1086) [449], (h) *Dethiosulfovibrio peptidovorans* (strain 1402) [450], (i) *Methanosarcina barkeri* (strain 1060) [272], (j) *Streptococcus thermophilus* (strain 483) [412], (k) *Pseudomonas aeruginosa* (strain 197) [39], (l) *Synechocystis sp.* (strain 1492) [327], (m) *Synechocystis sp.* (strain 1490) [327], (n) *Ferroplasma acidiphilum* (strain 493) [201], (o) *Porphyrobacter tepidarius* (strain 765) [451], (p) *Clostridium perfringens* (strain 44) [423]. Shown for each is the mean posterior predicted curve and the observed data using circles. Strain codes in parentheses are listed in Table S2.

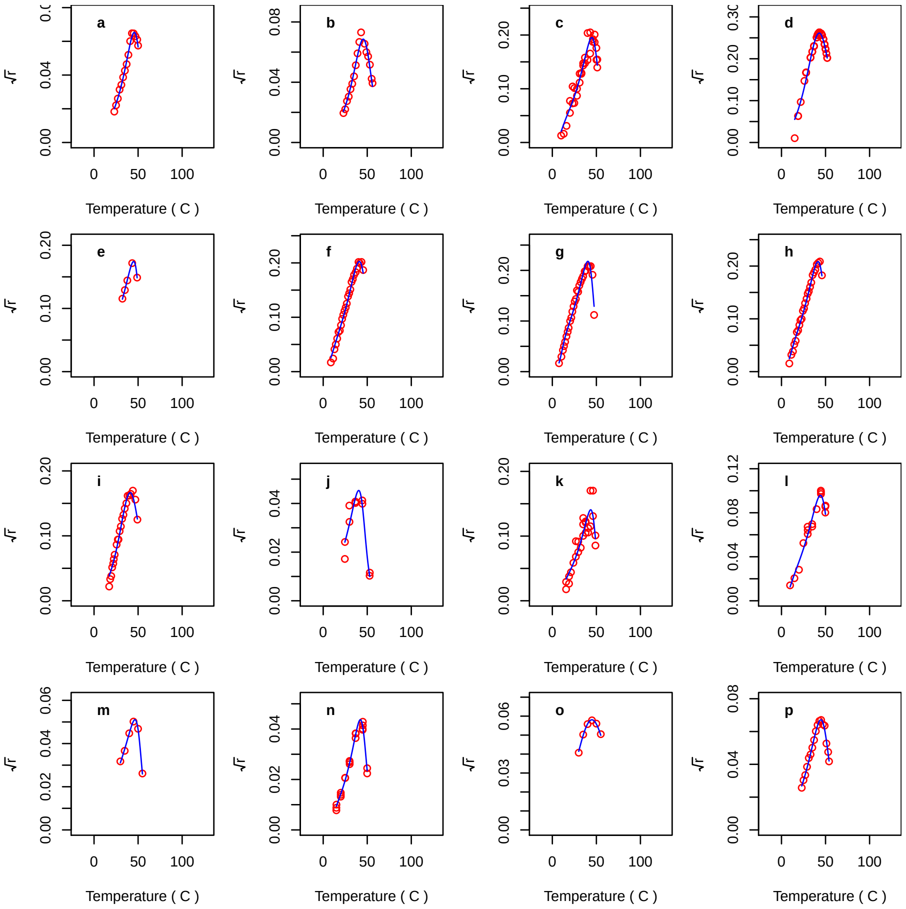

Figure 29. Fitted curve for strains. Fitted curve for strains: (a) *Natronomonas bangense* (strain 542) [452], (b) *Natronomonas pharaonis* (strain 543) [452], (c) *Clostridium perfringens* (strain 43) [423], (d) *Clostridium perfringens* (strain 853) [455], (e) *Clostridium perfringens* (strain 917) [456], (f) *Escherichia coli* (strain 554) [403], (g) *Escherichia coli* (strain 548) [403], (h) *Escherichia coli* (strain 553) [403], (i) *Streptococcus thermophilus* (strain 486) [412], (j) *Sporohalobacter lortetii* (strain 154) [458], (k) *Clostridium perfringens* (strain 918) [459], (l) *Methanococcus aeolicus* (strain 1097) [460], (m) *Rubrobacter radiotolerans* (strain 1074) [461], (n) *Methanoculleus submarinus* (strain 860) [462], (o) *Alkalithermophilic Bacteria* (strain 366) [400], (p) *Halobaculum gomorrense* (strain 532) [452]. Shown for each is the mean posterior predicted curve and the observed data using circles. Strain codes in parentheses are listed in Table S2.

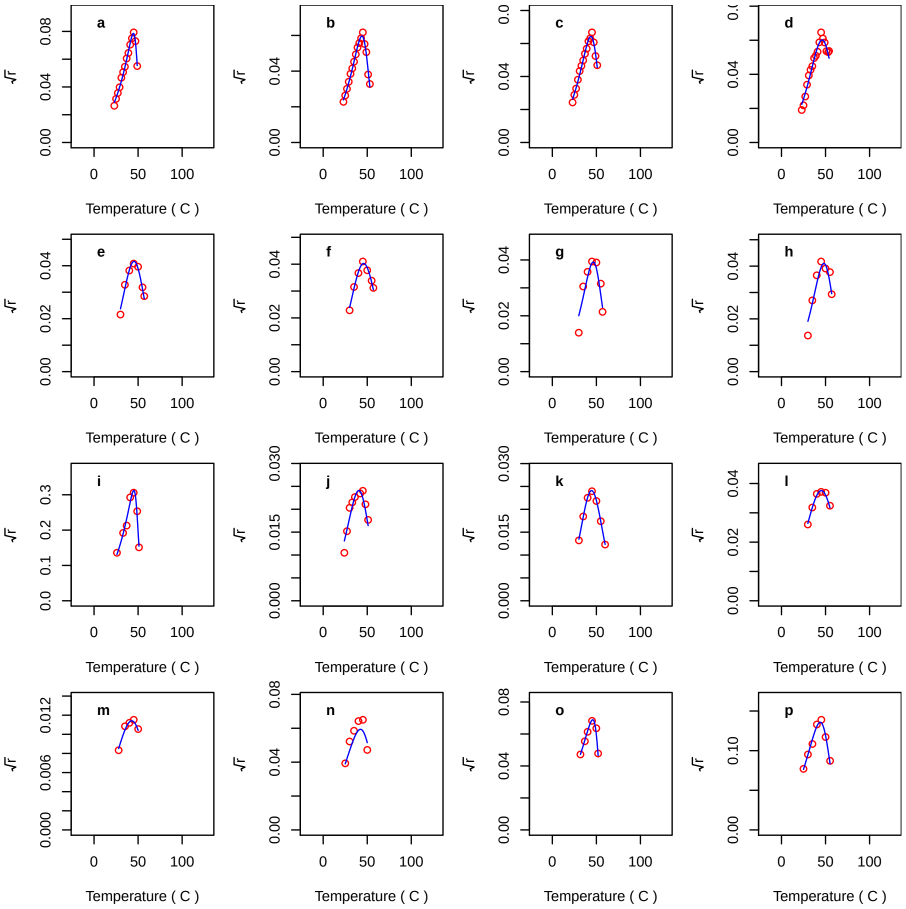

Figure 30. Fitted curve for strains. Fitted curve for strains: (a) *Haloferax volcanii* (strain 534) [452], (b) *Halorubrum saccharovorum* (strain 536) [452], (c) *Natrialba asiatica* (strain 538) [452], (d) *Natronococcus occultus* (strain 541) [452], (e) *Synechococcus clone* (strain 776) [470], (f) *Synechococcus clone* (strain 777) [470], (g) *Synechococcus clone* (strain 779) [470], (h) *Synechococcus clone* (strain 780) [470], (i) *Clostridium perfringens* (strain 1020) [444], (j) *Cyanidium caldarium* (strain 1429) [473], (k) *Geotoga subterranea* (strain 1146) [474], (l) *Alkalithermophilic Bacteria* (strain 367) [400], (m) *Halomicronema excentricum* (strain 842) [416], (n) *Haloanaerobacter chitinovorans* (strain 1323) [464], (o) *Thiobacillus caldus* (strain 840) [475], (p) *Thermomonas haemolytica* (strain 1259) [439]. Shown for each is the mean posterior predicted curve and the observed data using circles. Strain codes in parentheses are listed in Table S2.

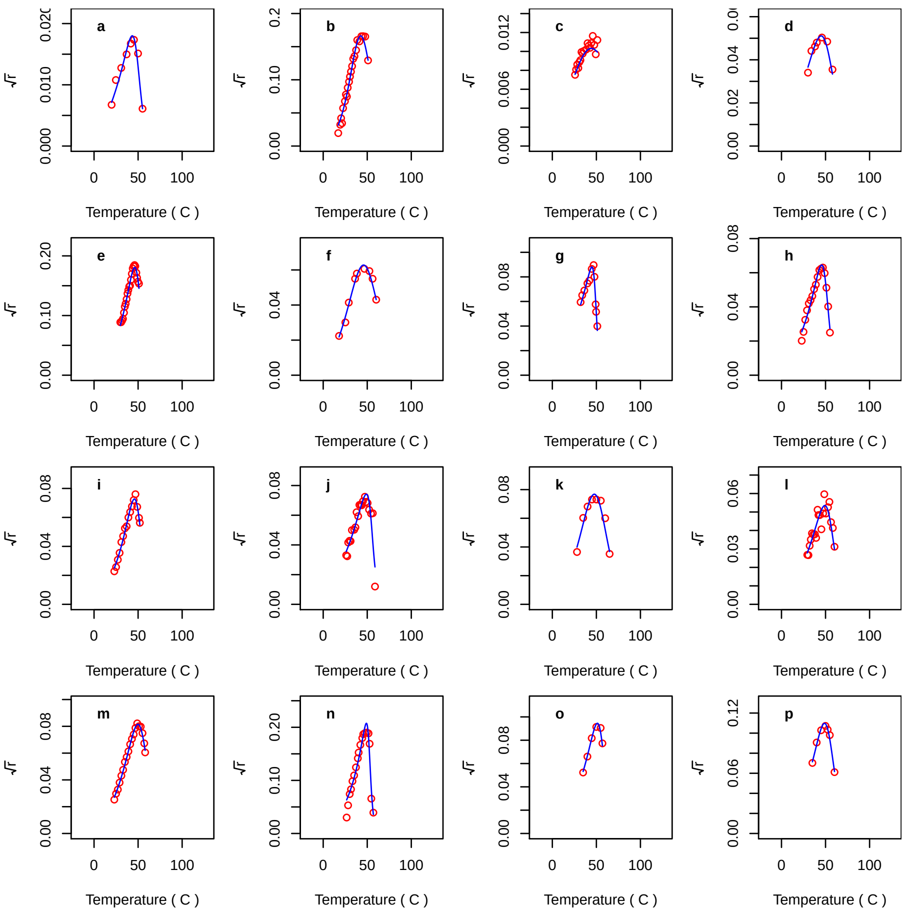

Figure 31. Fitted curve for strains. Fitted curve for strains: (a) *Methanosarcina barkeri* (strain 1055) [272], (b) *Streptococcus thermophilus* (strain 482) [412], (c) *Acidithiobacillus caldus* (strain 498) [100], (d) *Alkalithermophilic Bacteria* (strain 363) [400], (e) *Streptococcus thermophilus* (strain 33) [289], (f) *Halonatronum saccharophilum* (strain 991) [476], (g) *Chlorobium tepidum* (strain 815) [477], (h) *Haloarcula vallismortis* (strain 531) [452], (i) *Natronobacterium gregoryi* (strain 540) [452], (j) *Acidimicrobium ferrooxidans* (strain 489) [201], (k) *Geobacillus sp.* (strain 3) [478], (l) *Sulfobacillus thermosulfidooxidans* (strain 495) [201], (m) *Halogeometricum borinquense* (strain 535) [452], (n) *Clostridium thermoalcaliphilum* (strain 1394) [482], (o) *Coccobacillus sp.* (strain 947) [398], (p) *Alicyclobacillus acidoterrestris* (strain 1082) [484]. Shown for each is the mean posterior predicted curve and the observed data using circles. Strain codes in parentheses are listed in Table S2.

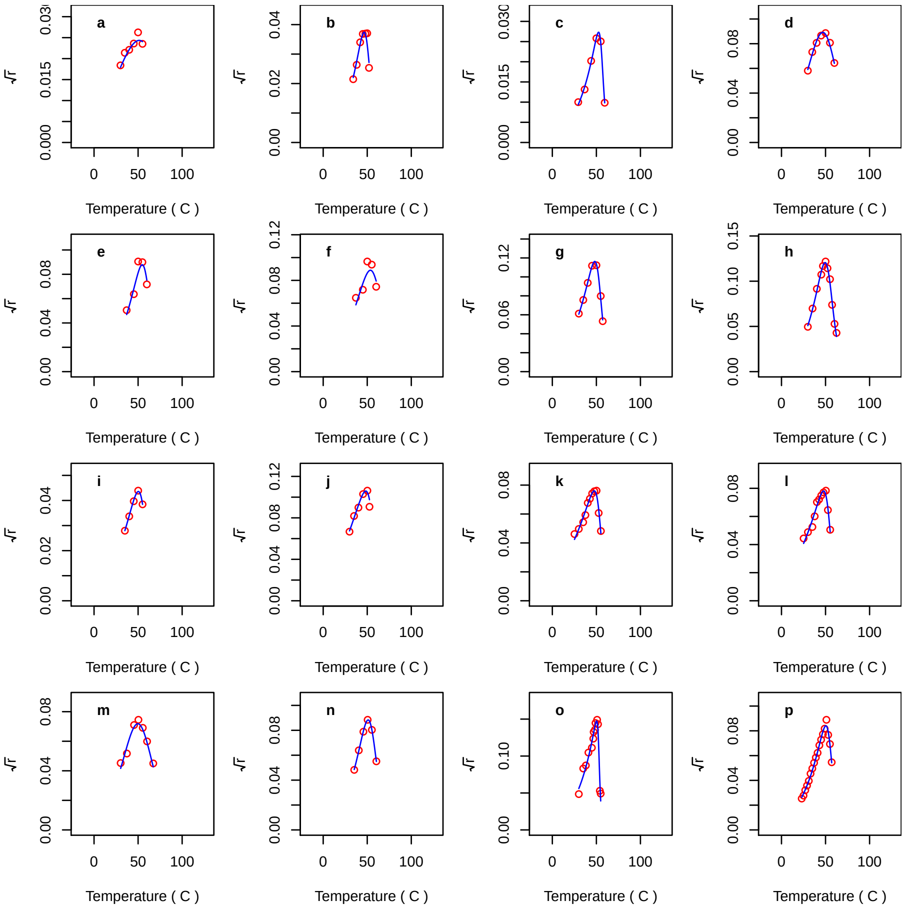

Figure 32. Fitted curve for strains. Fitted curve for strains: (a) *Geotoga petraea* (strain 1145) [474], (b) *Flexistipes sinusarabici* (strain 378) [485], (c) *Desulfotomaculum alkaliphilum* (strain 1163) [487], (d) *Pseudoxanthomonas taiwanensi* (strain 981) [262], (e) *Sulfurivirga caldicuralii* (strain 280) [498], (f) *Sulfurivirga caldicuralii* (strain 281) [498], (g) *Deinococcus geothermalis* (strain 1135) [499], (h) *Thermomonas hydrothermalis* (strain 1260) [439], (i) *Alkalithermophilic Bacteria* (strain 368) [400], (j) *Deinococcus murrayi* (strain 1136) [499], (k) *Porphyrobacter cryptus* (strain 868) [437], (l) *Porphyrobacter cryptus* (strain 867) [437], (m) *Lebetimonas acidiphila* (strain 751) [501], (n) *Thermus chliarophilus* (strain 1307) [502], (o) *Clostridium isatidis* (strain 854) [503], (p) *Halococcus morrhuae* (strain 533) [452]. Shown for each is the mean posterior predicted curve and the observed data using circles. Strain codes in parentheses are listed in Table S2.

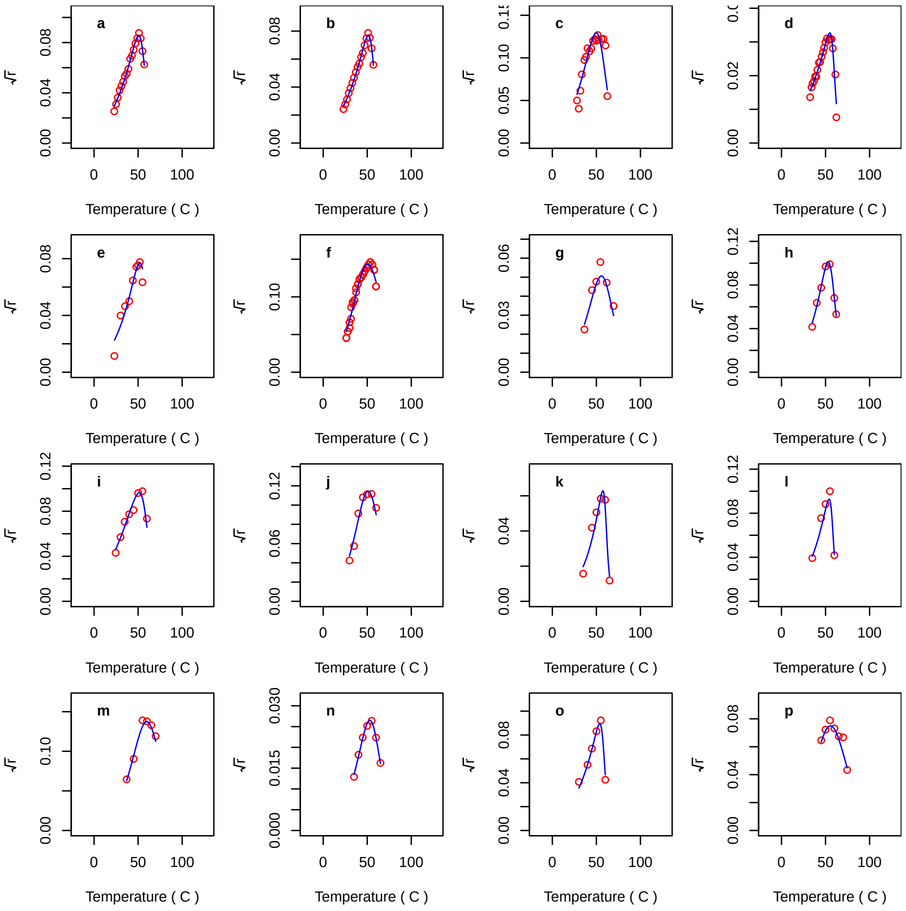

Figure 33. Fitted curve for strains. Fitted curve for strains: (a) *Haloterrigena turkmenica* (strain 537) [452], (b) *Natrinema pellirubrum* (strain 539) [452], (c) *Bacillus coagulans* (strain 203) [39], (d) *Ferroplasma cyprexacervatum* (strain 494) [201], (e) *Heliobacterium modesticaldum* (strain 375) [432], (f) *Clostridium thermobutyricum* (strain 23) [509], (g) *Methanobacterium thermoflexum* (strain 1579) [512], (h) *Thiobacter subterraneus* (strain 1085) [513], (i) *Nautilia nitratireducens* (strain 714) [514], (j) *Tepidimonas ignava* (strain 737) [516], (k) *Anaerobaculum mobile* (strain 1395) [517], (l) *Hydrogenimonas thermophila* (strain 771) [528], (m) *Geobacillus stearothermophilus* (strain 810) [529], (n) *Petrotoga miotherma* (strain 1147) [474], (o) *Sulfobacillus sibiricus* (strain 1427) [534], (p) *Thermoanaerobacter brockii* (strain 1401) [536]. Shown for each is the mean posterior predicted curve and the observed data using circles. Strain codes in parentheses are listed in Table S2.

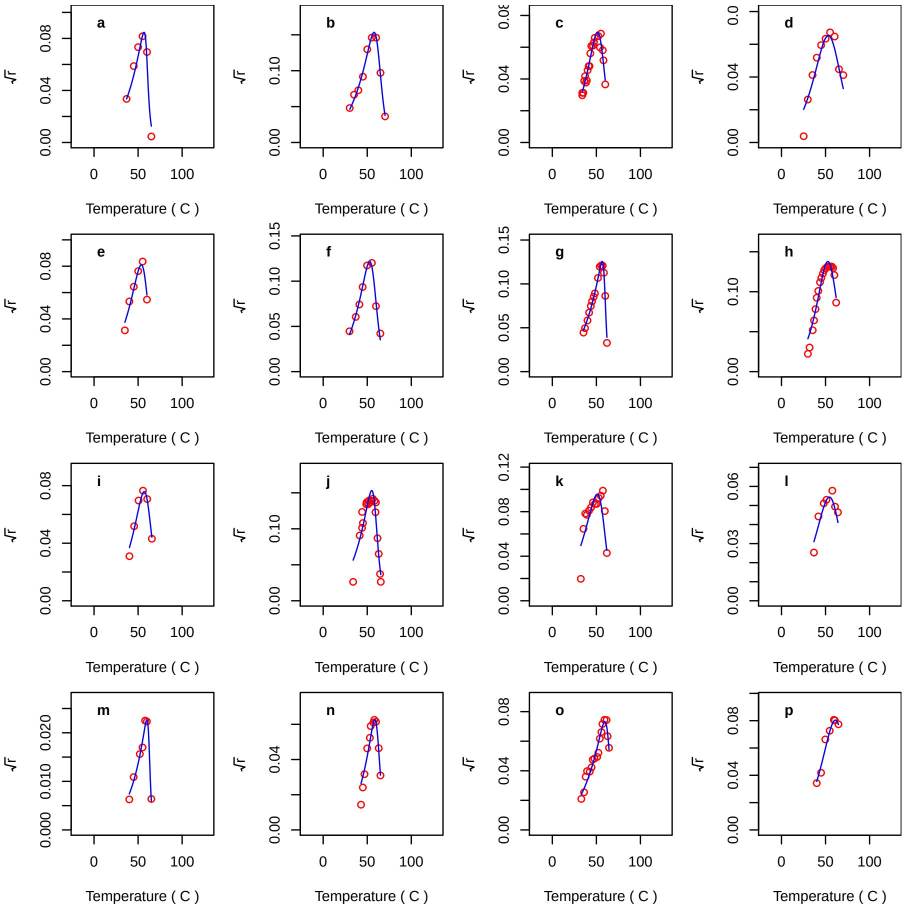

Figure 34. Fitted curve for strains. Fitted curve for strains: (a) *Methanogenium thermophilicum* (strain 4) [537], (b) *Marinitoga okinawensis* (strain 212) [538], (c) *Bacterial str.* (strain 17) [539], (d) *Rubrobacter taiwanensis* (strain 1075) [461], (e) *Meiothermus cerberus* (strain 797) [540], (f) *Anaerobranca gottschalkii* (strain 405) [542], (g) *Clostridium paradoxum* (strain 1396) [544], (h) *Clostridium paradoxum* (strain 1397) [544], (i) *Thermus silvanus* (strain 1309) [502], (j) *Anaerobranca horikoshii* (strain 1560) [545], (k) *Methanogenium frittonii* (strain 1538) [547], (l) *Methanogenium thermophilicum* (strain 1552) [550], (m) *Thermacetogenium phaeum* (strain 809) [552], (n) *Moorella gycerini* (strain 1537) [554], (o) *Caloramator viterbensis* (strain 408) [558], (p) *Clostridium thermosulfurogenes* (strain 846) [560]. Shown for each is the mean posterior predicted curve and the observed data using circles. Strain codes in parentheses are listed in Table S2.

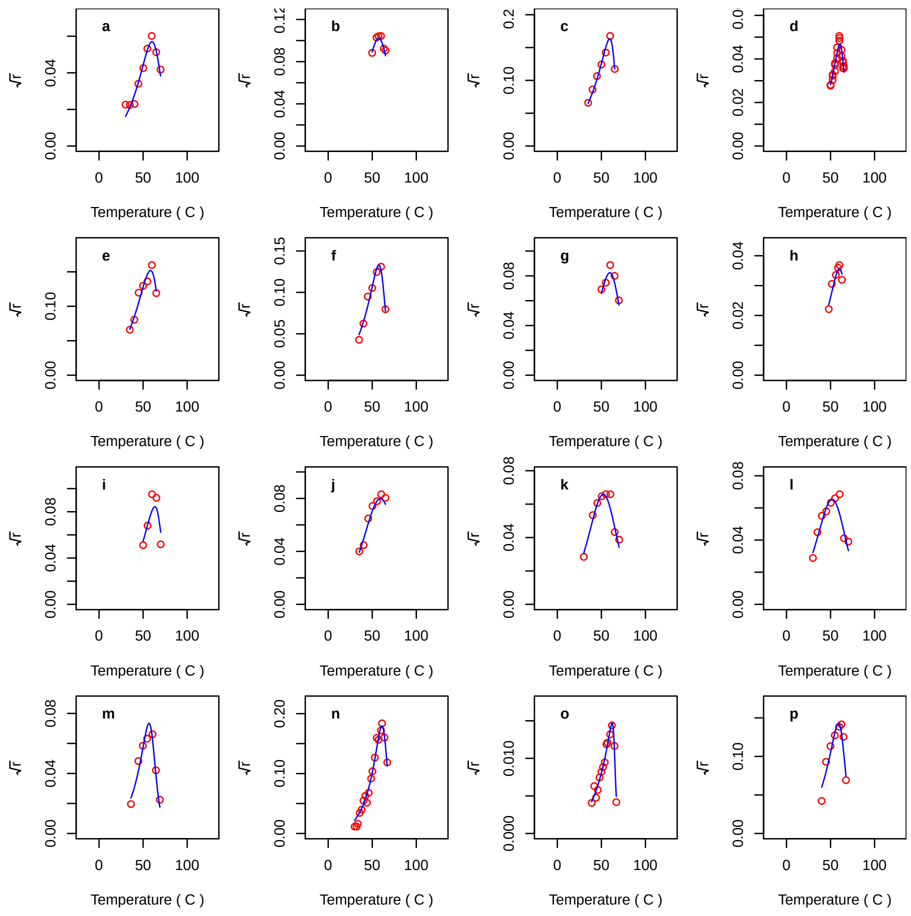

Figure 35. Fitted curve for strains. Fitted curve for strains: (a) *Rubrobacter xylanophilus* (strain 1562) [483], (b) *Deferribacter thermophilus* (strain 1398) [564], (c) *Thermonema rossianum* (strain 1556) [565], (d) *Thermomicrobium fosteri* (strain 1535) [566], (e) *Thermonema rossianum* (strain 1557) [565], (f) *Thermonema lapsum* (strain 1555) [565], (g) *Caminibacter hydrogeniphilus* (strain 334) [581], (h) *Picrophilus oshimae* (strain 800) [582], (i) *Desulfurobacterium crinifex* (strain 1587) [584], (j) *Thermus ruber* (strain 1308) [502], (k) *Rubrobacter taiwanensis* (strain 1076) [461], (l) *Rubrobacter xylanophilus* (strain 1077) [461], (m) *Methanobacterium defluvii* (strain 1578) [512], (n) *Thermoanaerobacter uzonensis* (strain 1080) [590], (o) *Alicyclobacillus sp.* (strain 1424) [592], (p) *Alicyclobacillus sp.* (strain 1084) [484]. Shown for each is the mean posterior predicted curve and the observed data using circles. Strain codes in parentheses are listed in Table S2.

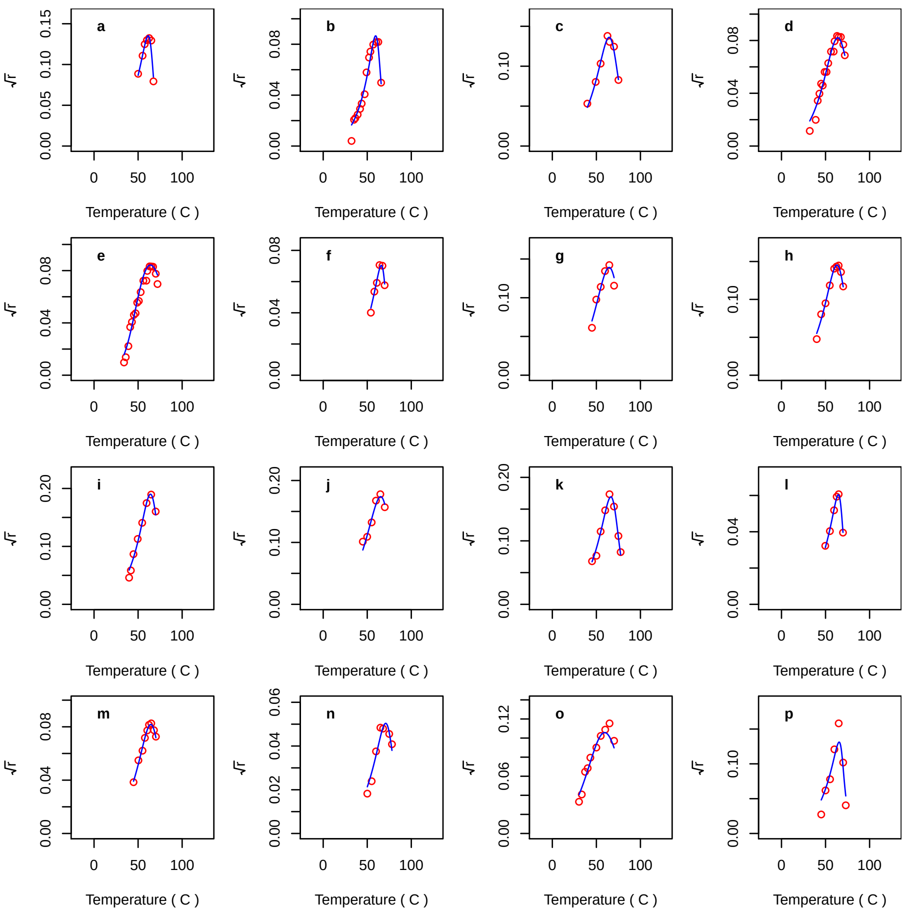

Figure 36. Fitted curve for strains. Fitted curve for strains: (a) *Hydrogenophilus hirschii* (strain 1264) [595], (b) *Thermoanaerobacterium aotearoense* (strain 1176) [596], (c) *Methanothermococcus okinawensis* (strain 1089) [597], (d) *Thermoanaerobacter sulfurigignens* (strain 1079) [590], (e) *Thermoanaerobacter sulfurigignens* (strain 716) [598], (f) *Thermoanaerobacter kivui* (strain 812) [599], (g) *Marinitoga piezophila* (strain 1042) [562], (h) *Alicyclobacillus acidocaldarius* (strain 1081) [484], (i) *Bacillus caldotenax* (strain 953) [603], (j) *Marinitoga piezophila* (strain 1041) [562], (k) *Hydrogenobacter hydrogenophilus* (strain 1569) [604], (l) *Thermotoga lettingae* (strain 845) [605], (m) *Methanobacterium thermoautotrophicum* (strain 955) [606], (n) *Caldicellulosiruptor acetigenus* (strain 10) [607], (o) *Methanococcus thermolithotrophic* (strain 1574) [438], (p) *Bacillus sp.* (strain 1426) [608]. Shown for each is the mean posterior predicted curve and the observed data using circles. Strain codes in parentheses are listed in Table S2.

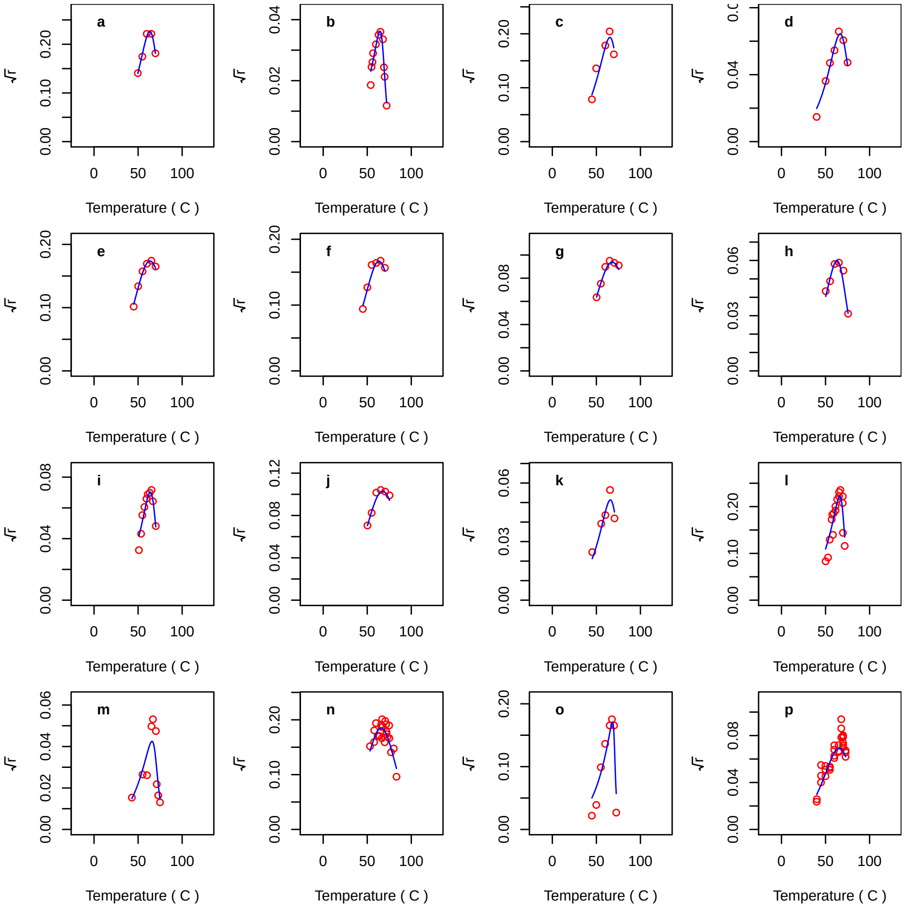

Figure 37. Fitted curve for strains. Fitted curve for strains: (a) *Bacillus caldotenax* (strain 952) [603], (b) *Synechococcus lividus* (strain 146) [609], (c) *Geobacillus thermoleovorans* (strain 811) [621], (d) *Thermoanaerobacter subterraneus* (strain 829) [624], (e) *Alicyclobacillus acidocaldarius* (strain 806) [626], (f) *Alicyclobacillus acidocaldarius* (strain 805) [626], (g) *Thermus sp.* (strain 1294) [627], (h) *Methanothermus fervidus* (strain 1283) [589], (i) *Firmicutes sp.* (strain 1325) [630], (j) *Thermus brockianus* (strain 1291) [627], (k) *Methanobacterium thermoaggregans* (strain 1276) [632], (l) *Thermobrachium celere* (strain 1565) [634], (m) *Thermobaculum terrenum* (strain 1245) [635], (n) *Thermus thermophilus* (strain 802) [636], (o) *Marinithermus hydrothermalis* (strain 1141) [641], (p) *Sulfurihydrogenibium kristjanssonii* (strain 711) [642]. Shown for each is the mean posterior predicted curve and the observed data using circles. Strain codes in parentheses are listed in Table S2.

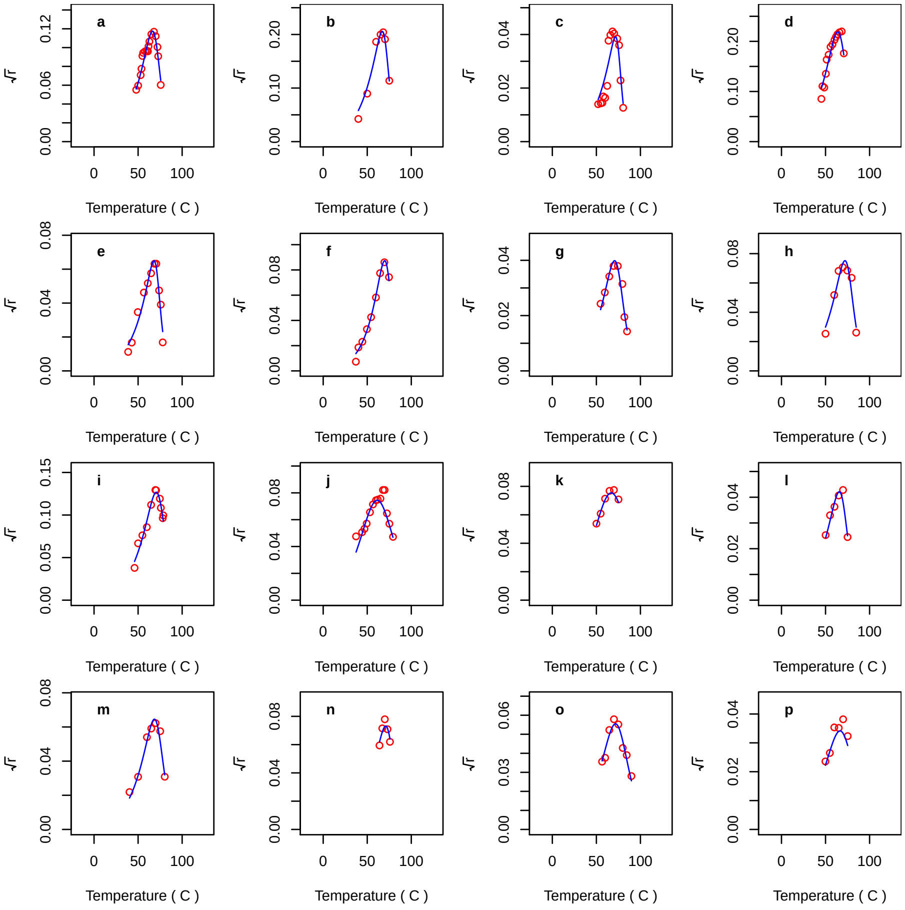

Figure 38. Fitted curve for strains. Fitted curve for strains: (a) *Clostridium thermohydrosulfuricum* (strain 1463) [646], (b) *Thermosipho globiformans* (strain 720) [647], (c) *Acidianus brierleyi* (strain 490) [201], (d) *Geobacillus stearothermophilus* (strain 206) [39], (e) *Thermoanaerobacter siderophilus* (strain 1409) [649], (f) *Desulfurobacterium thermolithotrophum* (strain 1536) [651], (g) *Thermotoga caldifontis* (strain 217) [586], (h) *Thermodesulfobacterium commune* (strain 841) [652], (i) *Thermus aquaticus* (strain 803) [653], (j) *Thermoanaerobacter ethanolicus* (strain 1106) [654], (k) *Thermosipho melanesiensis* (strain 986) [656], (l) *Thermotoga subterranea* (strain 985) [657], (m) *Fervidobacterium pennivorans* (strain 1142) [658], (n) *Ammonifex degensii* (strain 1287) [659], (o) *Thermotoga hypogea* (strain 1406) [660], (p) *Sulfolobus metallicus* (strain 1284) [623]. Shown for each is the mean posterior predicted curve and the observed data using circles. Strain codes in parentheses are listed in Table S2.

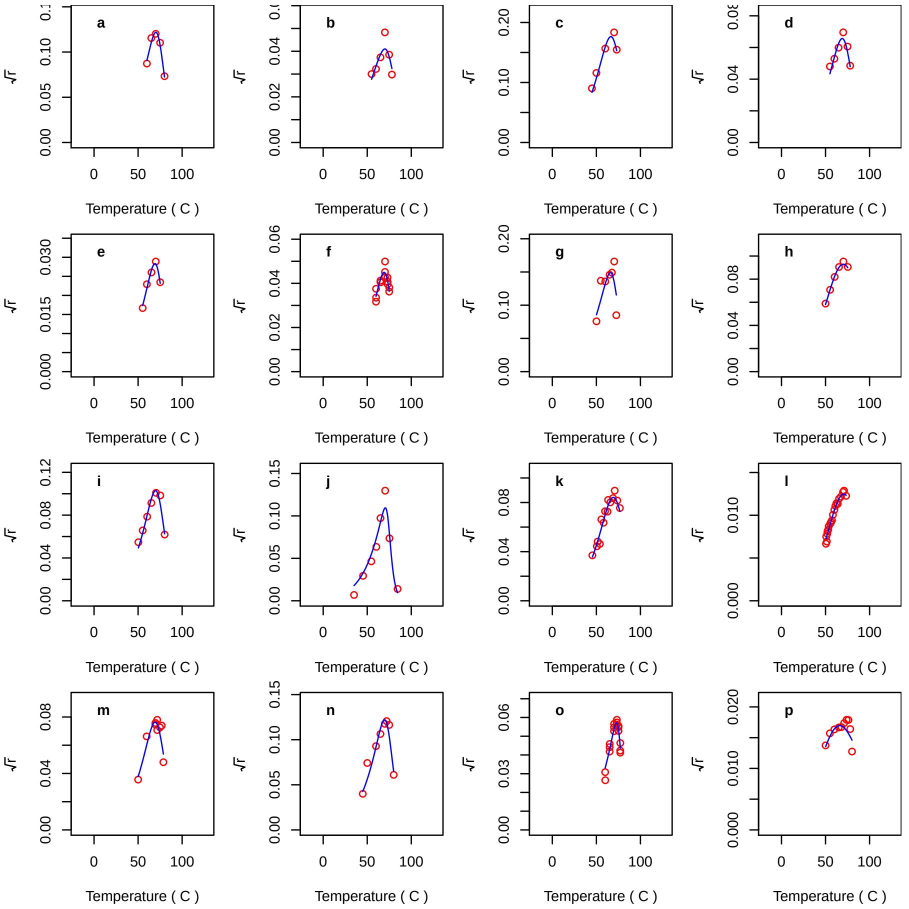

Figure 39. Fitted curve for strains. Fitted curve for strains: (a) *Thermus sp.* (strain 332) [665], (b) *Thermosulfidibacter takaii* (strain 705) [670], (c) *Geobacillus sp.* (strain 1170) [671], (d) *Sulfurihydrogenibium yellowstonense* (strain 1094) [672], (e) *Sulfolobus hakonensis* (strain 1262) [673], (f) *Persephonella guaymasensis* (strain 838) [675], (g) *Persephonella hydrogeniphil* (strain 708) [676], (h) *Thermus scotoductus* (strain 1293) [627], (i) *Thermus igniterrae* (strain 1292) [627], (j) *Thermoanaerobium brockii* (strain 1585) [678], (k) *Thermus aquaticus* (strain 207) [39], (l) *Sulfolobus metallicus* (strain 492) [201], (m) *Hydrogenobacter thermophilus* (strain 835) [680], (n) *Thermosipho japonicus* (strain 982) [681], (o) *Persephonella marina* (strain 839) [675], (p) *Thermaerobacter marianensis* (strain 478) [683]. Shown for each is the mean posterior predicted curve and the observed data using circles. Strain codes in parentheses are listed in Table S2.

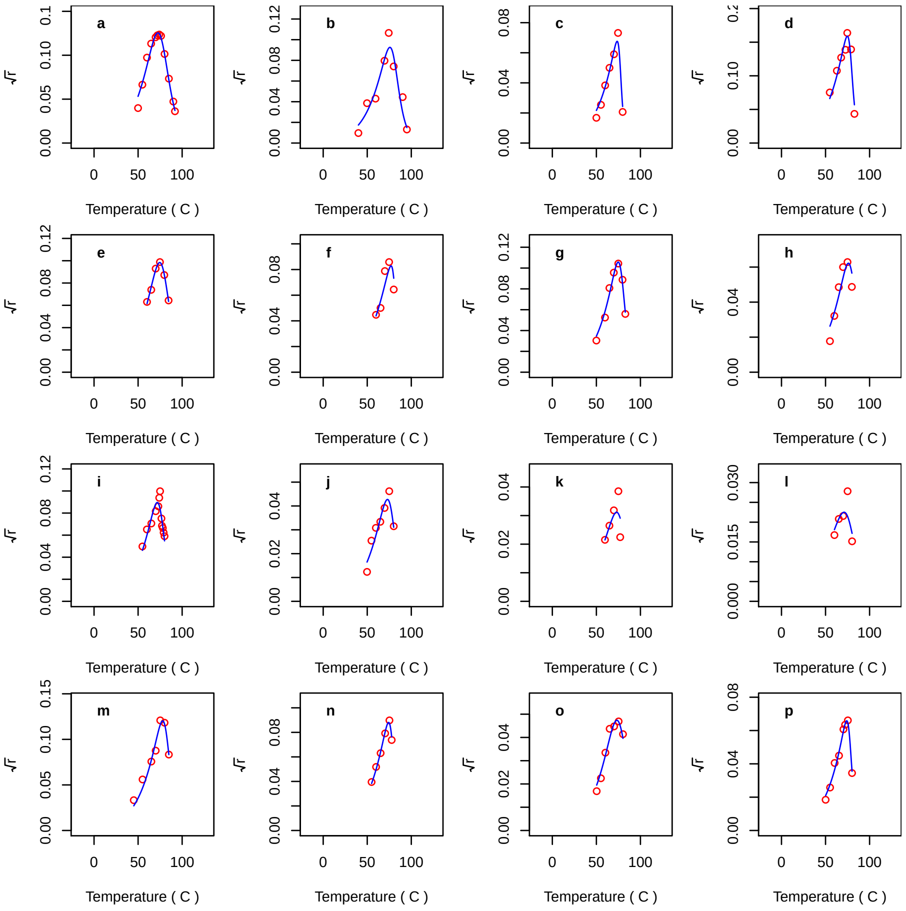

Figure 40. Fitted curve for strains. Fitted curve for strains: (a) *Thermosulfurimonas dismutans* (strain 729) [684], (b) *Caldicellulosiruptor changbaiensis* (strain 814) [685], (c) *Thermodesulfobacterium hydrogeniphilum* (strain 1090) [686], (d) *Methanotorris formicicus* (strain 709) [687], (e) *Thermococcus stetteri* (strain 1577) [688], (f) *Thermovibrio ammonificans* (strain 1088) [689], (g) *Thermoanaerobacter yonseiensis* (strain 1410) [690], (h) *Metallosphaera prunae* (strain 1174) [691], (i) *Sulfurihydrogenibium rodmanii* (strain 710) [692], (j) *Caldicellulosiruptor owensensis* (strain 1160) [693], (k) *Sulfolobus sp.* (strain 799) [694], (l) *Thermocladium modestius* (strain 1091) [698], (m) *Palaeococcus helgesonii* (strain 959) [700], (n) *Hydrogenivirga caldilitoris* (strain 1087) [701], (o) *Metallosphaera sedula* (strain 1175) [702], (p) *Thermoanaerobacter tengcongensis* (strain 837) [703]. Shown for each is the mean posterior predicted curve and the observed data using circles. Strain codes in parentheses are listed in Table S2.

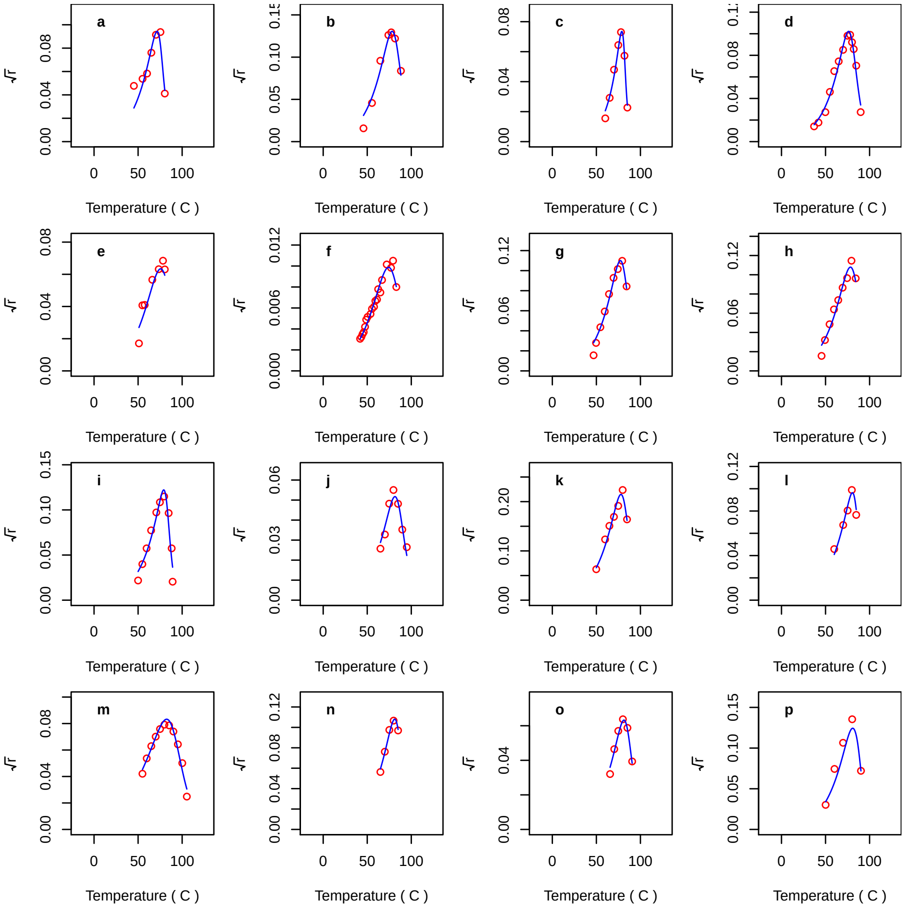

Figure 41. Fitted curve for strains. Fitted curve for strains: (a) *Balnearium lithotrophicum* (strain 1099) [704], (b) *Eubacteria sp.* (strain 957) [707], (c) *Hydrogenobacter subterraneus* (strain 983) [709], (d) *Caldicellulosiruptor bescii* (strain 11) [710], (e) *Dictyoglomus thermophilum* (strain 951) [715], (f) *Acidianus brierleyi* (strain 491) [201], (g) *Thermotoga naphthophila* (strain 975) [716], (h) *Thermotoga petrophila* (strain 976) [716], (i) *Methanococcus vulcanius* (strain 1043) [718], (j) *Sulfolobus yangmingensis* (strain 1269) [719], (k) *Thermococcus celericrescens* (strain 703) [720], (l) *Thermocrinis ruber* (strain 850) [721], (m) *Thermococcus hydrothermalis* (strain 1046) [722], (n) *Archaeoglobus veneficus* (strain 1553) [723], (o) *Methanobacterium thermoautotrophicum* (strain 1281) [589], (p) *Thermococcus profundus* (strain 1582) [727]. Shown for each is the mean posterior predicted curve and the observed data using circles. Strain codes in parentheses are listed in Table S2.

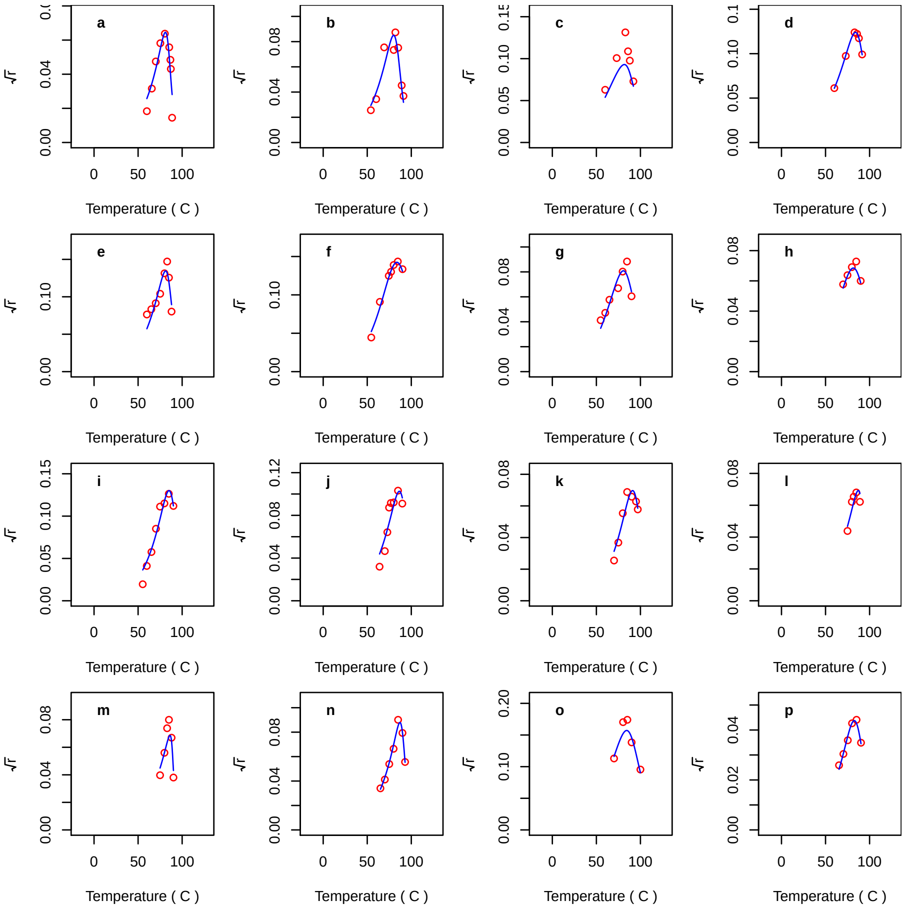

Figure 42. Fitted curve for strains. Fitted curve for strains: (a) *Stygiolobus azoricus* (strain 1265) [728], (b) *Archaebacterial str.* (strain 1024) [730], (c) *Thermococcus barossii* (strain 961) [732], (d) *Thermococcus celer* (strain 962) [732], (e) *Palaeococcus ferrophilus* (strain 979) [733], (f) *Desulfurococcus strain* (strain 336) [736], (g) *Thermococcus alcaliphilus* (strain 987) [737], (h) *Thermococcus siculi* (strain 476) [739], (i) *Methanococcus infernus* (strain 1070) [740], (j) *Thermococcus waiotapuensis* (strain 960) [742], (k) *Aeropyrum camini* (strain 1100) [743], (l) *Acidilobus aceticus* (strain 1277) [744], (m) *Thermosphaera aggregans* (strain 1030) [749], (n) *Thermococcus chitonophagus* (strain 958) [750], (o) *Thermococcus peptonophilus* (strain 474) [752], (p) *Sulfurisphaera ohwakuensis* (strain 984) [753]. Shown for each is the mean posterior predicted curve and the observed data using circles. Strain codes in parentheses are listed in Table S2.

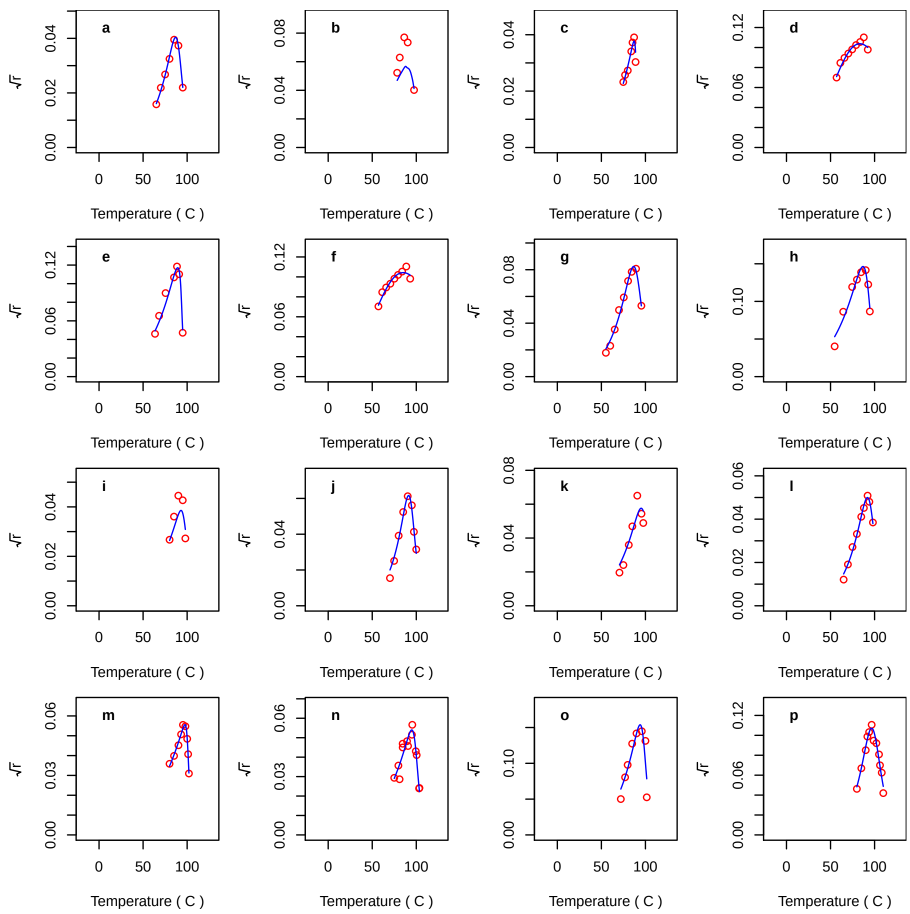

Figure 43. Fitted curve for strains. Fitted curve for strains: (a) *Sulfolobus tengchongensis* (strain 1278) [754], (b) *Thermoproteus uzoniensis* (strain 831) [756], (c) *Sulfolobus sp.* (strain 798) [694], (d) *Thermococcus nautili* (strain 722) [759], (e) *Archaebacterial str.* (strain 956) [761], (f) *Thermococcus prieurii* (strain 733) [762], (g) *Thermococcus gammatolerans* (strain 707) [763], (h) *Desulfurococcus strain* (strain 337) [736], (i) *Pyrobaculum calidifontis* (strain 863) [765], (j) *Aeropyrum pernix* (strain 1098) [769], (k) *Pyrobaculum oguniense* (strain 1261) [770], (l) *Staphylothermus marinus* (strain 1018) [738], (m) *Stetteria hydrogenophila* (strain 1280) [771], (n) *Pyrococcus glycovorans* (strain 335) [772], (o) *Pyrococcus abyssi* (strain 828) [773], (p) *Pyrodictium abyssi* (strain 1319) [774]. Shown for each is the mean posterior predicted curve and the observed data using circles. Strain codes in parentheses are listed in Table S2.

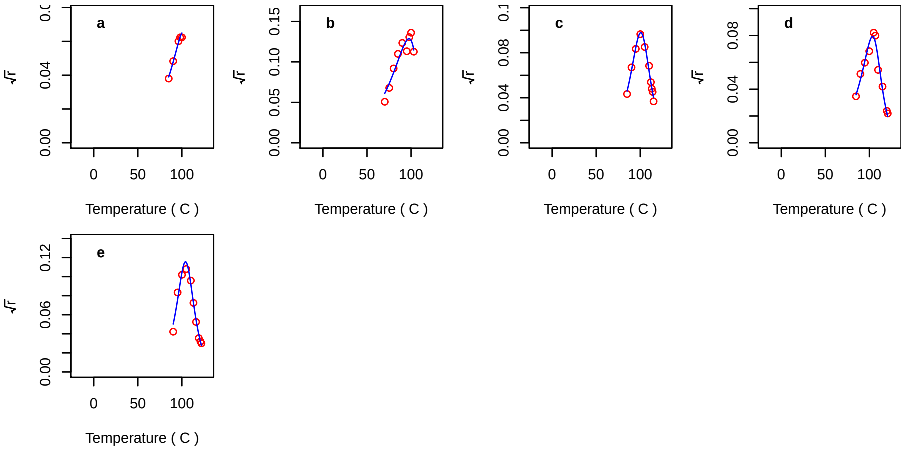

Figure 44. Fitted curve for strains. Fitted curve for strains: (a) *Pyrobaculum aerophilum* (strain 862) [778], (b) *Pyrococcus furiosus* (strain 966) [780], (c) *Methanopyrus kandleri* (strain 428) [782], (d) *Archaeal str.* (strain 770) [783], (e) *Methanopyrus kandleri* (strain 427) [782]. Shown for each is the mean posterior predicted curve and the observed data using circles. Strain codes in parentheses are listed in Table S2.
