# Supplementary material for: The Biokinetic Spectrum for Temperature
Source: PLoS One. 2016 Apr 18;11(4):e0153343. doi: 10.1371/journal.pone.0153343 (PMC4835062; doi:10.1371/journal.pone.0153343)
Supplement: S1 Table — The strain posterior parameter estimates are shown first, followed by universal posterior parameter estimates. For the strains are shown the strain code, strain name, c (scaling constant), ΔHA‡ (enthalpy of activation, J/mol) ΔCP (heat capacity change, J/K mol-amino acid-residue), and n (number of amino acid residues). At the bottom of the table are shown the universal posterior parameter estimates consisting of ΔH* (enthalpy change, J/mol amino acid residue), ΔS* (entropy change, J/K), TH* (convergence temperature for enthalpy, K), and TS* (convergence temperature for entropy, K). Strains are sorted by Topt. (PDF) [file pone.0153343.s002.pdf]

Table S1. Posteriors for the thermodynamic model parameters. The strain posterior parameter estimates are shown first, followed by universal posterior parameter estimates. For the strains are shown the strain code, strain name, c (scaling constant), $\Delta H_A^\ddagger$ (enthalpy of activation, J/mol) ΔC_P (heat capacity change, J/K mol-amino acid-residue), and n (number of amino acid residues). At the bottom of the table are shown the universal posterior parameter estimates consisting of $\Delta H^\star$ (enthalpy change, J/mol amino acid residue), $\Delta S^\star$ (entropy change, J/K), $T_H^\star$ (convergence temperature for enthalpy, K), and $T_S^\star$ (convergence temperature for entropy, K). Strains are sorted by T_{opt}.

Code	Strain/species name	c	$\Delta H_A^\ddagger$	ΔC_P	n	T_{mes}
876	*Clostridium sp.*	12.4	59305	32.88	296.9	225
877	*Clostridium algoriphilium*	6.7	46309	30.88	272.2	216
1444	*Stichococcus sp.*	3.7	42485	35.01	94.7	235
178	*Desulfofrigus marinus*	6.9	52227	30.09	1739.3	193
180	*Desulfotalea psychrophila*	-7.8	13057	51.97	568.8	282
1466	*Synura sphagnicola*	24.9	96599	40.34	322.2	251
1467	*Synura sphagnicola*	25.2	96514	40.14	292.4	244
859	*Methanogenium frigidum*	22.9	87777	41.73	288.6	257
1359	*Arcobacter sp.*	18.9	71697	35.66	137.7	237
1366	*Clostridium lacusfryxellense*	-7.0	9610	32.92	4290.9	221
1355	*Pseudomonas sp.*	13.7	57797	30.32	105.8	205
879	*Colwellia hornerae*	12.0	55061	33.78	173.5	228
1127	*Cryptomonas marssonii*	13.2	61643	46.40	173.9	269
1051	*Psychroflexus torquis*	3.0	37238	42.79	110.1	257
1363	*Clostridium bowmanii*	17.1	65943	40.70	146.4	253
1414	*Brochothrix thermosphacta*	40.7	128146	46.16	3358.4	265
996	*Psychromonas antarcticus*	23.7	84988	40.84	260.6	254
1005	*Clostridium vincentii*	26.8	93764	39.75	304.6	251
1543	*Chlamydomonas raudensis*	16.1	72810	42.71	194.9	261
1440	*Chlamydomonas alpina*	48.6	145090	37.37	391.7	243
1443	*Chlamydomonas subcaudata*	13.6	61823	45.10	664.7	267
1468	*Synura sphagnicola*	28.1	102062	43.11	300.5	261
1470	*Synura sphagnicola*	25.5	94397	47.50	280.8	272
819	*Candida curiosa*	13.2	78919	31.29	308.3	216
1342	*Flavobacterium segetis*	19.2	83136	40.21	261.0	252
878	*Colwellia demingiae*	16.5	65045	24.91	157.3	175
1381	*Arthrobacter glacialis*	12.7	58908	44.83	182.8	266
200	*Vibrio marinus*	14.3	60660	52.78	267.5	282
522	*Glaciecola punicea*	17.2	70131	44.67	190.0	264
861	*Photobacterium frigidiphilum*	16.5	64049	32.12	296.1	223
881	*Colwellia psychrerythraea*	16.0	62550	50.30	264.6	279
396	*Psychrobacter glacincola*	9.0	45546	47.63	382.7	272
92	*Cyanobacterial str.*	16.2	72943	36.67	83.2	234
97	*Cyanobacterial str.*	16.8	73226	45.18	151.0	263
1380	*Arthrobacter glacialis*	18.7	73620	45.42	376.2	267
82	*Cyanobacterial str.*	15.5	70579	39.13	92.2	241
502	*Pseudomonas sp.*	13.5	58188	46.92	381.7	271
83	*Cyanobacterial str.*	16.2	72226	50.60	203.0	279
184	*Mucor racemosus*	18.5	71922	43.56	151.4	259
1194	*Gonyaulax tamarensis*	18.9	76151	48.14	195.2	273
1464	*Synura sphagnicola*	28.4	100304	47.50	320.5	270
1469	*Synura sphagnicola*	29.7	105154	45.40	301.7	265
1471	*Synura sphagnicola*	30.2	104736	47.61	335.8	271
85	*Cyanobacterial str.*	16.6	75925	50.74	268.7	278
265	*Clostridium tagluense*	18.4	72693	29.96	63.6	205
523	*Shewanella gelidimarina*	13.2	56947	50.17	341.3	278
525	*Shewanella gelidimarina*	13.2	56960	50.16	340.5	278
1357	*Shewanella sp.*	25.7	85164	49.28	321.8	277
94	*Cyanobacterial str.*	19.7	81133	49.42	176.8	274
1368	*Salmo gairdneri*	19.5	83724	49.44	242.9	277
1343	*Flavobacterium weaverense*	16.7	74907	42.22	199.0	258

(Table S1 continued.)

Code	Strain/species	c	$\Delta H_A^\ddagger$	ΔC_P	n	T_{mes}
1378	*Rhodomonas salina*	24.1	87585	48.06	179.9	273
86	*Cyanobacterial str.*	2.8	40975	49.96	123.9	274
524	*Shewanella gelidimarina*	19.4	70733	50.78	380.2	279
1124	*Asterionella formosa*	15.9	66760	50.44	171.4	279
1128	*Dinobryon divergens*	15.6	67976	48.69	206.5	275
1029	*Shewanella donghaensis*	21.4	75654	46.81	219.1	270
370	*Pseudoalteromonas antarctica*	18.9	71202	51.08	321.2	280
880	*Colwellia psychotropica*	10.3	50526	47.74	547.9	273
1362	*Psychrobacter muriicola*	27.6	94318	51.61	278.0	281
1052	*Rhodoglobus vestalii*	19.2	75148	48.25	290.0	274
1452	*Chlorella sp.*	18.4	76635	31.19	59.6	208
1456	*Hantzschia amphyoxis*	20.5	78402	49.43	195.8	277
177	*Desulfofrigus fragile*	9.3	52616	47.25	102.0	271
1001	*Methanolobus psychrophilus*	10.4	55372	55.67	311.6	287
874	*Psychrobacter sp.*	26.7	92785	52.97	284.2	283
875	*Psychrobacter sp.*	22.3	82328	51.54	273.4	280
1450	*Chlamydomonas sp.*	17.3	73812	34.48	91.2	216
179	*Desulfotalea arctica*	11.4	58966	49.15	263.5	276
1418	*Aeromonas hydrophila*	20.6	81523	50.55	206.7	278
1352	*Thiobacillus sp.*	18.4	72778	50.95	309.5	279
1361	*Bacterial str.*	17.6	67847	53.54	229.0	284
88	*Cyanobacterial str.*	15.5	70461	44.36	114.1	263
425	*Acyrthosiphon pisum*	15.5	70705	52.91	194.6	283
599	*Aphis gossypii*	10.0	56154	49.59	147.3	277
1207	*Thalassiosira rotula*	16.7	68771	48.55	135.0	274
1420	*Aeromonas hydrophila*	19.5	78689	55.33	303.7	287
586	*Desulfobacter curvatus*	23.5	87659	51.67	224.0	281
80	*Cyanobacterial str.*	17.5	75288	49.44	184.0	270
994	*Acetobacterium paludosum*	18.7	71701	46.32	138.5	268
1134	*Phaeodactylum tricornutum*	18.8	74145	47.38	291.3	268
1374	*Skeletonema costatum*	18.8	75358	45.45	131.1	263
1126	*Tychonema bourrellyi*	14.2	64694	54.72	298.0	286
1447	*Chlorella sp.*	5.9	46569	36.28	54.7	237
1026	*Acetobacterium tundrae*	18.0	74248	54.39	221.3	285
1032	*Desulfobacter psychrotolerans*	27.9	99473	50.60	358.7	279
1200	*Thalassiosira rotula*	16.3	68037	52.53	196.5	282
858	*Rhodoferax antarcticus*	24.8	89480	50.14	317.2	278
1372	*Ditylum brightwellii*	17.3	72190	46.07	124.1	267
1373	*Phaeodactylum tricornutum*	14.4	64688	52.87	239.1	283
1206	*Thalassiosira rotula*	23.6	85602	52.19	224.7	282
89	*Cyanobacterial str.*	12.1	64442	57.70	222.8	290
1614	*Flavobacterium antarcticum*	19.2	73393	50.24	257.7	278
1053	*Clostridium gasigenes*	24.2	83533	54.69	330.7	286
556	*Candida stellata*	0.4	30576	59.42	521.6	293
557	*Hanseniaspora uvarum*	12.4	57762	58.26	361.7	292
559	*Pichia fermentans*	-6.0	12584	61.04	266.4	296
574	*Saccharomyces kudriavzevii*	12.6	57746	57.46	336.6	291
575	*Saccharomyces kudriavzevii*	6.7	42882	58.54	364.1	292
576	*Saccharomyces kudriavzevii*	11.9	55489	57.79	363.5	291
577	*Saccharomyces kudriavzevii*	12.0	55575	57.73	319.8	291
582	*Torulaspora delbrueckii*	3.9	37264	60.49	304.2	295
501	*Methanococcoides burtonii*	18.9	80733	55.96	547.3	288
424	*Acyrthosiphon pisum*	16.0	71220	50.44	125.5	279
172	*Cellulomonas sp.*	20.7	76197	54.44	213.9	286
84	*Cyanobacterial str.*	15.5	70218	49.63	165.1	277
521	*Gelidibacter sp.*	5.9	42767	56.43	207.5	289
100	*Cyanobacterial str.*	13.4	66794	54.80	311.0	286
1385	*Bacillus circulans*	15.8	62864	54.56	181.6	286
1095	*Flavobacterium hibernum*	21.1	78507	54.43	193.2	286
181	*Mucor racemosus*	18.9	71326	57.59	362.5	291
182	*Mucor racemosus*	18.8	71032	57.47	281.5	291
183	*Mucor racemosus*	18.9	71600	56.65	289.5	289
185	*Mucor racemosus*	19.2	71956	56.49	263.3	289
186	*Mucor racemosus*	19.2	72022	56.21	215.2	289
187	*Mucor racemosus*	19.1	71932	55.43	196.0	288

(Table S1 continued.)

Code	Strain/species	c	$\Delta H_A^\ddagger$	ΔC_P	n	T_{mes}
189	*Mucor racemosus*	18.9	71347	57.95	498.5	291
190	*Mucor racemosus*	18.8	70973	57.78	340.9	291
191	*Mucor racemosus*	19.0	71736	57.09	302.8	290
193	*Mucor racemosus*	18.9	71792	59.30	409.0	293
195	*Mucor racemosus*	18.1	70803	58.74	429.2	292
415	*Aphis spiraecola*	13.3	66887	58.25	485.9	292
439	*Aphis gossypii*	17.0	75188	57.81	349.1	291
446	*Aphis gossypii*	12.1	62920	59.01	414.7	292
602	*Aphis gossypii*	16.5	74858	58.91	490.7	293
608	*Bemisia argentifolii*	11.7	64766	59.69	421.2	294
610	*Hyadaphis pseudobrassicae*	17.9	77380	57.27	427.9	290
665	*Rhopalosiphum rufiabdominalis*	17.5	75379	55.76	220.5	288
669	*Eriosoma lanigerum*	17.2	76578	55.44	236.0	287
670	*Diatraea lineolata*	13.0	70315	57.81	641.0	291
679	*Trichogrammatoidea bactrae*	16.1	66599	65.56	384.5	300
1392	*Methanococcoides alaskense*	17.2	68986	55.64	314.4	287
1457	*Pseudomonas fluorescens*	19.6	70648	51.01	107.4	280
1459	*Pseudomonas fluorescens*	19.6	71278	53.51	153.0	284
1460	*Pseudomonas fluorescens*	19.5	71339	54.13	140.4	285
1461	*Pseudomonas fluorescens*	19.4	70808	51.65	137.9	280
1462	*Pseudomonas fluorescens*	19.5	71429	54.31	139.3	286
161	*Acarus siro*	70.6	204131	44.43	162.2	265
660	*Sitobion miscanthi*	17.8	77766	56.57	624.6	289
162	*Aleuroglyphus ovatus*	54.2	165098	48.42	185.2	275
459	*Paronychiurus kimi*	17.8	75937	59.45	348.7	291
924	*Brochothrix thermosphacta*	20.5	73048	55.79	231.5	288
1399	*Bacillus sp.*	14.7	65315	54.37	229.9	286
1203	*Thalassiosira rotula*	16.9	68867	50.77	184.1	279
997	*Methanosarcina lacustris*	15.4	69844	51.87	153.3	281
81	*Cyanobacterial str.*	18.3	77535	49.85	147.0	276
1384	*Pseudomonas sp.*	17.3	66547	55.41	203.0	288
1205	*Thalassiosira rotula*	15.7	66415	50.30	140.5	278
644	*Sitophilus oryzae*	4.9	46560	58.33	255.0	292
645	*Sitophilus oryzae*	5.1	46867	59.07	209.3	292
676	*Sitophilus oryzae*	7.8	51473	59.14	538.2	293
677	*Sitophilus oryzae*	9.7	56101	59.77	449.2	294
678	*Sitophilus oryzae*	10.1	56747	62.59	395.5	297
1400	*Gram-negative str.*	17.2	71429	54.57	157.2	286
643	*Sitophilus oryzae*	1.9	39689	59.22	430.1	293
163	*Tyrophagus putrescentiae*	142.9	376388	25.25	130.8	182
461	*Liposcelis badia*	13.8	74662	59.90	776.9	294
627	*Macrolophus pygmaeus*	15.3	74783	58.25	443.5	292
628	*Macrolophus pygmaeus*	13.0	69555	60.74	536.5	295
148	*Pseudomonas sp.*	19.2	69346	56.13	194.9	289
210	*Synechococcus sp.*	19.5	80404	58.73	503.7	293
64	*Desulfovibrio litoralis*	11.5	57813	46.67	75.2	271
62	*Desulfovibrio cuneatus*	18.0	74307	46.09	84.6	268
150	*Xylella fastidiosa*	19.9	78828	61.79	541.7	296
63	*Desulfovibrio cuneatus*	15.6	68706	49.38	105.8	275
442	*Toxoptera aurantii*	13.0	65948	58.26	521.0	292
458	*Toxoptera citricida*	14.0	68774	59.31	549.5	292
1266	*Methanobacterium Bryantii*	15.7	70632	56.36	142.1	289
1451	*Chlamydomonas sp.*	16.5	74107	49.37	154.1	273
1382	*Arthrobacter sp.*	20.7	75232	53.98	177.9	285
1383	*Arthrobacter sp.*	16.4	65607	55.20	187.8	286
315	*Acidithiobacillus ferridurans*	23.6	87824	59.23	343.6	293
561	*Saccharomyces bayanus*	9.6	49928	58.76	293.7	293
562	*Saccharomyces bayanus*	7.7	45310	59.13	280.8	293
571	*Saccharomyces cerevisiae*	4.9	38726	62.10	303.2	297
578	*Saccharomyces mikatae*	-6.1	12136	62.48	327.7	298
580	*Saccharomyces paradoxus*	2.5	33378	61.47	299.6	296
581	*Saccharomyces paradoxus*	0.7	29199	61.94	308.3	297
1130	*Nannochloropsis oceanica*	20.7	81024	57.86	406.1	291
500	*Acidithiobacillus thiooxidans*	4.0	46560	61.44	695.6	296
1272	*Methanocorpusculum sinsese*	19.6	79443	55.96	135.4	288

(Table S1 continued.)

Code	Strain/species	c	$\Delta H_A^{\ddagger}$	ΔC_P	n	T_{mes}
410	*Plutella xylostella*	14.7	71466	58.95	429.4	293
1339	*Chimaereicella alkaliphila*	13.7	62317	59.51	198.4	293
634	*Oryzaephilus surinamensis*	6.7	52379	60.53	514.4	295
638	*Rhyzopertha dominica*	5.2	47343	56.24	178.2	288
503	*Spirillum sp.*	19.0	71893	51.30	92.9	280
1417	*Pseudomonas fluorescens*	19.6	72047	54.42	105.0	286
817	*Aspergillus candidus*	12.7	56874	59.81	316.9	294
1249	*Acaryochloris marina*	14.1	71001	66.63	377.3	300
1386	*Bacillus coagulans*	18.6	71626	60.18	319.9	295
1340	*Chimaereicella alkaliphila*	16.1	69852	64.15	346.8	299
149	*Xanthomonas campestris*	14.8	62856	57.71	180.6	291
411	*Haliea salexigens*	14.9	64782	57.13	247.4	290
1341	*Methanospirillum stamsii*	8.8	48290	62.05	260.0	297
687	*Balneola vulgaris*	18.1	74102	61.79	541.2	297
409	*Haliea rubra*	15.5	66371	60.99	192.5	295
341	*Tetranychus evansi*	14.3	70669	66.28	329.2	301
343	*Tetranychus evansi*	16.9	77002	65.03	325.1	299
416	*Iphiseius degenerans*	12.0	64758	59.01	301.1	293
417	*Elasmopalpus lignosellus*	14.3	73290	60.74	530.2	295
449	*Euseius finlandicus*	9.4	58782	65.16	399.7	300
450	*Tyrophagus putrescentiae*	15.5	72977	60.57	580.9	295
467	*Bemisia argentifolii*	20.3	86838	60.29	349.0	294
626	*Dactylopius austrinus*	31.6	115960	60.49	466.7	295
631	*Oryzaephilus surinamensis*	7.5	54441	60.37	526.1	295
761	*Erwinia amylovora*	19.3	72614	58.83	319.2	293
901	*Bacillus cereus*	21.4	76185	60.36	262.7	295
1479	*Pseudomonas sp.*	17.1	74821	58.34	157.3	292
1599	*Alkalibacterium iburiense*	17.4	72355	61.73	206.0	295
1600	*Alkalibacterium iburiense*	18.3	75451	64.00	300.8	298
785	*Thalassobaculum salexigens*	13.0	60003	58.57	244.1	292
1036	*Trichococcus patagoniensis*	12.0	58490	58.11	157.9	291
1387	*Bacillus coagulans*	14.7	60355	53.12	165.7	283
432	*Urolepis rufipes*	24.1	94783	61.68	348.1	296
381	*Acidithiobacillus ferrivorans*	20.7	79045	55.85	156.0	288
1102	*Nisaea denitrificans*	14.8	63478	58.66	155.7	292
768	*Melitea salexigens*	15.7	70029	61.03	358.6	296
430	*Muscidifurax raptor*	22.0	89754	58.77	308.3	293
77	*Marinobacter alkaliphilus*	6.3	39575	60.44	207.3	295
1033	*Spirochaeta africana*	19.8	80717	59.52	153.3	293
1415	*Chlorella vulgaris*	9.6	54974	44.40	50.1	265
1335	*Microcella putealis*	10.6	54041	54.67	155.9	277
954	*Sulfurimonas paralvinellae*	17.6	74189	57.29	232.7	290
431	*Muscidifurax raptor*	25.6	98408	58.27	297.2	292
1353	*Thiobacillus thioparus*	8.5	53185	62.46	442.4	297
519	*Pseudomonas fluorescens*	15.4	60368	56.93	185.7	290
504	*Aeromonas hydrophila*	17.7	66262	58.76	181.2	293
1297	*Methanosarcina semesiae*	0.9	30715	62.43	272.5	296
499	*Acidithiobacillus ferrooxidans*	10.9	63991	40.37	54.2	252
516	*Halorubrum lacusprofundi*	17.1	76447	59.57	249.7	294
520	*Pseudomonas putida*	19.7	70077	55.61	152.9	288
69	*Kluyveromyces battae*	16.1	66401	59.54	455.6	294
65	*Candida sphaerica*	15.2	71615	57.73	338.7	291
68	*Candia sphaerica*	17.6	69825	58.10	360.9	292
66	*Kluyveromyces blattae*	12.5	65369	59.51	411.1	294
617	*Chlorella pyrenoidosa*	23.2	86606	62.80	203.8	297
143	*Pseudomonas fluorescens*	17.0	65459	56.64	125.1	289
1096	*Thioreductor micantisoli*	12.3	61541	61.23	414.4	296
263	*Saccharomyces rouxii*	8.0	46018	61.09	314.1	296
636	*Oryzaephilus surinamensis*	5.3	49093	61.08	819.5	296
637	*Oryzaephilus surinamensis*	7.4	54168	61.14	710.0	296
434	*Clostridiurn frigidicarnis*	26.2	89360	59.14	164.8	293
632	*Oryzaephilus surinamensis*	10.5	61507	61.41	421.4	296
633	*Oryzaephilus surinamensis*	9.8	59849	61.64	531.8	296
635	*Oryzaephilus surinamensis*	4.9	48182	61.07	723.1	296
641	*Rhyzopertha dominica*	1.5	39482	62.28	727.0	297

(Table S1 continued.)

Code	Strain/species	c	$\Delta H_A^{\ddagger}$	ΔC_P	n	T_{mes}
640	*Rhyzopertha dominica*	0.8	37787	62.17	666.3	297
1274	*Thiobacillus prosperus*	21.5	84891	63.56	287.2	298
32	*Lactococcus paracasei*	20.8	79193	60.23	178.5	295
1286	*Acinetobacter calcoaceticus*	9.7	48395	61.74	298.9	297
1296	*Methanosarcina semesiae*	17.0	73840	60.46	270.7	295
1423	*Aeromonas shigelloides*	15.3	71348	57.72	126.1	291
419	*Amblyseius womersleyi*	24.3	95949	60.73	252.4	295
508	*Paracoccus halodenitrificans*	17.3	69545	60.01	153.2	294
510	*Paracoccus halodenitrificans*	20.1	80908	59.76	290.4	294
560	*Saccharomyces arboricolus*	7.7	45498	59.85	314.0	294
563	*Saccharomyces cariocanus*	-1.1	24218	61.58	372.9	296
566	*Saccharomyces cerevisiae*	0.5	29232	63.11	309.2	298
567	*Saccharomyces cerevisiae*	2.5	33510	62.85	341.7	298
569	*Saccharomyces cerevisiae*	3.1	34952	62.89	344.9	298
570	*Saccharomyces cerevisiae*	0.2	27578	63.19	292.9	298
573	*Saccharomyces cerevisiae*	0.3	27603	63.06	233.3	298
579	*Saccharomyces paradoxus*	1.4	30248	61.60	303.3	296
818	*Saccharomyces uvarum*	17.5	69954	56.26	114.5	288
528	*Listeria monocytogenes*	12.9	56742	62.97	261.2	298
639	*Rhyzopertha dominica*	1.1	38480	61.89	428.2	297
587	*Desulforhopalus species*	8.8	54509	61.17	317.9	296
527	*Listeria monocytogenes*	12.9	56722	62.84	256.0	298
199	*Serratia marcescens*	20.0	74322	61.96	421.9	297
1422	*Aeromonas shigelloides*	24.6	93837	62.17	190.3	297
1360	*Listeria monocytogenes*	24.7	84323	58.13	153.3	292
1603	*Alkalibacterium psychrotolerans*	11.1	50440	61.01	272.8	295
1542	*Sporomusa acidovorans*	-3.6	22238	68.62	362.3	303
647	*Tribolium castaneum*	3.2	44053	62.32	446.4	297
67	*Kluyveromyces thermotolerans*	11.8	63465	60.32	271.6	295
648	*Tribolium castaneum*	3.9	45514	62.52	504.6	298
530	*Listeria monocytogenes*	13.6	58226	61.92	218.1	297
70	*Kluyveromyces thermotolerans*	13.9	60747	60.46	274.9	295
1138	*Escherichia coli*	14.9	58984	60.11	196.3	294
1316	*Anabaena variabilis*	8.5	54052	67.96	409.4	302
517	*Halorubrum lacusprofundi*	19.3	81336	59.47	144.6	294
509	*Paracoccus halodenitrificans*	16.2	69690	60.25	250.6	295
413	*Salinisphaera hydrothermalis*	18.7	73812	63.41	236.0	297
646	*Tribolium castaneum*	0.3	36878	62.28	322.1	297
225	*Monascus ruber*	17.8	70673	65.84	251.9	301
418	*Scolothrips longicornis*	15.4	74254	63.89	440.4	299
497	*Leptospirillum ferrooxidans*	16.4	73147	63.01	508.6	298
511	*Paracoccus halodenitrificans*	14.8	64439	60.85	273.7	295
624	*Cryptolestes ferrugineus*	21.1	91044	61.36	335.1	296
668	*Callosobruchus maculatus*	20.7	87205	62.76	385.9	298
773	*Saccharomyces cerevisiae*	10.4	51201	61.19	368.4	296
848	*Methanobacterium flexile*	15.3	71070	60.21	166.7	295
849	*Methanobacterium movens*	9.1	56578	62.01	215.8	297
902	*Bacillus cereus*	15.2	61496	63.22	311.6	298
903	*Bacillus cereus*	15.4	61806	63.28	313.2	298
1554	*Galenea microaerophila*	21.9	81077	60.46	141.1	295
998	*Thiobacillus hydrothermalis*	16.7	68601	63.05	248.8	298
1003	*Anoxynatronum sibiricum*	0.3	32694	68.69	347.7	304
1140	*Salmonella enterica*	19.4	71120	61.62	377.9	296
1419	*Aeromonas hydrophila*	24.9	94666	62.30	204.8	297
1337	*Microcella putealis*	9.5	51406	63.71	320.6	299
1288	*Methanobacterium espanolae*	16.7	72587	48.03	82.3	270
1290	*Methanohalophilus oregonense*	14.3	67442	62.20	350.7	297
545	*Listeria monocytogenes*	14.6	60626	62.94	237.1	298
283	*Staphylococcus xylosus*	14.8	64669	62.32	436.4	297
1488	*Synechocystis sp.*	17.5	84770	63.69	271.3	298
514	*Halomonas elongata*	21.3	79466	61.95	188.0	297
852	*Vibrio alginolyticus*	18.9	70854	63.40	368.8	299
205	*Bacillus subtilis*	22.2	80387	62.37	187.6	297
999	*Amphibacillus fermentum*	20.5	79603	59.90	105.6	294
774	*Candida valida*	10.3	52756	62.50	494.0	298

(Table S1 continued.)

Code	Strain/species	c	$\Delta H_A^\ddagger$	ΔC_P	n	T_{mes}
1404	*Haloanaerobium alcaliphilum*	21.9	83428	61.98	144.5	296
287	*Staphylococcus xylosus*	16.6	64717	62.85	230.4	298
1388	*Bacillus laterosporus*	17.0	68221	59.46	251.5	294
1137	*Escherichia coli*	17.0	65275	62.18	279.6	297
72	*Methanofollis aquaemaris*	25.0	96065	66.15	247.3	300
285	*Staphylococcus xylosus*	20.9	77004	61.71	178.4	297
856	*Clostridium sp.*	12.7	57548	63.24	285.2	298
783	*Escherichia coli*	20.8	77564	63.78	322.5	299
407	*Escherichia coli*	18.7	74158	63.55	349.3	299
990	*Methanolobus bombayensis*	22.5	86302	61.52	210.3	296
137	*Methanococcus deltae*	17.5	73146	63.86	192.4	299
1035	*Spirochaeta asiatica*	14.4	68818	63.86	360.5	298
801	*Kluyveromyces marxianus*	12.2	56618	63.75	201.9	299
1493	*Synechocystis sp.*	12.4	71592	64.35	552.3	300
1273	*Thiobacillus prosperus*	9.4	55353	63.61	426.8	299
1034	*Spirochaeta alkalica*	31.5	112244	60.45	205.1	295
174	*Clostridium botulinum*	21.3	79146	65.44	402.1	301
175	*Clostridium botulinum*	22.0	80686	65.01	380.9	300
518	*Klebsiella oxytoca*	10.1	58221	63.70	236.4	299
564	*Saccharomyces cerevisiae*	-0.3	25710	63.24	302.4	298
565	*Saccharomyces cerevisiae*	6.1	42694	62.19	320.4	297
568	*Saccharomyces cerevisiae*	3.2	34751	62.30	339.4	297
572	*Saccharomyces cerevisiae*	-0.1	26850	63.60	280.7	299
898	*Bacillus cereus*	17.4	67078	63.69	277.0	299
904	*Bacillus cereus*	18.9	70260	61.81	261.5	297
908	*Listeria monocytogenes*	15.4	61965	60.67	124.8	295
1139	*Salmonella enterica*	24.3	83462	60.08	129.3	294
221	*Haloanaerobium praevalens*	25.5	92050	59.58	163.0	294
1413	*Methanocalculus taiwanensis*	25.9	98647	65.38	547.1	300
1267	*Methanobacterium veterum*	16.6	75558	63.01	245.8	298
1298	*Methanosarcina semesiae*	23.8	90406	62.76	273.0	298
1333	*Methanococcus maripaludis*	28.2	99876	61.22	219.7	296
282	*Staphylococcus xylosus*	11.2	59886	63.37	476.1	299
507	*Escherichia coli*	36.8	115207	53.76	92.9	285
529	*Listeria monocytogenes*	15.1	62033	62.37	280.9	297
6	*Escherichia coli*	15.4	62870	65.24	472.5	301
284	*Staphylococcus xylosus*	15.1	63943	63.08	410.7	298
515	*Halomonas elongata*	17.7	71289	62.51	194.5	298
58	*Clostridium termitidis*	9.7	56948	64.57	384.6	300
1295	*Bacillus halodenitrificans*	17.3	71387	64.25	515.7	300
946	*Klebsiella pneumoniae*	19.6	74039	62.72	421.4	298
25	*Desulfitobacterium dehalogenans*	21.8	84884	63.60	466.4	299
364	*Alkalithermophilic Bacteria*	13.1	60980	57.40	71.0	291
151	*Citrobacter intermedius*	17.8	74401	68.15	437.7	303
1000	*Amphibacillus tropicus*	23.3	89171	63.79	277.1	299
1246	*Bacillus beveridgei*	10.4	54988	61.32	179.0	296
286	*Staphylococcus xylosus*	13.7	57806	63.19	238.5	298
547	*Escherichia coli*	17.5	67162	64.21	338.8	300
748	*Sulfobacillus benefaciens*	19.7	80004	63.75	408.6	299
208	*Escherichia coli*	15.7	64671	64.59	344.2	300
513	*Halomonas elongata*	25.6	94893	60.53	152.8	295
198	*Pseudomonas fluorescens*	15.9	65473	64.03	449.2	299
944	*Escherichia coli*	14.5	61188	64.83	399.6	300
505	*Escherichia coli*	14.6	64098	63.98	279.7	299
506	*Escherichia coli*	15.3	64030	63.95	308.7	299
526	*Listeria monocytogenes*	11.8	54066	62.94	178.0	298
485	*Streptococcus thermophilus*	27.0	93437	63.68	259.5	299
141	*Escherichia coli*	28.8	96297	62.52	184.3	298
616	*Chlorella pyrenoidosa*	26.6	95357	64.02	442.0	299
1391	*Desulfovibrio capillatus*	23.4	86505	58.59	111.9	292
496	*Leptospirillum ferriphilum*	26.4	97008	62.72	233.8	298
550	*Escherichia coli*	17.0	66590	64.44	291.1	300
1072	*Methanoculleus marisnigri*	18.0	74676	61.93	139.9	297
1064	*Methanosarcina mazel*	20.0	82729	63.97	255.8	299
718	*Nautilia profundicola*	13.3	67869	66.13	191.7	300

(Table S1 continued.)

Code	Strain/species	c	$\Delta H_A^\ddagger$	ΔC_P	n	T_{mes}
480	*Streptococcus thermophilus*	16.4	65466	65.14	191.1	301
1327	*Magnetospirillum bellicus*	21.7	79407	54.81	105.0	284
1069	*Methanobacterium kanagiense*	25.9	100192	64.39	222.4	300
1031	*Alkaliphilus transvaalensis*	25.4	91213	64.42	266.0	300
76	*Methanoculleus palmolei*	15.6	72178	61.87	147.9	295
44	*Clostridium perfringens*	24.2	85214	64.91	168.2	300
142	*Pseudomonas aeruginosa*	16.9	66807	63.73	269.8	299
365	*Alkalithermophilic Bacteria*	15.0	66772	60.15	88.6	294
558	*Kluyveromyces marxianus*	4.2	38016	64.91	168.0	300
701	*Methanobacterium ferruginis*	14.8	71952	66.29	341.6	301
899	*Bacillus cereus*	19.1	70611	62.80	175.3	298
900	*Bacillus cereus*	19.1	70677	62.79	174.4	298
907	*Listeria monocytogenes*	18.4	69079	58.07	91.5	291
1268	*Thiomicrospira thermophila*	18.2	70656	60.38	168.7	295
909	*Escherichia coli*	20.0	73308	62.89	167.7	298
915	*Escherichia coli*	21.4	76820	64.02	222.7	299
1405	*Haloanaerobium lacusroseus*	15.2	68448	63.87	201.0	299
766	*Methanobacterium subterraneum*	13.9	61776	50.75	69.4	279
1487	*Synechocystis sp.*	14.4	76442	65.00	550.6	300
369	*Alkalithermophilic Bacteria*	7.3	48830	62.89	94.9	298
910	*Escherichia coli*	16.1	64519	64.56	372.0	300
911	*Escherichia coli*	28.7	94291	60.39	164.2	293
912	*Escherichia coli*	20.9	76051	63.56	400.9	299
913	*Escherichia coli*	18.7	70584	63.95	349.9	299
914	*Escherichia coli*	18.7	70665	63.88	362.4	299
916	*Escherichia coli*	18.6	70437	63.89	261.2	299
978	*Methanosarcina acetivorans*	21.3	85382	63.62	254.5	299
484	*Streptococcus thermophilus*	24.4	85882	63.54	254.4	299
1312	*Sulfobacillus thermotolerans*	17.3	72079	62.29	99.9	297
362	*Alkalithermophilic Bacteria*	-1.0	26495	64.55	76.5	300
549	*Escherichia coli*	15.9	63714	64.79	288.3	300
1061	*Methanosarcina barkeri*	11.9	63811	64.40	356.8	300
471	*Sporanaerobacter acetigenes*	6.5	43651	63.18	222.1	298
1047	*Vibrio diabolicus*	12.9	56143	63.73	208.8	299
866	*Porphyrobacter cryptus*	11.6	58052	66.33	288.3	302
1258	*Pseudomonas thermotolerans*	17.2	70638	65.37	313.0	301
204	*Bacillus megaterium*	21.8	78676	61.84	137.9	297
488	*Streptococcus thermophilus*	37.6	120490	63.76	316.9	299
1494	*Synechocystis sp.*	15.4	79546	64.00	803.7	299
74	*Halomonas mongoliensis*	17.2	72927	63.41	275.9	299
830	*Isosphaera pallida*	21.3	90244	67.69	242.6	303
487	*Streptococcus thermophilus*	27.9	95504	64.03	327.6	299
945	*Klebsiella pneumoniae*	13.2	57359	64.48	358.7	300
406	*Haloanaerobiurn salsugo*	19.5	80659	62.64	142.7	297
1071	*Methanoculleus marisnigri*	17.8	75268	65.03	179.3	301
481	*Streptococcus thermophilus*	22.7	82900	64.62	320.9	300
73	*Halomonas kenyensis*	16.9	71277	57.08	96.8	290
546	*Escherichia coli*	19.1	71794	64.54	287.6	300
1021	*Clostridium perfringens*	20.4	75078	66.18	299.9	302
1022	*Clostridium perfringens*	19.5	71392	66.85	236.3	302
1023	*Clostridium perfringens*	24.0	83475	66.98	247.7	302
555	*Escherichia coli*	17.1	66649	64.39	310.7	300
1315	*Anacystis nidulans*	21.0	84149	63.91	286.9	299
551	*Escherichia coli*	18.5	69098	63.82	263.7	299
552	*Escherichia coli*	15.9	63796	64.53	296.6	300
152	*Sporohalobacter marismortui*	16.7	67327	65.23	332.7	301
479	*Streptococcus thermophilus*	32.1	105665	63.49	236.7	299
1062	*Methanosarcina mazel*	16.6	77980	64.45	267.3	299
1059	*Methanosarcina barkeri*	17.2	82640	64.98	439.7	300
59	*Methanolobus zinderi*	17.6	76754	62.37	112.6	296
1489	*Synechocystis sp.*	13.7	75501	64.61	635.6	300
1063	*Methanosarcina mazel*	20.0	80958	64.21	267.5	299
1086	*Lactobacillus thermotolerans*	21.4	81043	64.83	276.0	300
1402	*Dethiosulfovibrio peptidovorans*	23.0	87463	66.81	241.1	302
1060	*Methanosarcina barkeri*	29.3	106818	64.28	322.5	300

(Table S1 continued.)

Code	Strain/species	c	$\Delta H_A^{\ddagger}$	ΔC_P	n	T_{mes}
483	*Streptococcus thermophilus*	28.5	97102	64.18	353.6	300
197	*Pseudomonas aeruginosa*	14.3	61237	63.94	416.9	299
1492	*Synechocystis sp.*	17.0	84358	65.20	795.8	301
1490	*Synechocystis sp.*	48.0	165261	64.80	813.4	300
493	*Ferroplasma acidiphilum*	17.7	82456	63.90	425.6	299
765	*Porphyrobacter tepidarius*	7.4	46508	66.74	419.3	302
43	*Clostridium perfringens*	20.5	77077	66.36	225.6	302
542	*Natronomonas bangense*	24.9	94502	66.33	215.4	302
543	*Natronomonas pharaonis*	28.6	103271	65.37	171.0	301
45	*Clostridium perfringens*	22.0	80184	65.29	209.9	301
853	*Clostridium perfringens*	32.3	103587	61.81	122.5	297
917	*Clostridium perfringens*	17.0	68477	66.59	303.4	302
554	*Escherichia coli*	20.1	74388	64.32	201.5	300
548	*Escherichia coli*	16.4	64814	64.75	309.1	300
553	*Escherichia coli*	19.1	71956	64.88	231.8	300
486	*Streptococcus thermophilus*	37.6	119244	61.56	155.1	296
154	*Sporohalobacter lortetii*	17.6	75850	64.47	283.2	300
918	*Clostridium perfringens*	20.9	79560	65.85	332.9	301
1097	*Methanococcus aeolicus*	16.9	70832	65.93	194.8	302
1074	*Rubrobacter radiotolerans*	9.8	56625	67.69	250.6	304
860	*Methanoculleus submarinus*	21.2	86060	65.11	277.0	301
366	*Alkalithermophilic Bacteria*	25.6	92934	57.38	71.5	290
532	*Halobaculum gomorrense*	19.7	79993	65.74	170.3	301
534	*Haloferax volcanii*	18.4	76867	66.49	412.3	302
536	*Halorubrum saccharovorum*	19.1	79211	65.89	224.4	302
538	*Natrialba asiatica*	19.7	80288	65.56	198.9	301
541	*Natronococcus occultus*	23.4	89569	64.36	115.0	300
776	*Synechococcus clone*	27.9	102786	63.11	117.1	298
777	*Synechococcus clone*	29.1	105217	61.18	95.8	296
779	*Synechococcus clone*	24.1	94657	65.87	163.3	301
780	*Synechococcus clone*	26.7	101151	66.01	143.8	301
1020	*Clostridium perfringens*	21.4	77549	66.68	414.2	302
1429	*Cyanidium caldarium*	22.2	89425	61.63	134.0	296
1146	*Geotoga subterranea*	36.2	126154	61.56	122.6	296
367	*Alkalithermophilic Bacteria*	19.2	79654	60.35	76.9	295
842	*Halomicronema excentricum*	10.3	62685	61.99	93.4	296
1323	*Haloanaerobacter chitinovorans*	15.8	69104	61.06	146.8	292
840	*Thiobacillus caldus*	9.6	54264	67.33	305.0	303
1259	*Thermomonas haemolytica*	14.3	61878	65.65	140.5	301
1055	*Methanosarcina barkeri*	12.2	67276	65.93	232.6	302
482	*Streptococcus thermophilus*	41.6	129646	61.29	140.5	296
498	*Acidithiobacillus caldus*	17.8	80072	48.43	45.7	274
363	*Alkalithermophilic Bacteria*	11.7	59694	64.81	102.7	300
33	*Streptococcus thermophilus*	26.4	93438	66.74	239.0	302
991	*Halonatronum saccharophilum*	20.2	79981	61.49	79.4	296
815	*Chlorobium tepidum*	12.3	60222	66.74	467.1	302
531	*Haloarcula vallismortis*	16.2	71954	66.94	250.6	303
540	*Natronobacterium gregoryi*	21.4	84741	66.56	206.9	302
489	*Acidimicrobium ferrooxidans*	10.9	57847	68.77	288.0	305
3	*Geobacillus sp.*	20.9	81887	64.52	96.8	300
495	*Sulfobacillus thermosulfidooxidans*	14.2	67924	68.12	147.2	304
535	*Halogeometricum borinquense*	18.5	76878	67.87	129.1	304
1394	*Clostridium thermoalcaliphilum*	24.4	88910	68.30	458.4	304
947	*Coccobacillus sp.*	17.6	74684	69.33	186.4	305
1082	*Alicyclobacillus acidoterrestris*	19.7	78360	67.48	138.9	303
1145	*Geotoga petraea*	13.1	65526	53.16	69.3	275
378	*Flexistipes sinusarabici*	23.0	92797	67.07	261.9	303
1163	*Desulfotomaculum alkaliphilum*	16.7	79882	70.07	353.7	306
981	*Pseudoxanthomonas taiwanensi*	17.1	70522	63.22	79.9	298
280	*Sulfurivirga caldicuralii*	20.3	82603	69.25	156.4	302
281	*Sulfurivirga caldicuralii*	20.2	80688	66.69	118.9	298
1135	*Deinococcus geothermalis*	15.8	68376	68.20	231.3	304
1260	*Thermomonas hydrothermalis*	22.9	87074	68.21	183.3	304
368	*Alkalithermophilic Bacteria*	12.9	65909	68.92	178.0	305
1136	*Deinococcus murrayi*	10.0	52979	68.86	194.2	303

(Table S1 continued.)

Code	Strain/species	c	$\Delta H_A^{\ddagger}$	ΔC_P	n	T_{mes}
868	*Porphyrobacter cryptus*	6.1	44787	68.54	248.8	304
867	*Porphyrobacter cryptus*	8.1	50050	68.49	234.9	304
751	*Lebetimonas acidiphila*	20.1	80022	63.30	73.0	298
1307	*Thermus chliarophilus*	22.2	86887	68.59	150.7	304
854	*Clostridium isatidis*	20.0	79356	68.73	719.5	305
533	*Halococcus morrhuae*	16.5	72595	69.19	234.0	305
537	*Haloterrigena turkmenica*	14.2	66173	69.43	207.4	305
539	*Natrinema pellirubrum*	13.0	63925	69.79	236.6	306
203	*Bacillus coagulans*	19.5	77226	67.81	143.7	304
494	*Ferroplasma cyprexacervatum*	8.9	58460	71.50	318.9	307
375	*Heliobacterium modesticaldum*	18.1	77136	72.62	225.7	308
23	*Clostridium thermobutyricum*	30.6	103875	62.56	81.1	298
1579	*Methanobacterium thermoflexum*	24.9	97295	68.21	104.9	304
1085	*Thiobacter subterraneus*	23.2	89852	69.99	187.4	306
714	*Nautilia nitratireducens*	8.9	51077	69.83	166.6	306
737	*Tepidimonas ignava*	29.4	103145	65.60	103.1	301
1395	*Anaerobaculum mobile*	22.8	93359	72.60	397.4	308
771	*Hydrogenimonas thermophila*	19.5	81003	70.37	363.0	305
810	*Geobacillus stearothermophilus*	25.8	94777	68.54	69.9	304
1147	*Petrotoga miotherma*	22.3	93573	68.11	109.3	304
1427	*Sulfobacillus sibiricus*	15.2	69666	71.13	329.5	307
1401	*Thermoanaerobacter brockii*	23.4	89626	64.25	63.3	300
4	*Methanogenium thermophilicum*	21.3	87247	71.80	424.9	307
212	*Marinitoga okinawensis*	19.4	78748	72.70	189.4	308
17	*Bacterial str.*	24.3	94156	68.96	183.4	305
1075	*Rubrobacter taiwanensis*	22.6	88963	67.31	96.1	303
797	*Meiothermus cerberus*	21.1	85557	70.31	201.9	306
405	*Anaerobranca gottschalkii*	23.2	88789	69.94	199.6	306
1396	*Clostridium paradoxum*	19.1	79407	71.90	483.9	308
1397	*Clostridium paradoxum*	33.4	114342	67.98	132.3	304
1309	*Thermus silvanus*	23.5	93333	72.13	177.2	308
1560	*Anaerobranca horikoshii*	22.6	86995	71.28	256.3	307
1538	*Methanogenium frittonii*	18.5	76459	68.54	180.6	304
1552	*Methanogenium thermophilicum*	20.9	85905	68.10	97.7	303
809	*Thermacetogenium phaeum*	23.0	100313	73.40	521.7	309
1537	*Moorella gycerini*	28.8	109819	72.91	268.7	309
408	*Caloramator viterbensis*	16.2	74879	73.69	241.9	309
846	*Clostridium thermosulfurogenes*	26.3	100324	70.60	98.7	306
1562	*Rubrobacter xylanophilus*	18.7	82285	73.10	109.9	309
1398	*Deferribacter thermophilus*	24.4	92740	69.42	91.4	305
1556	*Thermonema rossianum*	16.8	71663	73.53	211.9	309
1535	*Thermomicrobium fosteri*	25.9	104154	73.90	252.5	310
1557	*Thermonema rossianum*	19.4	77946	71.42	142.2	307
1555	*Thermonema lapsum*	22.4	87466	72.15	203.5	308
334	*Caminibacter hydrogeniphilus*	23.4	92025	71.18	102.6	307
800	*Picrophilus oshimae*	22.5	95084	72.62	151.3	308
1587	*Desulfurobacterium crinifex*	22.4	90996	74.89	155.6	307
1308	*Thermus ruber*	21.0	84171	66.82	60.2	302
1076	*Rubrobacter taiwanensis*	22.5	87914	65.34	81.5	301
1077	*Rubrobacter xylanophilus*	20.4	82181	65.51	79.6	301
1578	*Methanobacterium defluvii*	28.9	108256	71.64	201.1	308
1080	*Thermoanaerobacter uzonensis*	35.1	121961	73.74	208.5	309
1424	*Alicyclobacillus sp.*	19.9	94823	74.58	475.1	310
1084	*Alicyclobacillus sp.*	24.8	94180	72.92	182.3	309
1264	*Hydrogenophilus hirschii*	23.0	90456	74.31	219.0	310
1176	*Thermoanaerobacterium aotearoense*	29.2	109456	73.20	235.1	309
1089	*Methanothermococcus okinawensis*	22.8	89817	75.25	105.0	311
1079	*Thermoanaerobacter sulfurigignens*	25.3	98439	72.95	81.0	309
716	*Thermoanaerobacter sulfurigignens*	73.6	221592	60.52	88.9	295
812	*Thermoanaerobacter kivui*	22.6	94414	76.82	205.4	312
1042	*Marinitoga piezophila*	26.0	97712	73.33	90.5	309
1081	*Alicyclobacillus acidocaldarius*	24.7	94172	74.08	105.2	310
953	*Bacillus caldotenax*	27.8	101828	75.13	129.4	311
1041	*Marinitoga piezophila*	23.1	88897	74.39	98.6	310
1569	*Hydrogenobacter hydrogenophilus*	24.2	93588	76.74	126.7	312

(Table S1 continued.)

Code	Strain/species	c	$\Delta H_A^{\ddagger}$	ΔC_P	n	T_{mes}
845	*Thermotoga lettingae*	22.0	93257	76.24	224.1	312
955	*Methanobacterium thermoautotrophicum*	22.5	91627	74.80	96.2	310
10	*Caldicellulosiruptor acetigenus*	21.7	94492	79.16	108.2	314
1574	*Methanococcus thermolithotrophic*	22.0	85166	66.12	58.2	302
1426	*Bacillus sp.*	25.7	99353	76.59	234.1	312
952	*Bacillus caldotenax*	25.2	93543	74.75	123.4	310
146	*Synechococcus lividus*	21.8	95602	75.99	249.7	311
811	*Geobacillus thermoleovorans*	23.4	89858	77.32	129.7	312
829	*Thermoanaerobacter subterraneus*	22.5	93951	76.32	105.6	312
806	*Alicyclobacillus acidocaldarius*	26.2	95529	69.37	60.1	305
805	*Alicyclobacillus acidocaldarius*	23.8	89840	71.67	82.7	307
1294	*Thermus sp.*	23.0	90781	69.97	48.6	306
1283	*Methanothermus fervidus*	22.1	91947	73.83	104.2	309
1325	*Firmicutes sp.*	23.1	94898	75.45	175.0	311
1291	*Thermus brockianus*	23.1	90470	70.48	52.3	306
1276	*Methanobacterium thermoaggregans*	22.5	95192	76.71	126.0	312
1565	*Thermobrachium celere*	23.6	90901	77.00	223.1	312
1245	*Thermobaculum terrenum*	21.6	94022	76.68	211.2	312
802	*Thermus thermophilus*	23.4	88628	72.23	60.2	308
1141	*Marinithermus hydrothermalis*	25.3	97875	78.21	555.7	313
711	*Sulfurihydrogenibium kristjanssonii*	22.0	89748	69.52	65.2	302
1463	*Clostridium thermohydrosulfuricum*	22.0	89582	76.87	130.9	312
720	*Thermosipho globiformans*	23.4	90768	77.43	158.3	313
490	*Acidianus brierleyi*	21.5	96205	80.10	182.4	315
206	*Geobacillus stearothermophilus*	24.7	92321	74.87	113.1	310
1409	*Thermoanaerobacter siderophilus*	21.9	93364	77.99	168.9	313
1536	*Desulfurobacterium thermolithotrophum*	28.3	109818	78.45	137.7	313
217	*Thermotoga caldifontis*	21.3	94559	78.92	105.3	314
841	*Thermodesulfobacterium commune*	21.9	93333	79.98	110.5	315
803	*Thermus aquaticus*	22.5	91437	78.90	99.5	314
1106	*Thermoanaerobacter ethanolicus*	22.5	89008	67.72	58.9	303
986	*Thermosipho melanesiensis*	22.5	90507	69.62	50.9	305
985	*Thermotoga subterranea*	21.6	93506	75.70	123.9	311
1142	*Fervidobacterium pennivorans*	21.6	92125	77.64	108.5	313
1287	*Ammonifex degensii*	22.1	93363	79.14	115.7	314
1406	*Thermotoga hypogea*	21.9	93692	77.64	68.5	313
1284	*Sulfolobus metallicus*	21.9	93642	71.05	70.6	305
332	*Thermus sp.*	22.6	91744	78.76	107.7	314
705	*Thermosulfidibacter takaii*	21.7	93601	75.32	96.8	310
1170	*Geobacillus sp.*	23.1	89305	75.38	91.2	311
1094	*Sulfurihydrogenibium yellowstonense*	21.9	92234	76.59	89.9	311
1262	*Sulfolobus hakonensis*	21.0	95150	77.83	111.8	313
838	*Persephonella guaymasensis*	21.6	94328	78.25	117.2	313
708	*Persephonella hydrogeniphil*	22.9	90092	76.73	136.9	312
1293	*Thermus scotoductus*	22.5	90100	71.05	45.9	307
1292	*Thermus igniterrae*	22.0	90720	78.31	99.4	313
1585	*Thermoanaerobium brockii*	22.7	93351	79.46	202.7	314
207	*Thermus aquaticus*	22.6	92503	75.86	70.7	311
492	*Sulfolobus metallicus*	21.8	99549	72.13	51.0	308
835	*Hydrogenobacter thermophilus*	22.1	92452	77.56	90.9	313
982	*Thermosipho japonicus*	22.4	91005	78.47	115.9	313
839	*Persephonella marina*	21.8	95363	80.86	193.4	315
478	*Thermaerobacter marianensis*	21.8	93658	59.88	40.4	292
729	*Thermosulfurimonas dismutans*	22.6	92001	79.47	82.6	314
814	*Caldicellulosiruptor changbaiensis*	22.0	93274	82.25	104.2	316
1090	*Thermodesulfobacterium hydrogeniphilum*	21.9	94830	81.08	270.4	315
709	*Methanotorris formicicus*	22.3	91414	81.98	194.7	316
1577	*Thermococcus stetteri*	22.1	92646	81.10	88.0	315
1088	*Thermovibrio ammonificans*	19.2	86553	84.86	213.5	318
1410	*Thermoanaerobacter yonseiensis*	22.2	93330	82.00	138.7	316
1174	*Metallosphaera prunae*	21.6	94473	82.88	126.4	316
710	*Sulfurihydrogenibium rodmanii*	22.0	92459	79.97	124.5	315
1160	*Caldicellulosiruptor owensensis*	21.2	94367	78.67	115.4	311
799	*Sulfolobus sp.*	21.2	95169	78.32	102.0	311
1091	*Thermocladium modestius*	21.0	95650	77.60	94.7	312

(Table S1 continued.)

Code	Strain/species	c	$\Delta H_A^{\ddagger}$	ΔC_P	n	T_{mes}
959	*Palaeococcus helgesonii*	21.9	92320	83.74	116.2	317
1087	*Hydrogenivirga caldilitoris*	21.6	92411	80.01	170.8	312
1175	*Metallosphaera sedula*	21.6	94679	79.52	81.7	314
837	*Thermoanaerobacter tengcongensis*	21.7	94681	81.73	203.6	316
1099	*Balnearium lithotrophicum*	21.8	91836	80.04	158.2	315
957	*Eubacteria sp.*	22.3	92743	83.70	92.2	317
983	*Hydrogenobacter subterraneus*	38.4	144094	84.29	308.6	318
11	*Caldicellulosiruptor bescii*	21.9	92669	82.66	106.7	317
951	*Dictyoglomus thermophilum*	21.7	93117	78.08	76.3	311
491	*Acidianus brierleyi*	15.0	84477	78.89	66.4	314
975	*Thermotoga naphthophila*	22.5	94193	83.16	100.4	317
976	*Thermotoga petrophila*	22.2	93257	82.81	81.3	317
1043	*Methanococcus vulcanius*	21.9	92862	84.82	153.7	318
1269	*Sulfolobus yangmingensis*	21.2	95877	85.68	85.7	319
703	*Thermococcus celericrescens*	22.5	90656	83.60	101.2	317
850	*Thermocrinis ruber*	21.8	94172	85.50	126.5	319
1046	*Thermococcus hydrothermalis*	8.6	56032	87.21	49.8	320
1553	*Archaeoglobus veneficus*	22.0	94036	85.76	107.2	319
1281	*Methanobacterium thermoautotrophicum*	21.4	95377	85.68	95.0	319
1582	*Thermococcus profundus*	22.1	93019	84.10	118.0	315
1265	*Stygiolobus azoricus*	21.2	95163	85.90	164.8	319
1024	*Archaebacterial str.*	19.3	87420	85.51	120.2	319
961	*Thermococcus barossii*	15.9	77676	82.92	168.6	312
962	*Thermococcus celer*	13.9	70193	88.52	73.9	320
979	*Palaeococcus ferrophilus*	21.9	92416	86.08	130.5	319
336	*Desulfurococcus strain*	22.3	92402	84.02	50.2	318
987	*Thermococcus alcaliphilus*	21.9	93289	80.62	61.7	315
476	*Thermococcus siculi*	21.6	93364	80.37	54.4	314
1070	*Methanococcus infernus*	21.9	93765	88.69	94.4	321
960	*Thermococcus waiotapuensis*	21.6	94196	88.62	98.4	320
1100	*Aeropyrum camini*	23.0	102069	92.26	123.4	322
1277	*Acidilobus aceticus*	18.6	88330	91.78	231.2	322
1030	*Thermosphaera aggregans*	22.2	98473	87.31	298.7	319
958	*Thermococcus chitonophagus*	21.5	95864	90.31	144.7	322
474	*Thermococcus peptonophilus*	22.4	91901	83.38	63.2	316
984	*Sulfurisphaera ohwakuensis*	20.5	94969	86.12	74.8	319
1278	*Sulfolobus tengchongensis*	20.0	95703	90.12	113.1	321
831	*Thermoproteus uzoniensis*	13.1	71902	81.39	187.4	307
798	*Sulfolobus sp.*	18.6	92543	91.45	384.3	322
722	*Thermococcus nautili*	22.9	89782	61.56	26.6	295
956	*Archaebacterial str.*	14.7	74477	92.05	330.8	323
733	*Thermococcus prieurii*	22.8	89125	58.41	25.0	291
707	*Thermococcus gammatolerans*	21.4	95412	89.64	86.7	321
337	*Desulfurococcus strain*	13.3	68048	90.54	118.0	322
863	*Pyrobaculum calidifontis*	14.5	80137	89.96	153.5	311
1098	*Aeropyrum pernix*	30.7	126717	92.78	115.6	323
1261	*Pyrobaculum oguniense*	15.7	82910	99.20	196.1	326
1018	*Staphylothermus marinus*	22.7	103868	93.55	91.1	323
1280	*Stetteria hydrogenophila*	7.8	59864	98.51	215.1	326
335	*Pyrococcus glycovorans*	12.0	72194	96.87	120.7	325
828	*Pyrococcus abyssi*	21.5	94403	95.41	123.5	324
1319	*Pyrodictium abyssi*	35.5	139256	95.72	70.8	325
862	*Pyrobaculum aerophilum*	18.3	91291	101.38	379.2	327
966	*Pyrococcus furiosus*	13.9	72060	96.56	104.4	324
428	*Methanopyrus kandleri*	39.3	152994	98.56	66.8	326
770	*Archaeal str.*	27.3	118677	102.83	67.0	328
427	*Methanopyrus kandleri*	52.1	193102	101.15	73.2	327

	**Universal parameters**			
	$\Delta H^{\star}$	$\Delta S^{\star}$	$T_H^{\star}$	$T_S^{\star}$
Mean	4884	17.0	376	391
Lower 95% CI	4803	16.8	374	389
Upper 95% CI	4999	17.4	378	394
