# Supplementary material for: The Biokinetic Spectrum for Temperature
Source: PLoS One. 2016 Apr 18;11(4):e0153343. doi: 10.1371/journal.pone.0153343 (PMC4835062; doi:10.1371/journal.pone.0153343)
Supplement: S2 Table — Shown are the strain code, strain name, Aero. (aerobic status: A = aerobe, AN = anerobe, FA = facultative anaerobe, microA = microaerobe, U = unknown), Troph. (trophic status: A = autotroph, H = heterotroph, M = mixotroph, U = unknown), Smp. (size of data set), Tmin (minimum temperature for observed growth, C), Topt (temperature of maximal observed growth, C), Tmax (maximum temperature for observed growth, C), and Lit. (literature source). (PDF) [file pone.0153343.s003.pdf]

**Table S2. Sources of data.** Shown are the strain code, strain name, Aero. (aerobic status: A=aerobe, AN=anerobe, FA=Facultative anaerobe, microA=microaerobe, U=unknown), Troph. (trophic status: A=autotroph, H=heterotroph, M=mixotroph, U=unknown), Smp. (size of data set), $T_{\mathrm{min}}$ (minimum temperature for observed growth, C), $T_{\mathrm{opt}}$ (temperature of maximal observed growth, C), $T_{\mathrm{max}}$ (maximum temperature for observed growth, C), and Lit. (literature source).

| Code | Strain/species name | Aero. | Troph. | Smp. | $T_{\mathrm{min}}$ | $T_{\mathrm{opt}}$ | $T_{\mathrm{max}}$ | Lit. |
|---|---|---|---|---|---|---|---|---|
| 1 | *Vibrio natriegens* | FA | H | 1 | 37.0 | 37.0 | 37.0 | [1] |
| 2 | *Desulfotomaculum sp.* | AN | A | 1 | 65.0 | 65.0 | 65.0 | [2] |
| 3 | *Geobacillus sp.* | FA | H | 8 | 28.0 | 47.5 | 65.0 | [3] |
| 4 | *Methanogenium thermophilicum* | AN | H | 6 | 36.9 | 55.1 | 65.1 | [4] |
| 5 | *Tetreutreptia pomquetensis* | A | M | 4 | -0.0 | 5.0 | 10.0 | [5] |
| 6 | *Escherichia coli* | FA | H | 20 | 14.4 | 37.6 | 47.5 | [6] |
| 7 | *Caldicellulosiruptor hydrothermalis* | AN | H | 1 | 65.0 | 65.0 | 65.0 | [7] |
| 8 | *Caldicellulosiruptor kronotskyensis* | AN | H | 1 | 70.0 | 70.0 | 70.0 | [7] |
| 9 | *Caldicellulosiruptor kristjanssonii* | AN | H | 1 | 78.0 | 78.0 | 78.0 | [8] |
| 10 | *Caldicellulosiruptor acetigenus* | AN | H | 7 | 50.1 | 64.9 | 78.1 | [9] |
| 11 | *Caldicellulosiruptor bescii* | AN | H | 13 | 37.0 | 77.9 | 89.9 | [10] |
| 12 | *Caminibacter hydrogeniphilus* | AN | A | 1 | 60.0 | 60.0 | 60.0 | [11] |
| 13 | *Caminibacter mediatlanticus* | AN | A | 1 | 55.0 | 55.0 | 55.0 | [11] |
| 14 | *Caminibacter profundus* | FA | A | 1 | 55.0 | 55.0 | 55.0 | [11] |
| 15 | *Caminibacter profundus* | FA | A | 1 | 55.0 | 55.0 | 55.0 | [12] |
| 16 | *Noctiluca scintillans* | A | H | 4 | 12.0 | 16.0 | 27.0 | [13] |
| 17 | *Bacterial str.* | A | M | 21 | 34.0 | 55.2 | 60.1 | [14] |
| 18 | *Thermoanaerobaculum aquaticum* | AN | H | 1 | 60.0 | 60.0 | 60.0 | [15] |
| 19 | *Bacterial str.* | A | H | 5 | -9.4 | 5.0 | 5.0 | [16] |
| 20 | *Thermococcoides shengliensis* | AN | H | 1 | 65.0 | 65.0 | 65.0 | [17] |
| 21 | *Mesoaciditoga lauensis* | AN | H | 1 | 58.5 | 58.5 | 58.5 | [18] |
| 22 | *Clostridium thermobutyricum* | AN | H | 1 | 55.0 | 55.0 | 55.0 | [19] |

(Table S2 continued.)

| Code | Strain/species name | Aero. | Troph. | Smp. | $T_{\mathrm{min}}$ | $T_{\mathrm{opt}}$ | $T_{\mathrm{max}}$ | Lit. |
|---|---|---|---|---|---|---|---|---|
| 23 | *Clostridium thermobutyricum* | AN | H | 36 | 26.2 | 53.5 | 60.0 | [20] |
| 24 | *Vibrio sp.* | FA | H | 5 | 15.0 | 30.0 | 35.0 | [21] |
| 25 | *Desulfitobacterium dehalogenans* | AN | H | 15 | 17.1 | 38.1 | 44.9 | [22] |
| 26 | *Clostridium thermocellum* | AN | H | 2 | 60.0 | 60.0 | 60.0 | [23] |
| 27 | *Macrotrachela quadricornifra* | A | H | 3 | 16.0 | 24.0 | 24.0 | [24] |
| 28 | *Macrotrachela quadricornifra* | A | H | 3 | 16.0 | 24.0 | 24.0 | [24] |
| 29 | *Macrotrachela quadricornifra* | A | H | 3 | 16.0 | 24.0 | 24.0 | [24] |
| 30 | *Macrotrachela quadricornifra* | A | H | 3 | 16.0 | 24.0 | 24.0 | [24] |
| 31 | *Macrotrachela quadricornifra* | A | H | 3 | 16.0 | 24.0 | 24.0 | [24] |
| 32 | *Lactococcus paracasei* | FA | H | 35 | 5.9 | 32.9 | 39.9 | [25] |
| 33 | *Streptococcus thermophilus* | FA | H | 22 | 29.8 | 46.0 | 51.0 | [25] |
| 34 | *Clostridium perfringens* | AN | H | 11 | 19.0 | 49.0 | 49.0 | [26] |
| 35 | *Acroperus harpae* | A | H | 4 | 5.0 | 20.0 | 20.0 | [27] |
| 36 | *Alona affinis* | A | H | 4 | 5.0 | 20.0 | 20.0 | [27] |
| 37 | *Chydorus sphaericus* | A | H | 4 | 5.0 | 20.0 | 20.0 | [27] |
| 38 | *Eurycercus lamellatus* | A | H | 4 | 5.0 | 20.0 | 20.0 | [27] |
| 39 | *Graptoleberis testudinaria* | A | H | 4 | 5.0 | 20.0 | 20.0 | [27] |
| 40 | *Pleuroxus uncinatus* | A | H | 4 | 5.0 | 20.0 | 20.0 | [27] |
| 41 | *Sida crystallina* | A | H | 3 | 10.0 | 20.0 | 20.0 | [27] |
| 42 | *Simocephalus vetulus* | A | H | 3 | 10.0 | 20.0 | 20.0 | [27] |
| 43 | *Clostridium perfringens* | AN | H | 29 | 13.0 | 43.0 | 51.0 | [28] |
| 44 | *Clostridium perfringens* | AN | H | 28 | 13.0 | 40.0 | 51.0 | [28] |
| 45 | *Clostridium perfringens* | AN | H | 31 | 10.0 | 43.0 | 51.1 | [28] |
| 46 | *Desulfuromonas acetoxidans* | AN | H | 1 | 30.0 | 30.0 | 30.0 | [29] |
| 47 | *Sulfolobus acidocaldarius* | A | M | 1 | 70.0 | 70.0 | 70.0 | [30] |

(Table S2 continued.)

| Code | Strain/species name | Aero. | Troph. | Smp. | $T_{\mathrm{min}}$ | $T_{\mathrm{opt}}$ | $T_{\mathrm{max}}$ | Lit. |
|---|---|---|---|---|---|---|---|---|
| 48 | *Methanothermus sociabilis* | AN | A | 1 | 80.0 | 80.0 | 80.0 | [31] |
| 49 | *Methanothermus sociabilis* | AN | A | 1 | 88.0 | 88.0 | 88.0 | [31] |
| 50 | *Methanothermus sociabilis* | AN | A | 1 | 77.0 | 77.0 | 77.0 | [31] |
| 51 | *Archaeoglobus fulgidus* | AN | M | 1 | 76.0 | 76.0 | 76.0 | [32] |
| 52 | *Ceriodaphnia dubia* | A | H | 4 | 10.0 | 25.0 | 25.0 | [33] |
| 53 | *Plasmodium falciparum* | A | H | 1 | 37.0 | 37.0 | 37.0 | [34] |
| 54 | *Clostridium perfringens* | AN | H | 2 | 41.0 | 41.0 | 45.0 | [35] |
| 55 | *Clostridium perfringens* | AN | H | 1 | 45.0 | 45.0 | 45.0 | [35] |
| 56 | *Clostridium perfringens* | AN | H | 2 | 41.0 | 45.0 | 45.0 | [35] |
| 57 | *Clostridium perfringens* | AN | H | 2 | 41.0 | 45.0 | 45.0 | [35] |
| 58 | *Clostridium termitidis* | AN | H | 8 | 20.1 | 37.6 | 45.0 | [36] |
| 59 | *Methanolobus zinderi* | AN | H | 6 | 24.9 | 42.0 | 50.1 | [37] |
| 60 | *Paraphysomonas imperforata* | A | H | 4 | 14.0 | 26.0 | 26.0 | [38] |
| 61 | *Monas sp.* | A | H | 4 | 3.0 | 23.5 | 30.0 | [39] |
| 62 | *Desulfovibrio cuneatus* | FA | H | 6 | 4.0 | 27.9 | 32.9 | [40] |
| 63 | *Desulfovibrio cuneatus* | FA | H | 5 | 3.9 | 28.0 | 32.8 | [40] |
| 64 | *Desulfovibrio litoralis* | FA | H | 5 | 4.0 | 27.9 | 33.0 | [40] |
| 65 | *Candida sphaerica* | FA | H | 10 | 3.8 | 31.8 | 36.8 | [41] |
| 66 | *Kluyveromyces blattae* | FA | H | 9 | 7.8 | 31.9 | 37.1 | [41] |
| 67 | *Kluyveromyces thermotolerans* | FA | H | 11 | 3.7 | 34.5 | 40.7 | [41] |
| 68 | *Candia sphaerica* | FA | H | 10 | 3.8 | 31.8 | 37.0 | [41] |
| 69 | *Kluyveromyces battae* | FA | H | 10 | 7.9 | 31.7 | 40.6 | [41] |
| 70 | *Kluyveromyces thermotolerans* | FA | H | 11 | 3.5 | 34.7 | 40.8 | [41] |
| 71 | *Methanofollis aquaemaris* | AN | H | 4 | 19.9 | 36.9 | 36.9 | [42] |
| 72 | *Methanofollis aquaemaris* | AN | H | 6 | 19.9 | 36.6 | 42.6 | [42] |

(Table S2 continued.)

| Code | Strain/species name | Aero. | Troph. | Smp. | $T_{\mathrm{min}}$ | $T_{\mathrm{opt}}$ | $T_{\mathrm{max}}$ | Lit. |
|---|---|---|---|---|---|---|---|---|
| 73 | *Halomonas kenyensis* | FA | H | 8 | 11.9 | 40.9 | 48.6 | [43] |
| 74 | *Halomonas mongoliensis* | FA | H | 7 | 15.9 | 40.5 | 51.0 | [43] |
| 75 | *Methanoculleus chikugoensis* | AN | H | 5 | 15.1 | 25.3 | 40.2 | [44] |
| 76 | *Methanoculleus palmolei* | AN | H | 6 | 25.9 | 40.0 | 49.0 | [45] |
| 77 | *Marinobacter alkaliphilus* | FA | H | 7 | 10.6 | 30.1 | 45.1 | [46] |
| 78 | *Clostridium difficile* | AN | H | 1 | 37.0 | 37.0 | 37.0 | [47] |
| 79 | *Cyanobacterial str.* | A | A | 5 | 5.8 | 25.3 | 25.3 | [48] |
| 80 | *Cyanobacterial str.* | A | A | 5 | 4.4 | 19.9 | 25.1 | [48] |
| 81 | *Cyanobacterial str.* | A | A | 5 | 5.3 | 25.3 | 30.2 | [48] |
| 82 | *Cyanobacterial str.* | A | A | 5 | 4.9 | 14.7 | 25.3 | [48] |
| 83 | *Cyanobacterial str.* | A | A | 6 | 5.0 | 14.8 | 29.8 | [48] |
| 84 | *Cyanobacterial str.* | A | A | 6 | 4.3 | 24.3 | 29.9 | [48] |
| 85 | *Cyanobacterial str.* | A | A | 5 | 10.0 | 15.1 | 30.1 | [48] |
| 86 | *Cyanobacterial str.* | A | A | 5 | 10.9 | 16.2 | 30.8 | [48] |
| 87 | *Cyanobacterial str.* | A | A | 5 | 5.2 | 25.1 | 25.1 | [48] |
| 88 | *Cyanobacterial str.* | A | A | 5 | 4.3 | 19.1 | 24.5 | [48] |
| 89 | *Cyanobacterial str.* | A | A | 6 | 6.0 | 21.5 | 31.3 | [48] |
| 90 | *Cyanobacterial str.* | A | A | 4 | 15.0 | 19.9 | 29.9 | [48] |
| 91 | *Cyanobacterial str.* | A | A | 3 | 9.4 | 14.4 | 19.4 | [48] |
| 92 | *Cyanobacterial str.* | A | A | 6 | 4.7 | 14.5 | 29.9 | [48] |
| 93 | *Cyanobacterial str.* | A | A | 4 | 10.1 | 24.8 | 24.8 | [48] |
| 94 | *Cyanobacterial str.* | A | A | 5 | 10.2 | 15.5 | 35.4 | [48] |
| 95 | *Cyanobacterial str.* | A | A | 5 | 5.1 | 15.4 | 30.4 | [48] |
| 96 | *Cyanobacterial str.* | A | A | 5 | 5.0 | 14.7 | 24.7 | [48] |
| 97 | *Cyanobacterial str.* | A | A | 5 | 4.7 | 14.6 | 24.8 | [48] |

(Table S2 continued.)

| Code | Strain/species name | Aero. | Troph. | Smp. | $T_{min}$ | $T_{opt}$ | $T_{max}$ | Lit. |
|---|---|---|---|---|---|---|---|---|
| 98 | *Cyanobacterial str.* | A | A | 5 | 6.5 | 16.9 | 26.8 | [48] |
| 99 | *Cyanobacterial str.* | A | A | 4 | 9.6 | 14.0 | 24.6 | [48] |
| 100 | *Cyanobacterial str.* | A | A | 6 | 4.4 | 24.4 | 29.4 | [48] |
| 101 | *Cyanobacterial str.* | A | A | 4 | 9.5 | 19.7 | 24.9 | [48] |
| 102 | *Cyanobacterial str.* | A | A | 4 | 10.1 | 19.5 | 24.8 | [48] |
| 103 | *Hyadaphis foeniculi* | A | H | 4 | 15.0 | 28.0 | 28.0 | [49] |
| 104 | *Aspergillus nidulans* | A | H | 5 | 20.0 | 37.0 | 37.0 | [50] |
| 105 | *Aspergillus nidulans* | A | H | 4 | 20.0 | 37.0 | 37.0 | [50] |
| 106 | *Mucor hiemalis* | A | H | 2 | 25.0 | 25.0 | 25.0 | [50] |
| 107 | *Penicillium chrysogenum* | A | H | 2 | 25.0 | 25.0 | 25.0 | [50] |
| 108 | *Aspergillus nidulans* | A | H | 1 | 25.0 | 25.0 | 25.0 | [51] |
| 109 | *Geotrichum candidum* | A | H | 1 | 25.0 | 25.0 | 25.0 | [51] |
| 110 | *Mucor hiemalis* | A | H | 1 | 25.0 | 25.0 | 25.0 | [51] |
| 111 | *Neurospora crassa* | A | H | 2 | 25.0 | 37.0 | 37.0 | [51] |
| 112 | *Penecillum chrysogenum* | A | H | 1 | 25.0 | 25.0 | 25.0 | [51] |
| 113 | *Neurospora spora* | A | H | 1 | 25.0 | 25.0 | 25.0 | [52] |
| 114 | *Neurospora spora* | A | H | 1 | 25.0 | 25.0 | 25.0 | [52] |
| 115 | *Neurospora spora* | A | H | 1 | 25.0 | 25.0 | 25.0 | [52] |
| 116 | *Neurospora spora* | A | H | 1 | 25.0 | 25.0 | 25.0 | [52] |
| 117 | *Streptococcus pneumoniae* | A | H | 3 | 37.0 | 37.0 | 37.0 | [53] |
| 118 | *Streptococcus xylosus* | A | H | 1 | 37.0 | 37.0 | 37.0 | [54] |
| 119 | *Streptococcus xylosus* | A | H | 1 | 37.0 | 37.0 | 37.0 | [54] |
| 120 | *Streptococcus xylosus* | A | H | 1 | 37.0 | 37.0 | 37.0 | [54] |
| 121 | *Streptococcus xylosus* | A | H | 1 | 37.0 | 37.0 | 37.0 | [54] |
| 122 | *Streptococcus xylosus* | A | H | 1 | 37.0 | 37.0 | 37.0 | [54] |

(Table S2 continued.)

| Code | Strain/species name | Aero. | Troph. | Smp. | $T_{\mathrm{min}}$ | $T_{\mathrm{opt}}$ | $T_{\mathrm{max}}$ | Lit. |
|---|---|---|---|---|---|---|---|---|
| 123 | *Streptococcus xylosus* | A | H | 1 | 37.0 | 37.0 | 37.0 | [54] |
| 124 | *Streptococcus xylosus* | A | H | 1 | 37.0 | 37.0 | 37.0 | [54] |
| 125 | *Streptococcus xylosus* | A | H | 1 | 37.0 | 37.0 | 37.0 | [54] |
| 126 | *Streptococcus xylosus* | A | H | 1 | 37.0 | 37.0 | 37.0 | [54] |
| 127 | *Streptococcus xylosus* | A | H | 1 | 37.0 | 37.0 | 37.0 | [54] |
| 128 | *Streptococcus xylosus* | A | H | 1 | 37.0 | 37.0 | 37.0 | [54] |
| 129 | *Streptococcus xylosus* | A | H | 1 | 37.0 | 37.0 | 37.0 | [54] |
| 130 | *Streptococcus xylosus* | A | H | 1 | 37.0 | 37.0 | 37.0 | [54] |
| 131 | *Streptococcus xylosus* | A | H | 1 | 37.0 | 37.0 | 37.0 | [54] |
| 132 | *Streptococcus xylosus* | A | H | 1 | 37.0 | 37.0 | 37.0 | [54] |
| 133 | *Ammoniibacillus agariperforans* | A | H | 1 | 60.0 | 60.0 | 60.0 | [55] |
| 134 | *Thiohalobacter thiocyanaticus* | AN | A | 2 | 30.0 | 30.0 | 30.0 | [56] |
| 135 | *Allisonella histaminiformans* | AN | H | 1 | 39.0 | 39.0 | 39.0 | [57] |
| 136 | *Mus musculus* | A | H | 6 | 28.0 | 37.0 | 40.0 | [58] |
| 137 | *Methanococcus deltae* | AN | H | 5 | 25.3 | 36.9 | 56.3 | [59] |
| 138 | *Methanococcus olentangyi* | AN | H | 4 | 25.3 | 37.1 | 44.0 | [59] |
| 139 | *Halobacteroides halobius* | AN | H | 4 | 30.0 | 40.8 | 44.9 | [60] |
| 140 | *Halobacteroides halobius* | AN | H | 4 | 30.0 | 36.9 | 44.9 | [60] |
| 141 | *Escherichia coli* | FA | H | 20 | 8.0 | 39.3 | 46.0 | [61] |
| 142 | *Pseudomonas aeruginosa* | A | H | 10 | 8.0 | 40.0 | 45.0 | [61] |
| 143 | *Pseudomonas fluorescens* | A | H | 16 | 0.0 | 32.0 | 34.0 | [61] |
| 144 | *Psychrophilic pseudomonads* | A | H | 16 | 0.0 | 30.0 | 32.0 | [61] |
| 145 | *Psychrophilic pseudomonads* | A | H | 16 | 0.0 | 28.0 | 30.0 | [61] |
| 146 | *Synechococcus lividus* | A | A | 11 | 54.0 | 65.0 | 72.0 | [62] |
| 147 | *Escherichia coli* | FA | H | 17 | 4.1 | 38.7 | 38.7 | [63] |

(Table S2 continued.)

| Code | Strain/species name | Aero. | Troph. | Smp. | $T_{\mathrm{min}}$ | $T_{\mathrm{opt}}$ | $T_{\mathrm{max}}$ | Lit. |
|---|---|---|---|---|---|---|---|---|
| 148 | *Pseudomonas sp.* | A | H | 14 | 4.1 | 27.8 | 34.6 | [63] |
| 149 | *Xanthomonas campestris* | A | H | 6 | 9.9 | 29.9 | 34.9 | [64] |
| 150 | *Xylella fastidiosa* | A | H | 8 | 17.9 | 27.9 | 31.9 | [64] |
| 151 | *Citrobacter intermedius* | AN | H | 10 | 22.1 | 38.1 | 40.2 | [65] |
| 152 | *Sporohalobacter marismortui* | AN | H | 7 | 25.1 | 41.6 | 52.0 | [66] |
| 153 | *Halobacteroides acetoethylicus* | AN | H | 4 | 25.8 | 33.5 | 37.0 | [67] |
| 154 | *Sporohalobacter lortetii* | AN | H | 10 | 24.6 | 44.4 | 52.9 | [68] |
| 155 | *Haloincola saccharolytica* | AN | H | 1 | 37.0 | 37.0 | 37.0 | [69] |
| 156 | *Phaeocystis globosa* | A | A | 4 | 1.5 | 12.0 | 12.0 | [70] |
| 157 | *Phaeocystis pouchetii* | A | A | 6 | 6.0 | 14.1 | 14.1 | [70] |
| 158 | *Phaeocystis pouchetii* | A | A | 5 | 4.1 | 20.1 | 20.1 | [70] |
| 159 | *Phaeocystis pouchetii* | A | A | 5 | 6.1 | 14.1 | 18.0 | [70] |
| 160 | *Methanoplanus petrolearius* | AN | H | 1 | 37.0 | 37.0 | 37.0 | [71] |
| 161 | *Acarus siro* | A | H | 21 | 10.2 | 25.0 | 35.0 | [72] |
| 162 | *Aleuroglyphus ovatus* | A | H | 56 | 10.0 | 25.1 | 35.0 | [72] |
| 163 | *Tyrophagus putrescentiae* | A | H | 52 | 9.9 | 27.4 | 35.0 | [72] |
| 164 | *Methanoculleus receptaculi* | AN | H | 1 | 50.0 | 50.0 | 50.0 | [73] |
| 165 | *Butyribacterium methylotrophicum* | AN | H | 1 | 38.5 | 38.5 | 38.5 | [74] |
| 166 | *Methermicoccus shengliensis* | AN | H | 1 | 65.0 | 65.0 | 65.0 | [75] |
| 167 | *Methanoculleus oldenburgensis* | AN | H | 1 | 45.0 | 45.0 | 45.0 | [76] |
| 168 | *Diaphorina citri* | A | H | 5 | 15.0 | 28.0 | 30.0 | [77] |
| 169 | *Haloincola saccharolytica* | AN | H | 1 | 40.0 | 40.0 | 40.0 | [78] |
| 170 | *Halococcus salifodinae* | A | H | 1 | 37.0 | 37.0 | 37.0 | [79] |
| 171 | *Halothemothrix orenii* | AN | H | 1 | 60.0 | 60.0 | 60.0 | [80] |
| 172 | *Cellulomonas sp.* | A | H | 17 | 6.4 | 24.1 | 28.4 | [81] |

(Table S2 continued.)

| Code | Strain/species name | Aero. | Troph. | Smp. | $T_{\mathrm{min}}$ | $T_{\mathrm{opt}}$ | $T_{\mathrm{max}}$ | Lit. |
|---|---|---|---|---|---|---|---|---|
| 173 | *Salmonella typhimurium* | FA | H | 17 | 6.7 | 33.0 | 33.0 | [81] |
| 174 | *Clostridium botulinum* | AN | H | 9 | 12.5 | 37.0 | 45.0 | [82] |
| 175 | *Clostridium botulinum* | AN | H | 9 | 12.5 | 37.0 | 45.0 | [82] |
| 176 | *Desulfofaba gelida* | AN | A | 10 | -1.9 | 7.5 | 7.6 | [83] |
| 177 | *Desulfofrigus fragile* | AN | A | 18 | -1.9 | 18.0 | 23.5 | [83] |
| 178 | *Desulfofrigus marinus* | AN | A | 16 | -1.7 | 9.7 | 13.8 | [83] |
| 179 | *Desulfotalea arctica* | AN | A | 18 | -1.7 | 18.2 | 23.5 | [83] |
| 180 | *Desulfotalea psychrophila* | AN | A | 39 | -1.8 | 9.9 | 18.1 | [83] |
| 181 | *Mucor racemosus* | A | H | 5 | 10.0 | 25.0 | 30.0 | [84] |
| 182 | *Mucor racemosus* | A | H | 5 | 10.0 | 25.0 | 30.0 | [84] |
| 183 | *Mucor racemosus* | A | H | 5 | 10.0 | 25.0 | 30.0 | [84] |
| 184 | *Mucor racemosus* | A | H | 5 | 10.0 | 15.0 | 30.0 | [84] |
| 185 | *Mucor racemosus* | A | H | 5 | 10.0 | 25.0 | 30.0 | [84] |
| 186 | *Mucor racemosus* | A | H | 5 | 10.0 | 25.0 | 30.0 | [84] |
| 187 | *Mucor racemosus* | A | H | 5 | 10.0 | 25.0 | 30.0 | [84] |
| 188 | *Mucor racemosus* | A | H | 5 | 10.0 | 15.0 | 30.0 | [84] |
| 189 | *Mucor racemosus* | A | H | 5 | 10.0 | 25.0 | 30.0 | [84] |
| 190 | *Mucor racemosus* | A | H | 5 | 10.0 | 25.0 | 30.0 | [84] |
| 191 | *Mucor racemosus* | A | H | 5 | 10.0 | 25.0 | 30.0 | [84] |
| 192 | *Mucor racemosus* | A | H | 5 | 10.0 | 15.0 | 30.0 | [84] |
| 193 | *Mucor racemosus* | A | H | 5 | 10.0 | 25.0 | 30.0 | [84] |
| 194 | *Mucor racemosus* | A | H | 5 | 10.0 | 20.0 | 30.0 | [84] |
| 195 | *Mucor racemosus* | A | H | 5 | 10.0 | 25.0 | 30.0 | [84] |
| 196 | *Mucor racemosus* | A | H | 5 | 10.0 | 15.0 | 30.0 | [84] |
| 197 | *Pseudomonas aeruginosa* | A | H | 14 | 20.5 | 42.3 | 46.2 | [85] |

(Table S2 continued.)

| Code | Strain/species name | Aero. | Troph. | Smp. | $T_{\mathrm{min}}$ | $T_{\mathrm{opt}}$ | $T_{\mathrm{max}}$ | Lit. |
|---|---|---|---|---|---|---|---|---|
| 198 | *Pseudomonas fluorescens* | A | H | 15 | 19.3 | 38.7 | 45.3 | [85] |
| 199 | *Serratia marcescens* | FA | U | 10 | 22.0 | 33.3 | 41.0 | [85] |
| 200 | *Vibrio marinus* | FA | H | 6 | 7.3 | 13.7 | 18.6 | [85] |
| 201 | *Vibrio marinus* | FA | H | 8 | 9.9 | 23.1 | 23.1 | [85] |
| 202 | *Vibrio psychroerythrus* | FA | H | 5 | 6.0 | 13.9 | 13.9 | [85] |
| 203 | *Bacillus coagulans* | FA | H | 18 | 27.9 | 51.6 | 62.4 | [85] |
| 204 | *Bacillus megaterium* | FA | H | 14 | 20.7 | 40.4 | 46.5 | [85] |
| 205 | *Bacillus subtilis* | FA | H | 14 | 19.8 | 36.1 | 50.2 | [85] |
| 206 | *Geobacillus stearothermophilus* | FA | A | 14 | 45.3 | 68.4 | 70.7 | [85] |
| 207 | *Thermus aquaticus* | A | H | 14 | 45.5 | 70.8 | 76.7 | [85] |
| 208 | *Escherichia coli* | FA | H | 15 | 20.5 | 38.5 | 48.2 | [85] |
| 209 | *Prochlorococcus marinus* | A | A | 6 | 12.7 | 23.6 | 23.6 | [86] |
| 210 | *Synechococcus sp.* | A | A | 9 | 14.6 | 27.9 | 32.2 | [86] |
| 211 | *Thermomarinilinea lacunofontalis* | A | A | 6 | 37.1 | 50.2 | 60.2 | [87] |
| 212 | *Marinitoga okinawensis* | AN | H | 9 | 30.1 | 55.1 | 70.2 | [88] |
| 213 | *Oceanotoga teriensis* | AN | H | 1 | 56.5 | 56.5 | 56.5 | [89] |
| 214 | *Marinitoga hydrogenitolerans* | AN | H | 1 | 60.0 | 60.0 | 60.0 | [90] |
| 215 | *Marinitoga litoralis* | AN | H | 1 | 60.0 | 60.0 | 60.0 | [91] |
| 216 | *Pyrococcus yayanosii* | AN | H | 1 | 98.0 | 98.0 | 98.0 | [92] |
| 217 | *Thermotoga caldifontis* | AN | H | 8 | 54.8 | 69.5 | 84.8 | [93] |
| 218 | *Thermotoga profunda* | AN | H | 6 | 50.1 | 60.3 | 72.1 | [93] |
| 219 | *Kosmotoga olearia* | AN | H | 1 | 65.0 | 65.0 | 65.0 | [94] |
| 220 | *Kosmotoga olearia* | AN | H | 2 | 37.0 | 65.0 | 65.0 | [94] |
| 221 | *Haloanaerobium praevalens* | AN | H | 10 | 4.7 | 37.1 | 59.8 | [95] |
| 222 | *Mesotoga prima* | AN | H | 1 | 37.0 | 37.0 | 37.0 | [96] |

(Table S2 continued.)

| Code | Strain/species name | Aero. | Troph. | Smp. | $T_{\mathrm{min}}$ | $T_{\mathrm{opt}}$ | $T_{\mathrm{max}}$ | Lit. |
|---|---|---|---|---|---|---|---|---|
| 223 | *Marinitoga camini* | AN | H | 1 | 55.0 | 55.0 | 55.0 | [97] |
| 224 | *Pyrolobus fumarii* | FA | A | 7 | 96.2 | 105.6 | 111.7 | [98] |
| 225 | *Monascus ruber* | FA | H | 5 | 20.0 | 35.0 | 40.0 | [99] |
| 226 | *Monascus ruber* | FA | H | 5 | 20.0 | 35.0 | 40.0 | [99] |
| 227 | *Monascus ruber* | FA | H | 5 | 20.0 | 35.0 | 40.0 | [99] |
| 228 | *Monascus ruber* | FA | H | 4 | 25.0 | 35.0 | 40.0 | [99] |
| 229 | *Monascus ruber* | FA | H | 5 | 20.0 | 35.0 | 40.0 | [99] |
| 230 | *Monascus ruber* | FA | H | 5 | 20.0 | 40.0 | 40.0 | [99] |
| 231 | *Monascus ruber* | FA | H | 5 | 20.0 | 40.0 | 40.0 | [99] |
| 232 | *Monascus ruber* | FA | H | 4 | 25.0 | 35.0 | 40.0 | [99] |
| 233 | *Monascus ruber* | FA | H | 5 | 20.0 | 30.0 | 40.0 | [99] |
| 234 | *Monascus ruber* | FA | H | 5 | 20.0 | 35.0 | 40.0 | [99] |
| 235 | *Monascus ruber* | FA | H | 5 | 20.0 | 40.0 | 40.0 | [99] |
| 236 | *Monascus ruber* | FA | H | 4 | 25.0 | 35.0 | 40.0 | [99] |
| 237 | *Monascus ruber* | FA | H | 5 | 20.0 | 35.0 | 40.0 | [99] |
| 238 | *Monascus ruber* | FA | H | 5 | 20.0 | 40.0 | 40.0 | [99] |
| 239 | *Monascus ruber* | FA | H | 3 | 25.0 | 40.0 | 40.0 | [99] |
| 240 | *Monascus ruber* | FA | H | 4 | 25.0 | 35.0 | 40.0 | [99] |
| 241 | *Kosmotoga pacifica* | AN | H | 1 | 70.0 | 70.0 | 70.0 | [100] |
| 242 | *Geminicoccus roseus* | A | H | 1 | 32.5 | 32.5 | 32.5 | [101] |
| 243 | *Thermobacteroides leptospartum* | AN | H | 1 | 60.0 | 60.0 | 60.0 | [102] |
| 244 | *Thermoanaerobacterium aciditolerans* | AN | H | 1 | 60.0 | 60.0 | 60.0 | [103] |
| 245 | *Calditerricola satsumensis* | A | H | 1 | 78.0 | 78.0 | 78.0 | [104] |
| 246 | *Dechloromonas denitrificans* | FA | H | 1 | 30.0 | 30.0 | 30.0 | [105] |
| 247 | *Paenibacillus anaericanus* | FA | H | 1 | 32.5 | 32.5 | 32.5 | [105] |

(Table S2 continued.)

| Code | Strain/species name | Aero. | Troph. | Smp. | $T_{\mathrm{min}}$ | $T_{\mathrm{opt}}$ | $T_{\mathrm{max}}$ | Lit. |
|---|---|---|---|---|---|---|---|---|
| 248 | *Paenibacillus terrae* | FA | H | 1 | 35.0 | 35.0 | 35.0 | [105] |
| 249 | *Flavobacterium denitrificans* | FA | H | 1 | 25.0 | 25.0 | 25.0 | [105] |
| 250 | *Enterococcus faecalis* | FA | H | 2 | 25.0 | 37.0 | 37.0 | [106] |
| 251 | *Enterococcus faecalis* | FA | H | 2 | 25.0 | 37.0 | 37.0 | [106] |
| 252 | *Enterococcus faecalis* | FA | H | 2 | 25.0 | 37.0 | 37.0 | [106] |
| 253 | *Enterococcus faecalis* | FA | H | 2 | 25.0 | 37.0 | 37.0 | [106] |
| 254 | *Enterococcus faecium* | FA | H | 2 | 25.0 | 37.0 | 37.0 | [106] |
| 255 | *Enterococcus faecium* | FA | H | 2 | 25.0 | 37.0 | 37.0 | [106] |
| 256 | *Enterococcus faecium* | FA | H | 2 | 25.0 | 37.0 | 37.0 | [106] |
| 257 | *Enterococcus faecium* | FA | H | 2 | 25.0 | 37.0 | 37.0 | [106] |
| 258 | *Enterococcus sp.* | FA | H | 2 | 25.0 | 37.0 | 37.0 | [106] |
| 259 | *Enterococcus sp.* | FA | H | 2 | 25.0 | 37.0 | 37.0 | [106] |
| 260 | *Enterococcus sp.* | FA | H | 2 | 25.0 | 37.0 | 37.0 | [106] |
| 261 | *Enterococcus sp.* | FA | H | 2 | 25.0 | 37.0 | 37.0 | [106] |
| 262 | *Saccharomyces rouxii* | FA | H | 5 | 25.0 | 32.0 | 40.1 | [107] |
| 263 | *Saccharomyces rouxii* | FA | H | 6 | 22.0 | 32.1 | 40.1 | [107] |
| 264 | *Saccharomyces rouxii* | FA | H | 4 | 22.0 | 28.0 | 32.0 | [107] |
| 265 | *Clostridium tagluense* | AN | H | 6 | 3.9 | 15.1 | 27.8 | [108] |
| 266 | *Clostridium jejuense* | AN | H | 1 | 30.0 | 30.0 | 30.0 | [109] |
| 267 | *Caloramator boliviensis* | AN | H | 4 | 45.0 | 60.0 | 60.0 | [110] |
| 268 | *Magnetovibrio blakemorei* | microA | A | 1 | 27.0 | 27.0 | 27.0 | [111] |
| 269 | *Thiofaba tepidiphila* | AN | A | 1 | 45.0 | 45.0 | 45.0 | [112] |
| 270 | *Clydonella rosenfieldi* | A | H | 4 | 5.0 | 20.0 | 20.0 | [113] |
| 271 | *Platyamoeba sp.* | A | H | 4 | 5.0 | 20.0 | 20.0 | [113] |
| 272 | *Vannella caledonica* | A | H | 4 | 5.0 | 20.0 | 20.0 | [113] |

(Table S2 continued.)

| Code | Strain/species name | Aero. | Troph. | Smp. | $T_{\mathrm{min}}$ | $T_{\mathrm{opt}}$ | $T_{\mathrm{max}}$ | Lit. |
|---|---|---|---|---|---|---|---|---|
| 273 | *Vannella sp.* | A | H | 4 | 5.0 | 20.0 | 20.0 | [113] |
| 274 | *Vahlkampfia baltica* | A | H | 4 | 5.0 | 20.0 | 20.0 | [113] |
| 275 | *Vahlkampfia damariscottae* | A | H | 4 | 5.0 | 20.0 | 20.0 | [113] |
| 276 | *Dactylamoeba sp.* | A | H | 3 | 10.0 | 20.0 | 20.0 | [113] |
| 277 | *Paraflabellula reniformis* | A | H | 4 | 5.0 | 20.0 | 20.0 | [113] |
| 278 | *Rhizamoeba sp.* | A | H | 4 | 5.0 | 20.0 | 20.0 | [113] |
| 279 | *Stereomyxa ramosa* | A | H | 4 | 5.0 | 15.0 | 20.0 | [113] |
| 280 | *Sulfurivirga caldicuralii* | microA | A | 5 | 37.0 | 50.1 | 59.9 | [114] |
| 281 | *Sulfurivirga caldicuralii* | microA | A | 5 | 37.1 | 50.1 | 60.2 | [114] |
| 282 | *Staphylococcus xylosus* | FA | H | 10 | 19.6 | 37.2 | 42.8 | [115] |
| 283 | *Staphylococcus xylosus* | FA | H | 11 | 13.8 | 35.6 | 43.1 | [115] |
| 284 | *Staphylococcus xylosus* | FA | H | 21 | 8.9 | 37.6 | 43.0 | [115] |
| 285 | *Staphylococcus xylosus* | FA | H | 17 | 11.5 | 36.6 | 42.0 | [115] |
| 286 | *Staphylococcus xylosus* | FA | H | 18 | 11.8 | 38.4 | 42.1 | [115] |
| 287 | *Staphylococcus xylosus* | FA | H | 19 | 11.4 | 36.4 | 41.6 | [115] |
| 288 | *Archaebacterial str.* | U | U | 5 | 85.1 | 95.0 | 100.0 | [116] |
| 289 | *Archaebacterial str.* | U | U | 2 | 95.1 | 95.1 | 100.0 | [116] |
| 290 | *Archaebacterial str.* | U | U | 5 | 85.1 | 90.1 | 100.0 | [116] |
| 291 | *Clostridium drakei* | AN | A | 1 | 30.0 | 30.0 | 30.0 | [117] |
| 292 | *Gracilibacter thermotolerans* | AN | H | 1 | 42.0 | 42.0 | 42.0 | [118] |
| 293 | *Thermoanaerobacter pentosaceus* | AN | H | 1 | 70.0 | 70.0 | 70.0 | [119] |
| 294 | *Natranaerobius thermophilus* | AN | H | 1 | 53.0 | 53.0 | 53.0 | [120] |
| 295 | *Methanococus aecolicus* | AN | A | 1 | 30.0 | 30.0 | 30.0 | [121] |
| 296 | *Methanococus deltae* | AN | A | 1 | 30.0 | 30.0 | 30.0 | [121] |
| 297 | *Methanococus maripaludis* | AN | A | 1 | 30.0 | 30.0 | 30.0 | [121] |

(Table S2 continued.)

| Code | Strain/species name | Aero. | Troph. | Smp. | $T_{\mathrm{min}}$ | $T_{\mathrm{opt}}$ | $T_{\mathrm{max}}$ | Lit. |
|---|---|---|---|---|---|---|---|---|
| 298 | *Methanococus sp.(A1)* | AN | A | 1 | 30.0 | 30.0 | 30.0 | [121] |
| 299 | *Methanococus sp.(A4)* | AN | A | 1 | 30.0 | 30.0 | 30.0 | [121] |
| 300 | *Methanococus sp.(A5)* | AN | A | 1 | 30.0 | 30.0 | 30.0 | [121] |
| 301 | *Methanococus sp.(C10)* | AN | A | 1 | 30.0 | 30.0 | 30.0 | [121] |
| 302 | *Methanococus sp.(C11)* | AN | A | 1 | 30.0 | 30.0 | 30.0 | [121] |
| 303 | *Methanococus sp.(C12)* | AN | A | 1 | 30.0 | 30.0 | 30.0 | [121] |
| 304 | *Methanococus sp.(C13)* | AN | A | 1 | 30.0 | 30.0 | 30.0 | [121] |
| 305 | *Methanococus sp.(C14)* | AN | A | 1 | 30.0 | 30.0 | 30.0 | [121] |
| 306 | *Methanococus sp.(C5)* | AN | A | 1 | 30.0 | 30.0 | 30.0 | [121] |
| 307 | *Methanococus sp.(C6)* | AN | A | 1 | 30.0 | 30.0 | 30.0 | [121] |
| 308 | *Methanococus sp.(C7)* | AN | A | 1 | 30.0 | 30.0 | 30.0 | [121] |
| 309 | *Methanococus sp.(C8)* | AN | A | 1 | 30.0 | 30.0 | 30.0 | [121] |
| 310 | *Methanococus sp.(C9)* | AN | A | 1 | 30.0 | 30.0 | 30.0 | [121] |
| 311 | *Methanococus sp.(D1)* | AN | A | 1 | 30.0 | 30.0 | 30.0 | [121] |
| 312 | *Methanococus sp.(S1)* | AN | A | 1 | 30.0 | 30.0 | 30.0 | [121] |
| 313 | *Methanococus sp.(S2)* | AN | A | 1 | 30.0 | 30.0 | 30.0 | [121] |
| 314 | *Methanococus vannielii* | AN | A | 1 | 30.0 | 30.0 | 30.0 | [121] |
| 315 | *Acidithiobacillus ferridurans* | FA | A | 9 | 19.7 | 29.0 | 37.8 | [122] |
| 316 | *Clostridium mayombei* | AN | A | 1 | 30.0 | 30.0 | 30.0 | [123] |
| 317 | *Clostridium acidisoli* | AN | H | 1 | 30.0 | 30.0 | 30.0 | [124] |
| 318 | *Clostridium akagii* | AN | H | 1 | 25.0 | 25.0 | 25.0 | [124] |
| 319 | *Chaetomium cellulolyticum* | A | H | 1 | 37.0 | 37.0 | 37.0 | [125] |
| 320 | *Clostridium thermoaceticum* | AN | H | 1 | 58.0 | 58.0 | 58.0 | [126] |
| 321 | *Clostridium thermocellum* | AN | H | 1 | 45.0 | 45.0 | 45.0 | [127] |
| 322 | *Clostridium thermocellum* | AN | H | 1 | 45.0 | 45.0 | 45.0 | [127] |

(Table S2 continued.)

| Code | Strain/species name | Aero. | Troph. | Smp. | $T_{\mathrm{min}}$ | $T_{\mathrm{opt}}$ | $T_{\mathrm{max}}$ | Lit. |
|---|---|---|---|---|---|---|---|---|
| 323 | *Clostridium thermocellum* | AN | H | 1 | 45.0 | 45.0 | 45.0 | [127] |
| 324 | *Clostridium thermocellum* | AN | H | 1 | 45.0 | 45.0 | 45.0 | [127] |
| 325 | *Clostridium thermocellum* | AN | H | 1 | 45.0 | 45.0 | 45.0 | [127] |
| 326 | *Clostridium thermocellum* | AN | H | 1 | 45.0 | 45.0 | 45.0 | [127] |
| 327 | *Clostridium thermocellum* | AN | H | 1 | 45.0 | 45.0 | 45.0 | [127] |
| 328 | *Strain SF1* | AN | H | 5 | 20.0 | 37.1 | 40.1 | [128] |
| 329 | *Bacterial str.* | microA | H | 1 | 32.5 | 32.5 | 32.5 | [129] |
| 330 | *Geosporobacter subterraneus* | AN | H | 1 | 42.0 | 42.0 | 42.0 | [130] |
| 331 | *Bacillus sp.(LCB41)* | A | H | 3 | 45.0 | 45.0 | 60.0 | [131] |
| 332 | *Thermus sp.* | A | H | 5 | 60.0 | 70.0 | 80.0 | [132] |
| 333 | *Clostridium sp.* | AN | H | 1 | 37.0 | 37.0 | 37.0 | [133] |
| 334 | *Caminibacter hydrogeniphilus* | AN | A | 5 | 50.0 | 60.0 | 69.9 | [134] |
| 335 | *Pyrococcus glycovorans* | AN | H | 13 | 75.0 | 95.5 | 103.6 | [135] |
| 336 | *Desulfurococcus strain* | AN | H | 7 | 54.6 | 84.7 | 90.0 | [136] |
| 337 | *Desulfurococcus strain* | AN | H | 8 | 54.7 | 89.9 | 94.6 | [136] |
| 338 | *Caloranaerobacter azorensis* | AN | H | 5 | 45.0 | 64.7 | 64.7 | [137] |
| 339 | *Anaeromyxobacter dehalogenans* | microA | H | 1 | 30.0 | 30.0 | 30.0 | [138] |
| 340 | *Shewanella oneidensis* | FA | H | 2 | 3.0 | 22.0 | 22.0 | [139] |
| 341 | *Tetranychus evansi* | A | H | 5 | 15.0 | 30.0 | 35.0 | [140] |
| 342 | *Tetranychus evansi* | A | H | 5 | 15.0 | 35.0 | 35.0 | [140] |
| 343 | *Tetranychus evansi* | A | H | 5 | 15.0 | 30.0 | 35.0 | [140] |
| 344 | *Tetranychus evansi* | A | H | 5 | 15.0 | 35.0 | 35.0 | [140] |
| 345 | *Tetranychus evansi* | A | H | 5 | 15.0 | 35.0 | 35.0 | [140] |
| 346 | *Tetranychus evansi* | A | H | 5 | 15.0 | 35.0 | 35.0 | [140] |
| 347 | *Tetranychus evansi* | A | H | 5 | 15.0 | 35.0 | 35.0 | [140] |

(Table S2 continued.)

| Code | Strain/species name | Aero. | Troph. | Smp. | $T_{\mathrm{min}}$ | $T_{\mathrm{opt}}$ | $T_{\mathrm{max}}$ | Lit. |
|---|---|---|---|---|---|---|---|---|
| 348 | *Bacterial str.* | AN | H | 1 | 30.0 | 30.0 | 30.0 | [141] |
| 349 | *Spirochaeta perfilievii* | AN | H | 1 | 28.0 | 28.0 | 28.0 | [142] |
| 350 | *Spirochaeta americana* | AN | H | 1 | 37.0 | 37.0 | 37.0 | [143] |
| 351 | *Exilispira thermophila* | AN | H | 1 | 50.0 | 50.0 | 50.0 | [144] |
| 352 | *Spirochaeta cellobiosiphila* | FA | H | 1 | 37.0 | 37.0 | 37.0 | [145] |
| 353 | *Spirochaeta coccoides* | FA | H | 1 | 30.0 | 30.0 | 30.0 | [146] |
| 354 | *Spirochaeta coccoides* | FA | H | 1 | 30.0 | 30.0 | 30.0 | [146] |
| 355 | *Spirochaeta coccoides* | FA | H | 1 | 30.0 | 30.0 | 30.0 | [146] |
| 356 | *Spirochaeta coccoides* | FA | H | 1 | 30.0 | 30.0 | 30.0 | [146] |
| 357 | *Spirochaeta coccoides* | FA | H | 1 | 30.0 | 30.0 | 30.0 | [146] |
| 358 | *Spirochaeta coccoides* | FA | H | 1 | 30.0 | 30.0 | 30.0 | [146] |
| 359 | *Spirochaeta coccoides* | FA | H | 1 | 30.0 | 30.0 | 30.0 | [146] |
| 360 | *Spirochaeta coccoides* | FA | H | 1 | 30.0 | 30.0 | 30.0 | [146] |
| 361 | *Spirochaeta coccoides* | FA | H | 1 | 30.0 | 30.0 | 30.0 | [146] |
| 362 | *Alkalithermophilic Bacteria* | A | H | 7 | 30.0 | 40.1 | 58.0 | [147] |
| 363 | *Alkalithermophilic Bacteria* | A | H | 7 | 30.1 | 45.9 | 57.9 | [147] |
| 364 | *Alkalithermophilic Bacteria* | A | H | 7 | 30.0 | 38.1 | 58.0 | [147] |
| 365 | *Alkalithermophilic Bacteria* | A | H | 7 | 30.0 | 40.0 | 58.0 | [147] |
| 366 | *Alkalithermophilic Bacteria* | A | H | 6 | 30.0 | 45.0 | 55.0 | [147] |
| 367 | *Alkalithermophilic Bacteria* | A | H | 6 | 29.9 | 45.0 | 55.0 | [147] |
| 368 | *Alkalithermophilic Bacteria* | A | H | 5 | 35.0 | 50.1 | 55.1 | [147] |
| 369 | *Alkalithermophilic Bacteria* | A | H | 6 | 30.0 | 40.0 | 55.2 | [147] |
| 370 | *Pseudoalteromonas antarctica* | A | H | 11 | 5.0 | 17.5 | 30.0 | [148] |
| 371 | *Spirochaeta caldaria* | AN | H | 1 | 50.0 | 50.0 | 50.0 | [149] |
| 372 | *Acetonema longum* | AN | H | 1 | 31.5 | 31.5 | 31.5 | [150] |

(Table S2 continued.)

| Code | Strain/species name | Aero. | Troph. | Smp. | $T_{min}$ | $T_{opt}$ | $T_{max}$ | Lit. |
|---|---|---|---|---|---|---|---|---|
| 373 | *Clostridium grantii* | AN | H | 1 | 30.0 | 30.0 | 30.0 | [151] |
| 374 | *Heliobacterium mobilis* | AN | H | 4 | 23.0 | 40.1 | 40.1 | [152] |
| 375 | *Heliobacterium modesticaldum* | AN | H | 9 | 23.1 | 52.0 | 55.0 | [152] |
| 376 | *Bacillus tusciae* | A | A | 1 | 55.0 | 55.0 | 55.0 | [153] |
| 377 | *Eubacterium angustum* | AN | H | 1 | 37.0 | 37.0 | 37.0 | [154] |
| 378 | *Flexistipes sinusarabici* | AN | H | 7 | 34.1 | 50.0 | 52.0 | [155] |
| 379 | *Fervidobacterium nodosum* | AN | H | 1 | 67.5 | 67.5 | 67.5 | [156] |
| 380 | *Desulfobacterium autotrophicum* | AN | A | 1 | 26.0 | 26.0 | 26.0 | [157] |
| 381 | *Acidithiobacillus ferrivorans* | FA | A | 7 | 20.0 | 30.0 | 35.0 | [158] |
| 382 | *Clostridium alkalicellum* | AN | H | 1 | 37.5 | 37.5 | 37.5 | [159] |
| 383 | *Symbiobacterium toebii* | microA | H | 6 | 45.2 | 60.3 | 69.9 | [160] |
| 384 | *Clostridium magnum* | AN | H | 1 | 31.0 | 31.0 | 31.0 | [161] |
| 385 | *Artemia franciscana* | A | H | 2 | 24.0 | 24.0 | 30.0 | [162] |
| 386 | *Artemia franciscana* | A | H | 2 | 15.0 | 15.0 | 24.0 | [162] |
| 387 | *Artemia parthenogenetica* | A | H | 1 | 24.0 | 24.0 | 24.0 | [162] |
| 388 | *Artemia parthenogenetica* | A | H | 1 | 24.0 | 24.0 | 24.0 | [162] |
| 389 | *Artemia persimilis* | A | H | 2 | 24.0 | 24.0 | 30.0 | [162] |
| 390 | *Artemia persimilis* | A | H | 3 | 15.0 | 30.0 | 30.0 | [162] |
| 391 | *Artemia persimilis* | A | H | 1 | 15.0 | 15.0 | 15.0 | [162] |
| 392 | *Artemia salina* | A | H | 2 | 15.0 | 24.0 | 24.0 | [162] |
| 393 | *Artemia salina* | A | H | 2 | 15.0 | 24.0 | 24.0 | [162] |
| 394 | *Artemia sinica* | A | H | 2 | 24.0 | 30.0 | 30.0 | [162] |
| 395 | *Artemia sinica* | A | H | 1 | 24.0 | 24.0 | 24.0 | [162] |
| 396 | *Psychrobacter glacincola* | A | H | 13 | 0.5 | 14.4 | 21.1 | [163] |
| 397 | *Methanobacterium thermoautotrophicum* | AN | A | 7 | 40.0 | 70.3 | 75.0 | [164] |

(Table S2 continued.)

| Code | Strain/species name | Aero. | Troph. | Smp. | $T_{\mathrm{min}}$ | $T_{\mathrm{opt}}$ | $T_{\mathrm{max}}$ | Lit. |
|---|---|---|---|---|---|---|---|---|
| 398 | *Clostridium ultunense* | AN | H | 1 | 37.0 | 37.0 | 37.0 | [165] |
| 399 | *Clostridium ultunense* | AN | H | 1 | 37.0 | 37.0 | 37.0 | [165] |
| 400 | *Clostridium purinolyticum* | AN | H | 1 | 36.0 | 36.0 | 36.0 | [166] |
| 401 | *Caloramator coolhaasii* | AN | H | 1 | 52.5 | 52.5 | 52.5 | [167] |
| 402 | *Clostridium ljungdahlii* | AN | A | 1 | 37.0 | 37.0 | 37.0 | [168] |
| 403 | *Clostridium scatologenes* | AN | H | 1 | 27.5 | 27.5 | 27.5 | [169] |
| 404 | *Chrysiogenes arsenatis* | AN | H | 1 | 28.0 | 28.0 | 28.0 | [170] |
| 405 | *Anaerobranca gottschalkii* | AN | H | 8 | 29.9 | 55.3 | 64.9 | [171] |
| 406 | *Haloanaerobiurn salsugo* | AN | H | 6 | 22.4 | 40.7 | 50.7 | [172] |
| 407 | *Escherichia coli* | FA | H | 10 | 11.9 | 36.8 | 44.0 | [173] |
| 408 | *Caloramator viterbensis* | AN | H | 17 | 32.9 | 59.1 | 64.4 | [174] |
| 409 | *Haliea rubra* | A | H | 6 | 15.0 | 30.0 | 44.1 | [175] |
| 410 | *Plutella xylostella* | A | H | 10 | 8.0 | 29.7 | 32.9 | [176] |
| 411 | *Haliea salexigens* | A | H | 7 | 9.9 | 29.9 | 44.0 | [177] |
| 412 | *Balneola alkaliphila* | A | H | 6 | 14.9 | 25.0 | 44.0 | [178] |
| 413 | *Salinisphaera hydrothermalis* | A | M | 5 | 20.0 | 35.0 | 40.0 | [179] |
| 414 | *Trichogrammatoidea bactrae* | A | H | 5 | 17.0 | 29.0 | 29.0 | [180] |
| 415 | *Aphis spiraecola* | A | H | 7 | 10.0 | 25.0 | 32.0 | [181] |
| 416 | *Iphiseius degenerans* | A | H | 5 | 15.0 | 30.0 | 32.5 | [182] |
| 417 | *Elasmopalpus lignosellus* | A | H | 9 | 13.0 | 30.0 | 36.0 | [183] |
| 418 | *Scolothrips longicornis* | A | H | 6 | 15.0 | 35.0 | 37.0 | [184] |
| 419 | *Amblyseius womersleyi* | A | H | 8 | 16.0 | 33.0 | 38.0 | [185] |
| 420 | *Escherichia coli* | FA | H | 4 | 10.0 | 37.0 | 37.0 | [186] |
| 421 | *Salmonella Typhimurium* | FA | H | 4 | 10.0 | 37.0 | 37.0 | [186] |
| 422 | *Clavigralla shadabi* | A | H | 6 | 20.0 | 29.8 | 31.9 | [187] |

(Table S2 continued.)

| Code | Strain/species name | Aero. | Troph. | Smp. | $T_{\min}$ | $T_{\text{opt}}$ | $T_{\max}$ | Lit. |
|---|---|---|---|---|---|---|---|---|
| 423 | *Clavigralla tomentosicollis* | A | H | 6 | 20.1 | 30.0 | 35.9 | [187] |
| 424 | *Acyrthosiphon pisum* | A | H | 5 | 11.9 | 23.1 | 26.7 | [188] |
| 425 | *Acyrthosiphon pisum* | A | H | 5 | 11.9 | 19.6 | 26.7 | [188] |
| 426 | *Acetanaerobacterium elongatum* | AN | H | 1 | 37.0 | 37.0 | 37.0 | [189] |
| 427 | *Methanopyrus kandleri* | AN | A | 10 | 90.1 | 105.0 | 122.1 | [190] |
| 428 | *Methanopyrus kandleri* | AN | A | 10 | 85.1 | 100.1 | 115.2 | [190] |
| 429 | *Caminicella sporogenes* | AN | H | 1 | 57.5 | 57.5 | 57.5 | [191] |
| 430 | *Muscidifurax raptor* | A | H | 9 | 14.7 | 30.1 | 32.6 | [192] |
| 431 | *Muscidifurax raptor* | A | H | 10 | 14.7 | 30.1 | 32.6 | [192] |
| 432 | *Urolepis rufipes* | A | H | 8 | 15.0 | 30.0 | 35.0 | [193] |
| 433 | *Trichomalopsis sarcophagae* | A | H | 4 | 19.3 | 25.3 | 33.0 | [194] |
| 434 | *Clostridiurn frigidicarnis* | AN | H | 33 | 3.9 | 32.5 | 40.5 | [195] |
| 435 | *Hypoaspis miles* | A | H | 4 | 15.0 | 25.0 | 30.0 | [196] |
| 436 | *Propylea dissecta* | A | H | 5 | 20.0 | 27.0 | 35.0 | [197] |
| 437 | *Stethorus punctillum* | A | H | 6 | 16.0 | 30.0 | 32.0 | [198] |
| 438 | *Hyperaspis notata* | A | H | 5 | 18.0 | 30.0 | 32.0 | [199] |
| 439 | *Aphis gossypii* | A | H | 5 | 10.0 | 25.0 | 30.0 | [200] |
| 440 | *Aphis gossypii* | A | H | 5 | 10.0 | 30.0 | 30.0 | [201] |
| 441 | *Stethorus gilvifrons* | A | H | 6 | 15.0 | 35.0 | 35.0 | [202] |
| 442 | *Toxoptera aurantii* | A | H | 7 | 10.0 | 28.0 | 32.0 | [203] |
| 443 | *Nephus bisignatus* | A | H | 4 | 20.0 | 25.0 | 32.5 | [204] |
| 444 | *Nephus includens* | A | H | 4 | 20.0 | 30.0 | 32.5 | [204] |
| 445 | *Liposcelis entomophila* | A | H | 6 | 20.0 | 27.5 | 35.0 | [205] |
| 446 | *Aphis gossypii* | A | H | 8 | 15.0 | 25.0 | 32.5 | [206] |
| 447 | *Hypothenemus hampei* | A | H | 5 | 20.0 | 26.0 | 30.0 | [207] |

(Table S2 continued.)

| Code | Strain/species name | Aero. | Troph. | Smp. | $T_{\mathrm{min}}$ | $T_{\mathrm{opt}}$ | $T_{\mathrm{max}}$ | Lit. |
|---|---|---|---|---|---|---|---|---|
| 448 | *Tetranychus evansi* | A | H | 5 | 15.0 | 35.0 | 35.0 | [208] |
| 449 | *Euseius finlandicus* | A | H | 6 | 15.0 | 30.0 | 32.0 | [209] |
| 450 | *Tyrophagus putrescentiae* | A | H | 6 | 15.0 | 30.0 | 34.0 | [210] |
| 451 | *Bemisia tabaci* | A | H | 5 | 16.0 | 28.0 | 28.0 | [211] |
| 452 | *Frankliniella occidentalis* | A | H | 6 | 15.0 | 30.0 | 30.0 | [212] |
| 453 | *Aphis gossypii* | A | H | 5 | 10.0 | 25.0 | 30.0 | [213] |
| 454 | *Sitotroga cerealella* | A | H | 3 | 20.0 | 30.0 | 30.0 | [214] |
| 455 | *Sitotroga cerealella* | A | H | 3 | 20.0 | 30.0 | 30.0 | [214] |
| 456 | *Sphaerochaeta globosa* | AN | H | 1 | 27.5 | 27.5 | 27.5 | [215] |
| 457 | *Sphaerochaeta pleomorpha* | AN | H | 1 | 27.5 | 27.5 | 27.5 | [215] |
| 458 | *Toxoptera citricida* | A | H | 7 | 10.0 | 28.0 | 32.0 | [216] |
| 459 | *Paronychiurus kimi* | A | H | 5 | 17.0 | 25.1 | 28.0 | [217] |
| 460 | *Myzus persicae* | A | H | 5 | 10.0 | 20.0 | 30.0 | [218] |
| 461 | *Liposcelis badia* | A | H | 7 | 22.0 | 27.5 | 35.0 | [219] |
| 462 | *Aulacorthum solani* | A | H | 4 | 10.0 | 25.0 | 25.0 | [220] |
| 463 | *Aphis punicae* | A | H | 5 | 17.5 | 25.0 | 27.5 | [221] |
| 464 | *Liposcelis bostrychophila* | A | H | 7 | 20.0 | 30.0 | 35.0 | [222] |
| 465 | *Adoxophyes orana* | A | H | 6 | 14.0 | 25.0 | 25.0 | [223] |
| 466 | *Aphis nasturti* | A | H | 5 | 15.0 | 27.0 | 30.0 | [224] |
| 467 | *Bemisia argentifolii* | A | H | 5 | 20.0 | 30.0 | 35.0 | [225] |
| 468 | *Tetraneura nigriabdominalis* | A | H | 4 | 15.0 | 30.0 | 30.0 | [226] |
| 469 | *Liposcelis paeta* | A | H | 4 | 23.8 | 33.8 | 37.5 | [227] |
| 470 | *Neoseiulus baraki* | A | H | 4 | 25.0 | 30.0 | 33.0 | [228] |
| 471 | *Sporanaerobacter acetigenes* | AN | H | 7 | 25.2 | 40.1 | 49.9 | [229] |
| 472 | *Sporomusa silvacetica* | AN | H | 1 | 30.0 | 30.0 | 30.0 | [230] |

(Table S2 continued.)

| Code | Strain/species name | Aero. | Troph. | Smp. | $T_{\text{min}}$ | $T_{\text{opt}}$ | $T_{\text{max}}$ | Lit. |
|---|---|---|---|---|---|---|---|---|
| 473 | *Caloramator proteoclasticus* | AN | H | 1 | 55.0 | 55.0 | 55.0 | [231] |
| 474 | *Thermococcus peptonophilus* | AN | H | 5 | 70.0 | 85.2 | 100.0 | [232] |
| 475 | *Thermococcus sp.* | AN | H | 4 | 55.0 | 82.5 | 82.5 | [233] |
| 476 | *Thermococcus siculi* | AN | H | 5 | 70.0 | 84.8 | 89.9 | [234] |
| 477 | *Pyrococcus horikoshii* | AN | H | 4 | 85.0 | 98.0 | 100.0 | [235] |
| 478 | *Thermaerobacter marianensis* | A | H | 10 | 50.1 | 73.9 | 80.1 | [236] |
| 479 | *Streptococcus thermophilus* | FA | H | 16 | 21.3 | 41.8 | 45.8 | [237] |
| 480 | *Streptococcus thermophilus* | FA | H | 24 | 16.2 | 39.9 | 48.5 | [237] |
| 481 | *Streptococcus thermophilus* | FA | H | 20 | 22.3 | 40.9 | 45.7 | [237] |
| 482 | *Streptococcus thermophilus* | FA | H | 22 | 17.1 | 45.3 | 50.9 | [237] |
| 483 | *Streptococcus thermophilus* | FA | H | 18 | 18.6 | 42.2 | 46.3 | [237] |
| 484 | *Streptococcus thermophilus* | FA | H | 23 | 16.5 | 40.1 | 45.2 | [237] |
| 485 | *Streptococcus thermophilus* | FA | H | 18 | 24.1 | 39.1 | 46.2 | [237] |
| 486 | *Streptococcus thermophilus* | FA | H | 22 | 17.2 | 44.1 | 49.2 | [237] |
| 487 | *Streptococcus thermophilus* | FA | H | 15 | 23.5 | 40.6 | 44.3 | [237] |
| 488 | *Streptococcus thermophilus* | FA | H | 19 | 24.1 | 40.4 | 51.5 | [237] |
| 489 | *Acidimicrobium ferrooxidans* | A | M | 21 | 26.1 | 47.3 | 58.8 | [238] |
| 490 | *Acidianus brierleyi* | FA | M | 14 | 52.0 | 68.3 | 80.4 | [238] |
| 491 | *Acidianus brierleyi* | FA | M | 19 | 41.9 | 79.3 | 83.0 | [238] |
| 492 | *Sulfolobus metallicus* | A | A | 20 | 50.5 | 71.0 | 73.4 | [238] |
| 493 | *Ferroplasma acidiphilum* | A | A | 11 | 26.9 | 42.5 | 45.8 | [238] |
| 494 | *Ferroplasma cyprexacervatum* | A | A | 20 | 32.6 | 51.6 | 62.3 | [238] |
| 495 | *Sulfobacillus thermosulfidooxidans* | FA | M | 20 | 29.3 | 48.6 | 60.2 | [238] |
| 496 | *Leptospirillum ferriphilum* | A | H | 18 | 17.5 | 39.6 | 45.0 | [238] |
| 497 | *Leptospirillum ferrooxidans* | A | H | 16 | 18.0 | 35.0 | 44.0 | [238] |

(Table S2 continued.)

| Code | Strain/species name | Aero. | Troph. | Smp. | $T_{\mathrm{min}}$ | $T_{\mathrm{opt}}$ | $T_{\mathrm{max}}$ | Lit. |
|---|---|---|---|---|---|---|---|---|
| 498 | *Acidithiobacillus caldus* | FA | H | 18 | 25.8 | 45.9 | 51.1 | [238] |
| 499 | *Acidithiobacillus ferrooxidans* | FA | H | 17 | 11.0 | 31.0 | 38.0 | [238] |
| 500 | *Acidithiobacillus thiooxidans* | FA | H | 20 | 12.1 | 29.6 | 39.3 | [238] |
| 501 | *Methanococcoides burtonii* | AN | H | 21 | 5.6 | 22.5 | 28.4 | [239] |
| 502 | *Pseudomonas sp.* | A | H | 11 | -2.2 | 14.7 | 18.8 | [240] |
| 503 | *Spirillum sp.* | A | H | 14 | -2.4 | 29.8 | 33.6 | [240] |
| 504 | *Aeromonas hydrophila* | FA | H | 30 | 0.5 | 30.9 | 38.9 | [241] |
| 505 | *Escherichia coli* | FA | H | 10 | 17.8 | 39.0 | 43.0 | [242] |
| 506 | *Escherichia coli* | FA | H | 12 | 12.1 | 39.0 | 42.6 | [242] |
| 507 | *Escherichia coli* | FA | H | 12 | 13.2 | 37.2 | 45.1 | [242] |
| 508 | *Paracoccus halodenitrificans* | FA | A | 13 | 1.8 | 33.0 | 36.5 | [242] |
| 509 | *Paracoccus halodenitrificans* | FA | A | 13 | 4.0 | 34.9 | 38.3 | [242] |
| 510 | *Paracoccus halodenitrificans* | FA | A | 11 | 10.5 | 33.0 | 36.3 | [242] |
| 511 | *Paracoccus halodenitrificans* | FA | A | 13 | 4.0 | 35.0 | 38.3 | [242] |
| 512 | *Halomonas elongata* | FA | H | 13 | 12.3 | 39.5 | 39.5 | [242] |
| 513 | *Halomonas elongata* | FA | H | 11 | 18.5 | 38.7 | 42.2 | [242] |
| 514 | *Halomonas elongata* | FA | H | 14 | 9.7 | 36.0 | 39.4 | [242] |
| 515 | *Halomonas elongata* | FA | H | 14 | 9.0 | 37.6 | 41.5 | [242] |
| 516 | *Halorubrum lacusprofundi* | A | H | 26 | 5.5 | 31.5 | 40.2 | [243] |
| 517 | *Halorubrum lacusprofundi* | A | H | 25 | 5.2 | 34.8 | 40.0 | [243] |
| 518 | *Klebsiella oxytoca* | FA | H | 27 | 5.4 | 37.0 | 44.7 | [244] |
| 519 | *Pseudomonas fluorescens* | A | H | 52 | -0.6 | 30.4 | 35.4 | [245] |
| 520 | *Pseudomonas putida* | A | H | 85 | -0.4 | 31.7 | 39.2 | [245] |
| 521 | *Gelidibacter sp.* | A | H | 69 | 8.5 | 24.3 | 32.3 | [246] |
| 522 | *Glaciecola punicea* | A | H | 24 | 0.3 | 13.7 | 20.2 | [246] |

(Table S2 continued.)

| Code | Strain/species name | Aero. | Troph. | Smp. | $T_{\min}$ | $T_{\text{opt}}$ | $T_{\max}$ | Lit. |
|---|---|---|---|---|---|---|---|---|
| 523 | *Shewanella gelidimarina* | FA | H | 28 | -0.3 | 15.4 | 22.4 | [247] |
| 524 | *Shewanella gelidimarina* | FA | H | 26 | 0.4 | 16.3 | 20.9 | [247] |
| 525 | *Shewanella gelidimarina* | FA | H | 28 | -0.3 | 15.4 | 22.4 | [247] |
| 526 | *Listeria monocytogenes* | FA | H | 26 | 6.1 | 39.1 | 42.8 | [248] |
| 527 | *Listeria monocytogenes* | FA | H | 27 | 6.2 | 33.2 | 46.6 | [248] |
| 528 | *Listeria monocytogenes* | FA | H | 27 | 6.2 | 33.1 | 46.2 | [248] |
| 529 | *Listeria monocytogenes* | FA | H | 27 | 3.4 | 37.2 | 44.3 | [248] |
| 530 | *Listeria monocytogenes* | FA | H | 28 | 3.4 | 34.7 | 44.3 | [248] |
| 531 | *Haloarcula vallismortis* | A | H | 17 | 23.0 | 47.0 | 55.0 | [249] |
| 532 | *Halobaculum gomorrense* | A | H | 17 | 23.0 | 45.0 | 54.0 | [249] |
| 533 | *Halococcus morrhuae* | A | H | 18 | 23.0 | 51.0 | 57.0 | [249] |
| 534 | *Haloferax volcanii* | A | H | 14 | 23.0 | 45.0 | 49.0 | [249] |
| 535 | *Halogeometricum borinquense* | A | H | 19 | 23.0 | 49.0 | 58.0 | [249] |
| 536 | *Halorubrum saccharovorum* | A | H | 16 | 23.0 | 45.0 | 53.0 | [249] |
| 537 | *Haloterrigena turkmenica* | A | H | 18 | 23.0 | 51.0 | 57.0 | [249] |
| 538 | *Natrialba asiatica* | A | H | 15 | 23.0 | 45.0 | 51.0 | [249] |
| 539 | *Natrinema pellirubrum* | A | H | 18 | 23.0 | 51.0 | 57.0 | [249] |
| 540 | *Natronobacterium gregoryi* | A | H | 16 | 23.0 | 47.0 | 52.0 | [249] |
| 541 | *Natronococcus occultus* | A | H | 17 | 23.0 | 45.0 | 54.0 | [249] |
| 542 | *Natronomonas bangense* | A | H | 15 | 23.0 | 43.0 | 50.0 | [249] |
| 543 | *Natronomonas pharaonis* | A | H | 17 | 23.0 | 43.0 | 56.0 | [249] |
| 544 | *Halobacterium salinarum* | A | A | 15 | 23.1 | 49.1 | 50.2 | [249] |
| 545 | *Listeria monocytogenes* | FA | H | 28 | 8.4 | 35.4 | 38.8 | [250] |
| 546 | *Escherichia coli* | FA | H | 27 | 7.7 | 41.0 | 46.8 | [251] |
| 547 | *Escherichia coli* | FA | H | 57 | 7.5 | 38.5 | 45.8 | [251] |

(Table S2 continued.)

| Code | Strain/species name | Aero. | Troph. | Smp. | $T_{min}$ | $T_{opt}$ | $T_{max}$ | Lit. |
|---|---|---|---|---|---|---|---|---|
| 548 | *Escherichia coli* | FA | H | 27 | 7.6 | 43.6 | 47.4 | [251] |
| 549 | *Escherichia coli* | FA | H | 26 | 10.4 | 40.1 | 47.2 | [251] |
| 550 | *Escherichia coli* | FA | H | 26 | 8.1 | 39.6 | 43.6 | [251] |
| 551 | *Escherichia coli* | FA | H | 48 | 8.0 | 41.4 | 44.8 | [251] |
| 552 | *Escherichia coli* | FA | H | 26 | 8.9 | 41.6 | 46.5 | [251] |
| 553 | *Escherichia coli* | FA | H | 25 | 8.9 | 43.6 | 45.9 | [251] |
| 554 | *Escherichia coli* | FA | H | 25 | 8.7 | 43.5 | 45.4 | [251] |
| 555 | *Escherichia coli* | FA | H | 26 | 9.0 | 41.2 | 46.5 | [251] |
| 556 | *Candida stellata* | FA | H | 15 | 4.0 | 22.0 | 29.0 | [252] |
| 557 | *Hanseniaspora uvarum* | FA | H | 21 | 4.0 | 22.0 | 33.0 | [252] |
| 558 | *Kluyveromyces marxianus* | FA | H | 27 | 4.0 | 40.0 | 42.0 | [252] |
| 559 | *Pichia fermentans* | FA | H | 21 | 4.0 | 22.0 | 37.0 | [252] |
| 560 | *Saccharomyces arboricolus* | FA | H | 24 | 4.0 | 33.0 | 37.0 | [252] |
| 561 | *Saccharomyces bayanus* | FA | H | 24 | 4.0 | 29.0 | 37.0 | [252] |
| 562 | *Saccharomyces bayanus* | FA | H | 21 | 4.0 | 29.0 | 33.0 | [252] |
| 563 | *Saccharomyces cariocanus* | FA | H | 27 | 4.0 | 33.0 | 40.0 | [252] |
| 564 | *Saccharomyces cerevisiae* | FA | H | 27 | 4.0 | 37.0 | 42.0 | [252] |
| 565 | *Saccharomyces cerevisiae* | FA | H | 27 | 8.0 | 37.0 | 42.0 | [252] |
| 566 | *Saccharomyces cerevisiae* | FA | H | 21 | 4.0 | 33.0 | 40.0 | [252] |
| 567 | *Saccharomyces cerevisiae* | FA | H | 24 | 4.0 | 33.0 | 42.0 | [252] |
| 568 | *Saccharomyces cerevisiae* | FA | H | 30 | 4.0 | 37.0 | 42.0 | [252] |
| 569 | *Saccharomyces cerevisiae* | FA | H | 24 | 4.0 | 33.0 | 42.0 | [252] |
| 570 | *Saccharomyces cerevisiae* | FA | H | 24 | 4.0 | 33.0 | 42.0 | [252] |
| 571 | *Saccharomyces cerevisiae* | FA | H | 30 | 4.0 | 29.0 | 42.0 | [252] |
| 572 | *Saccharomyces cerevisiae* | FA | H | 30 | 4.0 | 37.0 | 42.0 | [252] |

(Table S2 continued.)

| Code | Strain/species name | Aero. | Troph. | Smp. | $T_{\mathrm{min}}$ | $T_{\mathrm{opt}}$ | $T_{\mathrm{max}}$ | Lit. |
|---|---|---|---|---|---|---|---|---|
| 573 | *Saccharomyces cerevisiae* | FA | H | 24 | 4.0 | 33.0 | 42.0 | [252] |
| 574 | *Saccharomyces kudriavzevii* | FA | H | 21 | 4.0 | 22.0 | 33.0 | [252] |
| 575 | *Saccharomyces kudriavzevii* | FA | H | 21 | 4.0 | 22.0 | 33.0 | [252] |
| 576 | *Saccharomyces kudriavzevii* | FA | H | 21 | 4.0 | 22.0 | 33.0 | [252] |
| 577 | *Saccharomyces kudriavzevii* | FA | H | 21 | 4.0 | 22.0 | 33.0 | [252] |
| 578 | *Saccharomyces mikatae* | FA | H | 24 | 4.0 | 29.0 | 37.0 | [252] |
| 579 | *Saccharomyces paradoxus* | FA | H | 24 | 4.0 | 33.0 | 37.0 | [252] |
| 580 | *Saccharomyces paradoxus* | FA | H | 27 | 4.0 | 29.0 | 40.0 | [252] |
| 581 | *Saccharomyces paradoxus* | FA | H | 27 | 4.0 | 29.0 | 40.0 | [252] |
| 582 | *Torulaspora delbrueckii* | FA | H | 21 | 4.0 | 22.0 | 37.0 | [252] |
| 583 | *Anabaena cylindrica* | A | A | 1 | 29.0 | 29.0 | 29.0 | [253] |
| 584 | *Geobacillus toebii* | A | H | 1 | 68.0 | 68.0 | 68.0 | [254] |
| 585 | *Psychrobacter cryopegella* | A | H | 10 | -10.1 | 22.0 | 22.0 | [255] |
| 586 | *Desulfobacter curvatus* | HN | H | 24 | -0.7 | 19.9 | 22.7 | [256] |
| 587 | *Desulforhopalus species* | HN | H | 28 | 5.9 | 33.1 | 36.7 | [256] |
| 588 | *Amitus fuscipennis* | A | H | 4 | 15.0 | 25.0 | 30.0 | [257] |
| 589 | *Amitus fuscipennis* | A | H | 4 | 15.0 | 25.0 | 30.0 | [257] |
| 590 | *Amitus fuscipennis* | A | H | 4 | 15.0 | 25.0 | 30.0 | [257] |
| 591 | *Encarsia formosa* | A | H | 4 | 15.0 | 30.0 | 30.0 | [257] |
| 592 | *Trialeurodes vaporariorum* | A | H | 4 | 15.0 | 30.0 | 30.0 | [257] |
| 593 | *Aphelinus semiflavus* | A | H | 4 | 15.6 | 26.7 | 29.4 | [258] |
| 594 | *Praon palitans* | A | H | 3 | 18.3 | 23.9 | 23.9 | [258] |
| 595 | *Trioxys utilis* | A | H | 4 | 15.6 | 26.7 | 29.4 | [258] |
| 596 | *Chryseolinea serpens* | A | H | 1 | 25.0 | 25.0 | 25.0 | [259] |
| 597 | *Geobacillus gargensis* | A | H | 1 | 62.5 | 62.5 | 62.5 | [260] |

(Table S2 continued.)

| Code | Strain/species name | Aero. | Troph. | Smp. | $T_{min}$ | $T_{opt}$ | $T_{max}$ | Lit. |
|---|---|---|---|---|---|---|---|---|
| 598 | *Aphis citricola* | A | H | 5 | 14.6 | 27.2 | 29.5 | [261] |
| 599 | *Aphis gossypii* | A | H | 5 | 5.1 | 19.7 | 29.7 | [261] |
| 600 | *Toxoptera citricidus* | A | H | 6 | 11.3 | 27.2 | 27.2 | [261] |
| 601 | *Toxoptera citricidus* | A | H | 4 | 14.9 | 25.0 | 27.7 | [261] |
| 602 | *Aphis gossypii* | A | H | 6 | 10.0 | 25.0 | 35.0 | [262] |
| 603 | *Callosobruchus analis* | A | H | 3 | 25.0 | 30.0 | 35.0 | [263] |
| 604 | *Callosobruchus chinensis* | A | H | 3 | 25.0 | 30.0 | 35.0 | [263] |
| 605 | *Callosobruchus maculatus* | A | H | 4 | 20.0 | 32.5 | 35.0 | [263] |
| 606 | *Callosobruchus maculatus* | A | H | 3 | 25.0 | 30.0 | 35.0 | [263] |
| 607 | *Callosobruchus rhodesianus* | A | H | 4 | 20.0 | 30.0 | 35.0 | [263] |
| 608 | *Bemisia argentifolii* | A | H | 5 | 20.0 | 25.0 | 35.0 | [264] |
| 609 | *Brevicoryne brassicae* | A | H | 4 | 10.0 | 20.0 | 25.0 | [265] |
| 610 | *Hyadaphis pseudobrassicae* | A | H | 5 | 10.0 | 25.0 | 30.0 | [265] |
| 611 | *Myzus persicae* | A | H | 5 | 5.0 | 25.0 | 25.0 | [265] |
| 612 | *Bactrocera cucurbitae* | A | H | 4 | 16.0 | 29.0 | 29.0 | [266] |
| 613 | *Bactrocera dorsalis* | A | H | 3 | 18.0 | 29.0 | 29.0 | [266] |
| 614 | *Bactrocera latifrons* | A | H | 3 | 18.0 | 29.0 | 29.0 | [266] |
| 615 | *Ceratitis capitata* | A | H | 4 | 16.0 | 29.0 | 29.0 | [266] |
| 616 | *Chlorella pyrenoidosa* | A | A | 9 | 21.5 | 39.3 | 42.1 | [267] |
| 617 | *Chlorella pyrenoidosa* | A | A | 6 | 18.0 | 31.9 | 42.0 | [267] |
| 618 | *Chlorella pyrenoidosa* | A | A | 4 | 18.1 | 25.4 | 28.9 | [267] |
| 619 | *Chlorella pyrenoidosa* | A | A | 3 | 18.0 | 25.4 | 25.4 | [267] |
| 620 | *Anagasta kuehniella* | A | H | 4 | 20.0 | 25.0 | 27.5 | [268] |
| 621 | *Cotesia flavipes* | A | H | 4 | 22.0 | 25.0 | 31.0 | [269] |
| 622 | *Cotesia sesamiae* | A | H | 4 | 22.0 | 28.0 | 31.0 | [269] |

(Table S2 continued.)

| Code | Strain/species name | Aero. | Troph. | Smp. | $T_{\mathrm{min}}$ | $T_{\mathrm{opt}}$ | $T_{\mathrm{max}}$ | Lit. |
|---|---|---|---|---|---|---|---|---|
| 623 | *Cryptolestes ferrugineus* | A | H | 3 | 25.0 | 30.0 | 35.0 | [270] |
| 624 | *Cryptolestes ferrugineus* | A | H | 8 | 22.5 | 35.0 | 40.0 | [270] |
| 625 | *Cryptolestes ferrugineus* | A | H | 3 | 25.0 | 35.0 | 35.0 | [270] |
| 626 | *Dactylopius austrinus* | A | H | 8 | 17.5 | 30.0 | 34.0 | [271] |
| 627 | *Macrolophus pygmaeus* | A | H | 5 | 15.0 | 27.5 | 30.0 | [272] |
| 628 | *Macrolophus pygmaeus* | A | H | 5 | 15.0 | 27.5 | 30.0 | [272] |
| 629 | *Nephaspis oculatus* | A | H | 5 | 20.0 | 26.0 | 31.0 | [273] |
| 630 | *Bactrocera dorsalis* | A | H | 6 | 19.0 | 34.0 | 34.0 | [274] |
| 631 | *Oryzaephilus surinamensis* | A | H | 6 | 20.0 | 30.0 | 35.0 | [275] |
| 632 | *Oryzaephilus surinamensis* | A | H | 6 | 20.0 | 32.5 | 35.0 | [275] |
| 633 | *Oryzaephilus surinamensis* | A | H | 6 | 20.0 | 32.5 | 35.0 | [275] |
| 634 | *Oryzaephilus surinamensis* | A | H | 8 | 20.1 | 29.7 | 32.7 | [276] |
| 635 | *Oryzaephilus surinamensis* | A | H | 10 | 20.3 | 32.6 | 34.9 | [276] |
| 636 | *Oryzaephilus surinamensis* | A | H | 10 | 20.0 | 32.1 | 34.6 | [276] |
| 637 | *Oryzaephilus surinamensis* | A | H | 10 | 19.9 | 32.1 | 34.6 | [276] |
| 638 | *Rhyzopertha dominica* | A | H | 12 | 15.2 | 29.7 | 38.5 | [276] |
| 639 | *Rhyzopertha dominica* | A | H | 14 | 15.4 | 33.1 | 38.9 | [276] |
| 640 | *Rhyzopertha dominica* | A | H | 13 | 15.2 | 32.6 | 38.3 | [276] |
| 641 | *Rhyzopertha dominica* | A | H | 14 | 15.1 | 32.6 | 38.5 | [276] |
| 642 | *Sitophilus oryzae* | A | H | 10 | 15.1 | 23.4 | 32.1 | [276] |
| 643 | *Sitophilus oryzae* | A | H | 10 | 15.4 | 27.2 | 32.5 | [276] |
| 644 | *Sitophilus oryzae* | A | H | 10 | 15.1 | 26.8 | 32.2 | [276] |
| 645 | *Sitophilus oryzae* | A | H | 10 | 15.1 | 26.8 | 32.2 | [276] |
| 646 | *Tribolium castaneum* | A | H | 9 | 24.3 | 35.0 | 37.5 | [276] |
| 647 | *Tribolium castaneum* | A | H | 12 | 20.9 | 34.5 | 38.3 | [276] |

(Table S2 continued.)

| Code | Strain/species name | Aero. | Troph. | Smp. | $T_{\mathrm{min}}$ | $T_{\mathrm{opt}}$ | $T_{\mathrm{max}}$ | Lit. |
|---|---|---|---|---|---|---|---|---|
| 648 | *Tribolium castaneum* | A | H | 13 | 20.0 | 34.6 | 39.5 | [276] |
| 649 | *Pterohelaeus alternatus* | A | H | 4 | 20.6 | 28.6 | 33.1 | [277] |
| 650 | *Pterohelaeus darlingensis* | A | H | 5 | 15.8 | 28.6 | 33.1 | [277] |
| 651 | *Calandra oryzae* | A | H | 1 | 25.5 | 25.5 | 25.5 | [278] |
| 652 | *Calandra oryzae* | A | H | 3 | 18.2 | 24.3 | 29.1 | [278] |
| 653 | *Calandra oryzae* | A | H | 3 | 23.0 | 28.5 | 33.5 | [278] |
| 654 | *Calandra oryzae* | A | H | 7 | 15.2 | 25.3 | 33.5 | [278] |
| 655 | *Rhizopertha dominica* | A | H | 3 | 26.0 | 32.0 | 36.0 | [278] |
| 656 | *Rhizopertha dominica* | A | H | 2 | 22.0 | 30.1 | 38.2 | [278] |
| 657 | *Rhizopertha dominica* | A | H | 4 | 29.0 | 33.4 | 38.2 | [278] |
| 658 | *Saccharicoccus sacchari* | A | H | 5 | 20.0 | 30.0 | 33.0 | [279] |
| 659 | *Scirtothrips perseae* | A | H | 4 | 15.0 | 20.0 | 27.5 | [280] |
| 660 | *Sitobion miscanthi* | A | H | 9 | 12.0 | 25.0 | 28.0 | [281] |
| 661 | *Sitobion near* | A | H | 4 | 12.0 | 20.0 | 25.0 | [281] |
| 662 | *Thrips tabaci* | A | H | 4 | 15.0 | 25.0 | 25.0 | [282] |
| 663 | *Trichogramma pretiosum* | A | H | 6 | 18.0 | 30.0 | 32.0 | [283] |
| 664 | *Trichogramma pretiosum* | A | H | 6 | 18.0 | 30.0 | 32.0 | [283] |
| 665 | *Rhopalosiphum rufiabdominalis* | A | H | 5 | 10.0 | 25.0 | 30.0 | [284] |
| 666 | *Muscidifurax zaraptor* | A | H | 7 | 14.9 | 31.5 | 32.9 | [285] |
| 667 | *Muscidifurax raptorellus* | A | H | 5 | 14.8 | 31.4 | 32.9 | [286] |
| 668 | *Callosobruchus maculatus* | A | H | 7 | 20.0 | 35.0 | 40.0 | [287] |
| 669 | *Eriosoma lanigerum* | A | H | 6 | 10.0 | 25.0 | 30.0 | [288] |
| 670 | *Diatraea lineolata* | A | H | 5 | 22.0 | 25.0 | 31.0 | [289] |
| 671 | *Hyperomyzus lactucae* | A | H | 4 | 17.0 | 24.0 | 26.0 | [290] |
| 672 | *Tribolium castaneum* | A | H | 1 | 30.0 | 30.0 | 30.0 | [291] |

(Table S2 continued.)

| Code | Strain/species name | Aero. | Troph. | Smp. | $T_{\mathrm{min}}$ | $T_{\mathrm{opt}}$ | $T_{\mathrm{max}}$ | Lit. |
|---|---|---|---|---|---|---|---|---|
| 673 | *Tribolium castaneum* | A | H | 4 | 25.0 | 35.0 | 35.0 | [291] |
| 674 | *Tribolium castaneum* | A | H | 7 | 22.5 | 37.5 | 37.5 | [291] |
| 675 | *Tribolium castaneum* | A | H | 1 | 25.0 | 25.0 | 25.0 | [291] |
| 676 | *Sitophilus oryzae* | A | H | 7 | 15.0 | 27.0 | 32.3 | [292] |
| 677 | *Sitophilus oryzae* | A | H | 7 | 15.0 | 27.0 | 32.3 | [292] |
| 678 | *Sitophilus oryzae* | A | H | 7 | 15.0 | 27.0 | 32.3 | [292] |
| 679 | *Trichogrammatoidea bactrae* | A | H | 5 | 15.0 | 25.0 | 32.5 | [293] |
| 680 | *Natranaerobius trueperi* | AN | H | 1 | 52.0 | 52.0 | 52.0 | [294] |
| 681 | *Natronovirga wadinatrunensis* | AN | H | 1 | 51.0 | 51.0 | 51.0 | [294] |
| 682 | *Thermotalea metallivorans* | AN | H | 1 | 50.0 | 50.0 | 50.0 | [295] |
| 683 | *Anaerolinea thermophila* | AN | H | 1 | 55.0 | 55.0 | 55.0 | [296] |
| 684 | *Caldilinea aerophila* | A | H | 1 | 55.0 | 55.0 | 55.0 | [296] |
| 685 | *Acyrthosiphon kondoi* | A | H | 8 | 6.0 | 20.5 | 20.5 | [297] |
| 686 | *Halanaerobium sehlinense* | AN | H | 1 | 43.0 | 43.0 | 43.0 | [298] |
| 687 | *Balneola vulgaris* | A | H | 7 | 10.1 | 30.0 | 40.1 | [299] |
| 688 | *Ruminococcus hydrogenotrophicus* | AN | A | 1 | 37.0 | 37.0 | 37.0 | [300] |
| 689 | *Verminephrobacter aporrectodeae* | microA | H | 1 | 20.0 | 20.0 | 20.0 | [301] |
| 690 | *Verminephrobacter aporrectodeae* | microA | H | 1 | 20.0 | 20.0 | 20.0 | [301] |
| 691 | *Verminephrobacter eiseniae* | A | H | 1 | 25.0 | 25.0 | 25.0 | [302] |
| 692 | *Thermoanaerobacterium sp.* | AN | H | 1 | 50.0 | 50.0 | 50.0 | [303] |
| 693 | *Thermoanaerobacterium sp.* | AN | H | 1 | 50.0 | 50.0 | 50.0 | [303] |
| 694 | *Thermoanaerobacterium sp.* | AN | H | 1 | 50.0 | 50.0 | 50.0 | [303] |
| 695 | *Thermoanaerobacterium sp.* | AN | H | 1 | 50.0 | 50.0 | 50.0 | [303] |
| 696 | *Hydrogenophilus islandicus* | A | M | 1 | 50.0 | 50.0 | 50.0 | [304] |
| 697 | *Thermovorax subterraneus* | AN | H | 1 | 71.0 | 71.0 | 71.0 | [305] |

(Table S2 continued.)

| Code | Strain/species name | Aero. | Troph. | Smp. | $T_{\mathrm{min}}$ | $T_{\mathrm{opt}}$ | $T_{\mathrm{max}}$ | Lit. |
|---|---|---|---|---|---|---|---|---|
| 698 | *Sulfurihydrogenibium azorense* | microA | A | 1 | 68.0 | 68.0 | 68.0 | [306] |
| 699 | *Thermodesulfatator atlanticus* | AN | A | 5 | 55.0 | 65.0 | 75.0 | [307] |
| 700 | *Methanobacterium petrolearium* | AN | H | 5 | 20.0 | 35.0 | 40.0 | [308] |
| 701 | *Methanobacterium ferruginis* | AN | A | 6 | 20.0 | 40.0 | 45.0 | [308] |
| 702 | *Thermodesulfatator indicus* | AN | A | 1 | 70.0 | 70.0 | 70.0 | [309] |
| 703 | *Thermococcus celericrescens* | AN | H | 7 | 49.9 | 79.8 | 84.8 | [310] |
| 704 | *Moorella humiferrea* | AN | H | 1 | 65.0 | 65.0 | 65.0 | [311] |
| 705 | *Thermosulfidibacter takaii* | AN | A | 6 | 54.9 | 70.0 | 78.0 | [312] |
| 706 | *Sulfurihydrogenibium subterraneum* | AN | A | 1 | 62.5 | 62.5 | 62.5 | [313] |
| 707 | *Thermococcus gammatolerans* | AN | H | 9 | 55.1 | 89.4 | 95.4 | [314] |
| 708 | *Persephonella hydrogeniphil* | AN | A | 7 | 50.1 | 70.2 | 72.6 | [315] |
| 709 | *Methanotorris formicicus* | AN | A | 7 | 54.8 | 74.7 | 82.8 | [316] |
| 710 | *Sulfurihydrogenibium rodmanii* | microA | A | 12 | 54.9 | 74.9 | 80.0 | [317] |
| 711 | *Sulfurihydrogenibium kristjanssonii* | A | H | 30 | 40.0 | 67.8 | 72.8 | [318] |
| 712 | *Gemmatimonas aurantiaca* | A | H | 1 | 30.0 | 30.0 | 30.0 | [319] |
| 713 | *Thermosipho affectus* | AN | H | 1 | 70.0 | 70.0 | 70.0 | [320] |
| 714 | *Nautilia nitratireducens* | AN | A | 8 | 24.6 | 54.8 | 60.0 | [321] |
| 715 | *Tepidiphilus margaritifer* | A | H | 1 | 50.0 | 50.0 | 50.0 | [322] |
| 716 | *Thermoanaerobacter sulfurigignens* | AN | H | 18 | 34.0 | 63.1 | 72.0 | [323] |
| 717 | *Methanobacterium paludis* | AN | A | 1 | 34.5 | 34.5 | 34.5 | [324] |
| 718 | *Nautilia profundicola* | AN | M | 6 | 29.8 | 39.9 | 55.1 | [325] |
| 719 | *Thermococcus thioreducens* | AN | H | 1 | 84.0 | 84.0 | 84.0 | [326] |
| 720 | *Thermosipho globiformans* | AN | H | 7 | 39.9 | 68.1 | 75.0 | [327] |
| 721 | *Methanobacterium lacuss* | AN | A | 1 | 25.0 | 25.0 | 25.0 | [328] |
| 722 | *Thermococcus nautili* | AN | H | 9 | 56.9 | 87.8 | 92.3 | [329] |

(Table S2 continued.)

| Code | Strain/species name | Aero. | Troph. | Smp. | $T_{\mathrm{min}}$ | $T_{\mathrm{opt}}$ | $T_{\mathrm{max}}$ | Lit. |
|---|---|---|---|---|---|---|---|---|
| 723 | *Thermosipho atlanticus* | AN | H | 1 | 65.0 | 65.0 | 65.0 | [330] |
| 724 | *Selenomonas bovis* | AN | H | 1 | 39.0 | 39.0 | 39.0 | [331] |
| 725 | *Mahella australiensis* | AN | H | 1 | 50.0 | 50.0 | 50.0 | [332] |
| 726 | *Thermoanaerobacter pseudethanolicus* | AN | H | 1 | 65.0 | 65.0 | 65.0 | [333] |
| 727 | *Thioprofundum hispidum* | AN | A | 1 | 39.0 | 39.0 | 39.0 | [334] |
| 728 | *Thermodesulfovibrio aggregans* | AN | H | 1 | 60.0 | 60.0 | 60.0 | [335] |
| 729 | *Thermosulfurimonas dismutans* | AN | A | 12 | 49.9 | 74.1 | 91.8 | [336] |
| 730 | *Methylothermus subterraneus* | A | H | 1 | 57.5 | 57.5 | 57.5 | [337] |
| 731 | *Tepidimicrobium xylanilyticum* | AN | H | 1 | 60.0 | 60.0 | 60.0 | [338] |
| 732 | *Caldisericum exile* | AN | H | 4 | 55.1 | 65.0 | 70.0 | [339] |
| 733 | *Thermococcus prieurii* | AN | H | 9 | 57.0 | 88.6 | 93.1 | [340] |
| 734 | *Thermosipho activus* | AN | H | 1 | 65.0 | 65.0 | 65.0 | [341] |
| 735 | *Moorella stamsii* | AN | H | 1 | 65.0 | 65.0 | 65.0 | [342] |
| 736 | *Thermobacillus xylanilyticus* | A | H | 1 | 55.0 | 55.0 | 55.0 | [343] |
| 737 | *Tepidimonas ignava* | A | H | 7 | 29.8 | 54.9 | 60.1 | [344] |
| 738 | *Staphylothermus hellenicus* | AN | H | 1 | 85.0 | 85.0 | 85.0 | [345] |
| 739 | *Thermococcus aegaeicus* | AN | H | 1 | 90.0 | 90.0 | 90.0 | [345] |
| 740 | *Thermoanaerobacterium polysaccharolyticum* | AN | H | 1 | 68.0 | 68.0 | 68.0 | [346] |
| 741 | *Thermoanaerobacterium polysaccharolyticum* | AN | H | 1 | 68.0 | 68.0 | 68.0 | [346] |
| 742 | *Thermoanaerobacterium polysaccharolyticum* | AN | H | 1 | 68.0 | 68.0 | 68.0 | [346] |
| 743 | *Thermoanaerobacterium zeae* | AN | H | 1 | 67.5 | 67.5 | 67.5 | [346] |
| 744 | *Methanobacterium congolense* | AN | A | 1 | 39.5 | 39.5 | 39.5 | [347] |
| 745 | *Thermaerobacter subterraneus* | A | H | 1 | 70.0 | 70.0 | 70.0 | [348] |
| 746 | *Petrotoga olearia* | AN | H | 4 | 45.1 | 54.9 | 60.0 | [349] |
| 747 | *Thermovibrio ruber* | AN | A | 1 | 75.0 | 75.0 | 75.0 | [350] |

(Table S2 continued.)

| Code | Strain/species name | Aero. | Troph. | Smp. | $T_{min}$ | $T_{opt}$ | $T_{max}$ | Lit. |
|---|---|---|---|---|---|---|---|---|
| 748 | *Sulfobacillus benefaciens* | FA | M | 7 | 29.9 | 38.5 | 45.0 | [351] |
| 749 | *Thiobacillus thermosulfatu* | A | A | 2 | 50.0 | 50.0 | 52.5 | [352] |
| 750 | *Phenylobacterium immobile* | A | H | 1 | 29.0 | 29.0 | 29.0 | [353] |
| 751 | *Lebetimonas acidiphila* | AN | A | 7 | 30.2 | 50.4 | 67.2 | [354] |
| 752 | *Alicyclobacillus pomorum* | FA | H | 1 | 47.5 | 47.5 | 47.5 | [355] |
| 753 | *Tepidibacter formicigenes* | AN | H | 1 | 45.0 | 45.0 | 45.0 | [356] |
| 754 | *Methanohalophilus levihalophilus* | AN | H | 1 | 35.0 | 35.0 | 35.0 | [357] |
| 755 | *Anaerolinea thermolimosa* | AN | H | 1 | 50.0 | 50.0 | 50.0 | [358] |
| 756 | *Leptolinea tardivitalis* | AN | H | 1 | 37.0 | 37.0 | 37.0 | [358] |
| 757 | *Levilinea saccharolytica* | AN | H | 1 | 38.5 | 38.5 | 38.5 | [358] |
| 758 | *Caldilinea tarbellica* | AN | H | 1 | 55.0 | 55.0 | 55.0 | [359] |
| 759 | *Thermotoga elfii* | AN | H | 1 | 66.0 | 66.0 | 66.0 | [360] |
| 760 | *Litorilinea aerophila* | A | H | 1 | 55.0 | 55.0 | 55.0 | [361] |
| 761 | *Erwinia amylovora* | FA | H | 10 | 6.5 | 30.0 | 33.0 | [362] |
| 762 | *Thermoanaerobacter wiegelii* | AN | H | 1 | 67.5 | 67.5 | 67.5 | [363] |
| 763 | *Rhodothermus obamensis* | A | H | 1 | 80.0 | 80.0 | 80.0 | [364] |
| 764 | *Deferribacter abyssi* | AN | A | 1 | 60.0 | 60.0 | 60.0 | [365] |
| 765 | *Porphyrobacter tepidarius* | A | H | 9 | 30.1 | 42.7 | 50.3 | [366] |
| 766 | *Methanobacterium subterraneum* | AN | A | 9 | 3.7 | 40.0 | 44.9 | [367] |
| 767 | *Petrotoga mobilis* | AN | H | 1 | 59.0 | 59.0 | 59.0 | [368] |
| 768 | *Melitea salexigens* | A | H | 7 | 10.0 | 30.0 | 43.9 | [369] |
| 769 | *Thiomicrospira chilensis* | A | A | 1 | 29.0 | 29.0 | 29.0 | [370] |
| 770 | *Archaeal str.* | AN | A | 10 | 84.9 | 104.9 | 120.9 | [371] |
| 771 | *Hydrogenimonas thermophila* | microA | A | 5 | 35.0 | 55.0 | 60.0 | [372] |
| 772 | *Saccharomyces bayanus* | FA | H | 7 | 21.5 | 30.0 | 35.0 | [373] |

(Table S2 continued.)

| Code | Strain/species name | Aero. | Troph. | Smp. | $T_{min}$ | $T_{opt}$ | $T_{max}$ | Lit. |
|---|---|---|---|---|---|---|---|---|
| 773 | *Saccharomyces cerevisiae* | FA | H | 7 | 25.0 | 35.0 | 38.0 | [373] |
| 774 | *Candida valida* | FA | H | 15 | 17.0 | 36.3 | 43.0 | [374] |
| 775 | *Brettanomyces bruxellensis* | FA | H | 6 | 15.0 | 32.0 | 35.0 | [375] |
| 776 | *Synechococcus clone* | A | A | 7 | 30.0 | 45.0 | 57.0 | [376] |
| 777 | *Synechococcus clone* | A | A | 7 | 30.0 | 45.0 | 57.0 | [376] |
| 778 | *Synechococcus clone* | A | A | 7 | 30.0 | 45.0 | 57.0 | [376] |
| 779 | *Synechococcus clone* | A | A | 7 | 30.0 | 45.0 | 57.0 | [376] |
| 780 | *Synechococcus clone* | A | A | 7 | 30.0 | 45.0 | 57.0 | [376] |
| 781 | *Caloranaerobacter ferrireducens* | AN | H | 1 | 60.0 | 60.0 | 60.0 | [377] |
| 782 | *Thermococcus sibiricus* | AN | H | 1 | 78.0 | 78.0 | 78.0 | [378] |
| 783 | *Escherichia coli* | FA | H | 13 | 8.0 | 36.7 | 43.0 | [379] |
| 784 | *Escherichia coli* | FA | H | 15 | -0.1 | 37.0 | 42.8 | [379] |
| 785 | *Thalassobaculum salexigens* | A | H | 7 | 9.9 | 30.0 | 43.9 | [380] |
| 786 | *Echinamoeba thermarum* | A | H | 5 | 43.1 | 50.0 | 55.1 | [381] |
| 787 | *Amoeba algonquinensis* | A | H | 4 | 5.0 | 12.5 | 12.5 | [382] |
| 788 | *Saccamoeba limax* | A | H | 4 | 5.0 | 12.5 | 12.5 | [382] |
| 789 | *Vannella sp.* | A | H | 4 | 5.0 | 12.5 | 12.5 | [382] |
| 790 | *Vannella sp.* | A | H | 4 | 5.0 | 12.5 | 12.5 | [382] |
| 791 | *Acanthamoeba polyphaga* | A | H | 4 | 10.0 | 20.0 | 25.0 | [383] |
| 792 | *Cochliopodium minus* | A | H | 4 | 10.0 | 25.0 | 25.0 | [383] |
| 793 | *Glaeseria mira* | A | H | 4 | 10.0 | 25.0 | 25.0 | [383] |
| 794 | *Saccamoeba limax* | A | H | 4 | 10.0 | 20.0 | 25.0 | [383] |
| 795 | *Vannella sp.* | A | H | 4 | 10.0 | 25.0 | 25.0 | [383] |
| 796 | *Vexillifera bacillipedes* | A | H | 3 | 15.0 | 25.0 | 25.0 | [383] |
| 797 | *Meiothermus cerberus* | A | H | 6 | 35.0 | 55.2 | 60.1 | [384] |

(Table S2 continued.)

| Code | Strain/species name | Aero. | Troph. | Smp. | $T_{\mathrm{min}}$ | $T_{\mathrm{opt}}$ | $T_{\mathrm{max}}$ | Lit. |
|---|---|---|---|---|---|---|---|---|
| 798 | *Sulfolobus sp.* | A | M | 7 | 74.9 | 87.1 | 88.8 | [385] |
| 799 | *Sulfolobus sp.* | A | M | 5 | 59.7 | 75.0 | 77.0 | [385] |
| 800 | *Picrophilus oshimae* | A | H | 6 | 48.0 | 60.1 | 63.0 | [386] |
| 801 | *Kluyveromyces marxianus* | FA | H | 18 | 7.0 | 37.0 | 46.9 | [387] |
| 802 | *Thermus thermophilus* | A | H | 23 | 53.1 | 66.9 | 83.2 | [388] |
| 803 | *Thermus aquaticus* | A | H | 11 | 46.0 | 69.7 | 78.7 | [389] |
| 804 | *Methanococcus jannaschii* | AN | A | 7 | 51.3 | 85.6 | 85.6 | [390] |
| 805 | *Alicyclobacillus acidocaldarius* | FA | H | 6 | 45.0 | 65.1 | 70.1 | [391] |
| 806 | *Alicyclobacillus acidocaldarius* | FA | H | 6 | 45.0 | 65.1 | 69.9 | [391] |
| 807 | *Alicyclobacillus acidocaldarius* | FA | H | 5 | 45.0 | 65.1 | 65.1 | [391] |
| 808 | *Pelotomaculum thermopropionicum* | AN | H | 5 | 45.0 | 55.0 | 65.0 | [392] |
| 809 | *Thermacetogenium phaeum* | AN | A | 7 | 40.0 | 58.0 | 65.1 | [393] |
| 810 | *Geobacillus stearothermophilus* | FA | A | 6 | 37.0 | 55.0 | 70.0 | [394] |
| 811 | *Geobacillus thermoleovorans* | FA | H | 5 | 44.9 | 65.0 | 69.8 | [395] |
| 812 | *Thermoanaerobacter kivui* | AN | H | 6 | 54.1 | 64.0 | 69.8 | [396] |
| 813 | *Thermoanaerobacter mathranii* | AN | H | 6 | 50.1 | 68.9 | 75.0 | [397] |
| 814 | *Caldicellulosiruptor changbaiensis* | AN | H | 8 | 39.9 | 74.4 | 95.0 | [398] |
| 815 | *Chlorobium tepidum* | AN | A | 11 | 32.1 | 46.9 | 51.1 | [399] |
| 816 | *Halococcus hamelinensis* | A | H | 1 | 37.0 | 37.0 | 37.0 | [400] |
| 817 | *Aspergillus candidus* | A | H | 8 | 11.9 | 29.8 | 37.4 | [401] |
| 818 | *Saccharomyces uvarum* | FA | H | 6 | 25.0 | 33.0 | 43.0 | [402] |
| 819 | *Candida curiosa* | FA | H | 12 | 8.2 | 12.7 | 14.1 | [403] |
| 820 | *Cryptococcus neoformus* | FA | H | 22 | 29.4 | 37.0 | 39.4 | [403] |
| 821 | *Globigerina bulloides* | A | H | 8 | 9.8 | 22.0 | 25.1 | [404] |
| 822 | *Globigerinella siphonifera* | A | H | 18 | 11.8 | 26.1 | 29.7 | [404] |

(Table S2 continued.)

| Code | Strain/species name | Aero. | Troph. | Smp. | $T_{\min}$ | $T_{\text{opt}}$ | $T_{\max}$ | Lit. |
|---|---|---|---|---|---|---|---|---|
| 823 | *Globigerinoides ruber* | A | H | 11 | 15.4 | 26.5 | 30.7 | [404] |
| 824 | *Globigerinoides sacculifer* | A | H | 29 | 13.9 | 29.1 | 31.1 | [404] |
| 825 | *Neogloboquadrina dutertrei* | A | H | 9 | 8.8 | 16.2 | 31.6 | [404] |
| 826 | *Neogloboquadrina pachyderma* | A | H | 5 | 5.9 | 19.2 | 19.2 | [404] |
| 827 | *Orbulina universa* | A | H | 23 | 15.0 | 26.1 | 30.8 | [404] |
| 828 | *Pyrococcus abyssi* | AN | H | 8 | 72.1 | 95.9 | 101.5 | [405] |
| 829 | *Thermoanaerobacter subterraneus* | AN | H | 7 | 39.9 | 65.1 | 75.1 | [406] |
| 830 | *Isosphaera pallida* | A | H | 7 | 28.0 | 40.6 | 54.2 | [407] |
| 831 | *Thermoproteus uzoniensis* | AN | H | 5 | 78.3 | 86.3 | 97.4 | [408] |
| 832 | *Acidothermus cellulolyticus* | A | H | 3 | 44.9 | 54.8 | 59.9 | [409] |
| 833 | *Acidothermus cellulolyticus* | A | H | 4 | 44.7 | 48.8 | 60.6 | [409] |
| 834 | *Acidothermus cellulolyticus* | A | H | 4 | 44.8 | 59.4 | 64.9 | [409] |
| 835 | *Hydrogenobacter thermophilus* | A | A | 9 | 50.0 | 71.7 | 78.7 | [410] |
| 836 | *Syntrophothermus lipocalidus* | AN | H | 4 | 44.9 | 55.4 | 60.3 | [411] |
| 837 | *Thermoanaerobacter tengcongensis* | AN | H | 8 | 50.1 | 75.3 | 80.1 | [412] |
| 838 | *Persephonella guaymasensis* | microA | A | 13 | 60.0 | 70.2 | 75.1 | [413] |
| 839 | *Persephonella marina* | microA | A | 17 | 60.0 | 73.2 | 77.2 | [413] |
| 840 | *Thiobacillus caldus* | A | M | 6 | 31.9 | 45.1 | 52.0 | [414] |
| 841 | *Thermodesulfobacterium commune* | AN | H | 7 | 50.0 | 69.6 | 84.8 | [415] |
| 842 | *Halomicronema excentricum* | A | A | 5 | 27.9 | 45.0 | 50.1 | [416] |
| 843 | *Halomicronema excentricum* | A | A | 5 | 21.6 | 39.9 | 45.3 | [416] |
| 844 | *Halomicronema excentricum* | A | A | 4 | 27.9 | 40.0 | 44.9 | [416] |
| 845 | *Thermotoga lettingae* | AN | H | 6 | 49.7 | 64.9 | 69.8 | [417] |
| 846 | *Clostridium thermosulfurogenes* | AN | H | 7 | 40.0 | 59.5 | 64.7 | [418] |
| 847 | *Halococcus dombrowskii* | A | H | 1 | 37.0 | 37.0 | 37.0 | [419] |

(Table S2 continued.)

| Code | Strain/species name | Aero. | Troph. | Smp. | $T_{\mathrm{min}}$ | $T_{\mathrm{opt}}$ | $T_{\mathrm{max}}$ | Lit. |
|---|---|---|---|---|---|---|---|---|
| 848 | *Methanobacterium flexile* | AN | H | 9 | 10.0 | 35.0 | 50.0 | [420] |
| 849 | *Methanobacterium movens* | AN | H | 9 | 10.0 | 35.0 | 50.0 | [420] |
| 850 | *Thermocrinis ruber* | A | M | 5 | 59.9 | 79.9 | 84.8 | [421] |
| 851 | *Clostridium perfringens* | AN | H | 4 | 30.0 | 45.0 | 45.0 | [422] |
| 852 | *Vibrio alginolyticus* | FA | H | 6 | 19.9 | 36.1 | 48.9 | [423] |
| 853 | *Clostridium perfringens* | AN | H | 48 | 15.0 | 43.0 | 51.9 | [424] |
| 854 | *Clostridium isatidis* | AN | H | 13 | 30.1 | 50.8 | 54.9 | [425] |
| 855 | *Thermohalobacter berrensis* | AN | H | 5 | 45.2 | 65.2 | 65.2 | [426] |
| 856 | *Clostridium sp.* | AN | H | 6 | 15.1 | 36.7 | 41.9 | [427] |
| 857 | *Geoglobus ahangari* | AN | A | 5 | 70.0 | 87.6 | 87.6 | [428] |
| 858 | *Rhodoferax antarcticus* | FA | M | 9 | 0.0 | 20.6 | 24.8 | [429] |
| 859 | *Methanogenium frigidum* | AN | A | 17 | -0.0 | 10.1 | 19.0 | [430] |
| 860 | *Methanoculleus submarinus* | AN | H | 20 | 14.9 | 45.0 | 49.9 | [431] |
| 861 | *Photobacterium frigidiphilum* | FA | H | 8 | 5.9 | 13.8 | 20.6 | [432] |
| 862 | *Pyrobaculum aerophilum* | FA | M | 5 | 85.0 | 99.0 | 100.0 | [433] |
| 863 | *Pyrobaculum calidifontis* | FA | H | 5 | 80.1 | 90.0 | 98.0 | [434] |
| 864 | *Thermococcus acidaminovorans* | AN | H | 6 | 54.9 | 84.9 | 94.9 | [435] |
| 865 | *Ignicoccus hospitalis* | AN | A | 7 | 71.8 | 87.0 | 93.8 | [436] |
| 866 | *Porphyrobacter cryptus* | A | H | 10 | 25.2 | 40.1 | 52.7 | [437] |
| 867 | *Porphyrobacter cryptus* | A | H | 11 | 25.1 | 50.4 | 55.3 | [437] |
| 868 | *Porphyrobacter cryptus* | A | H | 11 | 25.1 | 50.2 | 55.1 | [437] |
| 869 | *Alteromonas haloplanctis* | A | H | 3 | 4.0 | 18.0 | 25.0 | [438] |
| 870 | *Moraxella sp.* | A | H | 3 | 3.0 | 25.0 | 25.0 | [438] |
| 871 | *Moraxella sp.* | A | H | 2 | 3.0 | 17.0 | 17.0 | [438] |
| 872 | *Psychrobacter immobilis* | A | H | 2 | 4.0 | 25.0 | 25.0 | [438] |

(Table S2 continued.)

| Code | Strain/species name | Aero. | Troph. | Smp. | $T_{min}$ | $T_{opt}$ | $T_{max}$ | Lit. |
|---|---|---|---|---|---|---|---|---|
| 873 | *Bacillus sp.* | A | H | 2 | 4.0 | 25.0 | 25.0 | [438] |
| 874 | *Psychrobacter sp.* | A | H | 6 | 4.9 | 18.1 | 36.8 | [439] |
| 875 | *Psychrobacter sp.* | A | H | 6 | 5.1 | 18.1 | 37.0 | [439] |
| 876 | *Clostridium sp.* | AN | H | 6 | -5.1 | 5.9 | 14.9 | [439] |
| 877 | *Clostridium algoriphilium* | AN | H | 6 | -2.0 | 6.0 | 15.0 | [440] |
| 878 | *Colwellia demingiae* | FA | H | 18 | 0.3 | 13.0 | 15.7 | [441] |
| 879 | *Colwellia hornerae* | FA | H | 15 | 3.2 | 10.6 | 16.7 | [441] |
| 880 | *Colwellia psychotropica* | FA | H | 19 | 2.0 | 17.9 | 24.5 | [441] |
| 881 | *Colwellia psychrerythraea* | FA | H | 16 | 2.0 | 13.9 | 16.9 | [441] |
| 882 | *Psychromonas antarctica* | AN | H | 3 | 4.0 | 15.0 | 15.0 | [442] |
| 883 | *Psychromonas antarctica* | AN | H | 3 | 4.0 | 15.0 | 15.0 | [442] |
| 884 | *Psychromonas antarctica* | AN | H | 3 | 4.0 | 15.0 | 15.0 | [442] |
| 885 | *Psychromonas antarctica* | AN | H | 3 | 4.0 | 15.0 | 15.0 | [442] |
| 886 | *Psychromonas antarctica* | AN | H | 3 | 4.0 | 15.0 | 15.0 | [442] |
| 887 | *Psychromonas antarctica* | AN | H | 3 | 4.0 | 4.0 | 15.0 | [442] |
| 888 | *Psychromonas antarctica* | AN | H | 1 | 4.0 | 4.0 | 4.0 | [442] |
| 889 | *Psychromonas antarctica* | AN | H | 1 | 4.0 | 4.0 | 4.0 | [442] |
| 890 | *Psychromonas kaikoae* | AN | H | 2 | 4.0 | 10.0 | 10.0 | [442] |
| 891 | *Psychromonas kaikoae* | AN | H | 2 | 4.0 | 4.0 | 10.0 | [442] |
| 892 | *Psychromonas kaikoae* | AN | H | 3 | 4.0 | 4.0 | 15.0 | [442] |
| 893 | *Psychromonas kaikoae* | AN | H | 3 | 4.0 | 4.0 | 15.0 | [442] |
| 894 | *Psychromonas kaikoae* | AN | H | 3 | 4.0 | 10.0 | 15.0 | [442] |
| 895 | *Psychromonas kaikoae* | AN | H | 3 | 4.0 | 10.0 | 15.0 | [442] |
| 896 | *Psychromonas kaikoae* | AN | H | 3 | 4.0 | 15.0 | 15.0 | [442] |
| 897 | *Psychromonas kaikoae* | AN | H | 3 | 4.0 | 15.0 | 15.0 | [442] |

(Table S2 continued.)

| Code | Strain/species name | Aero. | Troph. | Smp. | $T_{min}$ | $T_{opt}$ | $T_{max}$ | Lit. |
|---|---|---|---|---|---|---|---|---|
| 898 | *Bacillus cereus* | FA | H | 9 | 10.0 | 37.0 | 43.0 | [443] |
| 899 | *Bacillus cereus* | FA | H | 10 | 10.0 | 40.0 | 44.0 | [443] |
| 900 | *Bacillus cereus* | FA | H | 10 | 10.0 | 40.0 | 44.0 | [443] |
| 901 | *Bacillus cereus* | FA | H | 9 | 8.0 | 30.0 | 37.0 | [443] |
| 902 | *Bacillus cereus* | FA | H | 7 | 10.0 | 35.0 | 37.0 | [443] |
| 903 | *Bacillus cereus* | FA | H | 7 | 10.0 | 35.0 | 37.0 | [443] |
| 904 | *Bacillus cereus* | FA | H | 8 | 10.0 | 37.0 | 40.0 | [443] |
| 905 | *Bacillus cereus* | FA | H | 9 | 10.0 | 42.0 | 42.0 | [443] |
| 906 | *Listeria monocytogenes* | FA | H | 11 | 2.0 | 40.0 | 40.0 | [443] |
| 907 | *Listeria monocytogenes* | FA | H | 12 | 2.0 | 40.0 | 42.0 | [443] |
| 908 | *Listeria monocytogenes* | FA | H | 16 | 2.0 | 37.0 | 43.0 | [443] |
| 909 | *Escherichia coli* | FA | H | 7 | 8.0 | 40.0 | 44.9 | [443] |
| 910 | *Escherichia coli* | FA | H | 7 | 8.0 | 40.1 | 45.0 | [443] |
| 911 | *Escherichia coli* | FA | H | 7 | 8.0 | 40.1 | 45.0 | [443] |
| 912 | *Escherichia coli* | FA | H | 7 | 8.0 | 40.1 | 45.0 | [443] |
| 913 | *Escherichia coli* | FA | H | 7 | 8.0 | 40.1 | 45.0 | [443] |
| 914 | *Escherichia coli* | FA | H | 7 | 8.0 | 40.1 | 45.0 | [443] |
| 915 | *Escherichia coli* | FA | H | 7 | 8.0 | 40.0 | 45.0 | [443] |
| 916 | *Escherichia coli* | FA | H | 7 | 8.0 | 40.1 | 45.0 | [443] |
| 917 | *Clostridium perfringens* | AN | H | 5 | 32.2 | 43.3 | 48.9 | [444] |
| 918 | *Clostridium perfringens* | AN | H | 25 | 15.6 | 44.7 | 48.9 | [445] |
| 919 | *Clostridium perfringens* | AN | H | 3 | 30.0 | 45.0 | 45.0 | [446] |
| 920 | *Salmonellae sp.* | FA | H | 6 | 10.0 | 35.0 | 35.0 | [447] |
| 921 | *Salmonellae sp.* | FA | H | 6 | 10.0 | 35.0 | 35.0 | [447] |
| 922 | *Salmonella typhimurium* | FA | H | 5 | 20.0 | 40.0 | 40.0 | [448] |

(Table S2 continued.)

| Code | Strain/species name | Aero. | Troph. | Smp. | $T_{min}$ | $T_{opt}$ | $T_{max}$ | Lit. |
|---|---|---|---|---|---|---|---|---|
| 923 | *Salmonella typhimurium* | FA | H | 6 | 15.0 | 40.0 | 40.0 | [448] |
| 924 | *Brochothrix thermosphacta* | FA | H | 7 | 0.0 | 25.1 | 30.1 | [449] |
| 925 | *Lactococcus piscium* | FA | H | 7 | 0.0 | 25.0 | 27.1 | [449] |
| 926 | *Brochothrix thermosphacta* | FA | H | 10 | 2.1 | 25.0 | 25.0 | [450] |
| 927 | *Brochothrix thermosphacta* | FA | H | 9 | 2.1 | 25.0 | 25.0 | [450] |
| 928 | *Salmonellae typhimurium* | FA | H | 2 | 25.0 | 37.0 | 37.0 | [451] |
| 929 | *Salmonellae typhimurium* | FA | H | 2 | 25.0 | 37.0 | 37.0 | [451] |
| 930 | *Salmonellae typhimurium* | FA | H | 2 | 25.0 | 37.0 | 37.0 | [451] |
| 931 | *Salmonellae typhimurium* | FA | H | 1 | 37.0 | 37.0 | 37.0 | [451] |
| 932 | *Salmonellae typhimurium* | FA | H | 2 | 25.0 | 37.0 | 37.0 | [451] |
| 933 | *Salmonella typhimurium* | FA | H | 1 | 25.0 | 25.0 | 25.0 | [451] |
| 934 | *Chloroflexus aurantiacus* | FA | H | 1 | 55.0 | 55.0 | 55.0 | [452] |
| 935 | *Chloroflexus aurantiacus* | FA | H | 1 | 55.0 | 55.0 | 55.0 | [452] |
| 936 | *Clostridium perfringens* | AN | H | 4 | 37.0 | 43.0 | 46.0 | [453] |
| 937 | *Clostridium perfringens* | AN | H | 4 | 37.0 | 43.0 | 46.0 | [453] |
| 938 | *Clostridium perfringens* | AN | H | 4 | 37.0 | 46.0 | 46.0 | [453] |
| 939 | *Clostridium perfringens* | AN | H | 4 | 37.0 | 46.0 | 46.0 | [453] |
| 940 | *Clostridium perfringens* | AN | H | 4 | 37.0 | 43.0 | 46.0 | [453] |
| 941 | *Clostridium perfringens* | AN | H | 4 | 37.0 | 43.0 | 46.0 | [453] |
| 942 | *Clostridium perfringens* | AN | H | 4 | 37.0 | 46.0 | 46.0 | [453] |
| 943 | *Clostridium perfringens* | AN | H | 4 | 37.0 | 43.0 | 46.0 | [453] |
| 944 | *Escherichia coli* | FA | H | 17 | 13.3 | 39.0 | 47.9 | [454] |
| 945 | *Klebsiella pneumoniae* | FA | H | 11 | 20.1 | 40.6 | 47.3 | [455] |
| 946 | *Klebsiella pneumoniae* | FA | H | 10 | 20.2 | 37.9 | 44.7 | [455] |
| 947 | *Coccobacillus sp.* | FA | H | 6 | 34.9 | 49.9 | 56.9 | [455] |

(Table S2 continued.)

| Code | Strain/species name | Aero. | Troph. | Smp. | $T_{\mathrm{min}}$ | $T_{\mathrm{opt}}$ | $T_{\mathrm{max}}$ | Lit. |
|---|---|---|---|---|---|---|---|---|
| 948 | *Bacillus sp.* | FA | H | 6 | 35.0 | 55.0 | 57.0 | [456] |
| 949 | *Aeromonas hydrophila* | FA | H | 5 | 5.0 | 40.0 | 40.0 | [457] |
| 950 | *Latris lineata* | A | H | 4 | 12.0 | 14.0 | 18.0 | [458] |
| 951 | *Dictyoglomus thermophilum* | AN | H | 7 | 51.0 | 78.3 | 80.3 | [459] |
| 952 | *Bacillus caldotenax* | FA | H | 5 | 49.7 | 65.0 | 69.6 | [460] |
| 953 | *Bacillus caldotenax* | FA | H | 8 | 39.8 | 64.9 | 70.0 | [460] |
| 954 | *Sulfurimonas paralvinellae* | microA | A | 7 | 4.2 | 30.1 | 34.9 | [461] |
| 955 | *Methanobacterium thermoautotrophicum* | AN | A | 9 | 44.8 | 64.9 | 70.2 | [462] |
| 956 | *Archaebacterial str.* | AN | H | 7 | 63.5 | 88.5 | 94.9 | [463] |
| 957 | *Eubacteria sp.* | AN | H | 7 | 45.7 | 77.3 | 88.2 | [464] |
| 958 | *Thermococcus chitonophagus* | AN | H | 7 | 65.0 | 85.0 | 92.9 | [465] |
| 959 | *Palaeococcus helgesonii* | FA | H | 7 | 45.1 | 75.2 | 84.9 | [466] |
| 960 | *Thermococcus waiotapuensis* | AN | H | 8 | 64.0 | 84.9 | 89.8 | [467] |
| 961 | *Thermococcus barossii* | AN | H | 6 | 60.0 | 82.9 | 92.0 | [468] |
| 962 | *Thermococcus celer* | AN | H | 6 | 60.0 | 82.9 | 91.6 | [468] |
| 963 | *Thermococcus atlanticus* | AN | H | 6 | 69.9 | 89.8 | 94.8 | [469] |
| 964 | *Campylobacter jejuni* | microA | H | 6 | 33.0 | 41.8 | 43.9 | [470] |
| 965 | *Campylobacter jejuni* | microA | H | 7 | 31.2 | 36.9 | 44.0 | [470] |
| 966 | *Pyrococcus furiosus* | AN | H | 9 | 70.2 | 100.0 | 103.1 | [471] |
| 967 | *Pyrobaculum islandicum* | AN | M | 5 | 89.9 | 98.9 | 101.9 | [472] |
| 968 | *Pyrobaculum organotrophum* | AN | H | 5 | 89.9 | 100.5 | 102.0 | [472] |
| 969 | *Anoxybacillus pushchinensis* | AN | H | 4 | 37.0 | 61.9 | 61.9 | [473] |
| 970 | *Thermococcus barophilus* | AN | H | 7 | 75.1 | 84.9 | 100.1 | [474] |
| 971 | *Thermococcus barophilus* | AN | H | 4 | 75.1 | 85.1 | 90.0 | [474] |
| 972 | *Thermoanaerobacter keratinophilus* | AN | H | 5 | 49.9 | 70.0 | 75.2 | [475] |

(Table S2 continued.)

| Code | Strain/species name | Aero. | Troph. | Smp. | $T_{min}$ | $T_{opt}$ | $T_{max}$ | Lit. |
|---|---|---|---|---|---|---|---|---|
| 973 | *Thermodesulfobium narugense* | AN | A | 1 | 55.0 | 55.0 | 55.0 | [476] |
| 974 | *Thermosipho geolei* | AN | H | 5 | 45.0 | 70.1 | 74.7 | [477] |
| 975 | *Thermotoga naphthophila* | AN | H | 9 | 46.8 | 79.3 | 84.3 | [478] |
| 976 | *Thermotoga petrophila* | AN | H | 9 | 45.5 | 79.5 | 84.3 | [478] |
| 977 | *Ferroglobus placidus* | AN | M | 5 | 75.2 | 85.1 | 95.2 | [479] |
| 978 | *Methanosarcina acetivorans* | AN | H | 7 | 15.0 | 40.1 | 45.0 | [480] |
| 979 | *Palaeococcus ferrophilus* | AN | H | 8 | 60.0 | 83.0 | 88.0 | [481] |
| 980 | *Pseudoxanthomonas broegbernensis* | A | H | 3 | 24.9 | 30.0 | 35.0 | [482] |
| 981 | *Pseudoxanthomonas taiwanensi* | A | H | 7 | 29.9 | 50.0 | 60.0 | [482] |
| 982 | *Thermosipho japonicus* | AN | H | 8 | 44.9 | 72.0 | 80.1 | [483] |
| 983 | *Hydrogenobacter subterraneus* | A | H | 7 | 60.1 | 77.9 | 85.2 | [484] |
| 984 | *Sulfurisphaera ohwakuensis* | FA | H | 6 | 65.3 | 85.2 | 90.3 | [485] |
| 985 | *Thermotoga subterranea* | AN | H | 6 | 50.1 | 69.9 | 75.0 | [486] |
| 986 | *Thermosipho melanesiensis* | AN | H | 6 | 50.1 | 69.9 | 75.1 | [487] |
| 987 | *Thermococcus alcaliphilus* | AN | H | 7 | 54.8 | 84.8 | 89.9 | [488] |
| 988 | *Thermococcus fumicolans* | AN | H | 7 | 76.4 | 84.1 | 103.2 | [489] |
| 989 | *Methanocorpusculum labreanum* | AN | H | 4 | 25.0 | 37.1 | 40.0 | [490] |
| 990 | *Methanolobus bombayensis* | AN | H | 13 | 21.9 | 36.8 | 39.9 | [491] |
| 991 | *Halonatronum saccharophilum* | AN | H | 9 | 18.0 | 46.8 | 60.2 | [492] |
| 992 | *Acetobacterium bakii* | AN | A | 6 | 1.1 | 19.9 | 24.9 | [493] |
| 993 | *Acetobacterium fimetarium* | AN | A | 7 | 1.1 | 29.8 | 29.8 | [493] |
| 994 | *Acetobacterium paludosum* | AN | A | 6 | 1.1 | 19.9 | 24.8 | [493] |
| 995 | *Natroniella acetigena* | AN | H | 5 | 27.9 | 37.0 | 40.0 | [494] |
| 996 | *Psychromonas antarcticus* | FA | H | 6 | 1.9 | 12.1 | 17.0 | [495] |
| 997 | *Methanosarcina lacustris* | AN | H | 8 | 1.1 | 25.2 | 35.2 | [496] |

(Table S2 continued.)

| Code | Strain/species name | Aero. | Troph. | Smp. | $T_{\min}$ | $T_{\text{opt}}$ | $T_{\max}$ | Lit. |
|---|---|---|---|---|---|---|---|---|
| 998 | *Thiobacillus hydrothermalis* | A | A | 8 | 11.1 | 35.0 | 45.2 | [497] |
| 999 | *Amphibacillus fermentum* | FA | H | 8 | 18.1 | 36.2 | 55.4 | [498] |
| 1000 | *Amphibacillus tropicus* | FA | H | 8 | 17.8 | 38.2 | 55.2 | [498] |
| 1001 | *Methanolobus psychrophilus* | AN | H | 5 | 0.1 | 18.0 | 20.0 | [499] |
| 1002 | *Methanomicrobium paynteri* | AN | H | 6 | 25.0 | 39.9 | 41.9 | [500] |
| 1003 | *Anoxynatronum sibiricum* | AN | H | 5 | 25.0 | 35.1 | 41.1 | [501] |
| 1004 | *Methanogenium marinum* | AN | H | 5 | 10.0 | 24.8 | 24.9 | [502] |
| 1005 | *Clostridium vincentii* | AN | H | 5 | 2.1 | 12.2 | 16.9 | [503] |
| 1006 | *Methylocystis echinoides* | FA | H | 4 | 10.0 | 10.0 | 29.9 | [504] |
| 1007 | *Methylocystis methanolicus* | FA | H | 3 | 15.1 | 30.0 | 30.0 | [504] |
| 1008 | *Methylocystis minimus* | FA | H | 3 | 15.0 | 30.0 | 30.0 | [504] |
| 1009 | *Methylocystis parvus* | FA | H | 3 | 15.1 | 30.0 | 30.0 | [504] |
| 1010 | *Methylocystis pyriformis* | FA | H | 4 | 10.0 | 10.0 | 30.0 | [504] |
| 1011 | *Methylosinus sporium* | FA | H | 3 | 15.0 | 30.0 | 30.0 | [504] |
| 1012 | *Methylosinus trichosporium* | FA | H | 3 | 15.0 | 30.0 | 30.0 | [504] |
| 1013 | *Methylobacter bovis* | FA | H | 4 | 10.0 | 20.1 | 30.1 | [504] |
| 1014 | *Methylobacter chroococcum* | FA | H | 4 | 10.0 | 14.9 | 29.9 | [504] |
| 1015 | *Methylobacter vinelandii* | FA | H | 4 | 10.1 | 20.1 | 30.2 | [504] |
| 1016 | *Methylococcus capsulatus* | FA | H | 3 | 15.0 | 30.1 | 30.1 | [504] |
| 1017 | *Methylomonas methanica* | FA | H | 4 | 10.1 | 20.1 | 30.0 | [504] |
| 1018 | *Staphylothermus marinus* | AN | H | 9 | 64.9 | 92.1 | 98.1 | [505] |
| 1019 | *Staphylothermus marinus* | AN | H | 4 | 79.8 | 84.8 | 91.8 | [505] |
| 1020 | *Clostridium perfringens* | AN | H | 7 | 26.0 | 45.0 | 51.0 | [506] |
| 1021 | *Clostridium perfringens* | AN | H | 7 | 26.0 | 41.0 | 51.0 | [506] |
| 1022 | *Clostridium perfringens* | AN | H | 5 | 33.0 | 41.0 | 49.0 | [506] |

(Table S2 continued.)

| Code | Strain/species name | Aero. | Troph. | Smp. | $T_{\text{min}}$ | $T_{\text{opt}}$ | $T_{\text{max}}$ | Lit. |
|---|---|---|---|---|---|---|---|---|
| 1023 | *Clostridium perfringens* | AN | H | 5 | 33.0 | 41.0 | 49.0 | [506] |
| 1024 | *Archaebacterial str.* | AN | H | 8 | 54.1 | 81.9 | 91.1 | [507] |
| 1025 | *Clostridium aciditolerans* | AN | H | 1 | 37.0 | 37.0 | 37.0 | [508] |
| 1026 | *Acetobacterium tundrae* | AN | A | 8 | 1.0 | 20.1 | 30.0 | [509] |
| 1027 | *Methanobacterium beijingense* | AN | H | 1 | 37.0 | 37.0 | 37.0 | [510] |
| 1028 | *Flavobacterium limicola* | A | H | 5 | 4.9 | 19.9 | 25.0 | [511] |
| 1029 | *Shewanella donghaensis* | FA | H | 8 | 5.0 | 17.1 | 20.0 | [512] |
| 1030 | *Thermosphaera aggregans* | AN | H | 6 | 74.9 | 85.0 | 90.1 | [513] |
| 1031 | *Alkaliphilus transvaalensis* | AN | H | 9 | 20.0 | 40.0 | 50.3 | [514] |
| 1032 | *Desulfobacter psychrotolerans* | AN | H | 12 | -3.7 | 20.2 | 26.3 | [515] |
| 1033 | *Spirochaeta africana* | AN | H | 7 | 15.0 | 30.1 | 44.0 | [516] |
| 1034 | *Spirochaeta alkalica* | AN | H | 8 | 15.0 | 37.0 | 44.1 | [516] |
| 1035 | *Spirochaeta asiatica* | AN | H | 6 | 22.0 | 36.9 | 40.0 | [516] |
| 1036 | *Trichococcus patagoniensis* | FA | H | 8 | 5.1 | 30.0 | 35.3 | [517] |
| 1037 | *Stichococcus bacillaris* | A | A | 7 | 4.3 | 25.1 | 35.0 | [518] |
| 1038 | *Stichococcus bacillaris* | A | A | 5 | 4.0 | 25.0 | 25.0 | [518] |
| 1039 | *Stichococcus minutus* | A | A | 4 | 10.0 | 25.0 | 25.0 | [518] |
| 1040 | *Marinitoga piezophila* | AN | H | 5 | 49.9 | 59.9 | 70.0 | [519] |
| 1041 | *Marinitoga piezophila* | AN | H | 6 | 45.0 | 64.9 | 69.9 | [519] |
| 1042 | *Marinitoga piezophila* | AN | H | 6 | 45.0 | 64.7 | 70.1 | [519] |
| 1043 | *Methanococcus vulcanius* | AN | A | 10 | 50.0 | 79.6 | 89.2 | [520] |
| 1044 | *Hydrogenobacter acidophilus* | A | A | 4 | 49.8 | 64.8 | 70.0 | [521] |
| 1045 | *Caldivirga maquilingensis* | FA | H | 4 | 75.0 | 85.0 | 90.0 | [522] |
| 1046 | *Thermococcus hydrothermalis* | AN | H | 11 | 55.0 | 79.9 | 105.3 | [523] |
| 1047 | *Vibrio diabolicus* | FA | H | 7 | 15.1 | 40.1 | 45.0 | [524] |

(Table S2 continued.)

| Code | Strain/species name | Aero. | Troph. | Smp. | $T_{\mathrm{min}}$ | $T_{\mathrm{opt}}$ | $T_{\mathrm{max}}$ | Lit. |
|---|---|---|---|---|---|---|---|---|
| 1048 | *Brachionus patulus* | A | H | 9 | 15.0 | 35.0 | 35.0 | [525] |
| 1049 | *Keratella cochlearis* | A | H | 5 | 5.0 | 25.0 | 25.0 | [526] |
| 1050 | *Brachionus plicatilis* | A | H | 4 | 10.0 | 20.0 | 20.0 | [527] |
| 1051 | *Psychroflexus torquis* | A | H | 22 | 0.9 | 11.3 | 19.2 | [528] |
| 1052 | *Rhodoglobus vestalii* | A | H | 6 | 1.9 | 17.9 | 22.0 | [529] |
| 1053 | *Clostridium gasigenes* | AN | H | 21 | -0.5 | 22.0 | 25.9 | [530] |
| 1054 | *Palaeococcus pacificus* | AN | H | 1 | 80.0 | 80.0 | 80.0 | [531] |
| 1055 | *Methanosarcina barkeri* | AN | H | 8 | 19.9 | 45.3 | 55.1 | [532] |
| 1056 | *Methanosarcina barkeri* | AN | H | 5 | 19.6 | 31.0 | 40.1 | [532] |
| 1057 | *Methanosarcina barkeri* | AN | H | 5 | 24.7 | 40.9 | 44.9 | [532] |
| 1058 | *Methanosarcina barkeri* | AN | H | 4 | 19.9 | 36.9 | 36.9 | [532] |
| 1059 | *Methanosarcina barkeri* | AN | H | 6 | 24.9 | 41.9 | 45.0 | [532] |
| 1060 | *Methanosarcina barkeri* | AN | H | 6 | 25.0 | 42.1 | 46.2 | [532] |
| 1061 | *Methanosarcina barkeri* | AN | H | 7 | 20.0 | 40.1 | 44.9 | [532] |
| 1062 | *Methanosarcina mazel* | AN | H | 5 | 24.9 | 41.9 | 44.9 | [532] |
| 1063 | *Methanosarcina mazel* | AN | H | 6 | 20.0 | 42.0 | 45.3 | [532] |
| 1064 | *Methanosarcina mazel* | AN | H | 7 | 20.0 | 39.9 | 45.2 | [532] |
| 1065 | *Methanosarcina vacuolata* | AN | H | 5 | 20.1 | 42.1 | 42.1 | [532] |
| 1066 | *Methanosarcina vacuolata* | AN | H | 4 | 24.9 | 36.9 | 40.9 | [532] |
| 1067 | *Methanosarcina vacuolata* | AN | H | 4 | 24.8 | 36.9 | 39.7 | [532] |
| 1068 | *Methanosarcina vacuolata* | AN | H | 5 | 19.8 | 39.7 | 39.7 | [532] |
| 1069 | *Methanobacterium kanagiense* | AN | A | 6 | 15.0 | 39.9 | 44.9 | [533] |
| 1070 | *Methanococcus infernus* | AN | A | 8 | 55.3 | 84.9 | 90.1 | [534] |
| 1071 | *Methanoculleus marisnigri* | AN | H | 7 | 25.1 | 40.8 | 55.1 | [535] |
| 1072 | *Methanoculleus marisnigri* | AN | H | 6 | 25.0 | 39.8 | 45.0 | [535] |

(Table S2 continued.)

| Code | Strain/species name | Aero. | Troph. | Smp. | $T_{\mathrm{min}}$ | $T_{\mathrm{opt}}$ | $T_{\mathrm{max}}$ | Lit. |
|---|---|---|---|---|---|---|---|---|
| 1073 | *Methanogenium cariaci* | AN | H | 7 | 19.7 | 45.1 | 45.1 | [535] |
| 1074 | *Rubrobacter radiotolerans* | A | H | 6 | 29.9 | 44.8 | 54.9 | [536] |
| 1075 | *Rubrobacter taiwanensis* | A | H | 10 | 25.2 | 55.2 | 70.2 | [536] |
| 1076 | *Rubrobacter taiwanensis* | A | H | 8 | 30.0 | 60.2 | 70.2 | [536] |
| 1077 | *Rubrobacter xylanophilus* | A | H | 9 | 30.0 | 60.3 | 70.3 | [536] |
| 1078 | *Thiobacillus tepidarius* | A | A | 7 | 35.0 | 45.0 | 52.0 | [537] |
| 1079 | *Thermoanaerobacter sulfurigignens* | AN | H | 17 | 32.1 | 63.1 | 71.9 | [538] |
| 1080 | *Thermoanaerobacter uzonensis* | AN | H | 18 | 30.1 | 61.1 | 67.0 | [538] |
| 1081 | *Alicyclobacillus acidocaldarius* | FA | H | 9 | 40.0 | 64.9 | 70.1 | [539] |
| 1082 | *Alicyclobacillus acidoterrestris* | FA | H | 7 | 35.1 | 50.0 | 60.2 | [539] |
| 1083 | *Alicyclobacillus hesperidum* | FA | H | 5 | 39.9 | 52.4 | 54.6 | [539] |
| 1084 | *Alicyclobacillus sp.* | FA | H | 8 | 39.9 | 62.4 | 67.5 | [539] |
| 1085 | *Thiobacter subterraneus* | microA | A | 7 | 34.9 | 54.8 | 62.1 | [540] |
| 1086 | *Lactobacillus thermotolerans* | FA | H | 10 | 19.6 | 42.0 | 49.9 | [541] |
| 1087 | *Hydrogenivirga caldilitoris* | A | A | 6 | 54.9 | 75.2 | 77.8 | [542] |
| 1088 | *Thermovibrio ammonificans* | AN | A | 5 | 60.0 | 74.8 | 80.1 | [543] |
| 1089 | *Methanothermococcus okinawensis* | AN | A | 7 | 39.7 | 62.6 | 75.0 | [544] |
| 1090 | *Thermodesulfobacterium hydrogeniphilum* | AN | A | 7 | 50.0 | 74.5 | 79.8 | [545] |
| 1091 | *Thermocladium modestius* | FA | H | 5 | 60.1 | 75.0 | 80.1 | [546] |
| 1092 | *Desulfotomaculum putei* | AN | H | 10 | 40.0 | 60.3 | 60.3 | [547] |
| 1093 | *Bacillus infernus* | AN | H | 4 | 45.1 | 59.6 | 59.6 | [548] |
| 1094 | *Sulfurihydrogenibium yellowstonense* | A | H | 6 | 55.0 | 70.1 | 78.0 | [549] |
| 1095 | *Flavobacterium hibernum* | A | H | 28 | 2.9 | 24.9 | 28.8 | [550] |
| 1096 | *Thioreductor micantisoli* | AN | A | 6 | 19.9 | 32.1 | 44.9 | [551] |
| 1097 | *Methanococcus aeolicus* | AN | A | 16 | 9.8 | 44.7 | 49.9 | [552] |

(Table S2 continued.)

| Code | Strain/species name | Aero. | Troph. | Smp. | $T_{\mathrm{min}}$ | $T_{\mathrm{opt}}$ | $T_{\mathrm{max}}$ | Lit. |
|---|---|---|---|---|---|---|---|---|
| 1098 | *Aeropyrum pernix* | A | H | 8 | 70.1 | 90.3 | 100.0 | [553] |
| 1099 | *Balnearium lithotrophicum* | AN | A | 7 | 45.2 | 75.4 | 80.3 | [554] |
| 1100 | *Aeropyrum camini* | A | H | 7 | 70.1 | 84.9 | 97.0 | [555] |
| 1101 | *Selenomonas lipolytica* | AN | H | 5 | 29.9 | 40.0 | 46.9 | [556] |
| 1102 | *Nisaea denitrificans* | FA | H | 6 | 15.1 | 30.0 | 44.0 | [557] |
| 1103 | *Daphnia magna* | A | H | 20 | 14.0 | 29.0 | 29.0 | [558] |
| 1104 | *Spirochaeta sphaeroplastigenens* | AN | H | 1 | 37.0 | 37.0 | 37.0 | [559] |
| 1105 | *Thermothrix thioparus* | FA | M | 5 | 61.7 | 69.8 | 76.6 | [560] |
| 1106 | *Thermoanaerobacter ethanolicus* | AN | H | 14 | 37.3 | 69.8 | 79.2 | [561] |
| 1107 | *Halomonas elongata* | FA | H | 2 | 20.0 | 40.0 | 40.0 | [562] |
| 1108 | *Halomonas elongata* | FA | H | 2 | 20.0 | 40.0 | 40.0 | [562] |
| 1109 | *Halomonas elongata* | FA | H | 1 | 30.0 | 30.0 | 30.0 | [562] |
| 1110 | *Halomonas elongata* | FA | H | 3 | 20.0 | 40.0 | 40.0 | [562] |
| 1111 | *Halomonas elongata* | FA | H | 3 | 20.0 | 40.0 | 40.0 | [562] |
| 1112 | *Halomonas elongata* | FA | H | 3 | 20.0 | 30.0 | 40.0 | [562] |
| 1113 | *Halomonas elongata* | FA | H | 2 | 30.0 | 40.0 | 40.0 | [562] |
| 1114 | *Halomonas elongata* | FA | H | 2 | 20.0 | 30.0 | 30.0 | [562] |
| 1115 | *Halomonas elongata* | FA | H | 2 | 20.0 | 30.0 | 30.0 | [562] |
| 1116 | *Halomonas elongata* | FA | H | 3 | 20.0 | 30.0 | 40.0 | [562] |
| 1117 | *Halomonas elongata* | FA | H | 3 | 20.0 | 30.0 | 40.0 | [562] |
| 1118 | *Halomonas elongata* | FA | H | 1 | 30.0 | 30.0 | 30.0 | [562] |
| 1119 | *Halomonas elongata* | FA | H | 2 | 20.0 | 30.0 | 30.0 | [562] |
| 1120 | *Halomonas elongata* | FA | H | 2 | 20.0 | 30.0 | 30.0 | [562] |
| 1121 | *Halomonas elongata* | FA | H | 3 | 20.0 | 30.0 | 40.0 | [562] |
| 1122 | *Halomonas elongata* | FA | H | 3 | 20.0 | 30.0 | 40.0 | [562] |

(Table S2 continued.)

| Code | Strain/species name | Aero. | Troph. | Smp. | $T_{min}$ | $T_{opt}$ | $T_{max}$ | Lit. |
|---|---|---|---|---|---|---|---|---|
| 1123 | *Halomonas elongata* | FA | H | 1 | 30.0 | 30.0 | 30.0 | [562] |
| 1124 | *Asterionella formosa* | A | A | 8 | 2.0 | 17.0 | 25.0 | [563] |
| 1125 | *Staurastrum cingulum* | A | A | 9 | 2.0 | 30.0 | 30.0 | [563] |
| 1126 | *Tychonema bourrellyi* | A | A | 6 | 8.0 | 20.0 | 25.0 | [563] |
| 1127 | *Cryptomonas marssonii* | A | A | 8 | 2.0 | 11.0 | 25.0 | [563] |
| 1128 | *Dinobryon divergens* | A | A | 8 | 2.0 | 17.0 | 25.0 | [563] |
| 1129 | *Ceratium furcoides* | A | A | 5 | 11.0 | 20.0 | 25.0 | [563] |
| 1130 | *Nannochloropsis oceanica* | A | A | 7 | 14.5 | 29.0 | 32.3 | [564] |
| 1131 | *Porphyridium purpureum* | A | A | 7 | 5.0 | 20.1 | 35.0 | [565] |
| 1132 | *Scenedesmus sp.* | A | A | 4 | 10.0 | 25.0 | 30.0 | [566] |
| 1133 | *Phaeodactylum tricornutum* | A | A | 4 | 5.0 | 19.9 | 19.9 | [567] |
| 1134 | *Phaeodactylum tricornutum* | A | A | 6 | 4.8 | 19.9 | 30.0 | [567] |
| 1135 | *Deinococcus geothermalis* | A | H | 7 | 30.0 | 50.1 | 57.2 | [568] |
| 1136 | *Deinococcus murrayi* | A | H | 6 | 29.9 | 50.1 | 52.5 | [568] |
| 1137 | *Escherichia coli* | FA | H | 11 | 9.8 | 36.5 | 44.7 | [569] |
| 1138 | *Escherichia coli* | FA | H | 10 | 10.0 | 34.8 | 42.7 | [569] |
| 1139 | *Salmonella enterica* | FA | H | 9 | 9.9 | 37.0 | 43.1 | [569] |
| 1140 | *Salmonella enterica* | FA | H | 10 | 9.8 | 35.2 | 42.9 | [569] |
| 1141 | *Marinithermus hydrothermalis* | A | H | 8 | 44.9 | 67.6 | 72.6 | [570] |
| 1142 | *Fervidobacterium pennivorans* | AN | H | 7 | 40.3 | 69.9 | 80.3 | [571] |
| 1143 | *Thermotoga maritima* | AN | H | 4 | 74.9 | 80.0 | 86.0 | [572] |
| 1144 | *Methanosaeta harundinacea* | AN | H | 1 | 37.0 | 37.0 | 37.0 | [573] |
| 1145 | *Geotoga petraea* | AN | H | 6 | 30.0 | 50.0 | 55.0 | [574] |
| 1146 | *Geotoga subterranea* | AN | H | 7 | 30.2 | 45.0 | 59.9 | [574] |
| 1147 | *Petrotoga miotherma* | AN | H | 7 | 35.0 | 55.0 | 65.0 | [574] |

(Table S2 continued.)

| Code | Strain/species name | Aero. | Troph. | Smp. | $T_{\mathrm{min}}$ | $T_{\mathrm{opt}}$ | $T_{\mathrm{max}}$ | Lit. |
|---|---|---|---|---|---|---|---|---|
| 1148 | *Escherichia coli* | FA | H | 2 | 23.0 | 37.0 | 37.0 | [575] |
| 1149 | *Escherichia coli* | FA | H | 2 | 23.0 | 37.0 | 37.0 | [575] |
| 1150 | *Escherichia coli* | FA | H | 2 | 37.0 | 37.0 | 37.0 | [575] |
| 1151 | *Escherichia coli* | FA | H | 1 | 37.0 | 37.0 | 37.0 | [575] |
| 1152 | *Escherichia coli* | FA | H | 1 | 23.0 | 23.0 | 23.0 | [575] |
| 1153 | *Bacillus subtilis* | FA | H | 2 | 23.0 | 37.0 | 37.0 | [575] |
| 1154 | *Bacillus subtilis* | FA | H | 2 | 23.0 | 37.0 | 37.0 | [575] |
| 1155 | *Bacillus subtilis* | FA | H | 1 | 23.0 | 23.0 | 23.0 | [575] |
| 1156 | *Bacillus subtilis* | FA | H | 2 | 23.0 | 37.0 | 37.0 | [575] |
| 1157 | *Bacillus subtilis* | FA | H | 1 | 23.0 | 23.0 | 23.0 | [575] |
| 1158 | *Thermosipho africanus* | AN | H | 7 | 50.0 | 75.1 | 77.1 | [576] |
| 1159 | *Uncinula necator* | A | H | 4 | 19.0 | 26.0 | 30.0 | [577] |
| 1160 | *Caldicellulosiruptor owensensis* | AN | H | 7 | 49.8 | 75.0 | 80.1 | [578] |
| 1161 | *Caldicellulosiruptor obsidiansis* | AN | H | 6 | 64.9 | 77.7 | 83.9 | [579] |
| 1162 | *Dictyoglomus sp.* | AN | H | 5 | 54.9 | 67.8 | 75.0 | [580] |
| 1163 | *Desulfotomaculum alkaliphilum* | AN | H | 6 | 29.4 | 50.0 | 59.4 | [581] |
| 1164 | *Echinogammarus marinus* | A | H | 3 | 10.0 | 20.0 | 20.0 | [582] |
| 1165 | *Echinogammarus marinus* | A | H | 3 | 10.0 | 20.0 | 20.0 | [582] |
| 1166 | *Echinogammarus marinus* | A | H | 3 | 10.0 | 20.0 | 20.0 | [582] |
| 1167 | *Echinogammarus marinus* | A | H | 3 | 10.0 | 20.0 | 20.0 | [582] |
| 1168 | *Hyalella azteca* | A | H | 3 | 15.0 | 25.0 | 25.0 | [583] |
| 1169 | *Hyalella azteca* | A | H | 5 | 20.0 | 20.0 | 20.0 | [583] |
| 1170 | *Geobacillus sp.* | FA | H | 5 | 45.0 | 70.1 | 73.1 | [584] |
| 1171 | *Geobacillus sp.* | FA | H | 5 | 45.1 | 73.0 | 73.0 | [584] |
| 1172 | *Geobacillus sp.* | FA | H | 5 | 45.0 | 70.1 | 73.1 | [584] |

(Table S2 continued.)

| Code | Strain/species name | Aero. | Troph. | Smp. | $T_{\mathrm{min}}$ | $T_{\mathrm{opt}}$ | $T_{\mathrm{max}}$ | Lit. |
|---|---|---|---|---|---|---|---|---|
| 1173 | *Bacillus sp.* | A | H | 4 | 55.1 | 65.1 | 70.0 | [585] |
| 1174 | *Metallosphaera prunae* | A | M | 6 | 55.0 | 74.9 | 79.9 | [586] |
| 1175 | *Metallosphaera sedula* | A | M | 7 | 50.3 | 75.2 | 80.2 | [587] |
| 1176 | *Thermoanaerobacterium aotearoense* | AN | H | 14 | 32.2 | 62.6 | 65.8 | [588] |
| 1177 | *Thalassiosira pseudonana* | A | A | 3 | 15.7 | 23.8 | 23.8 | [589] |
| 1178 | *Thalassiosira pseudonana* | A | A | 3 | 15.8 | 23.8 | 23.8 | [589] |
| 1179 | *Thalassiosira pseudonana* | A | A | 4 | 11.9 | 23.8 | 23.8 | [589] |
| 1180 | *Thalassiosira pseudonana* | A | A | 3 | 15.8 | 24.0 | 24.0 | [589] |
| 1181 | *Thalassiosira pseudonana* | A | A | 4 | 11.9 | 24.0 | 24.0 | [589] |
| 1182 | *Thalassiosira pseudonana* | A | A | 4 | 12.1 | 24.2 | 24.2 | [589] |
| 1183 | *Thalassiosira pseudonana* | A | A | 4 | 11.8 | 23.9 | 23.9 | [589] |
| 1184 | *Thalassiosira pseudonana* | A | A | 4 | 12.1 | 24.2 | 24.2 | [589] |
| 1185 | *Thalassiosira pseudonana* | A | A | 4 | 12.0 | 24.2 | 24.2 | [589] |
| 1186 | *Thalassiosira pseudonana* | A | A | 3 | 16.0 | 23.9 | 23.9 | [589] |
| 1187 | *Thalassiosira pseudonana* | A | A | 4 | 12.0 | 24.0 | 24.0 | [589] |
| 1188 | *Thalassiosira pseudonana* | A | A | 4 | 12.1 | 24.0 | 24.0 | [589] |
| 1189 | *Thalassiosira pseudonana* | A | A | 3 | 16.1 | 24.0 | 24.0 | [589] |
| 1190 | *Thalassiosira pseudonana* | A | A | 4 | 11.9 | 23.9 | 23.9 | [589] |
| 1191 | *Thalassiosira nordenskioldii* | A | A | 4 | 0.0 | 5.0 | 15.0 | [590] |
| 1192 | *Skeletonema costatum* | A | A | 7 | 5.0 | 25.0 | 25.0 | [591] |
| 1193 | *Olisthodiscus luteus* | A | A | 6 | 10.0 | 25.0 | 25.0 | [591] |
| 1194 | *Gonyaulax tamarensis* | A | A | 6 | 5.0 | 15.0 | 20.0 | [591] |
| 1195 | *Thalassiosira rotula* | A | A | 2 | 15.3 | 15.3 | 20.1 | [592] |
| 1196 | *Thalassiosira rotula* | A | A | 3 | 10.1 | 20.3 | 20.3 | [592] |
| 1197 | *Thalassiosira rotula* | A | A | 4 | 10.2 | 19.9 | 25.5 | [592] |

(Table S2 continued.)

| Code | Strain/species name | Aero. | Troph. | Smp. | $T_{min}$ | $T_{opt}$ | $T_{max}$ | Lit. |
|---|---|---|---|---|---|---|---|---|
| 1198 | *Thalassiosira rotula* | A | A | 5 | 5.6 | 19.9 | 25.1 | [592] |
| 1199 | *Thalassiosira rotula* | A | A | 5 | 5.4 | 25.7 | 25.7 | [592] |
| 1200 | *Thalassiosira rotula* | A | A | 6 | 0.4 | 20.5 | 25.3 | [592] |
| 1201 | *Thalassiosira rotula* | A | A | 5 | 6.4 | 26.6 | 26.6 | [592] |
| 1202 | *Thalassiosira rotula* | A | A | 6 | 1.3 | 26.4 | 26.4 | [592] |
| 1203 | *Thalassiosira rotula* | A | A | 6 | 5.2 | 25.1 | 30.4 | [592] |
| 1204 | *Thalassiosira rotula* | A | A | 6 | 0.2 | 25.0 | 25.0 | [592] |
| 1205 | *Thalassiosira rotula* | A | A | 6 | 5.6 | 26.6 | 31.0 | [592] |
| 1206 | *Thalassiosira rotula* | A | A | 6 | 1.5 | 20.9 | 25.3 | [592] |
| 1207 | *Thalassiosira rotula* | A | A | 6 | 4.9 | 19.8 | 30.2 | [592] |
| 1208 | *Thalassiosira rotula* | A | A | 5 | 0.0 | 19.8 | 19.8 | [592] |
| 1209 | *Detonula confervacea* | A | A | 3 | 2.0 | 12.0 | 12.0 | [593] |
| 1210 | *Detonula confervacea* | A | A | 3 | 2.0 | 7.0 | 12.0 | [593] |
| 1211 | *Detonula confervacea* | A | A | 3 | 2.0 | 12.0 | 12.0 | [593] |
| 1212 | *Detonula confervacea* | A | A | 3 | 2.0 | 7.0 | 12.0 | [593] |
| 1213 | *Detonula confervacea* | A | A | 3 | 2.0 | 12.0 | 12.0 | [593] |
| 1214 | *Detonula confervacea* | A | A | 3 | 2.0 | 7.0 | 12.0 | [593] |
| 1215 | *Detonula confervacea* | A | A | 3 | 2.0 | 7.0 | 12.0 | [593] |
| 1216 | *Detonula confervacea* | A | A | 1 | 2.0 | 2.0 | 2.0 | [593] |
| 1217 | *Detonula confervacea* | A | A | 3 | 2.0 | 7.0 | 12.0 | [593] |
| 1218 | *Detonula confervacea* | A | A | 3 | 2.0 | 12.0 | 12.0 | [593] |
| 1219 | *Detonula confervacea* | A | A | 3 | 2.0 | 12.0 | 12.0 | [593] |
| 1220 | *Detonula confervacea* | A | A | 3 | 2.0 | 7.0 | 12.0 | [593] |
| 1221 | *Detonula confervacea* | A | A | 1 | 2.0 | 2.0 | 2.0 | [593] |
| 1222 | *Detonula confervacea* | A | A | 3 | 2.0 | 7.0 | 12.0 | [593] |

(Table S2 continued.)

| Code | Strain/species name | Aero. | Troph. | Smp. | $T_{\mathrm{min}}$ | $T_{\mathrm{opt}}$ | $T_{\mathrm{max}}$ | Lit. |
|---|---|---|---|---|---|---|---|---|
| 1223 | *Detonula confervacea* | A | A | 3 | 2.0 | 7.0 | 12.0 | [593] |
| 1224 | *Detonula confervacea* | A | A | 3 | 2.0 | 12.0 | 12.0 | [593] |
| 1225 | *Detonula confervacea* | A | A | 3 | 2.0 | 7.0 | 12.0 | [593] |
| 1226 | *Detonula confervacea* | A | A | 1 | 2.0 | 2.0 | 2.0 | [593] |
| 1227 | *Detonula confervacea* | A | A | 3 | 2.0 | 7.0 | 12.0 | [593] |
| 1228 | *Detonula confervacea* | A | A | 3 | 2.0 | 12.0 | 12.0 | [593] |
| 1229 | *Detonula confervacea* | A | A | 3 | 2.0 | 7.0 | 12.0 | [593] |
| 1230 | *Detonula confervacea* | A | A | 2 | 2.0 | 2.0 | 7.0 | [593] |
| 1231 | *Detonula confervacea* | A | A | 1 | 2.0 | 2.0 | 2.0 | [593] |
| 1232 | *Detonula confervacea* | A | A | 3 | 2.0 | 7.0 | 12.0 | [593] |
| 1233 | *Detonula confervacea* | A | A | 2 | 2.0 | 7.0 | 7.0 | [593] |
| 1234 | *Detonula confervacea* | A | A | 3 | 2.0 | 7.0 | 12.0 | [593] |
| 1235 | *Detonula confervacea* | A | A | 2 | 2.0 | 7.0 | 7.0 | [593] |
| 1236 | *Detonula confervacea* | A | A | 1 | 2.0 | 2.0 | 2.0 | [593] |
| 1237 | *Detonula confervacea* | A | A | 3 | 2.0 | 7.0 | 12.0 | [593] |
| 1238 | *Detonula confervacea* | A | A | 2 | 2.0 | 7.0 | 7.0 | [593] |
| 1239 | *Detonula confervacea* | A | A | 1 | 2.0 | 2.0 | 2.0 | [593] |
| 1240 | *Detonula confervacea* | A | A | 2 | 2.0 | 2.0 | 7.0 | [593] |
| 1241 | *Detonula confervacea* | A | A | 2 | 2.0 | 7.0 | 7.0 | [593] |
| 1242 | *Leptocylindrus danicus* | A | A | 4 | 5.0 | 19.8 | 19.8 | [594] |
| 1243 | *Skeletonema costatum* | A | A | 9 | 0.0 | 22.0 | 22.0 | [595] |
| 1244 | *Sulfophobococcus zilligii* | AN | H | 5 | 70.1 | 90.3 | 94.7 | [596] |
| 1245 | *Thermobaculum terrenum* | A | H | 9 | 42.9 | 66.8 | 74.9 | [597] |
| 1246 | *Bacillus beveridgei* | FA | H | 10 | 4.8 | 38.2 | 64.9 | [598] |
| 1247 | *Bellilinea caldifistulae* | AN | H | 1 | 55.0 | 55.0 | 55.0 | [599] |

(Table S2 continued.)

| Code | Strain/species name | Aero. | Troph. | Smp. | $T_{\mathrm{min}}$ | $T_{\mathrm{opt}}$ | $T_{\mathrm{max}}$ | Lit. |
|---|---|---|---|---|---|---|---|---|
| 1248 | *Longilinea arvoryzae* | AN | H | 1 | 37.0 | 37.0 | 37.0 | [599] |
| 1249 | *Acaryochloris marina* | A | A | 6 | 15.0 | 29.8 | 40.1 | [600] |
| 1250 | *Cyclotella nana* | A | A | 2 | 14.6 | 19.9 | 19.9 | [601] |
| 1251 | *Cyclotella nana* | A | A | 3 | 9.3 | 19.9 | 19.9 | [601] |
| 1252 | *Cyclotella nana* | A | A | 3 | 9.3 | 19.8 | 19.8 | [601] |
| 1253 | *Cyclotella nana* | A | A | 3 | 9.3 | 14.6 | 19.8 | [601] |
| 1254 | *Cyclotella nana* | A | A | 3 | 9.5 | 19.9 | 19.9 | [601] |
| 1255 | *Detonula confervacea* | A | A | 2 | 9.3 | 9.3 | 14.5 | [601] |
| 1256 | *Thermogladius shockii* | AN | H | 4 | 64.8 | 85.0 | 85.0 | [602] |
| 1257 | *Methanothermobacter crinale* | AN | A | 6 | 55.0 | 64.8 | 80.1 | [603] |
| 1258 | *Pseudomonas thermotolerans* | A | H | 6 | 25.1 | 40.3 | 50.3 | [604] |
| 1259 | *Thermomonas haemolytica* | A | H | 7 | 25.0 | 45.2 | 55.2 | [604] |
| 1260 | *Thermomonas hydrothermalis* | A | H | 11 | 29.9 | 50.1 | 62.5 | [604] |
| 1261 | *Pyrobaculum oguniense* | FA | H | 7 | 70.5 | 90.7 | 97.5 | [605] |
| 1262 | *Sulfolobus hakonensis* | A | M | 5 | 55.2 | 70.1 | 75.1 | [606] |
| 1263 | *Thermothrix azorensis* | A | A | 5 | 65.0 | 78.0 | 86.0 | [607] |
| 1264 | *Hydrogenophilus hirschii* | AN | A | 7 | 50.0 | 62.5 | 67.4 | [608] |
| 1265 | *Stygiolobus azoricus* | AN | A | 9 | 59.9 | 80.4 | 88.6 | [609] |
| 1266 | *Methanobacterium Bryantii* | AN | H | 7 | 10.0 | 28.2 | 45.9 | [610] |
| 1267 | *Methanobacterium veterum* | AN | H | 6 | 20.2 | 37.1 | 50.2 | [610] |
| 1268 | *Thiomicrospira thermophila* | microA | M | 8 | 15.0 | 40.0 | 55.0 | [611] |
| 1269 | *Sulfolobus yangmingensis* | A | M | 7 | 65.0 | 79.8 | 94.9 | [612] |
| 1270 | *Methanopyrus kandleri* | AN | A | 5 | 85.3 | 100.1 | 110.2 | [613] |
| 1271 | *Methanocorpusculum bavaricum* | AN | H | 4 | 13.9 | 37.3 | 37.3 | [614] |
| 1272 | *Methanocorpusculum sinsese* | AN | H | 5 | 21.4 | 29.7 | 40.2 | [614] |

(Table S2 continued.)

| Code | Strain/species name | Aero. | Troph. | Smp. | $T_{\min}$ | $T_{\mathrm{opt}}$ | $T_{\max}$ | Lit. |
|---|---|---|---|---|---|---|---|---|
| 1273 | *Thiobacillus prosperus* | A | A | 5 | 25.1 | 37.0 | 41.0 | [615] |
| 1274 | *Thiobacillus prosperus* | A | A | 6 | 23.1 | 32.8 | 41.1 | [615] |
| 1275 | *Thiobacillus prosperus* | A | A | 4 | 23.1 | 36.9 | 36.9 | [615] |
| 1276 | *Methanobacterium thermoaggregans* | AN | A | 5 | 45.3 | 65.5 | 70.6 | [616] |
| 1277 | *Acidilobus aceticus* | AN | H | 5 | 74.9 | 84.9 | 89.0 | [617] |
| 1278 | *Sulfolobus tengchongensis* | A | H | 7 | 65.0 | 85.2 | 95.0 | [618] |
| 1279 | *Magnetospira thiophila* | microA | M | 1 | 25.0 | 25.0 | 25.0 | [619] |
| 1280 | *Stetteria hydrogenophila* | AN | M | 9 | 80.0 | 95.2 | 102.0 | [620] |
| 1281 | *Methanobacterium thermoautotrophicum* | AN | A | 6 | 65.5 | 80.1 | 90.6 | [621] |
| 1282 | *Methanobacterium thermoautotrophicum* | AN | A | 1 | 60.8 | 60.8 | 60.8 | [621] |
| 1283 | *Methanothermus fervidus* | AN | A | 6 | 50.2 | 65.2 | 75.6 | [621] |
| 1284 | *Sulfolobus metallicus* | A | A | 6 | 49.9 | 70.0 | 75.2 | [622] |
| 1285 | *Sulfolobus metallicus* | A | A | 5 | 49.9 | 65.0 | 70.1 | [622] |
| 1286 | *Acinetobacter calcoaceticus* | A | H | 29 | 16.4 | 32.9 | 39.0 | [623] |
| 1287 | *Ammonifex degensii* | AN | M | 5 | 63.9 | 69.9 | 75.8 | [624] |
| 1288 | *Methanobacterium espanolae* | AN | A | 5 | 20.2 | 35.3 | 40.1 | [625] |
| 1289 | *Clostridium celevecrescens* | AN | H | 4 | 19.8 | 35.3 | 35.3 | [626] |
| 1290 | *Methanohalophilus oregonense* | AN | A | 6 | 20.1 | 35.4 | 41.3 | [627] |
| 1291 | *Thermus brockianus* | A | H | 6 | 50.3 | 65.4 | 75.3 | [628] |
| 1292 | *Thermus igniterrae* | A | H | 7 | 50.3 | 70.3 | 80.1 | [628] |
| 1293 | *Thermus scotoductus* | A | H | 6 | 50.0 | 70.3 | 75.3 | [628] |
| 1294 | *Thermus sp.* | A | H | 6 | 50.2 | 65.1 | 75.4 | [628] |
| 1295 | *Bacillus halodenitrificans* | FA | H | 12 | 10.9 | 37.8 | 44.7 | [629] |
| 1296 | *Methanosarcina semesiae* | AN | A | 7 | 18.1 | 33.0 | 38.9 | [630] |
| 1297 | *Methanosarcina semesiae* | AN | A | 7 | 20.1 | 31.0 | 37.0 | [630] |

(Table S2 continued.)

| Code | Strain/species name | Aero. | Troph. | Smp. | $T_{min}$ | $T_{opt}$ | $T_{max}$ | Lit. |
|---|---|---|---|---|---|---|---|---|
| 1298 | *Methanosarcina semesiae* | AN | A | 7 | 18.0 | 37.2 | 39.2 | [630] |
| 1299 | *Prochlorothrix hollandica* | A | A | 4 | 19.4 | 24.6 | 29.8 | [631] |
| 1300 | *Methanohalophilus portucalensis* | AN | A | 4 | 32.1 | 42.1 | 50.9 | [632] |
| 1301 | *Methanohalophilus portucalensis* | AN | A | 3 | 32.0 | 42.1 | 42.1 | [632] |
| 1302 | *Methanohalophilus portucalensis* | AN | A | 3 | 32.0 | 39.5 | 42.0 | [632] |
| 1303 | *Methanohalophilus portucalensis* | AN | A | 4 | 25.0 | 37.0 | 42.0 | [632] |
| 1304 | *Methanohalophilus portucalensis* | AN | A | 4 | 36.9 | 42.0 | 47.9 | [632] |
| 1305 | *Methanomethylovorans thermophila* | AN | H | 1 | 50.0 | 50.0 | 50.0 | [633] |
| 1306 | *Bacillus infernus* | AN | H | 4 | 45.0 | 60.2 | 60.2 | [634] |
| 1307 | *Thermus chliarophilus* | FA | H | 6 | 35.2 | 50.5 | 60.4 | [635] |
| 1308 | *Thermus ruber* | FA | H | 7 | 35.3 | 60.2 | 65.0 | [635] |
| 1309 | *Thermus silvanus* | FA | H | 6 | 40.2 | 55.5 | 65.6 | [635] |
| 1310 | *Nitratifractor salsuginis* | microA | A | 4 | 28.0 | 36.8 | 36.8 | [636] |
| 1311 | *Nitratiruptor tergarcus* | microA | A | 4 | 40.0 | 55.1 | 55.1 | [636] |
| 1312 | *Sulfobacillus thermotolerans* | FA | M | 9 | 19.7 | 40.1 | 60.1 | [637] |
| 1313 | *Methanocella paludicola* | AN | H | 1 | 36.0 | 36.0 | 36.0 | [638] |
| 1314 | *Methanomicrococcus blatticola* | AN | H | 4 | 23.5 | 39.1 | 39.1 | [639] |
| 1315 | *Anacystis nidulans* | A | A | 8 | 25.0 | 41.3 | 44.2 | [640] |
| 1316 | *Anabaena variabilis* | A | A | 5 | 25.0 | 34.8 | 37.5 | [640] |
| 1317 | *Nostoc muscorum* | A | A | 4 | 25.1 | 32.5 | 34.8 | [640] |
| 1318 | *Methanococcoides methylutens* | AN | H | 5 | 15.0 | 34.8 | 34.8 | [641] |
| 1319 | *Pyrodictium abyssi* | AN | H | 12 | 79.9 | 96.7 | 109.9 | [642] |
| 1320 | *Clostridium thermosuccinogenes* | AN | H | 7 | 42.9 | 71.6 | 71.6 | [643] |
| 1321 | *Clostridium thermosuccinogenes* | AN | H | 4 | 43.0 | 57.9 | 57.9 | [643] |
| 1322 | *Haloanaerobacter chitinovorans* | AN | H | 6 | 24.9 | 45.0 | 50.1 | [644] |

(Table S2 continued.)

| Code | Strain/species name | Aero. | Troph. | Smp. | $T_{min}$ | $T_{opt}$ | $T_{max}$ | Lit. |
|---|---|---|---|---|---|---|---|---|
| 1323 | *Haloanaerobacter chitinovorans* | AN | H | 6 | 25.0 | 45.1 | 50.0 | [644] |
| 1324 | *Geothermobacterium ferrireducens* | AN | A | 8 | 65.0 | 90.0 | 100.0 | [645] |
| 1325 | *Firmicutes sp.* | AN | A | 10 | 50.9 | 65.3 | 70.1 | [646] |
| 1326 | *Firmicutes sp.* | AN | A | 9 | 57.2 | 72.4 | 78.0 | [646] |
| 1327 | *Magnetospirillum bellicus* | FA | H | 5 | 19.8 | 39.9 | 41.9 | [647] |
| 1328 | *Magnetospirillum bellicus* | FA | H | 7 | 10.2 | 41.8 | 41.8 | [647] |
| 1329 | *Methanolobus chelungpuianus* | AN | A | 3 | 23.8 | 37.2 | 37.2 | [648] |
| 1330 | *Methanobacterium palustre* | AN | A | 4 | 21.9 | 37.1 | 45.1 | [648] |
| 1331 | *Profundimonas piezophila* | FA | H | 5 | 4.0 | 8.0 | 14.0 | [649] |
| 1332 | *Caldicellulosiruptor lactoaceticus* | AN | H | 5 | 49.9 | 68.2 | 74.9 | [650] |
| 1333 | *Methanococcus maripaludis* | AN | A | 9 | 18.2 | 37.2 | 45.9 | [651] |
| 1334 | *Desulfobacterium autotrophicum* | AN | A | 19 | -0.5 | 28.0 | 29.5 | [652] |
| 1335 | *Microcella putealis* | A | H | 6 | 15.0 | 30.1 | 40.2 | [653] |
| 1336 | *Microcella putealis* | A | H | 6 | 15.1 | 35.5 | 40.3 | [653] |
| 1337 | *Microcella putealis* | A | H | 6 | 15.1 | 35.3 | 40.2 | [653] |
| 1338 | *Chimaereicella alkaliphila* | A | H | 5 | 14.9 | 29.9 | 34.8 | [654] |
| 1339 | *Chimaereicella alkaliphila* | A | H | 5 | 15.0 | 29.7 | 34.9 | [654] |
| 1340 | *Chimaereicella alkaliphila* | A | H | 5 | 15.0 | 29.8 | 35.0 | [654] |
| 1341 | *Methanospirillum stamsii* | AN | A | 7 | 4.9 | 29.9 | 37.0 | [655] |
| 1342 | *Flavobacterium segetis* | A | H | 5 | 5.3 | 12.9 | 17.8 | [656] |
| 1343 | *Flavobacterium weaverense* | A | H | 5 | 5.3 | 15.6 | 17.9 | [656] |
| 1344 | *Colwellia piezophila* | FA | H | 1 | 4.0 | 4.0 | 4.0 | [657] |
| 1345 | *Colwellia piezophila* | FA | H | 1 | 4.0 | 4.0 | 4.0 | [657] |
| 1346 | *Colwellia piezophila* | FA | H | 2 | 4.0 | 4.0 | 10.0 | [657] |
| 1347 | *Colwellia piezophila* | FA | H | 2 | 4.0 | 4.0 | 10.0 | [657] |

(Table S2 continued.)

| Code | Strain/species name | Aero. | Troph. | Smp. | $T_{\mathrm{min}}$ | $T_{\mathrm{opt}}$ | $T_{\mathrm{max}}$ | Lit. |
|---|---|---|---|---|---|---|---|---|
| 1348 | *Colwellia piezophila* | FA | H | 2 | 4.0 | 10.0 | 10.0 | [657] |
| 1349 | *Colwellia piezophila* | FA | H | 2 | 4.0 | 10.0 | 10.0 | [657] |
| 1350 | *Colwellia piezophila* | FA | H | 2 | 4.0 | 10.0 | 10.0 | [657] |
| 1351 | *Colwellia piezophila* | FA | H | 2 | 4.0 | 4.0 | 10.0 | [657] |
| 1352 | *Thiobacillus sp.* | A | A | 6 | 0.1 | 18.4 | 30.0 | [658] |
| 1353 | *Thiobacillus thioparus* | FA | A | 5 | 10.2 | 30.2 | 35.0 | [658] |
| 1354 | *Clostridium hastiforme* | AN | H | 4 | 4.4 | 15.0 | 15.0 | [659] |
| 1355 | *Pseudomonas sp.* | FA | H | 6 | 0.0 | 10.5 | 24.9 | [660] |
| 1356 | *Psychromonas sp.* | FA | H | 6 | -0.0 | 5.5 | 19.6 | [660] |
| 1357 | *Shewanella sp.* | FA | H | 9 | 0.1 | 15.5 | 25.1 | [660] |
| 1358 | *Herminiimonas sp.* | A | H | 5 | 3.6 | 9.2 | 14.1 | [660] |
| 1359 | *Arcobacter sp.* | A | H | 6 | -0.2 | 10.4 | 25.4 | [660] |
| 1360 | *Listeria monocytogenes* | FA | H | 23 | -2.0 | 34.0 | 42.0 | [661] |
| 1361 | *Bacterial str.* | AN | H | 17 | -1.1 | 18.9 | 34.2 | [662] |
| 1362 | *Psychrobacter muriicola* | A | H | 6 | 4.9 | 17.9 | 36.8 | [663] |
| 1363 | *Clostridium bowmanii* | AN | H | 11 | 8.8 | 11.6 | 18.7 | [664] |
| 1364 | *Clostridium estertheticum* | AN | H | 7 | 3.1 | 6.8 | 11.5 | [664] |
| 1365 | *Clostridium frigoris* | AN | H | 7 | 3.0 | 5.4 | 11.5 | [664] |
| 1366 | *Clostridium lacusfryxellense* | AN | H | 8 | 3.1 | 10.5 | 11.4 | [664] |
| 1367 | *Clostridium psychrophilum* | AN | H | 6 | 2.9 | 4.5 | 10.2 | [664] |
| 1368 | *Salmo gairdneri* | A | H | 12 | 8.1 | 15.5 | 22.3 | [665] |
| 1369 | *Chaetoceros simplex* | A | A | 4 | 13.3 | 25.2 | 25.2 | [666] |
| 1370 | *Coscinodiscus sp.* | A | A | 3 | 9.9 | 16.4 | 16.4 | [666] |
| 1371 | *Cyclotella cryptica* | A | A | 5 | 10.7 | 25.6 | 25.6 | [666] |
| 1372 | *Ditylum brightwellii* | A | A | 5 | 9.3 | 20.7 | 25.5 | [666] |

(Table S2 continued.)

| Code | Strain/species name | Aero. | Troph. | Smp. | $T_{\mathrm{min}}$ | $T_{\mathrm{opt}}$ | $T_{\mathrm{max}}$ | Lit. |
|---|---|---|---|---|---|---|---|---|
| 1373 | *Phaeodactylum tricornutum* | A | A | 6 | 8.6 | 20.8 | 25.6 | [666] |
| 1374 | *Skeletonema costatum* | A | A | 5 | 9.0 | 20.0 | 24.8 | [666] |
| 1375 | *Thalassiosira eccentrica* | A | A | 5 | 9.4 | 24.7 | 24.7 | [666] |
| 1376 | *Thalassiosira weissflogii* | A | A | 5 | 9.7 | 14.2 | 26.0 | [666] |
| 1377 | *Isochrysis galbana* | A | A | 4 | 13.0 | 25.1 | 25.1 | [666] |
| 1378 | *Rhodomonas salina* | A | A | 5 | 9.1 | 16.1 | 25.1 | [666] |
| 1379 | *Vibrio sp.* | FA | H | 4 | 1.0 | 7.0 | 10.0 | [667] |
| 1380 | *Arthrobacter glacialis* | A | H | 7 | 0.3 | 14.6 | 16.3 | [668] |
| 1381 | *Arthrobacter glacialis* | A | H | 9 | 1.7 | 13.4 | 20.4 | [668] |
| 1382 | *Arthrobacter sp.* | A | H | 9 | 0.2 | 28.4 | 34.5 | [668] |
| 1383 | *Arthrobacter sp.* | A | H | 10 | 4.3 | 28.5 | 34.3 | [668] |
| 1384 | *Pseudomonas sp.* | A | H | 9 | 2.4 | 25.5 | 35.4 | [668] |
| 1385 | *Bacillus circulans* | FA | H | 12 | 0.3 | 24.8 | 34.7 | [669] |
| 1386 | *Bacillus coagulans* | FA | H | 9 | 6.1 | 29.8 | 39.6 | [669] |
| 1387 | *Bacillus coagulans* | FA | H | 12 | 0.6 | 30.0 | 34.7 | [669] |
| 1388 | *Bacillus laterosporus* | FA | H | 8 | 6.2 | 36.5 | 40.1 | [669] |
| 1389 | *Thermodesulfobacterium hveragerdense* | AN | A | 4 | 60.0 | 70.0 | 74.2 | [670] |
| 1390 | *Thermodesulfovibrio islandicus* | AN | A | 5 | 50.1 | 65.4 | 70.2 | [670] |
| 1391 | *Desulfovibrio capillatus* | AN | H | 6 | 19.9 | 39.4 | 45.1 | [671] |
| 1392 | *Methanococcoides alaskense* | AN | H | 6 | 15.0 | 25.0 | 28.0 | [672] |
| 1393 | *Methanosarcina baltica* | AN | H | 7 | 10.0 | 20.0 | 25.0 | [672] |
| 1394 | *Clostridium thermoalcaliphilum* | AN | H | 18 | 26.6 | 49.5 | 57.0 | [673] |
| 1395 | *Anaerobaculum mobile* | AN | H | 6 | 35.0 | 54.9 | 65.0 | [674] |
| 1396 | *Clostridium paradoxum* | AN | H | 15 | 35.4 | 55.5 | 62.0 | [675] |
| 1397 | *Clostridium paradoxum* | AN | H | 19 | 30.0 | 55.5 | 62.1 | [675] |

(Table S2 continued.)

| Code | Strain/species name | Aero. | Troph. | Smp. | $T_{\mathrm{min}}$ | $T_{\mathrm{opt}}$ | $T_{\mathrm{max}}$ | Lit. |
|---|---|---|---|---|---|---|---|---|
| 1398 | *Deferribacter thermophilus* | AN | H | 6 | 49.9 | 59.9 | 65.1 | [676] |
| 1399 | *Bacillus sp.* | FA | H | 24 | 1.8 | 25.1 | 31.7 | [677] |
| 1400 | *Gram-negative str.* | A | H | 19 | 3.1 | 27.0 | 31.6 | [677] |
| 1401 | *Thermoanaerobacter brockii* | AN | H | 7 | 45.1 | 55.0 | 74.6 | [678] |
| 1402 | *Dethiosulfovibrio peptidovorans* | AN | H | 7 | 24.8 | 42.1 | 44.9 | [679] |
| 1403 | *Methanocalculus halotolerans* | AN | A | 5 | 28.9 | 36.9 | 44.8 | [680] |
| 1404 | *Haloanaerobium alcaliphilum* | AN | H | 7 | 19.9 | 36.4 | 49.7 | [681] |
| 1405 | *Haloanaerobium lacusroseus* | AN | H | 7 | 25.2 | 40.0 | 45.3 | [682] |
| 1406 | *Thermotoga hypogea* | AN | H | 8 | 56.5 | 69.9 | 89.8 | [683] |
| 1407 | *Methanosarcina siciliae* | AN | H | 5 | 15.0 | 35.1 | 35.1 | [684] |
| 1408 | *Thermanaerovibrio velox* | AN | H | 6 | 45.1 | 59.9 | 70.1 | [685] |
| 1409 | *Thermoanaerobacter siderophilus* | AN | H | 11 | 38.8 | 68.6 | 78.0 | [686] |
| 1410 | *Thermoanaerobacter yonseiensis* | AN | H | 7 | 50.0 | 74.9 | 82.8 | [687] |
| 1411 | *Thermanaeromonas toyohensis* | AN | H | 5 | 55.0 | 70.0 | 72.9 | [688] |
| 1412 | *Methanocalculus taiwanensis* | AN | A | 2 | 31.9 | 37.1 | 37.1 | [689] |
| 1413 | *Methanocalculus taiwanensis* | AN | A | 8 | 24.7 | 37.1 | 41.9 | [689] |
| 1414 | *Brochothrix thermosphacta* | FA | H | 6 | 1.6 | 11.7 | 14.9 | [690] |
| 1415 | *Chlorella vulgaris* | A | A | 7 | 10.0 | 30.1 | 40.1 | [691] |
| 1416 | *Nautilia abyssi* | AN | A | 7 | 33.0 | 60.0 | 64.9 | [692] |
| 1417 | *Pseudomonas fluorescens* | A | H | 11 | 0.0 | 29.8 | 31.7 | [693] |
| 1418 | *Aeromonas hydrophila* | FA | H | 9 | 0.5 | 18.4 | 44.5 | [694] |
| 1419 | *Aeromonas hydrophila* | FA | H | 9 | 15.1 | 35.2 | 56.8 | [694] |
| 1420 | *Aeromonas hydrophila* | FA | H | 8 | 1.9 | 19.8 | 39.4 | [694] |
| 1421 | *Aeromonas salmonicida* | FA | H | 6 | 13.3 | 28.2 | 38.5 | [694] |
| 1422 | *Aeromonas shigelloides* | FA | H | 10 | 9.2 | 33.6 | 55.9 | [694] |

(Table S2 continued.)

| Code | Strain/species name | Aero. | Troph. | Smp. | $T_{\min}$ | $T_{\text{opt}}$ | $T_{\max}$ | Lit. |
|---|---|---|---|---|---|---|---|---|
| 1423 | *Aeromonas shigelloides* | FA | H | 10 | -0.0 | 33.0 | 44.4 | [694] |
| 1424 | *Alicyclobacillus sp.* | A | M | 14 | 39.0 | 61.9 | 66.9 | [695] |
| 1425 | *Desulfomicrobium thermophilum* | AN | H | 4 | 37.0 | 55.2 | 55.2 | [696] |
| 1426 | *Bacillus sp.* | A | H | 7 | 45.1 | 65.0 | 73.0 | [697] |
| 1427 | *Sulfobacillus sibiricus* | FA | M | 6 | 30.1 | 55.0 | 60.1 | [698] |
| 1428 | *Protogonyaulax tamarensis* | A | A | 3 | 8.0 | 16.0 | 16.0 | [699] |
| 1429 | *Cyanidium caldarium* | A | A | 9 | 24.0 | 45.0 | 51.0 | [700] |
| 1430 | *Cyanidium caldarium* | A | A | 5 | 33.0 | 45.0 | 45.0 | [700] |
| 1431 | *Vibrio sp.* | FA | H | 3 | 0.0 | 15.0 | 15.0 | [701] |
| 1432 | *Vibrio sp.* | FA | H | 3 | 0.0 | 15.0 | 15.0 | [701] |
| 1433 | *Vibrio sp.* | FA | H | 3 | 0.0 | 15.0 | 15.0 | [701] |
| 1434 | *Vibrio sp.* | FA | H | 4 | 0.0 | 15.0 | 23.0 | [701] |
| 1435 | *Vibrio sp.* | FA | H | 3 | 0.0 | 15.0 | 15.0 | [701] |
| 1436 | *Vibrio sp.* | FA | H | 4 | 0.0 | 15.0 | 23.0 | [701] |
| 1437 | *Vibrio sp.* | FA | H | 4 | 0.0 | 15.0 | 23.0 | [701] |
| 1438 | *Vibrio sp.* | FA | H | 3 | 0.0 | 15.0 | 15.0 | [701] |
| 1439 | *Vibrio sp.* | FA | H | 4 | 0.0 | 15.0 | 23.0 | [701] |
| 1440 | *Chlamydomonas alpina* | A | A | 8 | -1.0 | 12.5 | 18.0 | [702] |
| 1441 | *Chlamydomonas globosa* | A | A | 7 | 5.0 | 18.0 | 20.0 | [702] |
| 1442 | *Chlamydomonas intermedia* | A | A | 8 | -1.0 | 18.0 | 18.0 | [702] |
| 1443 | *Chlamydomonas subcaudata* | A | A | 8 | -1.0 | 12.5 | 18.0 | [702] |
| 1444 | *Stichococcus sp.* | A | A | 6 | 4.0 | 7.5 | 30.0 | [703] |
| 1445 | *Navicula sp.* | A | A | 6 | 4.0 | 4.0 | 30.0 | [703] |
| 1446 | *Chlorella sp.* | A | A | 6 | 4.0 | 20.0 | 30.0 | [703] |
| 1447 | *Chlorella sp.* | A | A | 6 | 4.0 | 20.0 | 30.0 | [703] |

(Table S2 continued.)

| Code | Strain/species name | Aero. | Troph. | Smp. | $T_{\mathrm{min}}$ | $T_{\mathrm{opt}}$ | $T_{\mathrm{max}}$ | Lit. |
|---|---|---|---|---|---|---|---|---|
| 1448 | *Klebsormidium sp.* | A | A | 6 | 4.0 | 14.0 | 30.0 | [703] |
| 1449 | *Chlamydomonas sp.* | A | A | 6 | 4.0 | 9.0 | 30.0 | [703] |
| 1450 | *Chlamydomonas sp.* | A | A | 5 | 4.1 | 18.2 | 32.1 | [704] |
| 1451 | *Chlamydomonas sp.* | A | A | 6 | 13.2 | 28.2 | 38.0 | [704] |
| 1452 | *Chlorella sp.* | A | A | 5 | 3.9 | 17.9 | 31.8 | [704] |
| 1453 | *Chlorella sp.* | A | A | 6 | 13.0 | 33.1 | 38.1 | [704] |
| 1454 | *Amphiprora sp.* | A | A | 6 | 12.6 | 27.8 | 37.9 | [704] |
| 1455 | *Navicula sp.* | A | A | 5 | 3.8 | 10.8 | 31.8 | [704] |
| 1456 | *Hantzschia amphyoxis* | A | A | 6 | 6.0 | 18.0 | 25.0 | [705] |
| 1457 | *Pseudomonas fluorescens* | A | H | 6 | 4.0 | 25.0 | 32.0 | [706] |
| 1458 | *Pseudomonas fluorescens* | A | H | 6 | 4.0 | 32.0 | 32.0 | [706] |
| 1459 | *Pseudomonas fluorescens* | A | H | 6 | 4.0 | 25.0 | 32.0 | [706] |
| 1460 | *Pseudomonas fluorescens* | A | H | 6 | 4.0 | 25.0 | 32.0 | [706] |
| 1461 | *Pseudomonas fluorescens* | A | H | 6 | 4.0 | 25.0 | 32.0 | [706] |
| 1462 | *Pseudomonas fluorescens* | A | H | 6 | 4.0 | 25.0 | 32.0 | [706] |
| 1463 | *Clostridium thermohydrosulfuricum* | AN | H | 17 | 47.9 | 68.0 | 75.7 | [707] |
| 1464 | *Synura sphagnicola* | A | A | 7 | 5.0 | 15.0 | 20.0 | [708] |
| 1465 | *Synura sphagnicola* | A | A | 5 | 5.0 | 10.0 | 15.0 | [708] |
| 1466 | *Synura sphagnicola* | A | A | 6 | 5.0 | 10.0 | 17.5 | [708] |
| 1467 | *Synura sphagnicola* | A | A | 6 | 5.0 | 10.0 | 17.5 | [708] |
| 1468 | *Synura sphagnicola* | A | A | 7 | 5.0 | 12.5 | 20.0 | [708] |
| 1469 | *Synura sphagnicola* | A | A | 7 | 5.0 | 15.0 | 20.0 | [708] |
| 1470 | *Synura sphagnicola* | A | A | 7 | 5.0 | 12.5 | 20.0 | [708] |
| 1471 | *Synura sphagnicola* | A | A | 7 | 5.0 | 15.0 | 20.0 | [708] |
| 1472 | *Escherichia coli* | FA | H | 5 | 10.0 | 30.0 | 30.0 | [709] |

(Table S2 continued.)

| Code | Strain/species name | Aero. | Troph. | Smp. | $T_{\mathrm{min}}$ | $T_{\mathrm{opt}}$ | $T_{\mathrm{max}}$ | Lit. |
|---|---|---|---|---|---|---|---|---|
| 1473 | *Pseudomonas aeruginosa* | A | H | 5 | 10.0 | 30.0 | 30.0 | [709] |
| 1474 | *Pseudomonas sp.* | A | H | 6 | 5.0 | 30.0 | 30.0 | [709] |
| 1475 | *Pseudomonas sp.* | A | H | 6 | 5.0 | 30.0 | 30.0 | [709] |
| 1476 | *Pseudomonas sp.* | A | H | 6 | 5.0 | 30.0 | 30.0 | [709] |
| 1477 | *Pseudomonas sp.* | A | H | 6 | 5.0 | 30.0 | 30.0 | [709] |
| 1478 | *Pseudomonas sp.* | A | H | 6 | 5.0 | 30.0 | 30.0 | [709] |
| 1479 | *Pseudomonas sp.* | A | H | 7 | 5.0 | 30.0 | 37.0 | [709] |
| 1480 | *Coliform sp.* | A | H | 7 | 5.0 | 37.0 | 37.0 | [709] |
| 1481 | *Coliform sp.* | A | H | 6 | 10.0 | 37.0 | 37.0 | [709] |
| 1482 | *Coliform sp.* | A | H | 5 | 10.0 | 30.0 | 30.0 | [709] |
| 1483 | *Coliform sp.* | A | H | 6 | 5.0 | 30.0 | 30.0 | [709] |
| 1484 | *Coliform sp.* | A | H | 6 | 10.0 | 37.0 | 37.0 | [709] |
| 1485 | *Psychrophilic coliform* | A | H | 5 | 10.0 | 30.0 | 30.0 | [709] |
| 1486 | *Psychrophilic coliform* | A | H | 1 | 5.0 | 5.0 | 5.0 | [709] |
| 1487 | *Synechocystis sp.* | A | A | 8 | 28.0 | 40.0 | 44.0 | [710] |
| 1488 | *Synechocystis sp.* | A | A | 7 | 30.0 | 36.0 | 44.0 | [710] |
| 1489 | *Synechocystis sp.* | A | A | 10 | 32.9 | 42.0 | 44.0 | [710] |
| 1490 | *Synechocystis sp.* | A | A | 14 | 33.0 | 42.5 | 44.5 | [710] |
| 1491 | *Synechocystis sp.* | A | A | 12 | 33.0 | 40.5 | 43.5 | [710] |
| 1492 | *Synechocystis sp.* | A | A | 14 | 33.0 | 42.4 | 45.0 | [710] |
| 1493 | *Synechocystis sp.* | A | A | 15 | 32.9 | 37.0 | 45.0 | [710] |
| 1494 | *Synechocystis sp.* | A | A | 13 | 33.0 | 40.5 | 43.5 | [710] |
| 1495 | *Synechocystis sp.* | A | A | 6 | 28.0 | 36.0 | 40.0 | [710] |
| 1496 | *Daphnia parvula* | A | H | 3 | 9.9 | 25.0 | 25.0 | [711] |
| 1497 | *Daphnia parvula* | A | H | 3 | 10.0 | 25.0 | 25.0 | [711] |

(Table S2 continued.)

| Code | Strain/species name | Aero. | Troph. | Smp. | $T_{min}$ | $T_{opt}$ | $T_{max}$ | Lit. |
|---|---|---|---|---|---|---|---|---|
| 1498 | *Daphnia parvula* | A | H | 3 | 10.0 | 25.1 | 25.1 | [711] |
| 1499 | *Daphnia parvula* | A | H | 3 | 8.0 | 25.0 | 25.0 | [711] |
| 1500 | *Daphnia parvula* | A | H | 3 | 7.9 | 25.0 | 25.0 | [711] |
| 1501 | *Nitzschia seriata* | A | A | 9 | -1.6 | 10.0 | 12.0 | [712] |
| 1502 | *Chaetoceros deflandrei* | A | A | 6 | 2.0 | 7.0 | 15.0 | [713] |
| 1503 | *Chaetoceros deflandrei* | A | A | 6 | 2.0 | 5.1 | 15.0 | [713] |
| 1504 | *Chaetoceros deflandrei* | A | A | 6 | 2.1 | 7.0 | 14.9 | [713] |
| 1505 | *Chaetoceros deflandrei* | A | A | 5 | 2.0 | 15.0 | 15.0 | [713] |
| 1506 | *Corethron criophilum* | A | A | 4 | 0.1 | 4.0 | 6.0 | [713] |
| 1507 | *Corethron criophilum* | A | A | 4 | 0.1 | 4.0 | 6.0 | [713] |
| 1508 | *Corethron criophilum* | A | A | 3 | 0.2 | 4.0 | 4.0 | [713] |
| 1509 | *Corethron criophilum* | A | A | 4 | 0.1 | 4.0 | 6.0 | [713] |
| 1510 | *Nitzchia kerguelensis* | A | A | 4 | 0.1 | 4.1 | 7.0 | [713] |
| 1511 | *Nitzchia kerguelensis* | A | A | 4 | 0.1 | 4.0 | 7.0 | [713] |
| 1512 | *Nitzchia kerguelensis* | A | A | 4 | 0.1 | 4.0 | 7.0 | [713] |
| 1513 | *Nitzchia kerguelensis* | A | A | 4 | 0.1 | 4.1 | 7.0 | [713] |
| 1514 | *Nitzschia cylindrus* | A | A | 5 | 0.1 | 2.5 | 4.5 | [713] |
| 1515 | *Nitzschia cylindrus* | A | A | 5 | 0.0 | 2.5 | 4.5 | [713] |
| 1516 | *Nitzschia cylindrus* | A | A | 5 | 0.1 | 2.5 | 4.5 | [713] |
| 1517 | *Nitzschia cylindrus* | A | A | 5 | 0.0 | 2.5 | 4.5 | [713] |
| 1518 | *Nitzschia turgiduloides* | A | A | 4 | 1.0 | 3.1 | 8.0 | [713] |
| 1519 | *Nitzschia turgiduloides* | A | A | 5 | 1.0 | 3.0 | 8.0 | [713] |
| 1520 | *Nitzschia turgiduloides* | A | A | 5 | 1.1 | 5.0 | 8.0 | [713] |
| 1521 | *Nitzschia turgiduloides* | A | A | 5 | 1.0 | 3.1 | 8.1 | [713] |
| 1522 | *Stellarima microtrias* | A | A | 3 | 1.0 | 4.0 | 7.0 | [713] |

(Table S2 continued.)

| Code | Strain/species name | Aero. | Troph. | Smp. | $T_{min}$ | $T_{opt}$ | $T_{max}$ | Lit. |
|---|---|---|---|---|---|---|---|---|
| 1523 | *Stellarima microtrias* | A | A | 3 | 1.0 | 4.0 | 7.0 | [713] |
| 1524 | *Stellarima microtrias* | A | A | 3 | 1.0 | 4.0 | 7.0 | [713] |
| 1525 | *Stellarima microtrias* | A | A | 3 | 1.0 | 4.0 | 7.0 | [713] |
| 1526 | *Synedra sp.* | A | A | 3 | 1.0 | 5.0 | 7.1 | [713] |
| 1527 | *Synedra sp.* | A | A | 3 | 1.1 | 5.0 | 7.0 | [713] |
| 1528 | *Synedra sp.* | A | A | 3 | 1.0 | 5.0 | 7.0 | [713] |
| 1529 | *Synedra sp.* | A | A | 3 | 1.0 | 5.0 | 7.1 | [713] |
| 1530 | *Chaetoceros sp.* | A | A | 2 | 0.0 | 10.0 | 10.0 | [714] |
| 1531 | *Chaetoceros sp.* | A | A | 2 | 0.0 | 10.0 | 10.0 | [714] |
| 1532 | *Navicula sp.* | A | A | 1 | 10.0 | 10.0 | 10.0 | [714] |
| 1533 | *Nitzschia sp.* | A | A | 2 | 0.0 | 10.0 | 10.0 | [714] |
| 1534 | *Kosmotoga arenicorallina* | A | H | 4 | 50.1 | 60.1 | 65.1 | [715] |
| 1535 | *Thermomicrobium fosteri* | A | H | 21 | 50.0 | 60.0 | 65.1 | [716] |
| 1536 | *Desulfurobacterium thermolithotrophum* | AN | A | 9 | 37.1 | 69.5 | 74.7 | [717] |
| 1537 | *Moorella gycerini* | AN | H | 11 | 43.0 | 58.0 | 65.0 | [718] |
| 1538 | *Methanogenium frittonii* | AN | A | 14 | 32.3 | 57.4 | 61.8 | [719] |
| 1539 | *Methanogenium cariaci* | AN | A | 4 | 19.9 | 37.4 | 37.4 | [720] |
| 1540 | *Methanogenium marisnigri* | AN | A | 5 | 24.8 | 37.5 | 45.0 | [720] |
| 1541 | *Methanogenium tatii* | AN | A | 5 | 24.8 | 39.9 | 44.9 | [720] |
| 1542 | *Sporomusa acidovorans* | AN | H | 5 | 24.9 | 34.4 | 38.8 | [721] |
| 1543 | *Chlamydomonas raudensis* | A | A | 7 | 6.2 | 12.4 | 27.0 | [722] |
| 1544 | *Chlamydomonas raudensis* | A | A | 7 | 6.1 | 12.2 | 27.1 | [722] |
| 1545 | *Chlamydomonas raudensis* | A | A | 8 | 6.1 | 20.3 | 30.1 | [722] |
| 1546 | *Mobilitalea sibirica* | AN | H | 1 | 37.0 | 37.0 | 37.0 | [723] |
| 1547 | *Chlamydomonas raudensis* | A | A | 2 | 8.0 | 15.0 | 15.0 | [724] |

(Table S2 continued.)

| Code | Strain/species name | Aero. | Troph. | Smp. | $T_{min}$ | $T_{opt}$ | $T_{max}$ | Lit. |
|---|---|---|---|---|---|---|---|---|
| 1548 | *Chlamydomonas raudensis* | A | A | 2 | 8.0 | 8.0 | 15.0 | [724] |
| 1549 | *Chlamydomonas raudensis* | A | A | 1 | 8.0 | 8.0 | 8.0 | [724] |
| 1550 | *Chlamydomonas raudensis* | A | A | 2 | 8.0 | 15.0 | 15.0 | [724] |
| 1551 | *Chlamydomonas raudensis* | A | A | 2 | 8.0 | 8.0 | 15.0 | [724] |
| 1552 | *Methanogenium thermophilicum* | AN | H | 7 | 36.9 | 57.7 | 64.1 | [725] |
| 1553 | *Archaeoglobus veneficus* | AN | A | 5 | 64.9 | 79.9 | 85.0 | [726] |
| 1554 | *Galenea microaerophila* | microA | M | 7 | 20.0 | 35.0 | 50.0 | [727] |
| 1555 | *Thermonema lapsum* | A | H | 7 | 35.0 | 60.0 | 65.0 | [728] |
| 1556 | *Thermonema rossianum* | A | H | 7 | 35.0 | 59.9 | 65.1 | [728] |
| 1557 | *Thermonema rossianum* | A | H | 7 | 35.0 | 60.0 | 65.0 | [728] |
| 1558 | *Methanolobus siciliae* | AN | A | 4 | 31.1 | 40.2 | 42.2 | [729] |
| 1559 | *Methanolobus siciliae* | AN | A | 5 | 19.9 | 40.1 | 40.1 | [729] |
| 1560 | *Anaerobranca horikoshii* | AN | H | 19 | 34.0 | 56.3 | 65.4 | [730] |
| 1561 | *Rubrobacter radiotolerans* | A | H | 5 | 37.0 | 49.9 | 59.9 | [731] |
| 1562 | *Rubrobacter xylanophilus* | A | H | 9 | 30.1 | 59.8 | 69.8 | [731] |
| 1563 | *Sphaerochaeta multiformis* | AN | H | 1 | 9.0 | 9.0 | 9.0 | [732] |
| 1564 | *Caloramator indicus* | AN | H | 5 | 55.3 | 65.3 | 75.2 | [733] |
| 1565 | *Thermobrachium celere* | AN | H | 17 | 50.0 | 66.8 | 71.8 | [734] |
| 1566 | *Thermosyntropha lipolytica* | AN | H | 5 | 52.1 | 67.3 | 70.2 | [735] |
| 1567 | *Thermosyntropha lipolytica* | AN | H | 6 | 52.1 | 67.7 | 73.1 | [735] |
| 1568 | *Thermosyntropha lipolytica* | AN | H | 5 | 52.1 | 67.6 | 70.2 | [735] |
| 1569 | *Hydrogenobacter hydrogenophilus* | microA | A | 8 | 45.1 | 64.9 | 77.6 | [736] |
| 1570 | *Methanopyrus sp.* | AN | A | 5 | 84.7 | 99.8 | 109.8 | [737] |
| 1571 | *Thermoplasma acidophila* | A | H | 6 | 45.3 | 59.2 | 62.3 | [738] |
| 1572 | *Thermoplasma acidophila* | A | H | 6 | 45.3 | 59.2 | 62.2 | [738] |

(Table S2 continued.)

| Code | Strain/species name | Aero. | Troph. | Smp. | $T_{\mathrm{min}}$ | $T_{\mathrm{opt}}$ | $T_{\mathrm{max}}$ | Lit. |
|---|---|---|---|---|---|---|---|---|
| 1573 | *Thermothrix thiopara* | FA | M | 5 | 59.1 | 72.5 | 72.5 | [739] |
| 1574 | *Methanococcus thermolithotrophic* | AN | A | 10 | 30.3 | 64.9 | 70.1 | [740] |
| 1575 | *Methanococcus voltae* | AN | A | 5 | 25.3 | 40.2 | 43.1 | [740] |
| 1576 | *Desulfurolobus ambivalens* | FA | A | 4 | 70.2 | 79.4 | 87.7 | [741] |
| 1577 | *Thermococcus stetteri* | AN | H | 6 | 60.1 | 74.8 | 84.6 | [742] |
| 1578 | *Methanobacterium defluvii* | AN | A | 7 | 36.2 | 60.8 | 69.0 | [743] |
| 1579 | *Methanobacterium thermoflexum* | AN | A | 6 | 36.5 | 54.6 | 69.5 | [743] |
| 1580 | *Methanococcus igneus* | AN | A | 10 | 40.7 | 88.2 | 90.1 | [744] |
| 1581 | *Archaeoglobus profundus* | AN | M | 6 | 75.4 | 81.9 | 90.6 | [745] |
| 1582 | *Thermococcus profundus* | AN | H | 5 | 50.1 | 80.3 | 90.2 | [746] |
| 1583 | *Gelria glutamica* | AN | H | 1 | 52.5 | 52.5 | 52.5 | [747] |
| 1584 | *Clostridium thiosulfatireducens* | AN | H | 4 | 20.1 | 37.0 | 40.0 | [748] |
| 1585 | *Thermoanaerobium brockii* | AN | H | 8 | 35.0 | 70.4 | 84.5 | [749] |
| 1586 | *Desulfotomaculum geothermicum* | AN | A | 6 | 44.7 | 53.9 | 55.2 | [750] |
| 1587 | *Desulfurobacterium crinifex* | AN | A | 5 | 50.0 | 60.2 | 70.0 | [751] |
| 1588 | *Methanomethylovorans uponensis* | AN | H | 1 | 37.0 | 37.0 | 37.0 | [752] |
| 1589 | *Coprothermobacter platensis* | AN | H | 1 | 55.0 | 55.0 | 55.0 | [753] |
| 1590 | *Clostridium thermopapyrolyticum* | AN | H | 1 | 59.0 | 59.0 | 59.0 | [754] |
| 1591 | *Nautilia lithotrophica* | AN | A | 1 | 53.0 | 53.0 | 53.0 | [755] |
| 1592 | *Fusibacter paucivorans* | AN | H | 4 | 19.0 | 37.0 | 37.0 | [756] |
| 1593 | *Thermus thermophilus* | A | H | 1 | 75.0 | 75.0 | 75.0 | [757] |
| 1594 | *Blastocatella fastidiosa* | A | H | 1 | 32.5 | 32.5 | 32.5 | [758] |
| 1595 | *Bryocella elongata* | A | H | 1 | 22.0 | 22.0 | 22.0 | [759] |
| 1596 | *Alkalibacterium indicireducens* | FA | H | 1 | 25.0 | 25.0 | 25.0 | [760] |
| 1597 | *Marinilactibacillus psychrotolerans* | AN | H | 4 | 25.0 | 37.0 | 42.5 | [761] |

(Table S2 continued.)

| Code | Strain/species name | Aero. | Troph. | Smp. | $T_{min}$ | $T_{opt}$ | $T_{max}$ | Lit. |
|---|---|---|---|---|---|---|---|---|
| 1598 | *Caldisphaera lagunensis* | AN | H | 1 | 75.0 | 75.0 | 75.0 | [762] |
| 1599 | *Alkalibacterium iburiense* | FA | H | 6 | 15.0 | 30.0 | 40.0 | [763] |
| 1600 | *Alkalibacterium iburiense* | FA | H | 6 | 15.0 | 30.0 | 40.0 | [763] |
| 1601 | *Alkalibacterium iburiense* | FA | H | 6 | 15.0 | 37.0 | 40.0 | [763] |
| 1602 | *Shewanella profunda* | AN | H | 1 | 30.0 | 30.0 | 30.0 | [764] |
| 1603 | *Alkalibacterium psychrotolerans* | AN | H | 7 | 10.0 | 34.0 | 40.0 | [765] |
| 1604 | *Desulfovibrio piezophilus* | AN | H | 1 | 30.0 | 30.0 | 30.0 | [766] |
| 1605 | *Acidiplasma aeolicum* | FA | H | 1 | 43.5 | 43.5 | 43.5 | [767] |
| 1606 | *Paludibaculum fermentans* | FA | H | 1 | 24.0 | 24.0 | 24.0 | [768] |
| 1607 | *Halobacterium noricense* | FA | H | 1 | 37.0 | 37.0 | 37.0 | [769] |
| 1608 | *Alkalibacterium olivoapovliticus* | AN | H | 1 | 29.0 | 29.0 | 29.0 | [770] |
| 1609 | *Halobacillus litoralis* | A | H | 1 | 35.0 | 35.0 | 35.0 | [771] |
| 1610 | *Halobacillus trueperi* | A | H | 1 | 35.0 | 35.0 | 35.0 | [771] |
| 1611 | *Terriglobus albidus* | A | H | 1 | 33.0 | 33.0 | 33.0 | [772] |
| 1612 | *Marinilactibacillus piezotolerans* | FA | H | 1 | 38.5 | 38.5 | 38.5 | [773] |
| 1613 | *Halobacillus mangrovi* | A | H | 1 | 34.0 | 34.0 | 34.0 | [774] |
| 1614 | *Flavobacterium antarcticum* | A | H | 8 | 5.0 | 21.9 | 24.1 | [775] |
| 1615 | *Amphibacillus cookii* | FA | H | 1 | 37.0 | 37.0 | 37.0 | [776] |
| 1616 | *Desulfovibrio dechloracetivorans* | AN | H | 2 | 25.0 | 30.0 | 30.0 | [777] |
| 1617 | *Desulfomicrobium orale* | AN | H | 1 | 37.0 | 37.0 | 37.0 | [778] |
| 1618 | *Desulfovibrio sp.* | AN | H | 1 | 37.0 | 37.0 | 37.0 | [778] |
| 1619 | *Desulfobacter curvatus* | AN | H | 1 | 30.0 | 30.0 | 30.0 | [779] |
| 1620 | *Desulfobacter hydrogenophilus* | AN | H | 1 | 30.5 | 30.5 | 30.5 | [779] |
| 1621 | *Desulfobacter latus* | AN | H | 1 | 30.5 | 30.5 | 30.5 | [779] |
| 1622 | *Halomonas sinaiensis* | FA | H | 1 | 35.0 | 35.0 | 35.0 | [780] |

(Table S2 continued.)

| Code | Strain/species name | Aero. | Troph. | Smp. | $T_{\mathrm{min}}$ | $T_{\mathrm{opt}}$ | $T_{\mathrm{max}}$ | Lit. |
|---|---|---|---|---|---|---|---|---|
| 1623 | *Desulfovibrio inopinatus* | AN | H | 1 | 30.0 | 30.0 | 30.0 | [781] |
| 1624 | *Anaerobacillus alkalilacustre* | AN | H | 1 | 32.5 | 32.5 | 32.5 | [782] |
| 1625 | *Desulfurella karnchatkensis* | AN | H | 1 | 54.0 | 54.0 | 54.0 | [783] |
| 1626 | *Desulfurella propionica* | AN | H | 1 | 55.0 | 55.0 | 55.0 | [783] |
| 1627 | *Desulfovibrio intestinalis* | AN | H | 1 | 37.0 | 37.0 | 37.0 | [784] |

# References


1. Eagon RG. *Pseudomonas natriegens*, a marine bacterium with a generation time of less than 10 minutes. J Bacteriol. 1962;83(4):736–737.

2. Karnauchow TM, Koval SF, Jarrell KF. Isolation and characterization of three thermophilic anaerobes from a St. Lucia hot spring. Syst Appl Microbiol. 1992;15(2):296–310.

3. Xiao Z, Wang X, Huang Y, Huo F, Zhu X, Xi L, et al. Thermophilic fermentation of acetoin and 2, 3-butanediol by a novel *Geobacillus* strain. Biotechnol Biofuels. 2012;5(1):88.

4. Rivard CJ, Smith PH. Isolation and characterization of a thermophilic marine methanogenic bacterium, *Methanogenium thermophilicum* sp. nov. Int J Syst Bacteriol. 1982;32(4):430–436.

5. McLachlan JL, Curtis JM, Boutilier K, Keusgen M, Seguel MR. *Tetreutreptia pomquetensis* (Euglenophyta), a psychrophilic species: growth and fatty acid composition. J Phycol. 1999;35(2):280–286.

6. Barber MA. The rate of multiplication of *Bacillus coli* at different temperatures. J Infect Dis. 1908 October;5(4):379–400. Syn Escherichia coli.

7. Miroshnichenko ML, Kublanov IV, Kostrikina NA, Tourova TP, Kolganova TV, Birkeland NK, et al. *Caldicellulosiruptor kronotskyensis* sp. nov. and *Caldicellulosiruptor hydrothermalis* sp. nov., two extremely thermophilic, cellulolytic, anaerobic bacteria from Kamchatka thermal springs. Int J Syst Evol Microbiol. 2008;58(6):1492–1496.

8. Bredholt S, Sonne-Hansen J, Nielsen P, Mathrani IM, Ahring BK. *Caldicellulosiruptor kristjanssonii* sp. nov., a cellulolytic, extremely thermophilic, anaerobic bacterium. Int J Syst Bacteriol. 1999;49(3):991–996.

9. Nielsen P, Mathrani IM, Ahring BK. *Thermoanaerobium acetigenum* spec. nov., a new anaerobic, extremely thermophilic, xylanolytic non-spore-forming bacterium isolated from an Icelandic hot spring. Arch Microbiol. 1993;159(5):460–464.

10. Yang SJ, Kataeva I, Wiegel J, Yin Y, Dam P, Xu Y, et al. Classification of '*Anaerocellum thermophilum*' strain DSM 6725 as *Caldicellulosiruptor bescii* sp. nov. Int J Syst Evol Microbiol. 2010;60(9):2011–2015.

11. Voordeckers JW, Starovoytov V, Vetriani C. *Caminibacter mediatlanticus* sp. nov., a thermophilic, chemolithoautotrophic, nitrate-ammonifying bacterium isolated from a deep-sea hydrothermal vent on the Mid-Atlantic Ridge. Int J Syst Evol Microbiol. 2005;55(2):773–779.

12. Miroshnichenko ML, l'haridon S, Schumann P, Spring S, Bonch-Osmolovskaya EA, Jeanthon C, et al. *Caminibacter profundus* sp. nov., a novel thermophile of *Nautiliales* ord. nov. within the class '*Epsilonproteobacteria*', isolated from a deep-sea hydrothermal vent. Int J Syst Evol Microbiol. 2004;54(1):41–45.

13. Tada K, Pithakpol S, Montani S. Seasonal variation in the abundance of *Noctiluca scintillans* in the Seto Inland Sea, Japan. Plankton Biol Ecol. 2004;51(1):7–14.

14. Robertson WJ, Kinnunen PHM, Plumb JJ, Franzmann PD, Puhakka JA, Gibson JAE, et al. Moderately thermophilic iron oxidising bacteria isolated from a pyritic coal deposit showing spontaneous combustion. Miner Eng. 2002;15(11):815–822.

15. Losey NA, Stevenson BS, Busse HJ, Damsté JSS, Rijpstra WIC, Rudd S, et al. *Thermoanaerobaculum aquaticum* gen. nov., sp. nov., the first cultivated member of *Acidobacteria* subdivision 23, isolated from a hot spring. Int J Syst Evol Microbiol. 2013;63(Pt 11):4149–4157.

16. Rivkina EM, Friedmann EI, McKay CP, Gilichinsky DA. Metabolic activity of permafrost bacteria below the freezing point. Appl Environ Microbiol. 2000;66(8):3230–3233.

17. Feng Y, Cheng L, Zhang X, Li X, Deng Y, Zhang H. *Thermococcoides shengliensis* gen. nov., sp. nov., a new member of the order Thermotogales isolated from oil-production fluid. Int J Syst Evol Microbiol. 2010;60(4):932–937.

18. Reysenbach AL, Liu Y, Lindgren AR, Wagner ID, Sislak CD, Mets A, et al. *Mesoaciditoga lauensis* gen. nov., sp. nov., a moderately thermoacidophilic member of the order Thermotogales from a deep-sea hydrothermal vent. Int J Syst Evol Microbiol. 2013;63(Pt 12):4724–4729.

19. Wiegel J, Kuk SU, Kohring GW. *Clostridium thermobutyricum* sp. nov., a moderate thermophile isolated from a cellulolytic culture, that produces butyrate as the major product. Int J Syst Bacteriol. 1989;39(2):199–204.

20. Canganella F, Kuk SU, Morgan H, Wiegel J. *Clostridium thermobutyricum*: growth studies and stimulation of butyrate formation by acetate supplementation. Microbiol Res. 2002;157(2):149–156.

21. Kushmarol A, Rosenberg E, Fine M, Haim YB, Loyal Y. Effect of temperature on bleaching of the coral *Oculina patagonica* by *Vibrio* AK-1. Mar Ecol Prog Ser. 1998;171:131–137.

22. Utkin I, Woese C, Wiegel J. Isolation and characterization of *Desulfitobacterium dehalogenans* gen. nov., sp. nov., an anaerobic bacterium

which reductively dechlorinates chlorophenolic compounds. Int J Syst Bacteriol. 1994;44(4):612–619.

23. Freier D, Mothershed CP, Wiegel J. Characterization of *Clostridium thermocellum* JW20. Appl Environ Microbiol. 1988;54(1):204–211.

24. Ricci C. Comparison of five strains of a parthenogenetic species, *Macrotrachela quadricornifera* (Rotifera, Bdelloidea). Hydrobiologia. 1991;211(2):147–155.

25. Adamberg K, Kask S, Laht TM, Paalme T. The effect of temperature and pH on the growth of lactic acid bacteria: a pH-auxostat study. Int J Food Microbiol. 2003;85(1):171–183.

26. Juneja VK, Huang L, Thippareddi HH. Predictive model for growth of *Clostridium perfringens* in cooked cured pork. Int J Food Microbiol. 2006;110(1):85–92.

27. Bottrell HH. Generation time, length of life, instar duration and frequency of moulting, and their relationship to temperature in eight species of Cladocera from the River Thames, Reading. Oecologia. 1975;19(2):129–140.

28. Juneja VK, Marks H, Huang L, Thippareddi H. Predictive model for growth of *Clostridium perfringens* during cooling of cooked uncured meat and poultry. Food Microbiol. 2011;28(4):791–795.

29. Pfennig N, Biebl H. *Desulfuromonas acetoxidans* gen. nov. and sp. nov., a new anaerobic, sulfur-reducing, acetate-oxidizing bacterium. Arch Microbiol. 1976;110(1):3–12.

30. Brock TD, Brock KM, Belly RT, Weiss RL. *Sulfolobus*: a new genus of sulfur-oxidizing bacteria living at low pH and high temperature. Arch Mikrobiol. 1972;84(1):54–68.

31. Lauerer G, Kristjansson JK, Langworthy TA, König H, Stetter KO. *Methanothermus sociabilis* sp. nov., a Second Species within the *Methanothermaceae* Growing at $97^o$ C. Syst Appl Microbiol. 1986;8(1):100–105.

32. Beeder J, Nilsen RK, Rosnes JT, Torsvik T, Lien T. *Archaeoglobus fulgidus* isolated from hot North Sea oil field waters. Appl Environ Microbiol. 1994;60(4):1227–1231.

33. Anderson DH, Benke AC. Growth and reproduction of the cladoceran *Ceriodaphnia dubia* from a forested floodplain swamp. Limnol Oceanogr. 1994;39(7):1517–1527.

34. Cheng Q, Lawrence G, Reed C, Stowers A, Ranford-Cartwright L, Creasey A, et al. Measurement of *Plasmodium falciparum* growth rates *in vivo*: a test of malaria vaccines. Am J Trop Med Hyg. 1997;57(4):495–500.

35. Willardsen RR, Busta FF, Allen CE. growth of *Clostridium-perfringens* in 3 different beef media and fluid thioglycollate medium at static and constantly rising temperatures. J Food Prot. 1979;42(2):144–148.

36. Hethener P, Brauman A, Garcia JL. *Clostridium termitidis* sp. nov., a cellulolytic bacterium from the gut of the wood-feeding termite, *Nasutitermes lujae*. Syst Appl Microbiol. 1992;15(1):52–58.

37. Doerfert SN, Reichlen M, Iyer P, Wang M, Ferry JG. *Methanolobus zinderi* sp. nov., a methylotrophic methanogen isolated from a deep subsurface coal seam. Int J Syst Evol Microbiol. 2009;59(5):1064–1069.

38. Caron DA, Goldman JC, Dennett MR. Effect of temperature on growth, respiration, and nutrient regeneration by an omnivorous microflagellate. Appl Environ Microbiol. 1986;52(6):1340–1347.

39. Sherr BF, Sherr EB, Berman T. Grazing, growth, and ammonium excretion rates of a heterotrophic microflagellate fed with four species of bacteria. Appl Environ Microbiol. 1983;45(4):1196–1201.

40. Sass H, Berchtold M, Branke J, König H, Cypionka H, Babenzien HD. Psychrotolerant sulfate-reducing bacteria from an oxic freshwater sediment description of *Desulfovibrio cuneatus* sp. nov. and *Desulfovibrio litoralis* sp. nov. Syst Appl Microbiol. 1998;21(2):212–219.

41. Vivier D, Rivemale M, Reverbel JP, Ratomahenina R, Galzy P. Study of the growth of yeasts from feta cheese. Int J Food Microbiol. 1994;22(2):207–215.

42. Lai MC, Chen SC. *Methanofollis aquaemaris* sp. nov., a methanogen isolated from an aquaculture fish pond. Int J Syst Evol Microbiol. 2001;51(5):1873–1880.

43. Boltyanskaya YV, Kevbrin V, Lysenko AM, Kolganova TV, Tourova TP, Osipov GA, et al. *Halomonas mongoliensis* sp. nov. and *Halomonas kenyensis* sp. nov., new haloalkaliphilic denitrifiers capable of N2O reduction, isolated from soda lakes. Microbiology. 2007;76(6):739–747.

44. Dianou D, Miyaki T, Asakawa S, Morii H, Nagaoka K, Oyaizu H, et al. *Methanoculleus chikugoensis* sp. nov., a novel methanogenic archaeon isolated from paddy field soil in Japan, and DNA-DNA hybridization among *Methanoculleus* species. Int J Syst Evol Microbiol. 2001;51(5):1663–1669.

45. Zellner G, Messner P, Winter J, Stackebrandt E. *Methanoculleus palmolei* sp. nov., an irregularly coccoid methanogen from an anaerobic digester treating wastewater of a palm oil plant in North-Sumatra, Indonesia. Int J Syst Bacteriol. 1998;48(4):1111–1117.

46. Takai K, Moyer CL, Miyazaki M, Nogi Y, Hirayama H, Nealson KH, et al. *Marinobacter alkaliphilus* sp. nov., a novel alkaliphilic bacterium isolated from subseafloor alkaline serpentine mud from Ocean Drilling Program Site 1200 at South Chamorro Seamount, Mariana Forearc. Extremophiles. 2005;9(1):17–27.

47. Wilson KH, Sheagren JN, Freter R. Population dynamics of ingested *Clostridium difficile* in the gastrointestinal tract of the Syrian hamster. J Infect Dis. 1985;151(2):355–361.

48. Tang EPY, Tremblay R, Vincent WF. Cyanobacterial dominance of polar freshwater ecosystems: are high-latitude mat-formers adapted to low temperature. J Phycol. 1997;33(2):171–181.

49. Ramalho FS, Malaquias JB, Lira ACS, Oliveira FQ, Zanuncio JC, Fernandes FS. Temperature-dependent fecundity and life table of the fennel aphid *Hyadaphis foeniculi* (Passerini) (Hemiptera: Aphididae). PLOS ONE. 2015;10(4):e0122490.

50. Trinci APJ. A kinetic study of the growth of *Aspergillus nidulans* and other fungi. J Gen Microbiol. 1969;57(1):11–24.

51. Trinci APJ. A study of the kinetics of hyphal extension and branch initiation of fungal mycelia. J Gen Microbiol. 1974;81(1):225–236.

52. Steele GC, Trinci APJ. Morphology and growth kinetics of hyphae of differentiated and undifferentiated mycelia of *Neurospora crassa*. J Gen Intern Med. 1975;91(2):362–368.

53. Camberlein E, Cohen JM, José R, Hyams CJ, Callard R, Chimalapati S, et al. Importance of bacterial replication and alveolar macrophage-independent clearance mechanisms during early lung infection with *Streptococcus pneumoniae*. Infect Immun. 2015;83(3):1181–1189.

54. Rebah FB, Frikha F, Kamoun W, Belbahri L, Gargouri Y, Miled N. Culture of *Staphylococcus xylosus* in fish processing by-product-based media for lipase production. Lett Appl Microbiol. 2008;47(6):549–554.

55. Sakai M, Deguchi D, Hosoda A, Kawauchi T, Ikenaga M. *Ammoniibacillus agariperforans* gen. nov., sp. nov., a thermophilic, agar-degrading bacterium isolated from compost. Int J Syst Evol Microbiol. 2015;65(Pt 2):570–577.

56. Sorokin DY, Kovaleva OL, Tourova TP, Muyzer G. *Thiohalobacter thiocyanaticus* gen. nov., sp. nov., a moderately halophilic, sulfur-oxidizing gammaproteobacterium from hypersaline lakes, that utilizes thiocyanate. Int J Syst Evol Microbiol. 2010;60:444–450.

57. Garner MR, Flint JF, Russell JB. *Allisonella histaminiformans* gen. nov., sp. nov.: A novel bacterium that produces histamine, utilizes histidine as its sole energy source, and could play a role in bovine and equine laminitis. Syst Appl Microbiol. 2002;25(4):498–506.

58. Watanabe I, Okada S. Effects of temperature on growth rate of cultured mammalian cells (L5178Y). J Cell Biol. 1967;32(2):309–323.

59. Corder RE, Hook LA, Larkin JM, Frea JI. Isolation and characterization of two new methane-producing cocci: *Methanogenium olentangi*, sp. nov., and *Methanococcus deltae*, sp. nov. Arch Microbiol. 1983;134(1):28–32.

60. Oren A, Weisburg WG, Kessel M, Woese CR. *Halobacteroides halobius* gen. nov., sp. nov., a moderately halophic anaerobic bacterium from the bottom sediments of the Dead Sea. Syst Appl Microbiol. 1984;5(1):58–70.

61. Ingraham JL. Growth of psychrophilic Bacteria. J Bacteriol. 1958;76(1):75–80.

62. Meeks JC, Castenholz RW. Growth and photosynthesis in an extreme thermophile, *Synechococcus lividus* (Cyanophyta). Arch Mikrobiol. 1971;78:25–41.

63. Espejo RT, Canelo ES. Properties and characterization of the host bacterium of bacteriophage PM2. J Bacteriol. 1968;95(5):1887–1891.

64. Feil H, Purcell AH. Temperature-dependent growth and survival of *Xylella fastidiosa* in vitro and in potted grapevines. Plant Dis. 2001 December;85(12):1230–1234. Refer to ID485.

65. Brosseau JD, Margaritis A, Zajic JE. The effect of temperature on the growth and hydrogen production by *Citrobacter intermedius.* Biol Lett. 1982;4(5):307–312.

66. Oren A, Pohla H, Stackebrandt E. Transfer of *Clostridium lortetii* to a new genus *Sporohalobacter* gen. nov. as *Sporohalobacter lortetii* comb. nov., and description of *Sporohalobacter marismortui* sp. nov. Syst Appl Microbiol. 1987;9(3):239–246.

67. Rengpipat S, Langworthy TA, Zeikus JG. *Halobacteroides acetoethylicus* sp. nov., a new obligately anaerobic halophile isolated from deep subsurface hypersaline environments. Syst Appl Microbiol. 1988;11(1):28–35.

68. Oren A. *Clostridium lortetii* sp. nov., a halophilic obligatory anaerobic bacterium producing endospores with attached gas vacuoles. Arch Microbiol. 1983;136(1):42–48.

69. Zhilina T, Zavarzin G, Bulygina E, Kevbrin V, Osipov G, Chumakov K. Ecology, physiology and taxonomy studies on a new taxon of Haloanaerobiaceae, *Haloincola saccharolytica* gen. nov., sp. nov. Syst Appl Microbiol. 1992;15(2):275–284.

70. Jahnke J. The light and temperature dependence of growth rate and elemental composition of *Phaeocystis globosa* Scherffel and *P. Pouchetii* (HAR.) Lagerh. in batch cultures. Neth J Sea Res. 1989;23(1):15–21.

71. Ollivier B, Cayol JL, Patel BKC, Magot M, Fardeau ML, Garcia JL. *Methanoplanus petrolearius* sp. nov., a novel methanogenic bacterium from an oil-producing well. FEMS Microbiol Ecol. 1997;147(1):51–56.

72. Aspaly G, Stejskal V, Pekár S, Hubert J. Temperature-dependent population growth of three species of stored product mites (Acari: Acaridida). Exp Appl Acarol. 2007;42(4):37–46.

73. Cheng L, Qiu TL, Li X, Wang WD, Deng Y, Yin XB, et al. Isolation and characterization of *Methanoculleus receptaculi* sp. nov. from Shengli oil field, China. FEMS Microbiol Ecol. 2008;285(1):65–71.

74. Zeikus J, Lynd LH, Thompson T, Krzycki J, Weimer P, Hegge P. Isolation and characterization of a new, methylotrophic, acidogenic anaerobe, the Marburg strain. Curr Microbiol. 1980;3(6):381–386.

75. Cheng L, Qiu TL, Yin XB, Wu XL, Hu GQ, Deng Y, et al. *Methermicoccus shengliensis* gen. nov., sp. nov., a thermophilic, methylotrophic methanogen isolated from oil-production water, and proposal of Methermicoccaceae fam. nov. Int J Syst Evol Microbiol. 2007;57(12):2964–2969.

76. Blotevogel KH, Gahl-Janßen R, Jannsen S, Fischer U, Pilz F, Auling G, et al. Isolation and characterization of a novel mesophilic, fresh-water methanogen from river sediment *Methanoculleus oldenburgensis* sp. nov. Arch Microbiol. 1991;157(1):54–59.

77. Liu YH, Tsai JH. Effects of temperature on biology and life table parameters of the Asian citrus psyllid, *Diaphorina citri* Kuwayama (Homoptera: Psyllidae). Ann Appl Biol. 2000;137(3):201–206.

78. Cayol JL, Ollivier B, Soh ALA, Fardeau ML, Ageron E, Grimont P, et al. *Haloincola saccharolytica* subsp. *senegalensis* subsp. nov., isolated from the sediments of a hypersaline lake, and emended description of *Haloincola saccharolytica*. Int J Syst Bacteriol. 1994;44(4):805–811.

79. Denner EBM, McGenity TJ, Busse HJ, Grant WD, Wanner G, Stan-Lotter H. *Halococcus salifodinae* sp. nov., an archaeal isolate from an Austrian salt mine. Int J Syst Bacteriol. 1994;44(4):774–780.

80. Cayol JL, Ollivier B, Patel BKC, Prensier G, Guezennec J, Garcia JL. Isolation and characterization of *Halothermothrix orenii* gen. nov., sp. nov., a halophilic, thermophilic, fermentative, strictly anaerobic bacterium. Int J Syst Bacteriol. 1994;44(3):534–540.

81. Baker JH. The use of a temperature-gradient incubator to investigate the temperature characteristics of some bacteria from Antarctic peat. Br Antarct Surv Bull. 1974;39:49–59.

82. Ohye DF, Scott WJ. The temperature relations of *Clostridium botulinum*, types A and B. Aust J Biol Sci. 1953;6(2):178–189.

83. Knoblauch C, Jorgensen BB. Effect of temperature on sulphate reduction, growth rate and growth yield in five psychrophilic sulphate-reducing bacteria from Arctic sediments. Environ Microbiol. 1999;1(5):457–467.

84. Bekada AMA, Benakriche B, Hamadi K, Bensoltane A. Modelling of effects of water activity, pH and temperature on the growth rate of *Mucor racemosus* isolated from soft camembert cheese. World J Agric Sci. 2008;4(6):790–4.

85. Mohr PW, Krawiec S. Temperature characteristics and Arrhenius plots for nominal psychrophiles, mesophiles and thermophiles. J Gen Microbiol. 1980;121(2):311–317.

86. Moore LR, Goericke R, Chisholm SW. Comparative physiology of *Synechococcus* and *Prochlorococcus*: influence of light and temperature on growth, pigments, fluorescence and absorptive properties. Mar Ecol Prog Ser. 1995;116(1):259–275.

87. Nunoura T, Hirai M, Miyazaki M, Kazama H, Makita H, Hirayama H, et al. Isolation and characterization of a thermophilic, obligately anaerobic and heterotrophic marine *Chloroflexi* bacterium from a *Chloroflexi*-dominated microbial community associated with a Japanese shallow hydrothermal system, and proposal for *Thermomarinilinea lacunofontalis* gen. nov., sp. nov. Microbes Environ. 2013;28(2):228–235.

88. Nunoura T, Oida H, Miyazaki M, Suzuki Y, Takai K, Horikoshi K. *Marinitoga okinawensis* sp. nov., a novel thermophilic and anaerobic heterotroph isolated from a deep-sea hydrothermal field, Southern Okinawa Trough. Int J Syst Evol Microbiol. 2007;57(3):467–471.

89. Jayasinghearachchi HS, Lal B. *Oceanotoga teriensis* gen. nov., sp. nov., a thermophilic bacterium isolated from offshore oil-producing wells. Int J Syst Evol Microbiol. 2011;61(3):554–560.

90. Postec A, Le Breton C, Fardeau ML, Lesongeur F, Pignet P, Querellou J, et al. *Marinitoga hydrogenitolerans* sp. nov., a novel member of the

order Thermotogales isolated from a black smoker chimney on the Mid-Atlantic Ridge. Int J Syst Evol Microbiol. 2005;55(3):1217–1221.

91. Postec A, Ciobanu M, Birrien JL, Bienvenu N, Prieur D, Le Romancer M. *Marinitoga litoralis* sp. nov., a thermophilic, heterotrophic bacterium isolated from a coastal thermal spring on Ile Saint-Paul, Southern Indian Ocean. Int J Syst Evol Microbiol. 2010;60(8):1778–1782.

92. Birrien JL, Zeng X, Jebbar M, Cambon-Bonavita MA, Quérellou J, Oger P, et al. *Pyrococcus yayanosii* sp. nov., an obligate piezophilic hyperthermophilic archaeon isolated from a deep-sea hydrothermal vent. Int J Syst Evol Microbiol. 2011;61(12):2827–2881.

93. Mori K, Yamazoe A, Hosoyama A, Ohji S, Fujita N, Ishibashi Ji, et al. *Thermotoga profunda* sp. nov. and *Thermotoga caldifontis* sp. nov., anaerobic thermophilic bacteria isolated from terrestrial hot springs. Int J Syst Evol Microbiol. 2014;64(Pt 6):2128–2136.

94. DiPippo JL, Nesbø CL, Dahle H, Doolittle WF, Birkland NK, Noll KM. *Kosmotoga olearia* gen. nov., sp. nov., a thermophilic, anaerobic heterotroph isolated from an oil production fluid. Int J Syst Evol Microbiol. 2009;59(12):2991–3000.

95. Zeikus J, Hegge P, Thompson T, Phelps T, Langworthy T. Isolation and description of *Haloanaerobium praevalens* gen. nov. and sp. nov., an obligately anaerobic halophile common to Great Salt Lake sediments. Curr Microbiol. 1983;9(4):225–233.

96. Nesbø CL, Bradnan DM, Adebusuyi A, Dlutek M, Petrus AK, Foght J, et al. *Mesotoga prima* gen. nov., sp. nov., the first described mesophilic species of the Thermotogales. Extremophiles. 2012;16(3):387–393.

97. Wery N, Lesongeur F, Pignet P, Derennes V, Cambon-Bonavita MA, Godfroy A, et al. *Marinitoga camini* gen. nov., sp. nov., a rod-shaped bacterium belonging to the order Thermotogales, isolated from a deep-sea hydrothermal vent. Int J Syst Evol Microbiol. 2001;51(2):495–504.

98. Blöchl E, Rachel R, Burggraf S, Hafenbradl D, Jannasch HW, Stetter KO. *Pyrolobus fumarii*, gen. and sp. nov., represents a novel group of archaea, extending the upper temperature limit for life to 113 C. Extremophiles. 1997;1(1):14–21.

99. Panagou EZ, Skandamis PN, Nychas GJE. Modelling the combined effect of temperature, pH and $a_w$ on the growth rate of *Monascus ruber*, a heat-resistant fungus isolated from green table olives. J Appl Microbiol. 2003;94:146–156.

100. L'Haridon S, Jiang L, Alain K, Chalopin M, Rouxel O, Beauverger M, et al. *Kosmotoga pacifica* sp. nov., a thermophilic

chemoorganoheterotrophic bacterium isolated from an East Pacific hydrothermal sediment. Extremophiles. 2014;18(1):81–88.

101. Foesel BU, Gößner AS, Drake HL, Schramm A. *Geminicoccus roseus* gen. nov., sp. nov., an aerobic phototrophic Alphaproteobacterium isolated from a marine aquaculture biofilter. Syst Appl Microbiol. 2007;30(8):581–586.

102. Toda Y, Saiki T, Uozumi T, Beppu T. Isolation and characterization of a protease-producing, thermophilic, anaerobic bacterium, *Thermobacteroides leptospartum* sp. nov. Agric Biol Chem. 1988;52(6):1339–1344.

103. Kublanov IV, Prokofeva MI, Kostrikina NA, Kolganova TV, Tourova TP, Wiegel J, et al. *Thermoanaerobacterium aciditolerans* sp. nov., a moderate thermoacidophile from a Kamchatka hot spring. Int J Syst Evol Microbiol. 2007;57(2):260–264.

104. Moriya T, Hikota T, Yumoto I, Ito T, Terui Y, Yamagishi A, et al. *Calditerricola satsumensis* gen. nov., sp. nov. and *Calditerricola yamamurae* sp. nov., extreme thermophiles isolated from a high-temperature compost. Int J Syst Evol Microbiol. 2011;61(3):631–636.

105. Horn MA, Ihssen J, Matthies C, Schramm A, Acker G, Drake HL. *Dechloromonas denitrificans* sp. nov., *Flavobacterium denitrificans* sp. nov., *Paenibacillus anaericanus* sp. nov. and *Paenibacillus terrae* strain MH72, N2O-producing bacteria isolated from the gut of the earthworm *Aporrectodea caliginosa*. Int J Syst Evol Microbiol. 2005;55(3):1255–1265.

106. Morandi S, Brasca M, Alfieri P, Lodi R, Tamburini A. Influence of pH and temperature on the growth of *Enterococcus faecium* and *Enterococcus faecalis*. Le Lait. 2005;85(3):181–192.

107. Restaino L, Bills S, Tscherneff K, Lenovich LM. Growth characteristics of *Saccharomyces rouxii* isolated from chocolate syrup. Appl Environ Microbiol. 1983;45(5):1614–1621.

108. Suetin SV, Shcherbakova VA, Chuvilskaya NA, Rivkina EM, Suzina NE, Lysenko AM, et al. *Clostridium tagluense* sp. nov., a psychrotolerant, anaerobic, spore-forming bacterium from permafrost. Int J Syst Evol Microbiol. 2009;59(6):1421–1426.

109. Jeong H, Yi H, Sekiguchi Y, Muramatsu M, Kamagata Y, Chun J. *Clostridium jejuense* sp. nov., isolated from soil. Int J Syst Evol Microbiol. 2004;54(5):1465–1468.

110. Crespo C, Pozzo T, Karlsson EN, Alvarez MT, Mattiasson B. *Caloramator boliviensis* sp. nov., a thermophilic, ethanol-producing bacterium isolated from a hot spring. Int J Syst Evol Microbiol. 2012;62(Pt 7):1679–1686.

111. Bazylinski DA, Williams TJ, Lefèvre CT, Trubitsyn D, Fang J, Beveridge TJ, et al. *Magnetovibrio blakemorei* gen. nov., sp. nov., a magnetotactic bacterium (Alphaproteobacteria: Rhodospirillaceae) isolated from a salt marsh. Int J Syst Evol Microbiol. 2013;63(Pt 5):1824–1833.

112. Mori K, Suzuki Ki. *Thiofaba tepidiphila* gen. nov., sp. nov., a novel obligately chemolithoautotrophic, sulfur-oxidizing bacterium of the Gammaproteobacteria isolated from a hot spring. Int J Syst Evol Microbiol. 2008;58(8):1885–1891.

113. Butler H, Rogerson A. Growth potential, production efficiency and annual production of marine benthic naked amoebae(gymnamoebae) inhabiting sediments of the Clyde Sea area, Scotland. Aquat Microb Ecol. 1996;10(2):123–129.

114. Takai K, Miyazaki M, Nunoura T, Hirayama H, Oida H, Furushima Y, et al. *Sulfurivirga caldicuralii* gen. nov., sp. nov., a novel microaerobic, thermophilic, thiosulfate-oxidizing chemolithoautotroph, isolated from a shallow marine hydrothermal system occurring in a coral reef, Japan. Int J Syst Evol Microbiol. 2006;56(8):1921–1929.

115. McMeekin TA, Chandler RE, Doe PE, Garland CD, Olley J, Putro S, et al. Model for combined effect of temperature and salt concentration/water activity on the growth rate of *Staphylococcus xylosus*. J Appl Bacteriol. 1987;62(6):543–550.

116. Nelson CM, Schuppenhauer MR, Clark DS. Effects of hyperbaric pressure on a deep-sea archaebacterium in stainless steel and glass-lined vessels. Appl Environ Microbiol. 1991;57(12):3576–3580.

117. Liou JSC, Balkwill DL, Drake GR, Tanner RS. *Clostridium carboxidivorans* sp. nov., a solvent-producing clostridium isolated from an agricultural settling lagoon, and reclassification of the acetogen *Clostridium scatologenes* strain SL1 as *Clostridium drakei* sp. nov. Int J Syst Evol Microbiol. 2005;55(5):2085–2091.

118. Lee YJ, Romanek CS, Mills GL, Davis RC, Whitman WB, Wiegel J. *Gracilibacter thermotolerans* gen. nov., sp. nov., an anaerobic, thermotolerant bacterium from a constructed wetland receiving acid sulfate water. Int J Syst Evol Microbiol. 2006;56(9):2089–2093.

119. Tomás AF, Karakashev D, Angelidaki I. *Thermoanaerobacter pentosaceus* sp. nov., an anaerobic, extremely thermophilic, high ethanol-yielding bacterium isolated from household waste. Int J Syst Evol Microbiol. 2013;63(Pt 7):2396–2404.

120. Mesbah NM, Hedrick DB, Peacock AD, Rohde M, Wiegel J. *Natranaerobius thermophilus* gen. nov., sp. nov., a halophilic, alkalithermophilic bacterium from soda lakes of the Wadi An Natrun, Egypt, and proposal

of Natranaerobiaceae fam. nov. and Natranaerobiales ord. nov. Int J Syst Evol Microbiol. 2007;57:2507–2512.

121. Whitman WB, Shieh J, Sohn S, Caras DS, Premachandran U. Isolation and characterization of 22 mesophilic Methanococci. Syst Appl Microbiol. 1986;7(2):235–240.

122. Hedrich S, Johnson DB. *Acidithiobacillus ferridurans* sp. nov., an acidophilic iron-, sulfur-and hydrogen-metabolizing chemolithotrophic gammaproteobacterium. Int J Syst Evol Microbiol. 2013;63(Pt 11):4018–4025.

123. Kane MD, Brauman A, Breznak JA. *Clostridium mayombei* sp. nov., an H2/CO2 acetogenic bacterium from the gut of the African soil-feeding termite, *Cubitermes speciosus*. Arch Microbiol. 1991;156(2):99–104.

124. Kuhner CH, Matthies C, Acker G, Schmittroth M, Gößner AS, Drake HL. *Clostridium akagii* sp. nov. and *Clostridium acidisoli* sp. nov.: acid-tolerant, N2-fixing clostridia isolated from acidic forest soil and litter. Int J Syst Evol Microbiol. 2000;50(2):873–881.

125. Chahal DS, Hawksworth DL. *Chaetomium cellulolyticum*, a new thermotolerant and cellulolytic *Chaetomium* I. Isolation, description and growth rate. Mycologia. 1976;p. 600–610.

126. Schwartz RD, Keller FA. Isolation of a strain of *Clostridium thermoaceticum* capable of growth and acetic acid production at pH 4.5. Appl Environ Microbiol. 1982;43(1):117–123.

127. Bender J, Vatcharapijarn Y, Jeffries TW. Characteristics and adaptability of some new isolates of *Clostridium thermocellum*. Appl Environ Microbiol. 1985;49(3):475–477.

128. Mathrani IM, Boone DR. Isolation and characterization of a moderately halophilic methanogen from a solar saltern. Appl Environ Microbiol. 1985;50(1):140–143.

129. Greene RV, Freer SN. Growth characteristics of a novel nitrogen-fixing cellulolytic bacterium. Appl Environ Microbiol. 1986;52(5):982–986.

130. Klouche N, Fardeau ML, Lascourrèges JF, Cayol JL, Hacene H, Thomas P, et al. *Geosporobacter subterraneus* gen. nov., sp. nov., a spore-forming bacterium isolated from a deep subsurface aquifer. Int J Syst Evol Microbiol. 2007;57(8):1757–1761.

131. Allais JJ, Hoyos-Lopez G, Kammoun S, Baratti JC. Isolation and characterization of thermophilic bacterial strains with inulinase activity. Appl Environ Microbiol. 1987;53(5):942–945.

132. Beffa T, Blanc M, Lyon PF, Vogt G, Marchiani M, Fischer JL, et al. Isolation of *Thermus* strains from hot composts (60 to 80 degrees C). Appl Environ Microbiol. 1996;62(5):1723–1727.

133. Kamlage B, Gruhl B, Blaut M. Isolation and characterization of two new homoacetogenic hydrogen-utilizing bacteria from the human intestinal tract that are closely related to *Clostridium coccoides*. Appl Environ Microbiol. 1997;63(5):1732–1738.

134. Alain K, Querellou J, Lesongeur F, Pignet P, Crassous P, Raguénès G, et al. *Caminibacter hydrogeniphilus* gen. nov., sp. nov., a novel thermophilic, hydrogen-oxidizing bacterium isolated from an East Pacific Rise hydrothermal vent. Int J Syst Evol Microbiol. 2002;52(4):1317–1323.

135. Barbier G, Godfroy A, Meunier JR, Quérellou J, Cambon MA, Lesongeur F, et al. *Pyrococcus glycovorans* sp. nov., a hyperthermophilic archaeon isolated from the East Pacific Rise. Int J Syst Evol Microbiol. 1999;49(4):1829–1837.

136. Jannasch HW, Wirsen CO, Molyneaux SJ, Langworthy TA. Extremely thermophilic fermentative archaebacteria of the genus Desulfurococcus from deep-sea hydrothermal vents. Appl Environ Microbiol. 1988;54(5):1203–1209.

137. Wery N, Moricet JM, Cueff V, Jean J, Pignet P, Lesongeur F, et al. *Caloranaerobacter azorensis* gen. nov., sp. nov., an anaerobic thermophilic bacterium isolated from a deep-sea hydrothermal vent. Int J Syst Evol Microbiol. 2001;51(5):1789–1796.

138. Sanford RA, Cole JR, Tiedje JM. Characterization and description of *Anaeromyxobacter dehalogenans* gen. nov., sp. nov., an arylhalorespiring facultative anaerobic myxobacterium. Appl Environ Microbiol. 2002;68(2):893–900.

139. Abboud R, Popa R, Souza-Egipsy V, Giometti CS, Tollaksen S, Mosher JJ, et al. Low-temperature growth of *Shewanella oneidensis* MR-1. Appl Environ Microbiol. 2005;71(2):811–816.

140. Gotoh T, Sugimoto N, Pallini A, Knapp M, Hernandez-Suarez E, Ferragut F, et al. Reproductive performance of seven strains of the tomato red spider mite *Tetranychus evansi* (Acari: Tetranychidae) at five temperatures. Exp Appl Acarol. 2010;52(3):239–259.

141. Traunecker J, Preuß A, Diekert G. Isolation and characterization of a methyl chloride utilizing, strictly anaerobic bacterium. Arch Microbiol. 1991;156(5):416–421.

142. Dubinina G, Grabovich M, Leshcheva N, Rainey FA, Gavrish E. *Spirochaeta perfilievii* sp. nov., an oxygen-tolerant, sulfide-oxidizing, sulfur-and thiosulfate-reducing spirochaete isolated from a saline spring. Int J Syst Evol Microbiol. 2011;61(1):110–117.

143. Hoover RB, Pikuta EV, Bej AK, Marsic D, Whitman WB, Tang J, et al. *Spirochaeta americana* sp. nov., a new haloalkaliphilic, obligately anaerobic spirochaete isolated from soda Mono Lake in California. Int J Syst Evol Microbiol. 2003;53(3):815–821.

144. Imachi H, Sakai S, Hirayama H, Nakagawa S, Nunoura T, Takai K, et al. *Exilispira thermophila* gen. nov., sp. nov., an anaerobic, thermophilic spirochaete isolated from a deep-sea hydrothermal vent chimney. Int J Syst Evol Microbiol. 2008;58(10):2258–2265.

145. Breznak JA, Warnecke F. *Spirochaeta cellobiosiphila* sp. nov., a facultatively anaerobic, marine spirochaete. Int J Syst Evol Microbiol. 2008;58(12):2762–2768.

146. Dröge S, Fröhlich J, Radek R, König H. *Spirochaeta coccoides* sp. nov., a novel coccoid spirochete from the hindgut of the termite *Neotermes castaneus*. Appl Environ Microbiol. 2006;72(1):392–397.

147. Zaĭtseva SV, Kozyreva LP, Hamsaraev BB. The effect of temperature and pH on the growth of aerobic alkalithermophilic bacteria from hot springs in Buryatia. Mikrobiologiia. 2004;73(4):443–448.

148. Nevot M, Deroncelé V, Montes M, Mercade E. Effect of incubation temperature on growth parameters of *Pseudoalteromonas antarctica* NF3 and its production of extracellular polymeric substances. J Appl Microbiol. 2008;105(1):255–263. See ID511.

149. Pohlschroeder M, Leschine SB, Canale-Parola E. *Spirochaeta caldaria* sp. nov., a thermophilic bacterium that enhances cellulose degradation by *Clostridium thermocellum*. Arch Microbiol. 1994;161(1):17–24.

150. Kane MD, Breznak JA. *Acetonema longum* gen. nov. sp. nov., an H2/CO2 acetogenic bacterium from the termite, *Pterotermes occidentis*. Arch Microbiol. 1991;156(2):91–98.

151. Mountfort DO, Rainey FA, Burghardt J, Stackebrandt E. *Clostridium grantii* sp. nov., a new obligately anaerobic, alginolytic bacterium isolated from mullet gut. Arch Microbiol. 1994;162(3):173–179.

152. Kimble LK, Mandelco L, Woese CR, Madigan MT. *Heliobacterium modesticaldum*, sp. nov., a thermophilic heliobacterium of hot springs and volcanic soils. Arch Microbiol. 1995;163(4):259–267.

153. Bonjour F, Aragno M. *Bacillus tusciae*, a new species of thermoacidophilic, facultatively chemolithoautotrophic hydrogen oxidizing sporeformer from a geothermal area. Arch Microbiol. 1984;139(4):397–401.

154. Beuscher HU, Andreesen JR. *Eubacterium angustum* sp. nov., a Gram-positive anaerobic, non-sporeforming, obligate purine fermenting organism. Arch Microbiol. 1984;140(1):2–8.

155. Fiala G, Woese CR, Langworthy TA, Stetter KO. *Flexistipes sinusarabici*, a novel genus and species of eubacteria occurring in the Atlantis II Deep brines of the Red Sea. Arch Microbiol. 1990;154(2):120–126.

156. Patel BKC, Morgan HW, Daniel RM. *Fervidobacterium nodosum* gen. nov. and spec. nov., a new chemoorganotrophic, caldoactive, anaerobic bacterium. Arch Microbiol. 1985;141(1):63–69.

157. Brysch K, Schneider C, Fuchs G, Widdel F. Lithoautotrophic growth of sulfate-reducing bacteria, and description of *Desulfobacterium autotrophicum* gen. nov., sp. nov. Arch Microbiol. 1987;148(4):264–274.

158. Hallberg KB, González-Toril E, Johnson DB. *Acidithiobacillus ferrivorans*, sp. nov.; facultatively anaerobic, psychrotolerant iron-, and sulfur-oxidizing acidophiles isolated from metal mine-impacted environments. Extremophiles. 2010;14(1):9–19.

159. Zhilina T, Kevbrin V, Tourova T, Lysenko A, Kostrikina N, Zavarzin G. *Clostridium alkalicellum* sp. nov., an obligately alkaliphilic cellulolytic bacterium from a soda lake in the Baikal region. Microbiology. 2005;74(5):557–566.

160. Rhee SK, Jeon C, Bae JW, Kim K, Song J, Kim JJ, et al. Characterization of *Symbiobacterium toebii*, an obligate commensal thermophile isolated from compost. Extremophiles. 2002;6(1):57–64.

161. Schink B. *Clostridium magnum* sp. nov., a non-autotrophic homoacetogenic bacterium. Arch Microbiol. 1984;137(3):250–255.

162. Browne RA, Wanigasekera G. Combined effects of salinity and temperature on survival and reproduction of five species of *Artemia*. J Exp Mar Biol Ecol. 2000;244(1):29–44.

163. Bowman JP, Nichols DS, McMeekin TA. *Psychrobacter glacincola* sp. nov., a halotolerant, psychrophilic bacterium Isolated from Antarctic sea ice. Syst Appl Microbiol. 1997;20(2):209–215.

164. Derikx P, De Jong GAH, Op den Camp HJM, Van der Drift C, Van Griensven LJLD, Vogels GD. Isolation and characterization of thermophilic methanogenic bacteria from mushroom compost. FEMS Microbiol Ecol. 1989;62(4):251–258.

165. Schnürer A, Schink B, Svensson BH. *Clostridium ultunense* sp. nov., a mesophilic bacterium oxidizing acetate in syntrophic association with a hydrogenotrophic methanogenic bacterium. Int J Syst Bacteriol. 1996;46(4):1145–1152.

166. Dürre P, Andersch W, Andreesen JR. Isolation and characterization of an adenine-utilizing, anaerobic sporeformer, *Clostridium purinolyticum* sp. nov. Int J Syst Bacteriol. 1981;31(2):184–194.

167. Plugge CM, Zoetendal EG, Stams AJM. *Caloramator coolhaasii* sp. nov., a glutamate-degrading, moderately thermophilic anaerobe. Int J Syst Evol Microbiol. 2000;50(3):1155–1162.

168. Tanner RS, Miller LM, Yang D. *Clostridium ljungdahlii* sp. nov., an acetogenic species in clostridial rRNA homology group I. Int J Syst Bacteriol. 1993;43(2):232–236.

169. Küsel K, Dorsch T, Acker G, Stackebrandt E, Drake HL. *Clostridium scatologenes* strain SL1 isolated as an acetogenic bacterium from acidic sediments. Int J Syst Evol Microbiol. 2000;50(2):537–546.

170. Macy JM, Nunan K, Hagen KD, Dixon DR, Harbour PJ, Cahill M, et al. *Chrysiogenes arsenatis* gen. nov., sp. nov., a new arsenate-respiring bacterium isolated from gold mine wastewater. Int J Syst Bacteriol. 1996;46(4):1153–1157.

171. Prowe SG, Antranikian G. *Anaerobranca gottschalkii* sp. nov., a novel thermoalkaliphilic bacterium that grows anaerobically at high pH and temperature. Int J Syst Evol Microbiol. 2001;51(2):457–465.

172. Bhupathiraju VK, Oren A, Sharma PK, Tanner RS, Woese CR, McInerney MJ. *Haloanaerobium salsugo* sp. nov., a moderately halophilic, anaerobic bacterium from a subterranean brine. Int J Syst Bacteriol. 1994;44(3):565–572.

173. Schoolfield RM, Sharpe PJH, Magnuson CE. Non-linear regression of biological temperature-dependent rate models based on absolute reaction-rate theory. J Theor Biol. 1981;88(4):719–731.

174. Seyfried M, Lyon D, Rainey FA, Wiegel J. *Caloramator viterbensis* sp. nov., a novel thermophilic, glycerol-fermenting bacterium isolated from a hot spring in Italy. Int J Syst Evol Microbiol. 2002;52(4):1177–1184.

175. Urios L, Intertaglia L, Lesongeur F, Lebaron P. *Haliea rubra* sp. nov., a member of the Gammaproteobacteria from the Mediterranean Sea. Int J Syst Evol Microbiol. 2009;59(5):1188–1192.

176. Shi P, Li BL, Ge F. Intrinsic optimum temperature of the diamondback moth and its ecological meaning. Environ Entomol. 2012;41(3):714–722.

177. Urios L, Intertaglia L, Lesongeur F, Lebaron P. *Haliea salexigens* gen. nov., sp. nov., a member of the Gammaproteobacteria from the Mediterranean Sea. Int J Syst Evol Microbiol. 2008;58(5):1233–1237.

178. Urios L, Intertaglia L, Lesongeur F, Lebaron P. *Balneola alkaliphila* sp. nov., a marine bacterium isolated from the Mediterranean Sea. Int J Syst Evol Microbiol. 2008;58(6):1288–1291.

179. Crespo-Medina M, Chatziefthimiou A, Cruz-Matos R, Pérez-Rodríguez I, Barkay T, Lutz RA, et al. *Salinisphaera hydrothermalis* sp. nov., a mesophilic, halotolerant, facultatively autotrophic, thiosulfate-oxidizing gammaproteobacterium from deep-sea hydrothermal vents, and emended description of the genus *Salinisphaera.* Int J Syst Evol Microbiol. 2009;59(6):1497–1503.

180. Chen KW, He YR, Lü YQ, Huang XLSS. Effect of temperature on the population parameters of *Trichogrammatoidea bactrae* Nagaraja. Acta Ecol Sinica. 2005;25(1):73–77.

181. Wang JJ, Tsai JH. Effect of temperature on the biology of *Aphis spiraecola* (Homoptera: Aphididae). Ann Entomol Soc Am. 2000;93(4):874–883.

182. Tsoukanas VI, Papadopoulos GD, Fantinou AA, Papadoulis GT. Temperature-dependent development and life table of *Iphiseius degenerans* (Acari: Phytoseiidae). Environ Entomol. 2006;35(2):212–218.

183. Sandhu HS, Nuessly GS, Webb SE, Cherry RH, Gilbert RA. Life table dtudies of *Elasmopalpus lignosellus* (Lepidoptera:Pyralidae) on sugarcane. Environ Entomol. 2010;39(6):2025–2032.

184. Pakyari H, Fathipour Y, Enkegaard A. Effect of temperature on life table parameters of predatory thrips *Scolothrips longicornis* (Thysanoptera: Thripidae) fed on twospotted spider mites (Acari: Tetranychidae). J Econ Entomol. 2011;104(3):799–805.

185. Lee JH, Ahn JJ. Temperature effects on development, fecundity, and life table parameters of *Amblyseius womersleyi* (Acari: Phytoseiidae). Environ Entomol. 2000;29(2):265–271.

186. Puerta-Gomez AF, Moreira RG, Kim J, Castell-Perez E. Modeling the growth rates of *Escherichia coli* spp. and *Salmonella* Typhimurium LT2 in baby spinach leaves under slow cooling. Food Control. 2013;29(1):11–17.

187. Dreyer H, Baumgärtner J. Temperature influence on cohort parameters and demographic characteristics of the two cowpea coreids *Clavigralla tomentosicollis* and *C. shadabi.* Entomol Exp Appl. 1996;78:201–213.

188. Morgan D, Walters KFA, Aegerter JN. Effect of temperature and cultivar on pea aphid, *Acyrthosiphon pisum* (Hemiptera: Aphididae) life history. Bull Entomol Res. 2001;91:47–52.

189. Chen S, Dong X. *Acetanaerobacterium elongatum* gen. nov., sp. nov., from paper mill waste water. Int J Syst Evol Microbiol. 2004;54(6):2257–2262.

190. Takai K, Nakamura K, Toki T, Tsunogai U, Miyazaki M, Miyazaki J, et al. Cell proliferation at 122°C and isotopically heavy $CH_4$ production by a hyperthermophilic methanogen under high-pressure cultivation. Proc Natl Acad Sci USA. 2008;105(31):10949–10954.

191. Alain K, Pignet P, Zbinden M, Quillevere M, Duchiron F, Donval JP, et al. *Caminicella sporogenes* gen. nov., sp. nov., a novel thermophilic spore-forming bacterium isolated from an East-Pacific Rise hydrothermal vent. Int J Syst Evol Microbiol. 2002;52(5):1621–1628.

192. Lysyk TJ. Relationships between temperature and life history parameters of *Muscidifurax raptor* (Hymenoptera: Pteromalidae). Environ Entomol. 2000;29(3):596–605.

193. Stenseng L, Skovgård H, Holter P. Life table studies of the pupal parasitoid *Urolepis rufipes* (Hymenoptera: Pteromalidae) on the house fly *Musca domestica* (Diptera: Muscidae) in Denmark. Environ Entomol. 2003;32(4):717–725.

194. Lysyk TJ. Relationships between temperature and life history parameters of *Trichomalopsis sarcophagae* (Hymenoptera: Peteromalidae). Environ Entomol. 1998;27(2):488–498.

195. Broda DM, Lawson PA, Bell RG, Musgrave DR. *Clostridium frigidicarnis* sp. nov., a psychrotolerant bacterium associated with 'blown pack' spoilage of vacuum-packed meats. Int J Syst Evol Microbiol. 1999;49(4):1539–1550.

196. Ydergaard S, Enkegaard A, Brødsgaard HF. The predatory mite *Hypoaspis miles*: temperature dependent life table characteristics on a diet of sciarid larvae, *Bradysia paupera* and *B. tritici*. Entomol Exp Appl. 1997;85(2):177–187.

197. Pervez A, Omkar. Temperature-dependent life attributes of an aphidophagous ladybird, *Propylea dissecta*. Biocontrol Sci Techn. 2004;14(6):587–594.

198. Roy M, Brodeur J, Cloutier C. Effect of temperature on intrinsic rates of natural increase ($r_m$) of a coccinellid and its spider mite prey. BioControl. 2003;48:57–72.

199. Dreyer BS, Neuenschwander P, Bouyjou B, Baumgärtner J, Dorn S. The influence of temperature on the life table of Hyperaspis notata. Entomol Exp Appl. 1997;84(1):85–92.

200. Zamani AA, Talebi AA, Fathipour Y, Baniameri V. Effect of temperature on biology and population growth parameters of *Aphis gossypii* Glover (Hom., Aphididae) on greenhouse cucumber. J Appl Entomol. 2006;130(8):453–460.

201. Kocourek F, Havelka J, Beránková J, Jarošik V. Effect of temperature on development rate and intrinsic rate of increase of *Aphis gossypii* reared on greenhouse cucumbers. Entomol Exp Appl. 1994;71(1):59–64.

202. Taghizadeh R, Fathipour Y, Kamali K. Influence of temperature on life-table parameters of *Stethorus gilvifrons* (Mulsant)(Coleoptera: Coccinellidae) fed on *Tetranychus urticae* Koch. J Appl Entomol. 2008;132(8):638–645.

203. Wang JJ, Tsai JH. Development, survival and reproduction of black citrus aphid, *Toxoptera aurantii* (Hemiptera: Aphididae), as a function of temperature. Bull Entomol Res. 2001;91:477–487.

204. Kontodimas DC, Milonas PG, Stathas GJ, Economou LP, Kavallieratos NG. Life table parameters of the pseudococcid predators *Nephus includens* and *Nephus bisignatus* (Coleoptera: Coccinelidae). Eur J Entomol. 2007;104(3):407–415.

205. Wang J, Zhao Z, Li L. Studies on bionomics of *Liposcelis entomophila* (Psocoptera:Liposcelididae) infesting stored product. Entomol Sinica. 1998;5(2):149–158.

206. Satar S, Kersting U, Uygun N. Effect of temperature on development and fecundity of *Aphis gossypii* Glover (Homoptera: Aphididae) on cucumber. J Pest Sci. 2005;78(3):133–137.

207. Jaramillo J, Chabi-Olaye A, Kamonjo C, Jaramillo A, Vega FE, Poehling HM, et al. Thermal tolerance of the coffee berry borer *Hypothenemus hampei*: predictions of climate change impact on a tropical insect pest. PLOS ONE. 2009;4(8):e6487.

208. De Moraes GJ, McMurtry JA. Effect of temperature and sperm supply on the reproductive potential of *Tetranychus evansi* (Acari: Tetranychidae). Exp Appl Acarol. 1987;3(2):95–107.

209. Broufas GD, Koveos DS. Development, survival and reproduction of *Euseius finlandicus* (Acari: Phytoseiidae) at different constant temperatures. Exp Appl Acarol. 2001;25:441–460.

210. Sánchez-Ramos I, Castañera P. Effect of temperature on reproductive parameters and longevity of *Tyrophagus putrescentiae* (Acari: Acaridae). Exp Appl Acarol. 2005;36(1-2):93–105.

211. Enkegaard A. The poinsettia strain of the cotton whitefly, *Bemisia tabaci* (Homoptera: Aleyrodidae), biological and demographic parameters on poinsettia (*Euphorbia pulcherrima*) in relation to temperature. Bull Entomol Res. 1993;83(04):535–546.

212. Gaum WG, Giliomee JH, Pringle KL. Life history and life tables of western flower thrips, *Frankliniella occidentalis* (Thysanoptera: Thripidae), on English cucumbers. Bull Entomol Res. 1994;84:219–224.

213. Aldyhim YN, Khalil AF. Influence of temperature and daylength on population development of *Aphis gossypii* on *Cucurbita pepo*. Entomol Exp Appl. 1993;67(2):167–172.

214. Hansen LS, Skovgård H, Hell K. Life table study of *Sitotroga cerealella* (Lepidoptera: Gelichiidae), a strain from West Africa. J Econ Entomol. 2004;97(4):1484–1490.

215. Ritalahti KM, Justicia-Leon SD, Cusick KD, Ramos-Hernandez N, Rubin M, Dornbush J, et al. *Sphaerochaeta globosa* gen. nov., sp. nov. and *Sphaerochaeta pleomorpha* sp. nov., free-living, spherical spirochaetes. Int J Syst Evol Microbiol. 2012;62(1):210–216.

216. Tsai JH, Wang K. Life table study of brown citrus aphid (Homoptera: Aphididae) at different temperatures. Environ Entomol. 1999;28(3):412–419.

217. Choi WI, Ryoo MI, Kim JG. Biology of *Paronychiurus kimi* (Collembola: Onychiuridae) under the influence of temperature, humidity and nutrition. Pedobiologia. 2002;46(6):548–557.

218. Davis JA, Radcliffe EB, Ragsdale DW. Effects of high and fluctuating temperatures on *Myzus persicae* (Hemiptera: Aphididae). Environ Entomol. 2006;35(6):1461–1468.

219. Jiang HB, Liu JC, Wang ZY, Wang JJ. Temperature-dependent development and reproduction of a novel stored product psocid, *Liposcelis badia* (Psocoptera: Liposcelididae). Environ Entomol. 2008;37(5):1105–1112.

220. Jandricic SE, Wraight SP, Bennett KC, Sanderson JP. Developmental times and life table statistics of *Aulacorthum solani* (Hemiptera: Aphididae) at six constant temperatures, with recommendations on the application of temperature-dependent development models. Environ Entomol. 2010;39(5):1631–1642.

221. Bayhan E, Ölmez-Bayhanb S, Ulusoy MR, Brown JK. Effect of temperature on the biology of *Aphis punicae* (Passerini) (Homoptera: Aphididae) on pomegranate. Environ Entomol. 2005;34(1):22–26.

222. Wang JJ, Tsai JH, Zhao ZM, Li LS. Development and reproduction of the psocid *Liposcelis bostrychophila* (Psocoptera: Liposcelididae) as a function of temperature. Ann Entomol Soc Am. 2000;93(2):261–270.

223. Milonas PG, Savopoulou-Soultani M. Development, survivorship, and reproduction of *Adoxophyes orana* (Lepidoptera: Tortricidae) at constant temperatures. Ann Entomol Soc Am. 2000;93(1):96–102.

224. Wang K, Tsai JH, Harrison NA. Influence of temperature on development, survivorship, and reproduction of buckthorn aphid (Homoptera: Aphididae). Ann Entomol Soc Am. 1997;90(1):62–68.

225. Yang TC, Chi H. Life tables and development of *Bemisia argentifolii* (Homoptera: Aleyrodidae) at different temperatures. J Econ Entomol. 2006;99(3):691–698.

226. Kuo MH, Lu WN, Chiu MC, Kuo YH, Hwang SH. Temperature-dependent development and population growth of *Tetraneura nigriabdominalis* (Homoptera: Pemphigidae) on three host plants. J Econ Entomol. 2006;99(4):1209–1213.

227. Wang JJ, Ren Y, Wei XQ, Dou W. Development, survival, and reproduction of the Psocid *liposcelis paeta* (psocoptera: liposcelididae) as a function of temperature. J Econ Entomol. 2009;102(4):1705–1713.

228. Domingos CA, Melo JWDS, Gondim Jr MG, De Moraes GJ, Hanna R, Lawson-Balagbo LM, et al. Diet-dependent life history, feeding preference and thermal requirements of the predatory mite *Neoseiulus baraki* (Acari: Phytoseiidae). Exp Appl Acarol. 2010;50(3):201–215.

229. Hernandez-Eugenio G, Fardeau ML, Cayol JL, Patel BKC, Thomas P, Macarie H, et al. *Sporanaerobacter acetigenes* gen. nov., sp. nov., a novel acetogenic, facultatively sulfur-reducing bacterium. Int J Syst Evol Microbiol. 2002;52(4):1217–1223.

230. Kuhner CH, Frank C, Grießhammer A, Schmittroth M, Acker G, Gößner A, et al. *Sporomusa silvacetica* sp. nov., an acetogenic bacterium isolated from aggregated forest soil. Int J Syst Bacteriol. 1997;47(2):352–358.

231. Tarlera S, Muxí L, Soubes M, Stams AJ. *Caloramator proteoclasticus* sp. nov., a new moderately thermophilic anaerobic proteolytic bacterium. Int J Syst Bacteriol. 1997;47(3):651–656.

232. González JM, Kato C, Horikoshi K. *Thermococcus peptonophilus* sp. nov., a fast-growing, extremely thermophilic archaebacterium isolated from deep-sea hydrothermal vents. Arch Microbiol. 1995;164(3):159–164.

233. Kwak YS, Kobayashi T, Akiba T, Horikoshi K, Kim YB. A hyperthermophilic sulfur-reducing archaebacterium, *Thermococcus* sp. DT1331, isolated from a deep-sea hydrothermal vent. Biosci Biotech Bioch. 1995;59(9):1666–1669.

234. Grote R, Li L, Tamaoka J, Kato C, Horikoshi K, Antranikian G. *Thermococcus siculi* sp. nov., a novel hyperthermophilic archaeon isolated from a deep-sea hydrothermal vent at the Mid-Okinawa Trough. Extremophiles. 1999;3(1):55–62.

235. González JM, Masuchi Y, Robb FT, Ammerman JW, Maeder DL, Yanagibayashi M, et al. *Pyrococcus horikoshii* sp. nov., a hyperthermophilic archaeon isolated from a hydrothermal vent at the Okinawa Trough. Extremophiles. 1998;2(2):123–130.

236. Takai K, Inoue A, Horikoshi K. *Thermaerobacter marianensis* gen. nov., sp. nov., an aerobic extremely thermophilic marine bacterium from the 11000 m deep Mariana Trench. Int J Syst Evol Microbiol. 1999;49(2):619–628.

237. Benson JG. Biofilms in pasteurisers: investigating growth of *Streptococcus thermophilus B.Sc. (Honours) Thesis.* Hobart: University of Tasmania; 1996.

238. Franzmann PD, Haddad CM, Hawkes RB, Robertson WJ, Plumb JJ. Effects of temperature on the rates of iron and sulfur oxidation by selected bioleaching Bacteria and Archaea: Application of the Ratkowsky equation. Miner Eng. 2005;18(13–14):1304–1314.

239. Franzmann PD, Springer N, Ludwig W, Conway de Macario E, Rohde M. A methanogenic Archaeon from Ace Lake, Antarctica: *Methanococcoides burtonii* sp. nov. Syst Appl Microbiol. 1992;15:573–581.

240. Harder W, Veldkamp H. Competition of marine psychrophilic bacteria at low temperatures. Antonie van Leeuwenhoek. 1971;37(1):51–63.

241. Hayward LJ. Predictive microbiology of *Aeromonas hydrophila*: the effect of temperature and water activity on the growth of *Aeromonas hydrophila*, *B.Sc. (Honours) Thesis.* Hobart: University of Tasmania; 1990.

242. Krist K. Description and mechanisms of bacterial growth responses to water activity and compatible solutes, *PhD Thesis.* Hobart: University of Tasmania; 1997. Refer hochstein1984effect.

243. McMeekin TA, Franzmann PD. Effect of temperature on the growth rates of halotolerant and halophilic Bacteria isolated from Antarctic saline lakes. Polar Biol. 1988;8:281–285.

244. Mellefont L. Predictive model development and lag phase characterisation for applications in the meat industry, *PhD Thesis*. Hobart: University of Tasmania; 2000.

245. Neumeyer K. Modelling pseudomonad growth in milk and milk-based products, *MSc Thesis*. Hobart: University of Tasmania; 1995.

246. Nichols DS, Greenhill AR, Shadbolt CT, Ross T, McMeekin TA. Physicochemical parameters for the growth of sea ice bacteria *Glaciecola punicea* ACAM $611^{T}$ and *Gelidibacter* sp. Strain IC158. Appl Environ Microbiol. 1999;65:3757–3760.

247. Nichols DS, Olley J, Garda J, Brenner RR, McMeekin T. Effect of temperature and salinity stress on growth and lipid composition of *Shewanella gelidmarina*. Appl Environ Microbiol. 2000;66:2422–2429.

248. Nichols DS, Presser KA, Olley J, Ross T, McMeekin T. Variation of branched-chain fatty acids marks the normal physiological range for growth in *Listeria monocytogenes*. Appl Environ Microbiol. 2002;68:2809–2813.

249. Robinson JL, Pyzyna B, Atrasz RG, Henderson CA, Morrill KL, Burd AM, et al. Growth Kinetics of Extremely Halophilic Archaea (Family Halobacteriaceae) as Revealed by Arrhenius Plots. J Bacteriol. 2005 February;187(3):923–929.

250. Ross T. A philosophy for the development of kinetic models in predictive microbiology, *PhD Thesis*. Hobart: University of Tasmania; 1993.

251. Salter MA, Ross T, McMeekin TA. Applicability of a model for non-pathogenic *Escherichia coli* for predicting the growth of pathogenic *Escherichia coli*. J Appl Microbiol. 1998;85:357–364.

252. Salvadó Z, Arroyo-López FN, Guillamón JM, Salazar G, Querol A, Barrio E. Temperature adaptation markedly determines evolution within the *Saccharomyces* genus. Appl Environ Microbiol. 2011;77(7):2292–2302.

253. Smith RV, Evans MCW. Nitrogenase activity in cell-free extracts of the blue-green alga, *Anabaena cylindrica*. J Bacteriol. 1971;105(3):913–917.

254. Poli A, Romano I, Caliendo G, Nicolaus G, Orlando P, de Falco A, et al. *Geobacillus toebii* subsp. *decanicus* subsp. nov., a hydrocarbon-degrading, heavy metal resistant bacterium from hot compost. J Gen Appl Microbiol. 2006;52(4):223–234.

255. Bakermans C, Nealson KH. Relationship of critical temperature to macromolecular synthesis and growth yield in *Psychrobacter cryopegella*. J Bacteriol. 2004;186(8):2340–2345.

256. Isaksen MF, Jorgensen BB. Adaptation of psychrophilic and psychrotrophic sulfate-reducing bacteria to permanently cold marine environments. Appl Environ Microbiol. 1996;62:408–414.

257. De Vis RMJ, Fuentes LE, van Lenteren JC. Life history of *Amitus fuscipennis* (Hym., Platygastridae) as parasitoid of the greenhouse white fly *Trialeurodes vaporariorum* (Hom., Aleyrodidae) on tomato as function of temperature. J Appl Entomol. 2002;126(1):24–33.

258. Force DC, Messenger PS. Fecundity, reproductive rates, and innate capacity for increase of three parasites of *Therioaphis maculata* (Buckton). Ecology. 1964;p. 706–715.

259. Kim JJ, Alkawally M, Brady AL, Rijpstra WIC, Damsté JSS, Dunfield PF. *Chryseolinea serpens* gen. nov., sp. nov., a member of the phylum Bacteroidetes isolated from soil. Int J Syst Evol Microbiol. 2013;63(Pt 2):654–660.

260. Nazina TN, Lebedeva EV, Poltaraus AB, Tourova TP, Grigoryan AA, Sokolova DS, et al. *Geobacillus gargensis* sp. nov., a novel thermophile from a hot spring, and the reclassification of *Bacillus vulcani* as *Geobacillus vulcani* comb. nov. Int J Syst Evol Microbiol. 2004;54(6):2019–2024.

261. Komazaki S. Effects of constant temperatures on population growth of three aphid species, *Toxoptera citricidus* (Kirkaldy), *Aphis citricola* van der Goot and *Aphis gossypii* Glover (Homoptera: Aphididae) on citrus. Appl Entomol Zool. 1982;17(1):75–81.

262. Xia JY, van der Werf W, Rabbinge R. Influence of temperature on bionomics of cotton aphid, *Aphis gossypii*, on cotton. Entomol Exp Appl. 1999;90(1):25–35.

263. Giga DP, Smith RH. Comparative life history studies of four *Callosobruchus* species infesting cowpeas with special reference to *Callosobruchus rhodesianus*(Pic)(Coleoptera: Bruchidae). J Stored Prod Res. 1983;19(4):189–198.

264. Wang KH, Tsai JH. Temperature effect on development and reproduction of silverleaf whitefly (Homoptera: Aleyrodidae). Ann Entomol Soc Am. 1996;89(3):375–384.

265. DeLoach CJ. Rate of increase of populations of cabbage, green peach, and turnip aphids at constant temperatures. Ann Entomol Soc Am. 1974;67(3):332–340.

266. Vargas RI, Walsh WA, Kanehisa D, Jang EB, Armstrong JW. Demography of four Hawaiian fruit flies (Diptera: Tephritidae) reared at five constant temperatures. Ann Entomol Soc Am. 1997;90(2):162–168.

267. Sorokin C. Kinetic Studies of Temperature Effects on the Cellular Level. Biochim Biophys Acta. 1960;38(2):197–204.

268. Siddiqui WH, Barlow CA. Population growth of *Drosophila melanogaster* (Diptera: Drosophilidae) at constant and alternating temperatures. Ann Entomol Soc Am. 1972;65(5):993–1001.

269. Mbapila JC, Overholt WA. Comparative development, longevity and population growth of exotic and native parasitoids of lepidopteran cereal stemborers in Kenya. Bull Entomol Res. 2001;91(05):347–353.

270. Smith LB. The intrinsic rate of natural increase of *Cryptolestes ferrugineus* (Stephens) (Coleoptera, Cucujidae). J Stored Prod Res. 1965;1(1):35–49.

271. Hosking JR. The effect of temperature on the population growth potential of *Dactylopius austrinus* De Lotto (Homoptera: Dactylopiidae), on *Opuntia aurantiaca* Lindley. Aust J Entomol. 1984;23(2):133–139.

272. Perdikis DC, Lykouressis DP. Life table and biological characteristics of *Macrolophus pygmaeus* when feeding on *Myzus persicae* and *Trialeurodes vaporariorum*. Entomol Exp Appl. 2002;102(3):261–272.

273. Ren SX, Stansly PA, Liu TX. Life history of the whitefly predator *Nephaspis oculatus* (Coleoptera: Coccinellidae) at six constant temperatures. Biol Control. 2002;23(3):262–268.

274. Yang P, Carey JR, Dowell RV. Temperature influences on the development and demography of *Bactrocera dorsalis* (Diptera: Tephritidae) in China. Environ Entomol. 1994;23(4):971–974.

275. Beckett SJ, Evans DE. The demography of *Oryzaephilus surinamensis* (L.) (Coleoptera: Silvanidae) on kibbled wheat. J Stored Prod Res. 1994;30(2):121–137.

276. Beckett SJ, Longstaff BC, Evans DE. A comparison of the demography of four major stored grain coleopteran pest species and its implications for pest management. In: Proceedings of the 6th International Working Conference on Stored-product Protection, Volume 1, Canberra; 1994. p. 491–497.

277. Allsopp PG. Development, longevity and fecundity of the false wireworms *Pterohelaeus darlingensis* and *P. alternatus* (Coleoptera : Tenebrionidae) I. Effect of constant temperature. Aust J Zool. 1981;29(4):605–619.

278. Birch LC. Experimental background to the study of the distribution and abundance of insects: I. The influence of temperature, moisture and food on the innate capacity for increase of three grain beetles. Ecology. 1953;34(4):698–711.

279. Rae DJ, De'ath G. Influence of constant temperature on development, survival and fecundity of sugarcane mealybug, *Saccharicoccus sacchari* (Cockerell) (Hemiptera, Pseudococcidae). Aust J Zool. 1991;39(1):105–122.

280. Hoddle MS. Developmental and reproductive biology of *Scirtothrips perseae* (Thysanoptera: Thripidae): a new avocado pest in California. Bull Entomol Res. 2002;92(04):279–285.

281. Turak E, Talent R, Sunnucks P, Hales DF. Different responses to temperature in three closely-related sympatric cereal aphids. Entomol Exp Appl. 1998;86(1):49–58.

282. Murai T. Effect of temperature on development and reproduction of the onion thrips, *Thrips tabaci* Lindeman (Thysanoptera: Thripidae), on pollen and honey solution. Appl Entomol Zool. 2000;35(4):499–504.

283. Pratissoli D, Parra JRP. Fertility life table of *Trichogramma pretiosum* (Hym., Trichogrammatidae) in eggs of *Tuta absoluta* and *Phthorimaea operculella* (Lep., Gelechiidae) at different temperatures. J Appl Entomol. 2000;124(9–10):339–342.

284. Tsai JH, Liu YH. Effect of temperature on development, survivorship, and reproduction of rice root aphid (Homoptera: Aphididae). Environ Entomol. 1998;27(3):662–666.

285. Lysyk TJ. Relationships between temperature and life history parameters of *Muscidifurax zaraptor* (Hymenoptera: Pteromalidae). Environ Entomol. 2001;30(1):147–156.

286. Lysyk TJ. Relationships between temperature and life history parameters of *Muscidifurax raptorellus* (Hymenoptera: Pteromalidae). Environ Entomol. 2001;30(5):982–992.

287. van Huis A, Arendse PW, Schilthuizen M, Wiegers PP, Heering H, Hulshof M, et al. *Uscana lariophaga*, egg parasitoid of bruchid beetle storage pests of cowpea in West Africa: the effect of temperature and humidity. Entomol Exp Appl. 1994;70:41–53.

288. Asante SK, Danthanarayana W, Heatwole H. Bionomics and population growth statistics of apterous virginoparae of woolly apple aphid, *Eriosoma lanigerum*, at constant temperatures. Entomol Exp Appl. 1991;60:261–270.

289. Rodriguez-del Bosque LA, Smith JW, Browning HW. Development and life-fertility tables for *Diatraea lineolata* (Lepidoptera: Pyralidae) at constant temperatures. Ann Entomol Soc Am. 1989;82(4):450–459.

290. Shu-Sheng L, Hughes RD. The influence of temperature and photoperiod on the development, survival and reproduction of the sowthistle aphid, *Hyperomyzus lactucae*. Entomol Exp Appl. 1987;43(1):31–38.

291. Howe RW. The effects of temperature and humidity on the oviposition rate of *Tribolium castaneum* (Hbst.) (Coleoptera:Tenebrionidae). Bull Entomol Res. 1962;53(2):301–310.

292. Longstaff BC, Evans DE. The demography of the rice weevil, *Sitophilus oryzae* (L.)(Coleoptera: Curculionidae), submodels of age-specific survivorship and fecundity. Bull Entomol Res. 1983;73(02):333–334.

293. Naranjo SE. Life history of *Trichogrammatoidea bactrae* (Hymenoptera: Trichogrammatidae), an egg parasitoid of pink bollworm (Lepidoptera: Gelechiidae), with emphasis on performance at high temperatures. Environ Entomol. 1993;22(5):1051–1059.

294. Mesbah NM, Wiegel J. *Natronovirga wadinatrunensis* gen. nov., sp. nov. and *Natranaerobius trueperi* sp. nov., halophilic, alkalithermophilic micro-organisms from soda lakes of the Wadi An Natrun, Egypt. Int J Syst Evol Microbiol. 2009;59(8):2042–2048.

295. Ogg CD, Patel BKC. *Thermotalea metallivorans* gen. nov., sp. nov., a thermophilic, anaerobic bacterium from the Great Artesian Basin of Australia aquifer. Int J Syst Evol Microbiol. 2009;59(5):964–971.

296. Sekiguchi Y, Yamada T, Hanada S, Ohashi A, Harada H, Kamagata Y. *Anaerolinea thermophila* gen. nov., sp. nov. and *Caldilinea aerophila* gen. nov., sp. nov., novel filamentous thermophiles that represent a previously uncultured lineage of the domain Bacteria at the subphylum level. Int J Syst Evol Microbiol. 2003;53(6):1843–1851.

297. Rohitha BH, Penman DR. Effect of temperature on the biology of bluegreen lucerne aphid, *Acyrthosiphon kondoi*. New Zeal J Zool. 1983;10(3):299–308.

298. Abdeljabbar H, Cayol JL, Hania WB, Boudabous A, Sadfi N, Fardeau ML. *Halanaerobium sehlinense* sp. nov., an extremely halophilic, fermentative, strictly anaerobic bacterium from sediments of the hypersaline lake Sehline Sebkha. Int J Syst Evol Microbiol. 2013;63(Pt 6):2069–2074.

299. Urios L, Agogué H, Lesongeur F, Stackebrandt E, Lebaron P. *Balneola vulgaris* gen. nov., sp. nov., a member of the phylum Bacteroidetes from the north-western Mediterranean Sea. Int J Syst Evol Microbiol. 2006;56(8):1883–1887.

300. Bernalier A, Willems A, Leclerc M, Rochet V, Collins MD. *Ruminococcus hydrogenotrophicus* sp. nov., a new H2/CO2-utilizing acetogenic bacterium isolated from human feces. Arch Microbiol. 1996;166(3):176–183.

301. Lund MB, Schätzle S, Schramm A, Kjeldsen KU. *Verminephrobacter aporrectodeae* sp. nov. subsp. *tuberculatae* and subsp. *caliginosae*, the specific nephridial symbionts of the earthworms *Aporrectodea tuberculata* and *A. caliginosa*. Antonie van Leeuwenhoek. 2012;101(3):507–514.

302. Pinel N, Davidson SK, Stahl DA. *Verminephrobacter eiseniae* gen. nov., sp. nov., a nephridial symbiont of the earthworm *Eisenia foetida* (Savigny). Int J Syst Evol Microbiol. 2008;58(9):2147–2157.

303. Orlygsson J, Baldursson SRB. Phylogenetic and physiological studies of four hydrogen-producing thermoanaerobes from Icelandic geothermal areas. Icel Agric Sci. 2007;20:93–106.

304. Vésteinsdóttir H, Reynisdóttir DB, Örlygsson J. *Hydrogenophilus islandicus* sp. nov., a thermophilic hydrogen-oxidizing bacterium isolated from an Icelandic hot spring. Int J Syst Evol Microbiol. 2011;61(2):290–294.

305. Mäkinen AE, Kaksonen AH, Puhakka JA. *Thermovorax subterraneus*, gen. nov., sp. nov., a thermophilic hydrogen-producing bacterium isolated from geothermally active underground mine. Extremophiles. 2009;13(3):505–510.

306. Aguiar P, Beveridge TJ, Reysenbach AL. *Sulfurihydrogenibium azorense*, sp. nov., a thermophilic hydrogen-oxidizing microaerophile from terrestrial hot springs in the Azores. Int J Syst Evol Microbiol. 2004;54(1):33–39.

307. Alain K, Postec A, Grinsard E, Lesongeur F, Prieur D, Godfroy A. *Thermodesulfatator atlanticus* sp. nov., a thermophilic, chemolithoautotrophic, sulfate-reducing bacterium isolated from a Mid-Atlantic Ridge hydrothermal vent. Int J Syst Evol Microbiol. 2010;60(1):33–38.

308. Mori K, Harayama S. *Methanobacterium petrolearium* sp. nov. and *Methanobacterium ferruginis* sp. nov., mesophilic methanogens isolated from salty environments. Int J Syst Evol Microbiol. 2011;61(1):138–143.

309. Moussard H, L'haridon S, Tindall B, Banta A, Schumann P, Stackebrandt E, et al. *Thermodesulfatator indicus* gen. nov., sp. nov., a novel thermophilic chemolithoautotrophic sulfate-reducing bacterium isolated from the Central Indian Ridge. Int J Syst Evol Microbiol. 2004;54(1):227–233.

310. Kuwabara T, Minaba M, Ogi N, Kamekura M. *Thermococcus celericrescens* sp. nov., a fast-growing and cell-fusing hyperthermophilic archaeon from a deep-sea hydrothermal vent. Int J Syst Evol Microbiol. 2007;57(3):437–443.

311. Nepomnyashchaya YN, Slobodkina GB, Baslerov RV, Chernyh NA, Bonch-Osmolovskaya EA, Netrusov AI, et al. *Moorella humiferrea* sp. nov., a thermophilic, anaerobic bacterium capable of growth via electron shuttling between humic acid and Fe (III). Int J Syst Evol Microbiol. 2012;62(Pt 3):613–617.

312. Nunoura T, Oida H, Miyazaki M, Suzuki Y. *Thermosulfidibacter takaii* gen. nov., sp. nov., a thermophilic, hydrogen-oxidizing, sulfur-reducing chemolithoautotroph isolated from a deep-sea hydrothermal field in the Southern Okinawa Trough. Int J Syst Evol Microbiol. 2008;58(3):659–665.

313. Takai K, Kobayashi H, Nealson KH, Horikoshi K. *Sulfurihydrogenibium subterraneum* gen. nov., sp. nov., from a subsurface hot aquifer. Int J Syst Evol Microbiol. 2003;53(3):823–827.

314. Jolivet E, L'Haridon S, Corre E, Forterre P, Prieur D. *Thermococcus gammatolerans* sp. nov., a hyperthermophilic archaeon from a deep-sea hydrothermal vent that resists ionizing radiation. Int J Syst Evol Microbiol. 2003;53(3):847–851.

315. Nakagawa S, Takai K, Horikoshi K, Sako Y. *Persephonella hydrogeniphila* sp. nov., a novel thermophilic, hydrogen-oxidizing bacterium from a deep-sea hydrothermal vent chimney. Int J Syst Evol Microbiol. 2003;53(3):863–869.

316. Takai K, Nealson KH, Horikoshi K. *Methanotorris formicicus* sp. nov., a novel extremely thermophilic, methane-producing archaeon isolated from a black smoker chimney in the Central Indian Ridge. Int J Syst Evol Microbiol. 2004;54(4):1095–1100.

317. O'Neill AH, Liu Y, Ferrera I, Beveridge TJ, Reysenbach AL. *Sulfurihydrogenibium rodmanii* sp. nov., a sulfur-oxidizing chemolithoautotroph from the Uzon Caldera, Kamchatka Peninsula, Russia, and emended description of the genus *Sulfurihydrogenibium*. Int J Syst Evol Microbiol. 2008;58(5):1147–1152.

318. Flores GE, Liu Y, Ferrera I, Beveridge TJ, Reysenbach AL. *Sulfurihydrogenibium kristjanssonii* sp. nov., a hydrogen-and sulfur-oxidizing thermophile isolated from a terrestrial Icelandic hot spring. Int J Syst Evol Microbiol. 2008;58(5):1153–1158.

319. Zhang H, Sekiguchi Y, Hanada S, Hugenholtz P, Kim H, Kamagata Y, et al. *Gemmatimonas aurantiaca* gen. nov., sp. nov., a Gram-negative, aerobic, polyphosphate-accumulating micro-organism, the first cultured representative of the new bacterial phylum Gemmatimonadetes phyl. nov. Int J Syst Evol Microbiol. 2003;53(4):1155–1163.

320. Podosokorskaya OA, Kublanov IV, Reysenbach AL, Kolganova TV, Bonch-Osmolovskaya EA. *Thermosipho affectus* sp. nov., a thermophilic, anaerobic, cellulolytic bacterium isolated from a Mid-Atlantic Ridge hydrothermal vent. Int J Syst Evol Microbiol. 2011;61(5):1160–1164.

321. Pérez-Rodríguez I, Ricci J, Voordeckers JW, Starovoytov V, Vetriani C. *Nautilia nitratireducens* sp. nov., a thermophilic, anaerobic, chemosynthetic, nitrate-ammonifying bacterium isolated from a deep-sea hydrothermal vent. Int J Syst Evol Microbiol. 2010;60(5):1182–1186.

322. Manaia CM, Nogales B, Nunes OC. *Tepidiphilus margaritifer* gen. nov., sp. nov., isolated from a thermophilic aerobic digester. Int J Syst Evol Microbiol. 2003;53(5):1405–1410.

323. Lee YJ, Dashti M, Prange A, Rainey FA, Rohde M, Whitman WB, et al. *Thermoanaerobacter sulfurigignens* sp. nov., an anaerobic thermophilic bacterium that reduces 1 M thiosulfate to elemental sulfur and tolerates 90 mM sulfite. Int J Syst Evol Microbiol. 2007;57(7):1429–1434.

324. Cadillo-Quiroz H, Bräuer SL, Goodson N, Yavitt JB, Zinder SH. *Methanobacterium paludis* sp. nov. and a novel strain of *Methanobacterium lacus* isolated from northern peatlands. Int J Syst Evol Microbiol. 2014;64(Pt 5):1473–1480.

325. Smith JL, Campbell BJ, Hanson TE, Zhang CL, Cary SC. *Nautilia profundicola* sp. nov., a thermophilic, sulfur-reducing epsilonproteobacterium from deep-sea hydrothermal vents. Int J Syst Evol Microbiol. 2008;58(7):1598–1602.

326. Pikuta EV, Marsic D, Itoh T, Bej AK, Tang J, Whitman WB, et al. *Thermococcus thioreducens* sp. nov., a novel hyperthermophilic, obligately sulfur-reducing archaeon from a deep-sea hydrothermal vent. Int J Syst Evol Microbiol. 2007;57(7):1612–1618.

327. Kuwabara T, Kawasaki A, Uda I, Sugai A. *Thermosipho globiformans* sp. nov., an anaerobic thermophilic bacterium that transforms into multicellular spheroids with a defect in peptidoglycan formation. Int J Syst Evol Microbiol. 2011;61(7):1622–1627.

328. Borrel G, Joblin K, Guedon A, Colombet J, Tardy V, Lehours AC, et al. *Methanobacterium lacus* sp. nov., isolated from the profundal sediment of a freshwater meromictic lake. Int J Syst Evol Microbiol. 2012;62(Pt 7):1625–1629.

329. Gorlas A, Croce O, Oberto J, Gauliard E, Forterre P, Marguet E. *Thermococcus nautili* sp. nov., a hyperthermophilic archaeon isolated from a hydrothermal deep-sea vent. Int J Syst Evol Microbiol. 2014;64(Pt 5):1802–1810.

330. Urios L, Cueff-Gauchard V, Pignet P, Postec A, Fardeau ML, Ollivier B, et al. *Thermosipho atlanticus* sp. nov., a novel member of the Thermotogales isolated from a Mid-Atlantic Ridge hydrothermal vent. Int J Syst Evol Microbiol. 2004;54(6):1953–1957.

331. Zhang K, Dong X. *Selenomonas bovis* sp. nov., isolated from yak rumen contents. Int J Syst Evol Microbiol. 2009;59(8):2080–2083.

332. Salinas MB, Fardeau ML, Thomas P, Cayol JL, Patel BK, Ollivier B. *Mahella australiensis* gen. nov., sp. nov., a moderately thermophilic anaerobic bacterium isolated from an Australian oil well. Int J Syst Evol Microbiol. 2004;54(6):2169–2173.

333. Onyenwoke RU, Kevbrin VV, Lysenko AM, Wiegel J. *Thermoanaerobacter pseudethanolicus* sp. nov., a thermophilic heterotrophic anaerobe from Yellowstone National Park. Int J Syst Evol Microbiol. 2007;57(10):2191–2193.

334. Mori K, Suzuki Ki, Urabe T, Sugihara M, Tanaka K, Hamada M, et al. *Thioprofundum hispidum* sp. nov., an obligately chemolithoautotrophic sulfur-oxidizing gammaproteobacterium isolated from the hydrothermal field on Suiyo Seamount, and proposal of Thioalkalispiraceae fam. nov. in the order Chromatiales. Int J Syst Evol Microbiol. 2011;61(10):2412–2418.

335. Sekiguchi Y, Muramatsu M, Imachi H, Narihiro T, Ohashi A, Harada H, et al. *Thermodesulfovibrio aggregans* sp. nov. and *Thermodesulfovibrio thiophilus* sp. nov., anaerobic, thermophilic, sulfate-reducing bacteria isolated from thermophilic methanogenic sludge, and emended description of the genus *Thermodesulfovibrio*. Int J Syst Evol Microbiol. 2008;58(11):2541–2548.

336. Slobodkin AI, Reysenbach AL, Slobodkina GB, Baslerov RV, Kostrikina NA, Wagner ID, et al. *Thermosulfurimonas dismutans* gen. nov., sp. nov., an extremely thermophilic sulfur-disproportionating bacterium from a deep-sea hydrothermal vent. Int J Syst Evol Microbiol. 2012;62(Pt 11):2565–2571.

337. Hirayama H, Suzuki Y, Abe M, Miyazaki M, Makita H, Inagaki F, et al. *Methylothermus subterraneus* sp. nov., a moderately thermophilic methanotroph isolated from a terrestrial subsurface hot aquifer. Int J Syst Evol Microbiol. 2011;61(11):2646–2653.

338. Niu L, Song L, Liu X, Dong X. *Tepidimicrobium xylanilyticum* sp. nov., an anaerobic xylanolytic bacterium, and emended description of the genus *Tepidimicrobium*. Int J Syst Evol Microbiol. 2009;59(11):2698–2701.

339. Mori K, Yamaguchi K, Sakiyama Y, Urabe T, Suzuki Ki. *Caldisericum exile* gen. nov., sp. nov., an anaerobic, thermophilic, filamentous bacterium of a novel bacterial phylum, Caldiserica phyl. nov., originally called the candidate phylum OP5, and description of Caldisericaceae fam. nov., Caldisericales ord. nov. and Caldisericia classis nov. Int J Syst Evol Microbiol. 2009;59(11):2894–2898.

340. Gorlas A, Alain K, Bienvenu N, Geslin C. *Thermococcus prieurii* sp. nov., a hyperthermophilic archaeon isolated from a deep-sea hydrothermal vent. Int J Syst Evol Microbiol. 2013;63(Pt 8):2920–2926.

341. Podosokorskaya OA, Bonch-Osmolovskaya EA, Godfroy A, Gavrilov SN, Beskorovaynaya DA, Sokolova TG, et al. *Thermosipho activus* sp. nov., a thermophilic, anaerobic, hydrolytic bacterium isolated from a deep-sea sample. Int J Syst Evol Microbiol. 2014;64(Pt 9):3307–3313.

342. Alves JI, van Gelder AH, Alves MM, Sousa DZ, Plugge CM. *Moorella stamsii* sp. nov., a new anaerobic thermophilic hydrogenogenic carboxydotroph isolated from digester sludge. Int J Syst Evol Microbiol. 2013;63(Pt 11):4072–4076.

343. Touzel JP, O'Donohue M, Debeire P, Samain E, Breton C. *Thermobacillus xylanilyticus* gen. nov., sp. nov., a new aerobic thermophilic xylan-degrading bacterium isolated from farm soil. Int J Syst Evol Microbiol. 2000;50(1):315–320.

344. Moreira C, Rainey FA, Nobre MF, da Silva MT, da Costa MS. *Tepidimonas ignava* gen. nov., sp. nov., a new chemolithoheterotrophic and slightly thermophilic member of the $\beta$-Proteobacteria. Int J Syst Evol Microbiol. 2000;50(2):735–742.

345. Arab H, Völker H, Thomm M. *Thermococcus aegaeicus* sp. nov. and *Staphylothermus hellenicus* sp. nov., two novel hyperthermophilic archaea isolated from geothermally heated vents off Palaeochori Bay, Milos, Greece. Int J Syst Evol Microbiol. 2000;50(6):2101–2108.

346. Cann I, Stroot PG, Mackie KR, White BA, Mackie RI. Characterization of two novel saccharolytic, anaerobic thermophiles, *Thermoanaerobacterium polysaccharolyticum* sp. nov. and *Thermoanaerobacterium zeae* sp. nov., and emendation of the genus *Thermoanaerobacterium*. Int J Syst Evol Microbiol. 2001;51(2):293–302.

347. Cuzin N, Ouattara AS, Labat M, Garcia JL. *Methanobacterium congolense* sp. nov., from a methanogenic fermentation of cassava peel. Int J Syst Evol Microbiol. 2001;51(2):489–493.

348. Spanevello MD, Yamamoto H, Patel BKC. *Thermaerobacter subterraneus* sp. nov., a novel aerobic bacterium from the Great Artesian Basin of Australia, and emendation of the genus *Thermaerobacter*. Int J Syst Evol Microbiol. 2002;52(3):795–800.

349. L'Haridon S, Miroshnichenko ML, Hippe H, Fardeau ML, Bonch-Osmolovskaya EA, Stackebrandt E, et al. *Petrotoga olearia* sp. nov. and *Petrotoga sibirica* sp. nov., two thermophilic bacteria isolated from a continental petroleum reservoir in Western Siberia. Int J Syst Evol Microbiol. 2002;52(5):1715–1722.

350. Huber H, Diller S, Horn C, Rachel R. *Thermovibrio ruber* gen. nov., sp. nov., an extremely thermophilic, chemolithoautotrophic, nitrate-reducing bacterium that forms a deep branch within the phylum Aquificae. Int J Syst Evol Microbiol. 2002;52(5):1859–1865.

351. Johnson DB, Joulian C, d'Hugues P, Hallberg KB. *Sulfobacillus benefaciens* sp. nov., an acidophilic facultative anaerobic Firmicute isolated from mineral bioleaching operations. Extremophiles. 2008;12(6):789–798.

352. Shooner F, Bousquet J, Tyagi RD. Isolation, phenotypic characterization, and phylogenetic position of a novel, facultatively autotrophic, moderately thermophilic bacterium, *Thiobacillus thermosulfatus* sp. nov. Int J Syst Bacteriol. 1996;46(2):409–415.

353. Lingens F, Blecher R, Blecher H, Blobel F, Eberspächer J, Fröhner C, et al. *Phenylobacterium immobile* gen. nov., sp. nov., a gram-negative bacterium that degrades the herbicide chloridazon. Int J Syst Bacteriol. 1985;35(1):26–39.

354. Takai K, Hirayama H, Nakagawa T, Suzuki Y, Nealson KH, Horikoshi K. *Lebetimonas acidiphila* gen. nov., sp. nov., a novel thermophilic, acidophilic, hydrogen-oxidizing chemolithoautotroph within the 'Epsilonproteobacteria', isolated from a deep-sea hydrothermal fumarole in the Mariana Arc. Int J Syst Evol Microbiol. 2005;55(1):183–189.

355. Goto K, Mochida K, Asahara M, Suzuki M, Kasai H, Yokota A. *Alicyclobacillus pomorum* sp. nov., a novel thermo-acidophilic, endospore-forming bacterium that does not possess $\omega$-alicyclic fatty acids, and emended description of the genus *Alicyclobacillus*. Int J Syst Evol Microbiol. 2003;53(5):1537–1544.

356. Urios L, Cueff V, Pignet P, Barbier G. *Tepidibacter formicigenes* sp. nov., a novel spore-forming bacterium isolated from a Mid-Atlantic Ridge hydrothermal vent. Int J Syst Evol Microbiol. 2004;54(2):439–443.

357. Katayama T, Yoshioka H, Mochimaru H, Meng XY, Muramoto Y, Usami J, et al. *Methanohalophilus levihalophilus* sp. nov., a slightly halophilic, methylotrophic methanogen isolated from natural gas-bearing deep aquifers, and emended description of the genus *Methanohalophilus*. Int J Syst Evol Microbiol. 2014;64(Pt 6):2089–2093.

358. Yamada T, Sekiguchi Y, Hanada S, Imachi H, Ohashi A, Harada H, et al. *Anaerolinea thermolimosa* sp. nov., *Levilinea saccharolytica* gen. nov., sp. nov. and *Leptolinea tardivitalis* gen. nov., sp. nov., novel filamentous anaerobes, and description of the new classes Anaerolineae classis nov. and Caldilineae classis nov. in the bacterial phylum Chloroflexi. Int J Syst Evol Microbiol. 2006;56(6):1331–1340.

359. Grégoire P, Bohli M, Cayol JL, Joseph M, Guasco S, Dubourg K, et al. *Caldilinea tarbellica* sp. nov., a filamentous, thermophilic, anaerobic bacterium isolated from a deep hot aquifer in the Aquitaine Basin. Int J Syst Evol Microbiol. 2011;61(6):1436–1441.

360. Ravot G, Magot M, Fardeau ML, Patel B, Prensier G, Egan A, et al. *Thermotoga elfii* sp. nov., a novel thermophilic bacterium from an African oil-producing well. Int J Syst Bacteriol. 1995;45(2):308–314.

361. Kale V, Björnsdóttir SH, Friðjónsson ÓH, Pétursdóttir SK, Ómarsdóttir S, Hreggviðsson GÓ. *Litorilinea aerophila* gen. nov., sp. nov., an aerobic member of the class Caldilineae, phylum Chloroflexi, isolated from an intertidal hot spring. Int J Syst Evol Microbiol. 2013;63(Pt 3):1149–1154.

362. Billing E. The effect of temperature on the growth of the fireblight pathogen, *Erwinia amylovora*. J Appl Bacteriol. 1974;37(4):643–648.

363. Cook GM, Rainey FA, Patel BKC, Morgan HW. Characterization of a new obligately anaerobic thermophile, *Thermoanaerobacter wiegelii* sp. nov. Int J Syst Bacteriol. 1996;46(1):123–127.

364. Sako Y, Takai K, Ishida Y, Uchida A, Katayama Y. *Rhodothermus obamensis* sp. nov., a modern lineage of extremely thermophilic marine bacteria. Int J Syst Bacteriol. 1996;46(4):1099–1104.

365. Miroshnichenko ML, Slobodkin AI, Kostrikina NA, L'Haridon S, Nercessian O, Spring S, et al. *Deferribacter abyssi* sp. nov., an anaerobic thermophile from deep-sea hydrothermal vents of the Mid-Atlantic Ridge. Int J Syst Evol Microbiol. 2003;53(5):1637–1641.

366. Hanada S, Kawase Y, Hiraishi A, Takaichi S, Matsuura K, Shimada K, et al. *Porphyrobacter tepidarius* sp. nov., a moderately thermophilic aerobic photosynthetic bacterium isolated from a hot spring. Int J Syst Bacteriol. 1997;47(2):408–413.

367. Kotelnikova S, Macario AJL, Pedersen K. *Methanobacterium subterraneum* sp. nov., a new alkaliphilic, eurythermic and halotolerant methanogen isolated from deep granitic groundwater. Int J Syst Bacteriol. 1998;48(2):357–367.

368. Lien T, Madsen M, Rainey FA, Birkeland NK. *Petrotoga mobilis* sp. nov., from a North Sea oil-production well. Int J Syst Bacteriol. 1998;48(3):1007–1013.

369. Urios L, Agogué H, Intertaglia L, Lesongeur F, Lebaron P. *Melitea salexigens* gen. nov., sp. nov., a gammaproteobacterium from the Mediterranean Sea. Int J Syst Evol Microbiol. 2008;58(11):2479–2483.

370. Brinkhoff T, Muyzer G, Wirsen CO, Kuever J. *Thiomicrospira chilensis* sp. nov., a mesophilic obligately chemolithoautotrophic sulfur-oxidizing bacterium isolated from a *Thioploca* mat. Int J Syst Bacteriol. 1999;49(2):875–879.

371. Kashefi K, Lovley DR. Extending the upper temperature limit for life. Science. 2003;301(5635):934.

372. Takai K, Nealson KH, Horikoshi K. *Hydrogenimonas thermophila* gen. nov., sp. nov., a novel thermophilic, hydrogen-oxidizing chemolithoautotroph within the $\varepsilon$-Proteobacteria, isolated from a black smoker in a Central Indian Ridge hydrothermal field. Int J Syst Evol Microbiol. 2004;54(1):25–32.

373. Serra A, Strehaiano P, Taillandier P. Influence of temperature and pH on *Saccharomyces bayanus* var. *uvarum* growth; impact of a wine yeast interspecific hybridization on these parameters. Int J Food Microbiol. 2005;104:257–265.

374. Chistyakova TA, Minkevich IG, Eroshin VK. Growth of the thermotolerant yeast, *Candida valida*, on ethanol: dependences of maximal growth rate and cell biomass yield on temperature. Eur J Appl Microbiol. 1983;18:225–228.

375. Brandam C, Castro-Martínez C, Délia ML, Ramón-Portugal F, Strehaiano P. Effect of temperature on *Brettanomyces bruxellensis*: metabolic and kinetic aspects. Can J Microbiol. 2008;5(1):11–18.

376. Miller SR, Castenholz RW. The evolution of thermotolerance in hot spring cyanobacteria of the genus *Synechococcus*. J Phycol. 2000;36(s3):48–48.

377. Zeng X, Zhang Z, Li X, Jebbar M, Alain K, Shao Z. *Caloranaerobacter ferrireducens* sp. nov., an anaerobic thermophilic iron (III)-reducing bacterium isolated from a deep-sea hydrothermal sulfide deposits. Int J Syst Evol Microbiol. 2015;p. ijs–0.

378. Miroshnichenko ML, Hippe H, Stackebrandt E, Kostrikina NA, Chernyh NA, Jeanthon C, et al. Isolation and characterization of *Thermococcus sibiricus* sp. nov. from a Western Siberia high-temperature oil reservoir. Extremophiles. 2001;5(2):85–91.

379. Ferrer M, Chernikova TN, Yakimov MM, Golyshin PN, Timmis KN. Chaperonins govern growth of *Escherichia coli* at low temperatures. Nat Biotechnol. 2003;21(11):1266–1267.

380. Urios L, Michotey V, Intertaglia L, Lesongeur F, Lebaron P. *Thalassobaculum salexigens* sp. nov., a new member of the family Rhodospirillaceae from the NW Mediterranean Sea, and emended description of the genus *Thalassobaculum.* Int J Syst Evol Microbiol. 2010;60(1):209–213.

381. Baumgartner M, Yapi A, Gröbner-Ferreira R, Stetter KO. Cultivation and properties of *Echinamoeba thermarum* n. sp., an extremely thermophilic amoeba thriving in hot springs. Extremophiles. 2003;7(4):267–274.

382. Baldock BM, Berger J. The effects of low temperatures on the growth of four fresh-water amoebae (Protozoa: Gymnamoebia). Trans Am Microsc Soc. 1984;p. 233–239.

383. Baldock BM, Baker JH, Sleigh MA. Laboratory growth rates of six species of freshwater Gymnamoebia. Oecologia. 1980;47(2):156–159.

384. Chung AP, Rainey F, Nobre MF, Burghardt J, Da Costa MS. *Meiothermus cerbereus* sp. nov., a new slightly thermophilic species with high levels of 3-hydroxy fatty acids. Int J Syst Bacteriol. 1997;47(4):1225–1230.

385. De Rosa M, Gambacorta A, Bu'Lock JD. Extremely thermophilic acidophilic bacteria convergent with *Sulfolobus acidocaldarius.* J Gen Microbiol. 1975;86(1):156–164.

386. Schleper C, Puehler G, Holz I, Gambacorta A, Janekovic D, Santarius U, et al. *Picrophilus* gen. nov., fam. nov.: a novel aerobic, heterotrophic, thermoacidophilic genus and family comprising archaea capable of growth around pH 0. J Bacteriol. 1995;177(24):7050–7059.

387. Urit T, Li M, Bley T, Löser C. Growth of *Kluyveromyces marxianus* and formation of ethyl acetate depending on temperature. Appl Microbiol Biotechnol. 2013;97(24):10359–10371.

388. Oshima T, Imhori K. Description of *Thermus thermophilus* (Yoshida and Oshima) comb. nov., a nonsporulating thermophilic bacterium from a Japanese thermal spa. Int J Syst Bacteriol. 1974;24(1):102–112.

389. Brock TD, Freeze H. *Thermus aquaticus* gen. n. and sp. n., a nonsporulating extreme thermophile. J Bacteriol. 1969;98(1):289–297.

390. Jones WJ, Leigh JA, Mayer F, Woese CR, Wolfe RS. *Methanococcus jannaschii* sp. nov., an extremely thermophilic methanogen from a submarine hydrothermal vent. Arch Microbiol. 1983;136(4):254–261.

391. Darland G, Brock TD. *Bacillus acidocaldarius* sp. nov., an acidophilic thermophilic spore-forming bacterium. J Gen Microbiol. 1971;67(1):9–15.

392. Imachi H, Sekiguchi Y, Kamagata Y, Hanada S, Ohashi A, Harada H. *Pelotomaculum thermopropionicum* gen. nov., sp. nov., an anaerobic, thermophilic, syntrophic propionate-oxidizing bacterium. Int J Syst Evol Microbiol. 2002;52(5):1729–1735.

393. Hattori S, Kamagata Y, Hanada S, Shoun H. *Thermacetogenium phaeum* gen. nov., sp. nov., a strictly anaerobic, thermophilic, syntrophic acetate-oxidizing bacterium. Int J Syst Evol Microbiol. 2000;50(4):1601–1609.

394. Mateles RI, Baruah JN, Tannenbaum SR. Growth of a thermophilic bacterium on hydrocarbons: a new source of single-cell protein. Science. 1967;157(3794):1322–1323. Bacillus stearothermophilus = Geobacillus stearothermophilus.

395. Lee DW, Koh YS, Kim KJ, Kim BC, Choi HJ, Kim DS, et al. Isolation and characterization of a thermophilic lipase from *Bacillus thermoleovorans* ID-1. FEMS Microbiol Lett. 1999;179(2):393–400.

396. Leigh JA, Mayer F, Wolfe RS. *Acetogenium kivui*, a new thermophilic hydrogen-oxidizing acetogenic bacterium. Arch Microbiol. 1981;129(4):275–280.

397. Larsen L, Nielsen P, Ahring BK. *Thermoanaerobacter mathranii* sp. nov., an ethanol-producing, extremely thermophilic anaerobic bacterium from a hot spring in Iceland. Arch Microbiol. 1997;168(2):114–119.

398. Bing W, Wang H, Zheng B, Zhang F, Zhu G, Feng Y, et al. *Caldicellulosiruptor changbaiensis* sp. nov., a cellulolytic and hydrogen-producing bacterium from a hot spring. Int J Syst Evol Microbiol. 2015;65(Pt 1):293–297.

399. Wahlund TM, Woese CR, Castenholz RW, Madigan MT. A thermophilic green sulfur bacterium from New Zealand hot springs, *Chlorobium tepidum* sp. nov. Arch Microbiol. 1991;156(2):81–90.

400. Goh F, Leuko S, Allen MA, Bowman JP, Kamekura M, Neilan BA, et al. *Halococcus hamelinensis* sp. nov., a novel halophilic archaeon isolated from stromatolites in Shark Bay, Australia. Int J Syst Evol Microbiol. 2006;56(6):1323–1329.

401. Huchet V, Pavan S, Lochardet A, Divanac'h ML, Postollec F, Thuault D. Development and application of a predictive model of *Aspergillus candidus* growth as a tool to improve shelf life of bakery products. Food Microbiol. 2013;36:254–259.

402. Lee JH, Williamson D, Rogers PL. The effect of temperature on the kinetics of ethanol production by *Saccharomyces uvarum.* Biol Lett. 1980;2(4):83–88.

403. Van Uden N. Temperature Profiles of Yeasts. Adv Microb Physiol. 1985;25:195.

404. Lombard F, Labeyrie L, Michel E, Spero HJ, Lea DW. Modelling the temperature dependent growth rates of planktic foraminifera. Mar Micropaleontol. 2009;70(1):1–7.

405. Erauso G, Reysenbach AL, Godfroy A, Meunier JR, Crump B, Partensky F, et al. *Pyrococcus abyssi* sp. nov., a new hyperthermophilic archaeon isolated from a deep-sea hydrothermal vent. Arch Microbiol. 1993;160(5):338–349.

406. Fardeau ML, Magot M, Patel BK, Thomas P, Garcia JL, Ollivier B. *Thermoanaerobacter subterraneus* sp. nov., a novel thermophile isolated from oilfield water. Int J Syst Evol Microbiol. 2000;50(6):2141–2149.

407. Giovannoni SJ, Schabtach E, Castenholz RW. *Isosphaera pallida*, gen. and comb. nov., a gliding, budding eubacterium from hot springs. Arch Microbiol. 1987;147(3):276–284.

408. Bonch-Osmolovskaya EA, Miroshnichenko ML, Kostrikina NA, Chernych NA, Zavarzin GA. *Thermoproteus uzoniensis* sp. nov., a new extremely thermophilic archaebacterium from Kamchatka continental hot springs. Arch Microbiol. 1990;154(6):556–559.

409. Mohagheghi A, Grohmann KMMH, Himmel M, Leighton L, Updegraff DM. Isolation and characterization of *Acidothermus cellulolyticus* gen. nov., sp. nov., a new genus of thermophilic, acidophilic, cellulolytic bacteria. Int J Syst Bacteriol. 1986;36(3):435–443.

410. Kawasumi T, Igarashi Y, Kodama T, Minoda Y. *Hydrogenobacter thermophilus* gen. nov., sp. nov., an extremely thermophilic, aerobic, hydrogen-oxidizing bacterium. Int J Syst Bacteriol. 1984;34(1):5–10.

411. Sekiguchi Y, Kamagata Y, Nakamura K, Ohashi A, Harada H. *Syntrophothermus lipocalidus* gen. nov., sp. nov., a novel thermophilic, syntrophic, fatty-acid-oxidizing anaerobe which utilizes isobutyrate. Int J Syst Evol Microbiol. 2000;50(2):771–779.

412. Xue Y, Xu Y, Liu Y, Ma Y, Zhou P. *Thermoanaerobacter tengcongensis* sp. nov., a novel anaerobic, saccharolytic, thermophilic bacterium isolated from a hot spring in Tengcong, China. Int J Syst Evol Microbiol. 2001;51(4):1335–1341.

413. Götz D, Banta A, Beveridge TJ, Rushdi AI, Simoneit BRT, Reysenbach AL. *Persephonella marina* gen. nov., sp. nov. and Persephonella guaymasensis sp. nov., two novel, thermophilic, hydrogen-oxidizing microaerophiles from deep-sea hydrothermal vents. Int J Syst Evol Microbiol. 2002;52(4):1349–1359.

414. Hallberg KB, Lindström EB. Characterization of *Thiobacillus caldus* sp. nov., a moderately thermophilic acidophile. Microbiology. 1994;140(12):3451–3456. Mixotroph which requires low ph, grows at 45C when at low ph – grows slowly as a result i.e. one reason why things gro slowly.

415. Zeikus JG, Dawson MA, Thompson TE, Ingvorsen K, Hatchikian EC. Microbial ecology of volcanic sulphidogenesis: isolation and characterization of *Thermodesulfobacterium commune* gen. nov. and sp. nov. J Gen Microbiol. 1983;129(4):1159–1169.

416. Abed RM, Garcia-Pichel F, Hernández-Mariné M. Polyphasic characterization of benthic, moderately halophilic, moderately thermophilic cyanobacteria with very thin trichomes and the proposal of *Halomicronema excentricum* gen. nov., sp. nov. Arch Microbiol. 2002;177(5):361–370.

417. Balk M, Weijma J, Stams AJM. *Thermotoga lettingae* sp. nov., a novel thermophilic, methanol-degrading bacterium isolated from a thermophilic anaerobic reactor. Int J Syst Evol Microbiol. 2002;52(4):1361–1368.

418. Schink B, Zeikus J. *Clostridium thermosulfurogenes* sp. nov., a new thermophile that produces elemental sulphur from thiosulphate. J Gen Microbiol. 1983;129(4):1149–1158.

419. Stan-Lotter H, Pfaffenhuemer M, Legat A, Busse HJ, Radax C, Gruber C. *Halococcus dombrowskii* sp. nov., an archaeal isolate from a Permian alpine salt deposit. Int J Syst Evol Microbiol. 2002;52(5):1807–1814.

420. Zhu J, Liu X, Dong X. *Methanobacterium movens* sp. nov. and *Methanobacterium flexile* sp. nov., isolated from lake sediment. Int J Syst Evol Microbiol. 2011;61(12):2974–2978.

421. Huber R, Eder W, Heldwein S, Wanner G, Huber H, Rachel R, et al. *Thermocrinis ruber* gen. nov., sp. nov., a pink-filament-forming hyperthermophilic bacterium isolated from Yellowstone National Park. Appl Environ Microbiol. 1998;64(10):3576–3583.

422. Amézquita A, Weller CL, Wang L, Thippareddi H, Burson DE. Development of an integrated model for heat transfer and dynamic growth of *Clostridium perfringens* during the cooling of cooked boneless ham. Int J Food Microbiol. 2005;101(2):123–144.

423. Ulitzur S. *Vibrio parahaemolyticus* and *Vibrio alginolyticus*: Short generation-time marine bacteria. Microb Ecol. 1974;1(1):127–135.

424. Le Marc Y, Plowman J, Aldus CF, Munoz-Cuevas M, Baranyi J, Peck MW. Modelling the growth of *Clostridium perfringens* during the cooling of bulk meat. Int J Food Microbiol. 2008;128(1):41–50.

425. Padden AN, Dillon VM, Edmonds J, Collins MD, Alvarez N, John P. An indigo-reducing moderate thermophile from a woad vat, *Clostridium isatidis* sp. nov. Int J Syst Bacteriol. 1999;49(3):1025–1031.

426. Cayol JL, Ducerf S, Patel BK, Garcia JL, Thomas P, Ollivier B. *Thermohalobacter berrensis* gen. nov., sp. nov., a thermophilic, strictly halophilic bacterium from a solar saltern. Int J Syst Evol Microbiol. 2000;50(2):559–564.

427. Park HS, Kim BH, Kim HS, Kim HJ, Kim GT, Kim M, et al. A novel electrochemically active and Fe (III)-reducing bacterium phylogenetically related to *Clostridium butyricum* isolated from a microbial fuel cell. Anaerobe. 2001;7(6):297–306.

428. Kashefi K, Tor JM, Holmes DE, Van Praagh CVG, Reysenbach AL, Lovley DR. *Geoglobus ahangari* gen. nov., sp. nov., a novel hyperthermophilic archaeon capable of oxidizing organic acids and growing autotrophically on hydrogen with Fe (III) serving as the sole electron acceptor. Int J Syst Evol Microbiol. 2002;52(3):719–728.

429. Madigan MT, Jung DO, Woese CR, Achenbach LA. *Rhodoferax antarcticus* sp. nov., a moderately psychrophilic purple nonsulfur bacterium isolated from an Antarctic microbial mat. Arch Microbiol. 2000;173(4):269–277.

430. Franzmann PD, Liu Y, Balkwill DL, Aldrich HC, De Macario EC, Boone DR. *Methanogenium frigidum* sp. nov., a psychrophilic, H2-using methanogen from Ace Lake, Antarctica. Int J Syst Bacteriol. 1997;47(4):1068–1072.

431. Mikucki JA, Liu Y, Delwiche M, Colwell FS, Boone DR. Isolation of a methanogen from deep marine sediments that contain methane hydrates, and description of *Methanoculleus submarinus* sp. nov. Appl Environ Microbiol. 2003;69(6):3311–3316.

432. Seo HJ, Bae SS, Lee JH, Kim SJ. *Photobacterium frigidiphilum* sp. nov., a psychrophilic, lipolytic bacterium isolated from deep-sea sediments of Edison Seamount. Int J Syst Evol Microbiol. 2005;55(4):1661–1666.

433. Völkl P, Huber R, Drobner E, Rachel R, Burggraf S, Trincone A, et al. *Pyrobaculum aerophilum* sp. nov., a novel nitrate-reducing hyperthermophilic archaeum. Appl Environ Microbiol. 1993;59(9):2918–2926.

434. Amo T, Paje MLF, Inagaki A, Ezaki S, Atomi H, Imanaka T. *Pyrobaculum calidifontis* sp. nov., a novel hyperthermophilic archaeon that grows in atmospheric air. Archaea. 2002;1(2):113–121.

435. Dirmeier R, Keller M, Hafenbradl D, Braun FJ, Rachel R, Burggraf S, et al. *Thermococcus acidaminovorans* sp. nov., a new hyperthermophilic alkalophilic archaeon growing on amino acids. Extremophiles. 1998;2(2):109–114.

436. Paper W, Jahn U, Hohn MJ, Kronner M, Näther DJ, Burghardt T, et al. *Ignicoccus hospitalis* sp. nov., the host of *'Nanoarchaeum equitans'*. Int J Syst Evol Microbiol. 2007;57(4):803–808.

437. Rainey FA, Silva J, Nobre MF, Silva MT, da Costa MS. *Porphyrobacter cryptus* sp. nov., a novel slightly thermophilic, aerobic, bacteriochlorophyll a-containing species. Int J Syst Evol Microbiol. 2003;53(1):35–41.

438. Feller G, Narinx E, Arpigny JL, Zekhnini Z, Swings J, Gerday C. Temperature dependence of growth, enzyme secretion and activity of psychrophilic Antarctic bacteria. Appl Microbiol Biotechnol. 1994;41(4):477–479.

439. Shcherbakova V, Rivkina E, Laurinavichuis K, Pecheritsina S, Gilichinsky D. Physiological characteristics of bacteria isolated from water brines within permafrost. Int J Astrobiology. 2004;3(01):37–43.

440. Shcherbakova VA, Chuvilskaya NA, Rivkina EM, Pecheritsyna SA, Laurinavichius KS, Suzina NE, et al. Novel psychrophilic anaerobic spore-forming bacterium from the overcooled water brine in permafrost: description *Clostridium algoriphilum* sp. nov. Extremophiles. 2005;9(3):239–246.

441. Bowman Jp, Gosink Jj, McCammon SA, Lewis TE, Nichols DS, Nichols PD, et al. *Colwellia demingiae* sp. nov., *Colwellia hornerae* sp. nov., *Colwellia rossensis* sp. nov. and *Colwellia psychrotropica* sp. nov.: psychrophilic Antarctic species with the ability to synthesize docosahexaenoic acid (22: $\omega$63). Int J Syst Bacteriol. 1998;48(4):1171–1180.

442. Nogi Y, Kato C, Horikoshi K. *Psychromonas kaikoae* sp. nov., a novel from the deepest piezophilic bacterium cold-seep sediments in the Japan Trench. Int J Syst Evol Microbiol. 2002;52(5):1527–1532.

443. Membré JM, Leporq B, Vialette M, Mettler E, Perrier L, Thuault D, et al. Temperature effect on bacterial growth rate: quantitative microbiology approach including cardinal values and variability estimates to perform growth simulations on/in food. Int J Food Microbiol. 2005;100(1):179–186.

444. Blankenship LC, Craven SE, Leffler RG, Custer C. Growth of *Clostridium perfringens* in cooked chili during cooling. Appl Environ Microbiol. 1988;54(5):1104–1108.

445. Juneja VK, Novak JS, Marks HM, Gombas DE. Growth of *Clostridium perfringens* from spore inocula in cooked cured beef: development of a predictive model. Innov Food Sci Emerg Technol. 2001;2(4):289–301.

446. Juneja VK, Marks H, Thippareddi H. Predictive model for growth of *Clostridium perfringens* during cooling of cooked uncured beef. Food Microbiol. 2008;25(1):42–55.

447. Mackey BM, Kerridge AL. The effect of incubation temperature and inoculum size on growth of salmonellae in minced beef. Int J Food Microbiol. 1988;6(1):57–65.

448. Dickson JS, Siragusa GR, Wray JE. Predicting the growth of *Salmonella typhimurium* on beef by using the temperature function integration technique. Appl Environ Microbiol. 1992;58(11):3482–3487.

449. Leroi F, Fall PA, Pilet MF, Chevalier F, Baron R. Influence of temperature, pH and NaCl concentration on the maximal growth rate of *Brochothrix thermosphacta* and a bioprotective bacteria *Lactococcus piscium* CNCM I-4031. Food Microbiol. 2012;31(2):222–228.

450. Baranyi J, Robinson TP, Kaloti A, Mackey BM. Predicting growth of *Brochothrix thermosphacta* at changing temperature. Int J Food Microbiol. 1995;27(1):61–75.

451. Schaechter M, Maaløe O, Kjeldgaard NO. Dependency on medium and temperature of cell size and chemical composition during balanced growth of *Salmonella typhimurium*. J Gen Microbiol. 1958;19(3):592–606.

452. Pierson BK, Castenholz RW. A phototrophic gliding filamentous bacterium of hot springs, *Chloroflexus aurantiacus*, gen. and sp. nov. Arch Microbiol. 1974;100(1):5–24.

453. Labbe RG, Huang TH. Generation times and modeling of enterotoxin-positive and enterotoxin-negative strains of *Clostridium perfringens* in laboratory media and ground beef. J Food Prot. 1995;58(12):1303–1306.

454. Herendeen SL, Vanbogelen RA, Neidhardt FC. Levels of major proteins of *Escherichia coli* during growth at different temperatures. J Bacteriol. 1979;139(1):185–194.

455. Heitzer A, Kohler H, Reichert P, Hamer G. Utility of phenomenological models for describing temperature dependence of bacterial growth. Appl Environ Microbiol. 1991;57(9):2656–2665.

456. Al-Awadhi N, Egli T, Hamer G. Growth characteristics of a thermotolerant methylotrophic *Bacillus* sp. (NCIB 12522) in batch culture. Appl Microbiol Biotechnol. 1988;29(5):485–493.

457. Park SY, Ha SD. Effect of temperature on the growth kinetics and predictive growth model of *Aeromonas hydrophila* on squid (*Sepioteuthis sepioidea*). Food Sci Biotechnol. 2014;23(1):307–312.

458. Choa BY, Carter CG, Battaglene SC. Effects of temperature regime on growth and development of post-larval striped trumpeter (*Latris lineata*). Aquaculture. 2010;305(1):95–101.

459. Saiki T, Kobayashi Y, Kawagoe K, Beppu T. *Dictyoglomus thermophilum* gen. nov., sp. nov., a chemoorganotrophic, anaerobic, thermophilic bacterium. Int J Syst Bacteriol. 1985;35(3):253–259.

460. Kuhn HJ, Cometta S, Fiechter A. Effects of growth temperature on maximal specific growth rate, yield, maintenance, and death rate in glucose-limited continuous culture of the thermophilic *Bacillus caldotenax*. Eur J Appl Microbiol. 1980;10(4):303–315.

461. Takai K, Suzuki M, Nakagawa S, Miyazaki M, Suzuki Y, Inagaki F, et al. *Sulfurimonas paralvinellae* sp. nov., a novel mesophilic, hydrogen-and sulfur-oxidizing chemolithoautotroph within the Epsilonproteobacteria isolated from a deep-sea hydrothermal vent polychaete nest, reclassification of *Thiomicrospira denitrificans* as *Sulfurimonas denitrificans* comb. nov. and emended description of the genus *Sulfurimonas*. Int J Syst Evol Microbiol. 2006;56(8):1725–1733.

462. Schönheit P, Moll J, Thauer RK. Growth parameters ($K_s$, $\mu_{\max}$, $Y_s$) of *Methanobacterium thermoautotrophicum*. Arch Microbiol. 1980;127(1):59–65.

463. Belkin S, Jannasch HW. A new extremely thermophilic, sulfur-reducing heterotrophic, marine bacterium. Arch Microbiol. 1985;141(3):181–186.

464. Belkin S, Wirsen CO, Jannasch HW. A new sulfur-reducing, extremely thermophilic eubacterium from a submarine thermal vent. Appl Environ Microbiol. 1986;51(6):1180–1185.

465. Huber R, Stöhr J, Hohenhaus S, Rachel R, Burggraf S, Jannasch HW, et al. *Thermococcus chitonophagus* sp. nov., a novel, chitin-degrading, hyperthermophilic archaeum from a deep-sea hydrothermal vent environment. Arch Microbiol. 1995;164(4):255–264.

466. Amend JP, D'Arcy R, Sheth SN, Zolotova N, Amend AC. *Palaeococcus helgesonii* sp. nov., a facultatively anaerobic, hyperthermophilic archaeon from a geothermal well on Vulcano Island, Italy. Arch Microbiol. 2003;179(6):394–401.

467. González JM, Sheckells D, Viebahn M, Krupatkina D, Borges KM, Robb FT. *Thermococcus waiotapuensis* sp. nov., an extremely thermophilic archaeon isolated from a freshwater hot spring. Arch Microbiol. 1999;172(2):95–101.

468. Duffaud GD, d'Hennezel OB, Peek AS, Reysenbach AL, Kelly RM. Isolation and characterization of *Thermococcus barossii*, sp. nov., a hyperthermophilic archaeon isolated from a hydrothermal vent flange formation. Syst Appl Microbiol. 1998;21(1):40–49.

469. Cambon-Bonavita MA, Lesongeur F, Pignet P, Wery N, Lambert C, Godfroy A, et al. Extremophiles, thermophily section, species description *Thermococcus atlanticus* sp. nov., a hyperthermophilic archaeon isolated from a deep-sea hydrothermal vent in the Mid-Atlantic Ridge. Extremophiles. 2003;7(2):101–109.

470. Hazeleger WC, Wouters JA, Rombouts FM, Abee T. Physiological activity of *Campylobacter jejuni* far below the minimal growth temperature. Appl Environ Microbiol. 1998;64(10):3917–3922.

471. Fiala G, Stetter KO. *Pyrococcus furiosus* sp. nov. represents a novel genus of marine heterotrophic archaebacteria growing optimally at 100 C. Arch Microbiol. 1986;145(1):56–61.

472. Huber R, Kristjansson JK, Stetter KO. *Pyrobaculum* gen. nov., a new genus of neutrophilic, rod-shaped archaebacteria from continental solfataras growing optimally at 100 C. Arch Microbiol. 1987;149(2):95–101.

473. Pikuta E, Lysenko A, Chuvilskaya N, Mendrock U, Hippe H, Suzina N, et al. *Anoxybacillus pushchinensis* gen. nov., sp. nov., a novel anaerobic, alkaliphilic, moderately thermophilic bacterium from manure, and description of *Anoxybacillus flavitherms* comb. nov. Int J Syst Evol Microbiol. 2000;50(6):2109–2117.

474. Marteinsson VT, Birrien JL, Reysenbach AL, Vernet M, Marie D, Gambacorta A, et al. *Thermococcus barophilus* sp. nov., a new barophilic and hyperthermophilic archaeon isolated under high hydrostatic pressure from a deep-sea hydrothermal vent. Int J Syst Bacteriol. 1999;49(2):351–359.

475. Riessen S, Antranikian G. Isolation of *Thermoanaerobacter keratinophilus* sp. nov., a novel thermophilic, anaerobic bacterium with keratinolytic activity. Extremophiles. 2001;5(6):399–408.

476. Mori K, Kim H, Kakegawa T, Hanada S. A novel lineage of sulfate-reducing microorganisms: Thermodesulfobiaceae fam. nov., *Thermodesulfobium narugense*, gen. nov., sp. nov., a new thermophilic isolate from a hot spring. Extremophiles. 2003;7(4):283–290.

477. Haridon S, Miroshnichenko M, Hippe H, Fardeau M, Bonch-Osmolovskaya E, Stackebrandt E, et al. *Thermosipho geolei* sp. nov., a thermophilic bacterium isolated from a continental petroleum reservoir in Western Siberia. Int J Syst Evol Microbiol. 2001;51(4):1327–1334.

478. Takahata Y, Nishijima M, Hoaki T, Maruyama T. *Thermotoga petrophila* sp. nov. and *Thermotoga naphthophila* sp. nov., two hyperthermophilic bacteria from the Kubiki oil reservoir in Niigata, Japan. Int J Syst Evol Microbiol. 2001;51(5):1901–1909.

479. Hafenbradl D, Keller M, Dirmeier R, Rachel R, Roßnagel P, Burggraf S, et al. *Ferroglobus placidus* gen. nov., sp. nov., a novel hyperthermophilic archaeum that oxidizes Fe2+ at neutral pH under anoxic conditions. Arch Microbiol. 1996;166(5):308–314.

480. Sowers KR, Baron SF, Ferry JG. *Methanosarcina acetivorans* sp. nov., an acetotrophic methane-producing bacterium isolated from marine sediments. Appl Environ Microbiol. 1984;47(5):971–978.

481. Takai K, Sugai A, Itoh T, Horikoshi K. *Palaeococcus ferrophilus* gen. nov., sp. nov., a barophilic, hyperthermophilic archaeon from a deep-sea hydrothermal vent chimney. Int J Syst Evol Microbiol. 2000;50(2):489–500.

482. Chen MY, Tsay SS, Chen KY, Shi YC, Lin YT, Lin GH. *Pseudoxanthomonas taiwanensis* sp. nov., a novel thermophilic, N2O-producing species isolated from hot springs. Int J Syst Evol Microbiol. 2002;52(6):2155–2161.

483. Takai K, Horikoshi K. *Thermosipho japonicus* sp. nov., an extremely thermophilic bacterium isolated from a deep-sea hydrothermal vent in Japan. Extremophiles. 2000;4(1):9–17.

484. Takai K, Komatsu T, Horikoshi K. *Hydrogenobacter subterraneus* sp. nov., an extremely thermophilic, heterotrophic bacterium unable to grow on hydrogen gas, from deep subsurface geothermal water. Int J Syst Evol Microbiol. 2001;51(4):1425–1435.

485. Kurosawa N, Itoh YH, Iwai T, Sugai A, Uda I, Kimura N, et al. *Sulfurisphaera ohwakuensis* gen. nov., sp. nov., a novel extremely thermophilic acidophile of the order *Sulfolobales*. Int J Syst Bacteriol. 1998;48(2):451–456.

486. Jeanthon C, Reysenbach AL, L'Haridon S, Gambacorta A, Pace NR, Glénat P, et al. *Thermotoga subterranea* sp. nov., a new thermophilic bacterium isolated from a continental oil reservoir. Arch Microbiol. 1995;164(2):91–97.

487. Antoine E, Cilia V, Meunier J, Guezennec J, Lesongeur F, Barbier G. *Thermosipho melanesiensis* sp. nov., a new thermophilic anaerobic bacterium belonging to the order Thermotogales, isolated from deep-sea hydrothermal vents in the southwestern Pacific Ocean. Int J Syst Bacteriol. 1997;47(4):1118–1123.

488. Keller M, Braun FJ, Dirmeier R, Hafenbradl D, Burggraf S, Rachel R, et al. *Thermococcus alcaliphilus* sp. nov., a new hyperthermophilic archaeum growing on polysulfide at alkaline pH. Arch Microbiol. 1995;164(6):390–395.

489. Godfroy A, Meunier JR, Guezennec J, Lesongeur F, Raguenes G, Rimbault A, et al. *Thermococcus fumicolans* sp. nov., a new hyperthermophilic archaeon isolated from a deep-sea hydrothermal vent in the North Fiji Basin. Int J Syst Bacteriol. 1996;46(4):1113–1119.

490. Zhao Y, Boone DR, Mah RA, Boone JE, Xun L. Isolation and characterization of *Methanocorpusculum labreanum* sp. nov. from the LaBrea Tar Pits. Int J Syst Bacteriol. 1989;39(1):10–13.

491. Kadam PC, Ranade DR, Mandelco L, Boone DR. Isolation and characterization of *Methanolobus bombayensis* sp. nov., a methylotrophic methanogen that requires high concentrations of divalent cations. Int J Syst Bacteriol. 1994;44(4):603–607.

492. Zhilina TN, Garnova ES, Tourova TP, Kostrikina NA, Zavarzin GA. *Halonatronum saccharophilum* gen. nov. sp. nov.: a new haloalkaliphilic bacterium of the order Haloanaerobiales from Lake Magadi. Microbiology. 2001;70(1):64–72.

493. Kotsyurbenko OR, Simankova MV, Nozhevnikova AN, Zhilina TN, Bolotina NP, Lysenko AM, et al. New species of psychrophilic acetogens: *Acetobacterium bakii* sp. nov., *A. paludosum* sp. nov., *A. fimetarium* sp. nov. Arch Microbiol. 1995;163(1):29–34.

494. Zhilina TN, Zavarzin GA, Detkova EN, Rainey FA. *Natroniella acetigena* gen. nov. sp. nov., an extremely haloalkaliphilic, homoacetic bacterium: a new member of *Haloanaerobiales.* Curr Microbiol. 1996;32(6):320–326.

495. Mountfort DO, Rainey FA, Burghardt J, Kaspar HF, Stackebrandt E. *Psychromonas antarcticus* gen. nov., sp. nov., a new aerotolerant anaerobic, halophilic psychrophile isolated from pond sediment of the McMurdo Ice Shelf, Antarctica. Arch Microbiol. 1998;169(3):231–238.

496. Simankova MV, Parshina SN, Tourova TP, Kolganova TV, Zehnder AJB, Nozhevnikova AN. *Methanosarcina lacustris* sp. nov., a new psychrotolerant methanogenic archaeon from anoxic lake sediments. Syst Appl Microbiol. 2001;24(3):362–367.

497. Durand P, Reysenbach AL, Prieur D, Pace N. Isolation and characterization of *Thiobacillus hydrothermalis* sp. nov., a mesophilic obligately chemolithotrophic bacterium isolated from a deep-sea hydrothermal vent in Fiji Basin. Arch Microbiol. 1993;159(1):39–44.

498. Zhilina T, Garnova E, Tourova T, Kostrikina N, Zavarzin G. *Amphibacillus fermentum* sp. nov. and *Amphibacillus tropicus* sp. nov., new alkaliphilic, facultatively anaerobic, saccharolytic bacilli from Lake Magadi. Microbiology. 2001;70(6):711–722.

499. Zhang G, Jiang N, Liu X, Dong X. Methanogenesis from methanol at low temperatures by a novel psychrophilic methanogen, '*Methanolobus psychrophilus*' sp. nov., prevalent in Zoige wetland of the Tibetan plateau. Appl Environ Microbiol. 2008;74(19):6114–6120.

500. Rivard CJ, Henson JM, Thomas MV, Smith PH. Isolation and characterization of *Methanomicrobium paynteri* sp. nov., a mesophilic methanogen isolated from marine sediments. Appl Environ Microbiol. 1983;46(2):484–490.

501. Garnova ES, Zhilina TN, Tourova TP, Lysenko AM. *Anoxynatronum sibiricum* gen. nov., sp. nov. alkaliphilic saccharolytic anaerobe from cellulolytic community of Nizhnee Beloe (Transbaikal region). Extremophiles. 2003;7(3):213–220.

502. Chong SC, Liu Y, Cummins M, Valentine DL, Boone DR. *Methanogenium marinum* sp. nov., a H2-using methanogen from Skan Bay, Alaska, and kinetics of H2 utilization. Antonie van Leeuwenhoek. 2002;81(1-4):263–270.

503. Mountfort DO, Rainey FA, Burghardt J, Kaspar HF, Stackebrandt E. *Clostridium vincentii* sp. nov., a new obligately anaerobic, saccharolytic, psychrophilic bacterium isolated from low-salinity pond sediment of the McMurdo Ice Shelf, Antarctica. Arch Microbiol. 1997;167(1):54–60.

504. Kevbrina MV, Okhapkina AA, Akhlynin DS, Kravchenko IK, Nozhevnikova AN, Gal'chenko VF. Growth of mesophilic methanotrophs at low temperatures. Microbiology. 2001;70(4):384–391.

505. Fiala G, Stetter KO, Jannasch HW, Langworthy TA, Madon J. *Staphylothermus marinus* sp. nov. represents a novel genus of extremely thermophilic submarine heterotrophic archaebacteria growing up to $98^o$ C. Syst Appl Microbiol. 1986;8(1):106–113.

506. Willardsen R, Busta F, Allen C, Smith L. Growth and survival of *Clostridium perfringens* during constantly rising temperatures. J Food Sci. 1978;43(2):470–475.

507. Pledger RJ, Baross JA. Characterization of an extremely thermophilic archaebacterium isolated from a black smoker polychaete (*Paralvinella sp.*) at the Juan de Fuca Ridge. Syst Appl Microbiol. 1989;12(3):249–256.

508. Lee YJ, Romanek CS, Wiegel J. *Clostridium aciditolerans* sp. nov., an acid-tolerant spore-forming anaerobic bacterium from constructed wetland sediment. Int J Syst Evol Microbiol. 2007;57(2):311–315.

509. Simankova MV, Kotsyurbenko OR, Stackebrandt E, Kostrikina NA, Lysenko AM, Osipov GA, et al. *Acetobacterium tundrae* sp. nov., a new psychrophilic acetogenic bacterium from tundra soil. Arch Microbiol. 2000;174(6):440–447.

510. Ma K, Liu X, Dong X. *Methanobacterium beijingense* sp. nov., a novel methanogen isolated from anaerobic digesters. Int J Syst Evol Microbiol. 2005;55(1):325–329.

511. Tamaki H, Hanada S, Kamagata Y, Nakamura K, Nomura N, Nakano K, et al. *Flavobacterium limicola* sp. nov., a psychrophilic, organic-polymer-degrading bacterium isolated from freshwater sediments. Int J Syst Evol Microbiol. 2003;53(2):519–526.

512. Yang SH, Lee JH, Ryu JS, Kato C, Kim SJ. *Shewanella donghaensis* sp. nov., a psychrophilic, piezosensitive bacterium producing high levels of polyunsaturated fatty acid, isolated from deep-sea sediments. Int J Syst Evol Microbiol. 2007;57(2):208–212.

513. Huber R, Dyba D, Huber H, Burggraf S, Rachel R. Sulfur-inhibited *Thermosphaera aggregans* sp. nov., a new genus of hyperthermophilic archaea isolated after its prediction from environmentally derived 16S rRNA sequences. Int J Syst Bacteriol. 1998;48(1):31–38.

514. Takai K, Moser DP, Onstott TC, Spoelstra N, Pfiffner SM, Dohnalkova A, et al. *Alkaliphilus transvaalensis* gen. nov., sp. nov., an extremely alkaliphilic bacterium isolated from a deep South African gold mine. Int J Syst Evol Microbiol. 2001;51(4):1245–1256.

515. Tarpgaard IH, Boetius A, Finster K. *Desulfobacter psychrotolerans* sp. nov., a new psychrotolerant sulfate-reducing bacterium and descriptions of its physiological response to temperature changes. Antonie van Leeuwenhoek. 2006;89(1):109–124.

516. Zhilina T, Zavarzin G, Rainey F, Kevbrin V, Kostrikina N, Lysenko A. *Spirochaeta alkalica* sp. nov., *Spirochaeta africana* sp. nov., and *Spirochaeta asiatica* sp. nov., alkaliphilic anaerobes from the continental soda lakes in Central Asia and the East African Rift. Int J Syst Bacteriol. 1996;46(1):305–312.

517. Pikuta EV, Hoover RB, Bej AK, Marsic D, Whitman WB, Krader PE, et al. *Trichococcus patagoniensis* sp. nov., a facultative anaerobe that grows at -5 C, isolated from penguin guano in Chilean Patagonia. Int J Syst Evol Microbiol. 2006;56(9):2055–2062.

518. Chen Z, He C, Hu H. Temperature responses of growth, photosynthesis, fatty acid and nitrate reductase in Antarctic and temperate *Stichococcus*. Extremophiles. 2012;16(1):127–133.

519. Alain K, Marteinsson VT, Miroshnichenko ML, Bonch-Osmolovskaya EA, Prieur D, Birrien JL. *Marinitoga piezophila* sp. nov., a rod-shaped, thermo-piezophilic bacterium isolated under high hydrostatic pressure from a deep-sea hydrothermal vent. Int J Syst Evol Microbiol. 2002;52(4):1331–1339.

520. Jeanthon C, L'Haridon S, Reysenbach AL, Corre E, Vernet M, Messner P, et al. *Methanococcus vulcanius* sp. nov., a novel hyperthermophilic methanogen isolated from East Pacific Rise, and identification of *Methanococcus* sp. DSM 4213Tas *Methanococcus fervens* sp. nov. Int J Syst Bacteriol. 1999;49(2):583–589.

521. Shima S, Suzuki KI. *Hydrogenobacter acidophilus* sp. nov., a thermoacidophilic, aerobic, hydrogen-oxidizing bacterium requiring elemental sulfur for growth. Int J Syst Bacteriol. 1993;43(4):703–708.

522. Itoh T, Suzuki Ki, Sanchez PC, Nakase T. *Caldivirga maquilingensis* gen. nov., sp. nov., a new genus of rod-shaped crenarchaeote isolated from a hot spring in the Philippines. Int J Syst Bacteriol. 1999;49(3):1157–1163.

523. Godfroy A, Lesongeur F, Raguénès G, Quérellou J, Antoine E, Meunier JR, et al. *Thermococcus hydrothermalis* sp. nov., a new hyperthermophilic archaeon isolated from a deep-sea hydrothermal vent. Int J Syst Bacteriol. 1997;47(3):622–626.

524. Raguénès G, Christen R, Guezennec J, Pignet P, Barbier G. *Vibrio diabolicus* sp. nov., a new polysaccharide-secreting organism isolated from a deep-sea hydrothermal vent polychaete annelid, *Alvinella pompejana*. Int J Syst Bacteriol. 1997;47(4):989–995.

525. Sarma SSS, Rao TR. The combined effects of food and temperature on the life history parameters of *Brachionus patulus* Muller (Rotifera). Int Revue ges Hydrobiol. 1991;76(2):225–239.

526. Walz N. Individual culture and experimental population dynamics of *Keratella cochlearis* (Rotatoria). Hydrobiologia. 1983;107(1):35–45.

527. Nagata WD. Long-term acclimation of a parthenogenetic strain of *Brachionus plicatilis* to subnormal temperatures: I. Influence on size, growth, and reproduction. Bull Mar Sci. 1985;37(2):716–725.

528. Bowman JP, McCammon SA, Lewis T, Skerratt JH, Brown JL, Nichols DS, et al. *Psychroflexus torquis* gen. nov., sp. nov. a psychrophilic species from Antarctic sea ice, and reclassification of *Flavobacterium gondwanense* (Dobson et al. 1993) as *Psychroflexus gondwanense* gen. nov., comb. nov. Microbiology. 1998;144(6):1601–1609.

529. Sheridan PP, Loveland-Curtze J, Miteva VI, Brenchley JE. *Rhodoglobus vestalii* gen. nov., sp. nov., a novel psychrophilic organism isolated from an Antarctic Dry Valley lake. Int J Syst Evol Microbiol. 2003;53(4):985–994.

530. Broda D, Saul D, Lawson P, Bell R, Musgrave D. *Clostridium gasigenes* sp. nov., a psychrophile causing spoilage of vacuum-packed meat. Int J Syst Evol Microbiol. 2000;50(1):107–118.

531. Zeng X, Zhang X, Jiang L, Alain K, Jebbar M, Shao Z. *Palaeococcus pacificus* sp. nov., an archaeon from deep-sea hydrothermal sediment. Int J Syst Evol Microbiol. 2013;63(Pt 6):2155–2159.

532. Maestrojuán GM, Boone DR. Characterization of *Methanosarcina barkeri* MST and 227, *Methanosarcina mazei* S-6T, and *Methanosarcina vacuolata* Z-761T. Int J Syst Bacteriol. 1991;41(2):267–274.

533. Kitamura K, Fujita T, Akada S, Tonouchi A. *Methanobacterium kanagiense* sp. nov., a hydrogenotrophic methanogen, isolated from rice-field soil. Int J Syst Evol Microbiol. 2011;61(6):1246–1252.

534. Jeanthon C, L'Haridon S, Reysenbach AL, Vernet M, Messner P, Sleytr U, et al. *Methanococcus infernus* sp. nov., a novel hyperthermophilic lithotrophic methanogen isolated from a deep-sea hydrothermal vent. Int J Syst Bacteriol. 1998;48(3):913–919.

535. Maestrojuán GM, Boone DR, Xun L, Mah RA, Zhang L. Transfer of *Methanogenium bourgense*, *Methanogenium marisnigri*, *Methanogenium olentangyi*, and *Methanogenium thermophilicum* to the Genus *Methanoculleus* gen. nov., emendation of *Methanoculleus marisnigri* and *Methanogenium*, and description of new strains of *Methanoculleus bourgense* and *Methanoculleus marisnigri.* Int J Syst Bacteriol. 1990;40(2):117–122.

536. Chen MY, Wu SH, Lin GH, Lu CP, Lin YT, Chang WC, et al. *Rubrobacter taiwanensis* sp. nov., a novel thermophilic, radiation-resistant species isolated from hot springs. Int J Syst Evol Microbiol. 2004;54(5):1849–1855.

537. Wood AP, Kelly DP. Physiological characteristics of a new thermophilic obligately chemolithotrophic *Thiobacillus* species,*Thiobacillus tepidarius.* Int J Syst Bacteriol. 1985;35(4):434–437.

538. Wagner ID, Zhao W, Zhang CL, Romanek CS, Rohde M, Wiegel J. *Thermoanaerobacter uzonensis* sp. nov., an anaerobic thermophilic bacterium isolated from a hot spring within the Uzon Caldera, Kamchatka, Far East Russia. Int J Syst Evol Microbiol. 2008;58(11):2565–2573.

539. Albuquerque L, Rainey FA, Chung AP, Sunna A, Nobre MF, Grote R, et al. *Alicyclobacillus hesperidum* sp. nov. and a related genomic species from solfataric soils of São Miguel in the Azores. Int J Syst Evol Microbiol. 2000;50(2):451–457.

540. Hirayama H, Takai K, Inagaki F, Nealson KH, Horikoshi K. *Thiobacter subterraneus* gen. nov., sp. nov., an obligately chemolithoautotrophic, thermophilic, sulfur-oxidizing bacterium from a subsurface hot aquifer. Int J Syst Evol Microbiol. 2005;55(1):467–472.

541. Niamsup P, Sujaya IN, Tanaka M, Sone T, Hanada S, Kamagata Y, et al. *Lactobacillus thermotolerans* sp. nov., a novel thermotolerant species isolated from chicken faeces. Int J Syst Evol Microbiol. 2003;53(1):263–268.

542. Nakagawa S, Nakamura S, Inagaki F, Takai K, Shirai N, Sako Y. *Hydrogenivirga caldilitoris* gen. nov., sp. nov., a novel extremely thermophilic, hydrogen-and sulfur-oxidizing bacterium from a coastal hydrothermal field. Int J Syst Evol Microbiol. 2004;54(6):2079–2084.

543. Vetriani C, Speck MD, Ellor SV, Lutz RA, Starovoytov V. *Thermovibrio ammonificans* sp. nov., a thermophilic, chemolithotrophic, nitrate-ammonifying bacterium from deep-sea hydrothermal vents. Int J Syst Evol Microbiol. 2004;54(1):175–181.

544. Takai K, Inoue A, Horikoshi K. *Methanothermococcus okinawensis* sp. nov., a thermophilic, methane-producing archaeon isolated from a Western Pacific deep-sea hydrothermal vent system. Int J Syst Evol Microbiol. 2002;52(4):1089–1095.

545. Jeanthon C, L'Haridon S, Cueff V, Banta A, Reysenbach AL, Prieur D. *Thermodesulfobacterium hydrogeniphilum* sp. nov., a thermophilic, chemolithoautotrophic, sulfate-reducing bacterium isolated from a deep-sea hydrothermal vent at Guaymas Basin, and emendation of the genus *Thermodesulfobacterium.* Int J Syst Evol Microbiol. 2002;52(3):765–772.

546. Itoh T, Suzuki Ki, Nakase T. *Thermocladium modestius* gen. nov., sp. nov., a new genus of rod-shaped, extremely thermophilic crenarchaeote. Int J Syst Bacteriol. 1998;48(3):879–887.

547. Liu Y, Karnauchow TM, Jarrell KF, Balkwill DL, Drake GR, Ringelberg D, et al. Description of two new thermophilic *Desulfotomaculum* spp., *Desulfotomaculum putei* sp. nov., from a deep terrestrial subsurface, and *Desulfotomaculum luciae* sp. nov., from a hot spring. Int J Syst Bacteriol. 1997;47(3):615–621.

548. Boone DR, Liu Y, Zhao ZJ, Balkwill DL, Drake GR, Stevens TO, et al. *Bacillus infernus* sp. nov., an Fe (III)- and Mn (IV)-reducing anaerobe from the deep terrestrial subsurface. Int J Syst Bacteriol. 1995;45(3):441–448.

549. Nakagawa S, Shtaih Z, Banta A, Beveridge TJ, Sako Y, Reysenbach AL. *Sulfurihydrogenibium yellowstonense* sp. nov., an extremely thermophilic, facultatively heterotrophic, sulfur-oxidizing bacterium from Yellowstone National Park, and emended descriptions of the genus *Sulfurihydrogenibium*, *Sulfurihydrogenibium subterraneum* and *Sulfurihydrogenibium azorense.* Int J Syst Evol Microbiol. 2005;55(6):2263–2268.

550. McCammon SA, Innes BH, Bowman JP, Franzmann PD, Dobson SJ, Holloway PE, et al. *Flavobacterium hibernum* sp. nov., a lactose-utilizing bacterium from a freshwater Antarctic lake. Int J Syst Bacteriol. 1998;48(4):1405–1412.

551. Nakagawa S, Inagaki F, Takai K, Horikoshi K, Sako Y. *Thioreductor micantisoli* gen. nov., sp. nov., a novel mesophilic, sulfur-reducing chemolithoautotroph within the $\varepsilon$-*Proteobacteria* isolated from hydrothermal sediments in the Mid-Okinawa Trough. Int J Syst Evol Microbiol. 2005;55(2):599–605.

552. Kendall MM, Liu Y, Sieprawska-Lupa M, Stetter KO, Whitman WB, Boone DR. *Methanococcus aeolicus* sp. nov., a mesophilic, methanogenic archaeon from shallow and deep marine sediments. Int J Syst Evol Microbiol. 2006;56(7):1525–1529.

553. Sako Y, Nomura N, Uchida A, Ishida Y, Morii H, Koga Y, et al. *Aeropyrum pernix* gen. nov., sp. nov., a novel aerobic hyperthermophilic archaeon growing at temperatures up to 100 C. Int J Syst Bacteriol. 1996;46(4):1070–1077.

554. Takai K, Nakagawa S, Sako Y, Horikoshi K. *Balnearium lithotrophicum* gen. nov., sp. nov., a novel thermophilic, strictly anaerobic, hydrogen-oxidizing chemolithoautotroph isolated from a black smoker chimney in the Suiyo Seamount hydrothermal system. Int J Syst Evol Microbiol. 2003;53(6):1947–1954.

555. Nakagawa S, Takai K, Horikoshi K, Sako Y. *Aeropyrum camini* sp. nov., a strictly aerobic, hyperthermophilic archaeon from a deep-sea hydrothermal vent chimney. Int J Syst Evol Microbiol. 2004;54(2):329–335.

556. Dighe AS, Shouche YS, Ranade DR. *Selenomonas lipolytica* sp. nov., an obligately anaerobic bacterium possessing lipolytic activity. Int J Syst Bacteriol. 1998;48(3):783–791.

557. Urios L, Michotey V, Intertaglia L, Lesongeur F, Lebaron P. *Nisaea denitrificans* gen. nov., sp. nov. and *Nisaea nitritireducens* sp. nov., two

novel members of the class *Alphaproteobacteria* from the Mediterranean Sea. Int J Syst Evol Microbiol. 2008;58(10):2336–2341.

558. Korpelainen H. The effects of temperature and photoperiod on life history parameters of *Daphnia magna* (Crustacea: Cladocera). Freshw Biol. 1986;16(5):615–620.

559. Reddy SV, Aspana S, Tushar D, Sasikala C, Ramana CV. *Spirochaeta sphaeroplastigenens* sp. nov., a halo-alkaliphilic, obligately anaerobic spirochaete isolated from soda lake Lonar. Int J Syst Evol Microbiol. 2013;63(Pt 6):2223–2228.

560. Caldwell DE, Caldwell SJ, Laycock JP. *Thermothrix thioparus* gen. et sp. nov. a facultatively anaerobic facultative chemolithotroph living at neutral pH and high temperature. Can J Microbiol. 1976;22(10):1509–1517.

561. Wiegel J, Ljungdahl LG. *Thermoanaerobacter ethanolicus* gen. nov., spec. nov., a new, extreme thermophilic, anaerobic bacterium. Arch Microbiol. 1981;128(4):343–348.

562. Vreeland RH, Litchfield CD, Martin EL, Elliot E. *Halomonas elongata*, a new genus and species of extremely salt-tolerant bacteria. Int J Syst Bacteriol. 1980;30(2):485–495.

563. Butterwick C, Heaney SI, Talling JF. Diversity in the influence of temperature on the growth rates of freshwater algae, and its ecological relevance. Freshw Biol. 2005;50(2):291–300.

564. Sandnes JM, Källqvist T, Wenner D, Gislerød HR. Combined influence of light and temperature on growth rates of *Nannochloropsis oceanica*: linking cellular responses to large-scale biomass production. J Appl Phycol. 2005;17(6):515–525.

565. Dermoun D, Chaumont D, Thebault JM, Dauta A. Modelling of growth of *Porphyridium cruentum* in connection with two interdependent factors: Light and temperature. Bioresour Technol. 1992;42(2):113–117.

566. Xin L, Hong-Ying H, Yu-Ping Z. Growth and lipid accumulation properties of a freshwater microalga *Scenedesmus* sp. under different cultivation temperature. Bioresour Technol. 2011;102(3):3098–3102.

567. Kudo I, Miyamoto M, Noiri Y, Maita Y. Combined effects of temperature and iron on the growth and physiology of the marine diatom *Phaeodactylum tricornutum* (Bacillariophyceae). J Phycol. 2000;36(6):1096–1102.

568. Ferreira AC, Nobre MF, Rainey FA, Silva MT, Wait R, Burghardt J, et al. *Deinococcus geothermalis* sp. nov. and *Deinococcus murrayi* sp. nov., two extremely radiation-resistant and slightly thermophilic species from hot springs. Int J Syst Bacteriol. 1997;47(4):939–947.

569. Bronikowski AM, Bennett AF, Lenski RE. Evolutionary adaptation to temperature. VIII. Effects of temperature on growth rate in natural isolates of *Escherichia coli* and *Salmonella enterica* from different thermal environments. Evolution. 2001;55(1):33–40.

570. Sako Y, Nakagawa S, Takai K, Horikoshi K. *Marinithermus hydrothermalis* gen. nov., sp. nov., a strictly aerobic, thermophilic bacterium from a deep-sea hydrothermal vent chimney. Int J Syst Evol Microbiol. 2003;53(1):59–65.

571. Friedrich AB, Antranikian G. Keratin degradation by *Fervidobacterium pennavorans*, a novel thermophilic anaerobic species of the order Thermotogales. Appl Environ Microbiol. 1996;62(8):2875–2882.

572. Huber R, Langworthy TA, König H, Thomm M, Woese CR, Sleytr UB, et al. *Thermotoga maritima* sp. nov. represents a new genus of unique extremely thermophilic eubacteria growing up to 90 C. Arch Microbiol. 1986;144(4):324–333.

573. Ma K, Liu X, Dong X. *Methanosaeta harundinacea* sp. nov., a novel acetate-scavenging methanogen isolated from a UASB reactor. Int J Syst Evol Microbiol. 2006;56(1):127–131.

574. Davey ME, Wood WA, Key R, Nakamura K, Stahl DA. Isolation of three species of *Geotoga* and *Petrotoga*: two new genera, representing a new lineage in the bacterial line of descent distantly related to the "*Thermotogales*". Syst Appl Microbiol. 1993;16(2):191–200.

575. Kacena MA, Merrell GA, Manfredi B, Smith EE, Klaus DM, Todd P. Bacterial growth in space flight: logistic growth curve parameters for *Escherichia coli* and *Bacillus subtilis*. Appl Microbiol Biotechnol. 1999;51(2):229–234.

576. Huber R, Woese CR, Langworthy TA, Fricke H, Stetter KO. *Thermosipho africanus* gen. nov., Represents a New Genus of Thermophilic Eubacteria within the *"Thermotogales"*. Syst Appl Microbiol. 1989;12(1):32–37.

577. Chellemi DO, Marois JJ. Population dynamics of the plant pathogenic fungus *Uncinula necator*. Can J Bot. 1992;70(5):942–946.

578. Huang CY, Patel BK, Mah RA, Baresi L. *Caldicellulosiruptor owensensis* sp. nov., an anaerobic, extremely thermophilic, xylanolytic bacterium. Int J Syst Bacteriol. 1998;48(1):91–97.

579. Hamilton-Brehm SD, Mosher JJ, Vishnivetskaya T, Podar M, Carroll S, Allman S, et al. *Caldicellulosiruptor obsidiansis* sp. nov., an anaerobic, extremely thermophilic, cellulolytic bacterium isolated from Obsidian Pool, Yellowstone National Park. Appl Environ Microbiol. 2010;76(4):1014–1020.

580. Mathrani IM, Ahring BK. Isolation and characterization of a strictly xylan-degrading *Dictyoglomus* from a man-made, thermophilic anaerobic environment. Arch Microbiol. 1991;157(1):13–17.

581. Pikuta E, Lysenko A, Suzina N, Osipov G, Kuznetsov B, Tourova T, et al. *Desulfotomaculum alkaliphilum* sp. nov., a new alkaliphilic, moderately thermophilic, sulfate-reducing bacterium. Int J Syst Evol Microbiol. 2000;50(1):25–33.

582. Maranhão P, Marques JC. The influence of temperature and salinity on the duration of embryonic development, fecundity and growth of the amphipod *Echinogammarus marinus* Leach (Gammaridae). Acta Oecologica. 2003;24:5–13.

583. Cooper WE. Dynamics and production of a natural population of a fresh-water amphipod, *Hyalella azteca*. Ecol Monogr. 1965;p. 377–394.

584. Marchant R, Banat IM, Rahman TJ, Berzano M. The frequency and characteristics of highly thermophilic bacteria in cool soil environments. Environ Microbiol. 2002;4(10):595–602.

585. Mutzel A, Reinscheid UM, Antranikian G, Müller R. Isolation and characterization of a thermophilic bacillus strain, that degrades phenol and cresols as sole carbon source at 70 C. Appl Microbiol Biotechnol. 1996;46(5-6):593–596.

586. Fuchs T, Huber H, Teiner K, Burggraf S, Stetter KO. *Metallosphaera prunae*, sp. nov., a novel metal-mobilizing, thermoacidophilic *Archaeum*, isolated from a uranium mine in Germany. Syst Appl Microbiol. 1995;18(4):560–566.

587. Huber G, Spinnler C, Gambacorta A, Stetter KO. *Metallosphaera sedula* gen, and sp. nov. represents a new genus of aerobic, metal-Mobilizing, thermoacidophilic Archaebacteria. Syst Appl Microbiol. 1989;12(1):38–47.

588. Liu SY, Rainey FA, Morgan HW, Mayer F, Wiegel J. *Thermoanaerobacterium aotearoense* sp. nov., a slightly acidophilic, anaerobic thermophile isolated from various hot springs in New Zealand, and emendation of the genus *Thermoanaerobacterium*. Int J Syst Bacteriol. 1996;46(2):388–396.

589. Brand LE, Murphy LS, Guillard RRL, Lee Ht. Genetic variability and differentiation in the temperature niche component of the diatom *Thalassiosira pseudonana*. Mar Biol. 1981;62(2-3):103–110.

590. Durbin EG. Studies on the autecology of the marine diatom *Thalassiosira nordenskiöldii* Cleve. 1. The influence of daylength, light intensity, and temperature on growth. J Phycol. 1974;10(2):220–225.

591. Langdon C. On the causes of interspecific differences in the growth-irradiance relationship for phytoplankton. II. A general review. J Plankton Res. 1988;10(6):1291–1312.

592. Krawiec RW. Autecology and clonal variability of the marine centric diatom *Thalassiosira rotula* (Bacillariophyceae) in response to light, temperature and salinity. Mar Biol. 1982;69(1):79–89.

593. Smayda TJ. Experimental observations on the influence of temperature, light, and salinity on cell division of the marine diatom, *Detonula confervacea* (Cleve) Gran. J Phycol. 1969;5(2):150–157.

594. Verity PG. Effects of temperature, irradiance, and daylength on the marine diatom *Leptocylindrus danicus* Cleve. I. Photosynthesis and cellular composition. J Exp Mar Biol Ecol. 1981;55(1):79–91.

595. Yoder JA. Effect of temperature on light-limited growth and chemical composition of *Skeletonema costatum* (Bacillariophyceae). J Phycol. 1979;15(4):362–370.

596. Hensel R, Matussek K, Michalke K, Tacke L, Tindall BJ, Kohlhoff M, et al. *Sulfophobococcus zilligii* gen. nov., spec. nov. a novel hyperthermophilic archaeum isolated from hot alkaline springs of Iceland. Syst Appl Microbiol. 1997;20(1):102–110.

597. Botero LM, Brown KB, Brumefield S, Burr M, Castenholz RW, Young M, et al. *Thermobaculum terrenum* gen. nov., sp. nov.: a non-phototrophic gram-positive thermophile representing an environmental clone group related to the Chloroflexi (green non-sulfur bacteria) and Thermomicrobia. Arch Microbiol. 2004;181(4):269–277.

598. Baesman SM, Stolz JF, Kulp TR, Oremland RS. Enrichment and isolation of *Bacillus beveridgei* sp. nov., a facultative anaerobic haloalkaliphile from Mono Lake, California, that respires oxyanions of tellurium, selenium, and arsenic. Extremophiles. 2009;13(4):695–705.

599. Yamada T, Imachi H, Ohashi A, Harada H, Hanada S, Kamagata Y, et al. *Bellilinea caldifistulae* gen. nov., sp. nov. and *Longilinea arvoryzae* gen. nov., sp. nov., strictly anaerobic, filamentous bacteria of the phylum Chloroflexi isolated from methanogenic propionate-degrading consortia. Int J Syst Evol Microbiol. 2007;57(10):2299–2306.

600. Miyashita H, Ikemoto H, Kurano N, Miyachi S, Chihara M. *Acaryochloris marina* gen. et sp. nov. (Cyanobacteria), an oxygenic photosynthetic prokaryote containing chl d as a major pigment. J Phycol. 2003;39(6):1247–1253.

601. Guillard RRL, Ryther JH. Studies of marine planktonic diatoms: I. *Cyclotella nana* Hustedt, and *Detonula confervacea* (Cleve) Gran. Can J Microbiol. 1962;8(2):229–239.

602. Osburn MR, Amend JP. *Thermogladius shockii* gen. nov., sp. nov., a hyperthermophilic crenarchaeote from Yellowstone National Park, USA. Arch Microbiol. 2011;193(1):45–52.

603. Cheng L, Dai L, Li X, Zhang H, Lu Y. Isolation and characterization of *Methanothermobacter crinale* sp. nov., a novel hydrogenotrophic methanogen from the Shengli oil field. Appl Environ Microbiol. 2011;77(15):5212–5219.

604. Alves MP, Rainey FA, Nobre MF, da Costa MS. *Thermomonas hydrothermalis* sp. nov., A New Slightly Thermophilic $\gamma$-Proteobacterium Isolated from a Hot Spring in Central Portugal. Syst Appl Microbiol. 2003;26(1):70–75.

605. Sako Y, Nunoura T, Uchida A. *Pyrobaculum oguniense* sp. nov., a novel facultatively aerobic and hyperthermophilic archaeon growing at up to 97 degrees C. Int J Syst Evol Microbiol. 2001;51(2):303–309.

606. Takayanagi S, Kawasaki H, Sugimori K, Yamada T, Sugai A, Ito T, et al. *Sulfolobus hakonensis* sp. nov., a novel species of acidothermophilic archaeon. Int J Syst Bacteriol. 1996;46(2):377–382.

607. Odintsova EV, Jannasch HW, Mamone JA, Langworthy TA. *Thermothrix azorensis* sp. nov., an obligately chemolithoautotrophic, sulfur-oxidizing, thermophilic bacterium. Int J Syst Bacteriol. 1996;46(2):422–428.

608. Stöhr R, Waberski A, Liesack W, Völker H, Wehmeyer U, Thomm M. *Hydrogenophilus hirschii* sp. nov., a novel thermophilic hydrogen-oxidizing beta-proteobacterium isolated from Yellowstone National Park. Int J Syst Evol Microbiol. 2001;51(2):481–488.

609. Segerer AH, Trincone A, Gahrtz M, Stetter KO. *Stygiolobus azoricus* gen. nov., sp. nov. represents a novel genus of anaerobic, extremely thermoacidophilic archaebacteria of the order Sulfolobales. Int J Syst Bacteriol. 1991;41(4):495–501.

610. Krivushin KV, Shcherbakova VA, Petrovskaya LE, Rivkina EM. *Methanobacterium veterum* sp. nov., from ancient Siberian permafrost. Int J Syst Evol Microbiol. 2010;60(2):455–459.

611. Takai K, Hirayama H, Nakagawa T, Suzuki Y, Nealson KH, Horikoshi K. *Thiomicrospira thermophila* sp. nov., a novel microaerobic, thermotolerant, sulfur-oxidizing chemolithomixotroph isolated from a deep-sea hydrothermal fumarole in the TOTO caldera, Mariana Arc, Western Pacific. Int J Syst Evol Microbiol. 2004;54(6):2325–2333.

612. Jan RL, Wu J, Chaw SM, Tsai CW, Tsen SD. A novel species of thermoacidophilic archaeon, *Sulfolobus yangmingensis* sp. nov. Int J Syst Evol Microbiol. 1999;49(4):1809–1816.

613. Kurr M, Huber R, König H, Jannasch HW, Fricke H, Trincone A, et al. *Methanopyrus kandleri*, gen. and sp. nov. represents a novel group of hyperthermophilic methanogens, growing at 110 C. Arch Microbiol. 1991;156(4):239–247.

614. Zellner G, Stackebrandt E, Messner P, Tindall BJ, de Macario EC, Kneifel H, et al. *Methanocorpusculaceae* fam. nov., represented by *Methanocorpusculum parvum*, *Methanocorpusculum sinense* spec. nov. and *Methanocorpusculum bavaricum* spec. nov. Arch Microbiol. 1989;151(5):381–390.

615. Huber H, Stetter KO. *Thiobacillus prosperus* sp. nov., represents a new group of halotolerant metal-mobilizing bacteria isolated from a marine geothermal field. Arch Microbiol. 1989;151(6):479–485.

616. Blotevogel KH, Fischer U. Isolation and characterization of a new thermophilic and autotrophic methane producing bacterium: *Methanobacterium thermoaggregans* spec. nov. Arch Microbiol. 1985;142(3):218–222.

617. Prokofeva MI, Miroshnichenko ML, Kostrikina NA, Chernyh NA, Kuznetsov BB, Tourova TP, et al. *Acidilobus aceticus* gen. nov., sp. nov., a novel anaerobic thermoacidophilic archaeon from continental hot vents in Kamchatka. Int J Syst Evol Microbiol. 2000;50(6):2001–2008.

618. Xiang X, Dong X, Huang L. *Sulfolobus tengchongensis* sp. nov., a novel thermoacidophilic archaeon isolated from a hot spring in Tengchong, China. Extremophiles. 2003;7(6):493–498.

619. Williams TJ, Lefèvre CT, Zhao W, Beveridge TJ, Bazylinski DA. *Magnetospira thiophila* gen. nov., sp. nov., a marine magnetotactic bacterium that represents a novel lineage within the Rhodospirillaceae (Alphaproteobacteria). Int J Syst Evol Microbiol. 2012;62(Pt 10):2443–2450.

620. Jochimsen B, Peinemann-Simon S, Völker H, Stüben D, Botz R, Stoffers P, et al. *Stetteria hydrogenophila*, gen. nov. and sp. nov., a novel mixotrophic sulfur-dependent crenarchaeote isolated from Milos, Greece. Extremophiles. 1997;1(2):67–73.

621. Stetter KO, Thomm M, Winter J, Wildgruber G, Huber H, Zillig W, et al. *Methanothermus fervidus*, sp. nov., a novel extremely thermophilic methanogen isolated from an Icelandic hot spring. Zbl Bakt Hyg, I Abt Orig C. 1981;2(2):166–178.

622. Huber G, Stetter KO. *Sulfolobus metallicus*, sp. nov., a novel strictly chemolithoautotrophic thermophilic archaeal species of metal-mobilizers. Syst Appl Microbiol. 1991;14(4):372–378.

623. Du Preez JC, Toerien DF, Lategan PM. Growth parameters of *Acinetobacter calcoaceticus* on acetate and ethanol. Eur J Appl Microbiol. 1981;13(1):45–53.

624. Huber R, Rossnagel P, Woese CR, Rachel R, Langworthy TA, Stetter KO. Formation of ammonium from nitrate during chemolithoautotrophic growth of the extremely thermophilic bacterium *Ammonifex degensii* gen. nov. sp. nov. Syst Appl Microbiol. 1996;19(1):40–49.

625. Patel GB, Sprott GD, Fein JE. Isolation and characterization of *Methanobacterium espanolae* sp. nov., a mesophilic, moderately acidiphilic methanogen. Int J Syst Bacteriol. 1990;40(1):12–18.

626. Palop ML, Valles S, Pinaga F, Flors A. Isolation and characterization of an anaerobic, cellulolytic bacterium, *Clostridium celerecrescens* sp. nov. Int J Syst Bacteriol. 1989;39(1):68–71.

627. Liu Y, Boone DR, Choy C. *Methanohalophilus oregonense* sp. nov., a methylotrophic methanogen from an alkaline, saline aquifer. Int J Syst Bacteriol. 1990;40(2):111–116.

628. Chung AP, Rainey FA, Valente M, Nobre MF, da Costa MS. *Thermus igniterrae* sp. nov. and *Thermus antranikianii* sp. nov., two new species from Iceland. Int J Syst Evol Microbiol. 2000;50(1):209–217.

629. Denariaz G, Payne WJ, Le Gall J. A halophilic denitrifier, *Bacillus halodenitrificans* sp. nov. Int J Syst Bacteriol. 1989;39(2):145–151.

630. Lyimo TJ, Pol A, Op den Camp HJ, Harhangi HR, Vogels GD. *Methanosarcina semesiae* sp. nov., a dimethylsulfide-utilizing methanogen from mangrove sediment. Int J Syst Evol Microbiol. 2000;50(1):171–178.

631. Burger-Wiersma T, Stal LJ, Mur LR. *Prochlorthrix hollandica* gen. nov. sp. nov., a filamentous oxygenic photoautotrophic procaryote containing chlorophylls a and b: Assignment to *Prochlorotrichaceae* fam. nov. and order *Prochlorales* Florenzano, Balloni, and Materassi 1986, with emendation of the ordinal description. Int J Syst Bacteriol. 1989;39(3):250–257.

632. Boone DR, Mathrani IM, Liu Y, Menaia JAGF, Mah RA, Boone JE. Isolation and characterization of *Methanohalophilus portucalensis* sp. nov. and DNA reassociation study of the genus *Methanohalophilus*. Int J Syst Bacteriol. 1993;43(3):430–437.

633. Jiang B, Parshina SN, Van Doesburg W, Lomans BP, Stams AJM. *Methanomethylovorans thermophila* sp. nov., a thermophilic, methylotrophic methanogen from an anaerobic reactor fed with methanol. Int J Syst Evol Microbiol. 2005;55(6):2465–2470.

634. Boone DR, Liu Y, Zhao ZJ, Balkwill DL, Drake GR, Stevens TO, et al. *Bacillus infernus* sp. nov., an Fe (III)-and Mn (IV)-reducing anaerobe from the deep terrestrial subsurface. Int J Syst Bacteriol. 1995;45(3):441–448.

635. Tenreiro S, Nobre MF, Da Costa MS. *Thermus silvanus* sp. nov. and *Thermus chliarophilus* sp. nov., two new species related to *Thermus ruber* but with lower growth temperatures. Int J Syst Bacteriol. 1995;45(4):633–639.

636. Nakagawa S, Takai K, Inagaki F, Horikoshi K, Sako Y. *Nitratiruptor tergarcus* gen. nov., sp. nov. and *Nitratifractor salsuginis* gen. nov., sp. nov., nitrate-reducing chemolithoautotrophs of the $\varepsilon$-Proteobacteria isolated from a deep-sea hydrothermal system in the Mid-Okinawa Trough. Int J Syst Evol Microbiol. 2005;55(2):925–933.

637. Bogdanova TI, Tsaplina IA, Kondrat'eva TF, Duda VI, Suzina NE, Melamud VS, et al. *Sulfobacillus thermotolerans* sp. nov., a thermotolerant, chemolithotrophic bacterium. Int J Syst Evol Microbiol. 2006;56(5):1039–1042.

638. Sakai S, Imachi H, Hanada S, Ohashi A, Harada H, Kamagata Y. *Methanocella paludicola* gen. nov., sp. nov., a methane-producing archaeon, the first isolate of the lineage 'Rice Cluster I', and proposal of the new archaeal order Methanocellales ord. nov. Int J Syst Evol Microbiol. 2008;58(4):929–936.

639. Sprenger WW, van Belzen MC, Rosenberg J, Hackstein JH, Keltjens JT. *Methanomicrococcus blatticola* gen. nov., sp. nov., a methanol-and methylamine-reducing methanogen from the hindgut of the cockroach *Periplaneta americana*. Int J Syst Evol Microbiol. 2000;50(6):1989–1999.

640. Kratz WA, Myers J. Nutrition and growth of several blue-green algae. Am J Bot. 1955;p. 282–287.

641. Sowers KR, Ferry JG. Isolation and characterization of a methylotrophic marine methanogen, *Methanococcoides methylutens* gen. nov., sp. nov. Appl Environ Microbiol. 1983;45(2):684–690.

642. Pley U, Schipka J, Gambacorta A, Jannasch HW, Fricke H, Rachel R, et al. *Pyrodictium abyssi* sp. nov. represents a novel heterotrophic marine archaeal hyperthermophile growing at 110 C. Syst Appl Microbiol. 1991;14(3):245–253.

643. Drent WJ, Lahpor GA, Wiegant WM, Gottschal JC. Fermentation of inulin by *Clostridium thermosuccinogenes* sp. nov., a thermophilic anaerobic bacterium isolated from various habitats. Appl Environ Microbiol. 1991;57(2):455–462.

644. Liaw HJ, Mah RA. Isolation and characterization of *Haloanaerobacter chitinovorans* gen. nov., sp. nov., a halophilic, anaerobic, chitinolytic bacterium from a solar saltern. Appl Environ Microbiol. 1992;58(1):260–266.

645. Kashefi K, Holmes DE, Reysenbach AL, Lovley DR. Use of Fe (III) as an electron acceptor to recover previously uncultured hyperthermophiles: isolation and characterization of *Geothermobacterium ferrireducens* gen. nov., sp. nov. Appl Environ Microbiol. 2002;68(4):1735–1742.

646. Kaksonen AH, Plumb JJ, Robertson WJ, Spring S, Schumann P, Franzmann PD, et al. Novel thermophilic sulfate-reducing bacteria from a geothermally active underground mine in Japan. Appl Environ Microbiol. 2006;72(5):3759–3762.

647. Thrash JC, Ahmadi S, Torok T, Coates JD. *Magnetospirillum bellicus* sp. nov., a novel dissimilatory perchlorate-reducing alphaproteobacterium isolated from a bioelectrical reactor. Appl Environ Microbiol. 2010;76(14):4730–4737.

648. Wu SY, Lai MC. Methanogenic archaea isolated from Taiwan's Chelungpu Fault. Appl Environ Microbiol. 2011;77(3):830–838.

649. Cao Y, Chastain RA, Eloe EA, Nogi Y, Kato C, Bartlett DH. Novel psychropiezophilic Oceanospirillales species *Profundimonas piezophila* gen. nov., sp. nov., isolated from the deep-sea environment of the Puerto Rico Trench. Appl Environ Microbiol. 2014;80(1):54–60.

650. Mladenovska Z, Mathrani IM, Ahring BK. Isolation and characterization of *Caldicellulosiruptor lactoaceticus* sp. nov., an extremely thermophilic, cellulolytic, anaerobic bacterium. Arch Microbiol. 1995;163(3):223–230.

651. Jones WJ, Paynter MJB, Gupta R. Characterization of *Methanococcus maripaludis* sp. nov., a new methanogen isolated from salt marsh sediment. Arch Microbiol. 1983;135(2):91–97.

652. Rabus R, Brüchert V, Amann J, Könneke M. Physiological response to temperature changes of the marine, sulfate-reducing bacterium *Desulfobacterium autotrophicum.* FEMS Microbiol Ecol. 2002;42(3):409–417.

653. Tiago I, Pires C, Mendes V, Morais PV, Da Costa M, Veríssimo A. *Microcella putealis* gen. nov., sp. nov., a Gram-positive alkaliphilic bacterium isolated from a nonsaline alkaline groundwater. Syst Appl Microbiol. 2005;28(6):479–487.

654. Tiago I, Mendes V, Pires C, Morais PV, Veríssimo A. *Chimaereicella alkaliphila* gen. nov., sp. nov., a Gram-negative alkaliphilic bacterium isolated from a nonsaline alkaline groundwater. Syst Appl Microbiol. 2006;29(2):100–108.

655. Parshina SN, Ermakova AV, Bomberg M, Detkova EN. *Methanospirillum stamsii* sp. nov., a psychrotolerant, hydrogenotrophic, methanogenic archaeon isolated from an anaerobic expanded granular sludge bed bioreactor operated at low temperature. Int J Syst Evol Microbiol. 2014;64(Pt 1):180–186.

656. Yi H, Chun J. *Flavobacterium weaverense* sp. nov. and *Flavobacterium segetis* sp. nov., novel psychrophiles isolated from the Antarctic. Int J Syst Evol Microbiol. 2006;56(6):1239–1244.

657. Nogi Y, Hosoya S, Kato C, Horikoshi K. *Colwellia piezophila* sp. nov., a novel piezophilic species from deep-sea sediments of the Japan Trench. Int J Syst Evol Microbiol. 2004;54(5):1627–1631.

658. Sattley WM, Madigan MT. Isolation, characterization, and ecology of cold-active, chemolithotrophic, sulfur-oxidizing bacteria from perennially ice-covered Lake Fryxell, Antarctica. Appl Environ Microbiol. 2006;72(8):5562–5568.

659. Bhadsavle CH, Shehata TE, Collins EB. Isolation and identification of psychrophilic species of *Clostridium* from milk. Appl Microbiol. 1972;24(5):699–702.

660. Canion A, Prakash O, Green SJ, Jahnke L, Kuypers MMM, Kostka JE. Isolation and physiological characterization of psychrophilic denitrifying bacteria from permanently cold Arctic fjord sediments (Svalbard, Norway). Environ Microbiol. 2013;15(5):1606–1618.

661. Bajard S, Rosso L, Fardel G, Flandrois JP. The particular behaviour of *Listeria monocytogenes* under sub-optimal conditions. Int J Food Microbiol. 1996;29(2):201–211.

662. Moschonas G, Bolton DJ. Characterization of a potentially novel 'blown pack' spoilage bacterium isolated from bovine hide. J Appl Microbiol. 2013;114(3):771–777.

663. Shcherbakova VA, Chuvil'skaya NA, Rivkina EM, Pecheritsyna SA, Suetin SV, Laurinavichius KS, et al. Novel halotolerant bacterium from cryopeg in permafrost: Description of *Psychrobacter muriicola* sp. nov. Microbiology. 2009;78(1):84–91.

664. Spring S, Merkhoffer B, Weiss N, Kroppenstedt RM, Hippe H, Stackebrandt E. Characterization of novel psychrophilic clostridia from an Antarctic microbial mat: description of *Clostridium frigoris* sp. nov., *Clostridium lacusfryxellense* sp. nov., *Clostridium bowmanii* sp. nov. and *Clostridium psychrophilum* sp. nov. and reclassification of *Clostridium laramiense* as *Clostridium estertheticum* subsp. *laramiense* subsp. nov. Int J Syst Evol Microbiol. 2003;53(4):1019–1029.

665. Hokanson KEF, Kleiner CF, Thorslund TW. Effects of constant temperatures and diel temperature fluctuations on specific growth and mortality rates and yield of juvenile rainbow trout, *Salmo gairdneri*. J Fish Res Board Can. 1977;34(5):639–648.

666. Montagnes DJS, Franklin DJ. Effect of temperature on diatom volume, growth rate, and carbon and nitrogen content: reconsidering some paradigms. Limnol Oceanogr. 2001;46(8):2008–2018.

667. Christian RR, Wiebe WJ. The effects of temperature upon the reproduction and respiration of a marine obligate psychrophile. Can J Microbiol. 1974;20(10):1341–1345.

668. Gounot AM. Effects of temperature on the growth of psychrophilic bacteria from glaciers. Can J Microbiol. 1976;22(6):839–846.

669. Shehata TE, Duran A, Collins EB. Influence of temperature on the growth of psychrophilic strains of *Bacillus*. J Dairy Sci. 1971;54(11):1579–1582.

670. Sonne-Hansen J, Ahring BK. *Thermodesulfobacterium hveragerdense* sp. nov., and *Thermodesulfovibrio islandicus* sp. nov., two thermophilic sulfate reducing bacteria isolated from a Icelandic hot spring. Syst Appl Microbiol. 1999;22(4):559–564.

671. Miranda-Tello E, Fardeau ML, Fernández L, Ramírez F, Cayol JL, Thomas P, et al. *Desulfovibrio capillatus* sp. nov., a novel sulfate-reducing bacterium isolated from an oil field separator located in the Gulf of Mexico. Anaerobe. 2003;9(2):97–103.

672. Singh N, Kendall MM, Liu Y, Boone DR. Isolation and characterization of methylotrophic methanogens from anoxic marine sediments in Skan Bay, Alaska: description of *Methanococcoides alaskense* sp. nov., and emended description of *Methanosarcina baltica*. Int J Syst Evol Microbiol. 2005;55(6):2531–2538.

673. Li Y, Engle M, Weiss N, Mandelco L, Wiegel J. *Clostridium thermoalcaliphilum* sp. nov., an anaerobic and thermotolerant facultative alkaliphile. Int J Syst Bacteriol. 1994;44(1):111–118.

674. Menes RJ, Muxí L. *Anaerobaculum mobile* sp. nov., a novel anaerobic, moderately thermophilic, peptide-fermenting bacterium that uses crotonate as an electron acceptor, and emended description of the genus *Anaerobaculum*. Int J Syst Evol Microbiol. 2002;52(1):157–164.

675. Li Y, Mandelco L, Wiegel J. Isolation and characterization of a moderately thermophilic anaerobic alkaliphile, *Clostridium paradoxum* sp. nov. Int J Syst Bacteriol. 1993;43(3):450–460.

676. Greene AC, Patel BKC, Sheehy AJ. *Deferribacter thermophilus* gen. nov., sp. nov., a novel thermophilic manganese-and iron-reducing bacterium isolated from a petroleum reservoir. Int J Syst Bacteriol. 1997;47(2):505–509.

677. Reichardt W, Morita RY. Temperature characteristics of psychrotrophic and psychrophilic bacteria. J Gen Microbiol. 1982;128(3):565–568.

678. Cayol JL, Ollivier B, Patel BKC, Ravot G, Magot M, Ageron E, et al. Description of *Thermoanaerobacter brockii* subsp. *lactiethylicus* subsp. nov., isolated from a deep subsurface French oil well, a proposal to reclassify *Thermoanaerobacter finnii* as *Thermoanaerobacter brockii* subsp. *finnii* comb. nov., and an emended description of *Thermoanaerobacter brockii*. Int J Syst Evol Microbiol. 1995;45:783–789.

679. Magot M, Ravot G, Campaignolle X, Ollivier B, Patel BK, Fardeau ML, et al. *Dethiosulfovibrio peptidovorans* gen. nov., sp. nov., a new anaerobic, slightly halophilic, thiosulfate-reducing bacterium from corroding offshore oil wells. Int J Syst Bacteriol. 1997;47(3):818–824.

680. Ollivier B, Fardeau ML, Cayol JL, Magot M, Patel BKC, Prensier G, et al. *Methanocalculus halotolerans* gen. nov., sp. nov., isolated from an oil-producing well. Int J Syst Bacteriol. 1998;48(3):821–828.

681. Tsai CR, Garcia JL, Patel BK, Cayol JL, Baresi L, Mah RA. *Haloanaerobium alcaliphilum* sp. nov., an anaerobic moderate halophile from the sediments of Great Salt Lake, Utah. Int J Syst Bacteriol. 1995;45(2):301–307.

682. Cayol JL, Ollivier B, Patel BKC, Ageron E, Grimont P, Prensier G, et al. *Haloanaerobium lacusroseus* sp. nov., an extremely halophilic fermentative bacterium from the sediments of a hypersaline lake. Int J Syst Bacteriol. 1995;45(4):790–797.

683. Fardeau ML, Ollivier B, Patel BKC, Magot M, Thomas P, Rimbault A, et al. *Thermotoga hypogea* sp. nov., a xylanolytic, thermophilic bacterium from an oil-producing well. Int J Syst Bacteriol. 1997;47(4):1013–1019.

684. Elberson MA, Sowers KR. Isolation of an aceticlastic strain of *Methanosarcina siciliae* from marine canyon sediments and emendation of the species description for *Methanosarcina siciliae*. Int J Syst Bacteriol. 1997;47(4):1258–1261.

685. Zavarzina D, Zhilina T, Tourova T, Kuznetsov B, Kostrikina N, Bonch-Osmolovskaya E. *Thermanaerovibrio velox* sp. nov., a new anaerobic, thermophilic, organotrophic bacterium that reduces elemental sulfur, and emended description of the genus *Thermanaerovibrio*. Int J Syst Evol Microbiol. 2000;50(3):1287–1295.

686. Slobodkin AI, Tourova TP, Kuznetsov BB, Kostrikina NA, Chernyh NA, Bonch-Osmolovskaya EA. *Thermoanaerobacter siderophilus* sp. nov., a novel dissimilatory Fe (III)-reducing, anaerobic, thermophilic bacterium. Int J Syst Evol Microbiol. 1999;49(4):1471–1478.

687. Kim BC, Grote R, Lee DW, Antranikian G, Pyun YR. *Thermoanaerobacter yonseiensis* sp. nov., a novel extremely thermophilic, xylose-utilizing bacterium that grows at up to 85 degrees C. Int J Syst Evol Microbiol. 2001;51(4):1539–1548.

688. Mori K, Hanada S, Maruyama A, Marumo K. *Thermanaeromonas toyohensis* gen. nov., sp. nov., a novel thermophilic anaerobe isolated from a subterranean vein in the Toyoha Mines. Int J Syst Evol Microbiol. 2002;52(5):1675–1680.

689. Lai MC, Chen SC, Shu CM, Chiou MS, Wang CC, Chuang MJ, et al. *Methanocalculus taiwanensis* sp. nov., isolated from an estuarine environment. Int J Syst Evol Microbiol. 2002;52(5):1799–1806.

690. Stannard CJ, Williams AP, Gibbs PA. Temperature/growth relationships for psychrotrophic food-spoilage bacteria. Food Microbiol. 1985;2(2):115–122.

691. Mayo AW. Effects of temperature and pH on the kinetic growth of unialga *Chlorella vulgaris* cultures containing bacteria. Water Environ Res. 1997;p. 64–72.

692. Alain K, Callac N, Guégan M, Lesongeur F, Crassous P, Cambon-Bonavita MA, et al. *Nautilia abyssi* sp. nov., a thermophilic, chemolithoautotrophic, sulfur-reducing bacterium isolated from an East Pacific Rise hydrothermal vent. Int J Syst Evol Microbiol. 2009;59(6):1310–1315.

693. Guillou C, Guespin-Michel JF. Evidence for two domains of growth temperature for the psychrotrophic bacterium *Pseudomonas fluorescens* MF0. Appl Environ Microbiol. 1996;62(9):3319–3324.

694. Rouf MA, Rigney MM. Growth temperatures and temperature characteristics of *Aeromonas*. Appl Microbiol. 1971;22(4):503–506.

695. Kinnunen PM, Robertson W, Plumb JJ, Gibson JAE, Nichols PD, Franzmann PD, et al. The isolation and use of iron-oxidizing, moderately thermophilic acidophiles from the Collie coal mine for the generation of ferric iron leaching solution. Appl Microbiol Biotechnol. 2003;60(6):748–753.

696. Thevenieau F, Fardeau ML, Ollivier B, Joulian C, Baena S. *Desulfomicrobium thermophilum* sp. nov., a novel thermophilic sulphate-reducing bacterium isolated from a terrestrial hot spring in Colombia. Extremophiles. 2007;11(2):295–303.

697. Wang L, Tang Y, Wang S, Liu RL, Liu MZ, Zhang Y, et al. Isolation and characterization of a novel thermophilic Bacillus strain degrading long-chain n-alkanes. Extremophiles. 2006;10(4):347–356.

698. Melamud VS, Pivovarova TA, Tourova TP, Kolganova TV, Osipov GA, Lysenko AM, et al. *Sulfobacillus sibiricus* sp. nov., a new moderately thermophilic bacterium. Microbiology. 2003;72(5):605–612.

699. Ogata T, Ishimaru T, Kodama M. Effect of water temperature and light intensity on growth rate and toxicity change in *Protogonyaulax tamarensis*. Mar Biol. 1987;95(2):217–220.

700. Rigano C, Violante U. Comparative growth of the thermal alga *Cyanidium caldarium* on nitrate and ammonia at different temperatures. Arch Microbiol. 1972;85(1):13–18.

701. Herbert RA, Bell CR. Growth characteristics of an obligately psychrophilic *Vibrio* sp. Arch Microbiol. 1977;113(3):215–220.

702. Seaburg KG, Parked BC, Wharton RA, Simmons GM. Temperature-growth responses of algal isolates from Antarctic oases. J Phycol. 1981;17(4):353–360.

703. Teoh ML, Chu WL, Marchant H, Phang SM. Influence of culture temperature on the growth, biochemical composition and fatty acid profiles of six Antarctic microalgae. J Appl Phycol. 2004;16(6):421–430.

704. Teoh ML, Phang SM, Chu WL. Response of Antarctic, temperate, and tropical microalgae to temperature stress. J Appl Phycol. 2013;25(1):285–297.

705. Kutlu B, Buyukisik B. Investigations on the growth kinetics of *Hantzschia amphioxys* homa lagoon by izmir bay (Aegean Sea). Indian J Geo-Mar Sci. 2011;40(4):522.

706. Olsen RH, Jezeski JJ. Some effects of carbon source, aeration, and temperature on growth of a psychrophilic strain of *Pseudomonas fluorescens*. J Bacteriol. 1963;86(3):429–433.

707. Wiegel J, Ljungdahl LG, Rawson JR. Isolation from soil and properties of the extreme thermophile *Clostridium thermohydrosulfuricum*. J Bacteriol. 1979;139(3):800–810.

708. Healey FP. Effect of temperature and light intensity on the growth rate of *Synura sphagnicola*. J Plankton Res. 1983;5(5):767–774.

709. Baig IA, Hopton JW. Psychrophilic properties and the temperature characteristic of growth of bacteria. J Bacteriol. 1969;100(1):552.

710. Tillich UM, Wolter N, Franke P, Dühring U, Frohme M. Screening and genetic characterization of thermo-tolerant *Synechocystis* sp. PCC6803 strains created by adaptive evolution. BMC Biotechnol. 2014;14(1):66.

711. Orcutt Jr JD, Porter KG. The synergistic effects of temperature and food concentration of life history parameters of *Daphnia*. Oecologia. 1984;63(3):300–306.

712. Smith RE, Stapleford LC, Ridings RS. The acclimated response of growth, photosynthesis, composition, and carbon balance to temperature in the psychrophilic ice diatom *Nitzschia seriata*. J Phycol. 1994;30(1):8–16.

713. Fiala M, Oriol L. Light-temperature interactions on the growth of Antarctic diatoms. Polar Biol. 1990;10(8):629–636.

714. Van Baalen C, O'Donnell R. Isolation and growth of psychrophilic diatoms from the ice-edge in the Bering Sea. J Gen Microbiol. 1983;129(4):1019–1023.

715. Nunoura T, Hirai M, Imachi H, Miyazaki M, Makita H, Hirayama H, et al. *Kosmotoga arenicorallina* sp. nov. a thermophilic and obligately anaerobic heterotroph isolated from a shallow hydrothermal system occurring within a coral reef, southern part of the Yaeyama Archipelago, Japan, reclassification of *Thermococcoides shengliensis* as *Kosmotoga shengliensis* comb. nov., and emended description of the genus *Kosmotoga*. Arch Microbiol. 2010;192(10):811–819.

716. Phillips WE, Perry JJ. *Thermomicrobium fosteri* sp. nov., a hydrocarbon-utilizing obligate thermophile. Int J Syst Bacteriol. 1976;26(2):220–225.

717. L'Haridon S, Cilia V, Messner P, Raguenes G, Gambacorta A, Sleytr UB, et al. Desulfurobacterium thermolithotrophum gen. nov., sp. nov., a novel autotrophic, sulphur-reducing bacterium isolated from a deep-sea hydrothermal vent. Int J Syst Bacteriol. 1998;48(3):701–711.

718. Slobodkin A, Reysenbach AL, Mayer F, Wiegel J. Isolation and characterization of the homoacetogenic thermophilic Bacterium *Moorella glycerini* sp. nov. Int J Syst Bacteriol. 1997;47(4):969–974.

719. Harris JE, Pinn PA, Davis RP. Isolation and characterization of a novel thermophilic, freshwater methanogen. Appl Environ Microbiol. 1984;48(6):1123–1128.

720. Zabel HP, König H, Winter J. Isolation and characterization of a new coccoid methanogen, *Methanogenium tatii* spec. nov. from a solfataric field on Mount Tatio. Arch Microbiol. 1984;137(4):308–315.

721. Ollivier B, Cordruwisch R, Lombardo A, Garcia JL. Isolation and characterization of *Sporomusa acidovorans* sp. nov., a methylotrophic homoacetogenic bacterium. Arch Microbiol. 1985;142(3):307–310.

722. Dolhi JM, Maxwell DP, Morgan-Kiss RM. Review: The Antarctic *Chlamydomonas raudensis*: an emerging model for cold adaptation of photosynthesis. Extremophiles. 2013;17(5):711–722.

723. Podosokorskaya OA, Bonch-Osmolovskaya EA, Beskorovaynyy AV, Toshchakov SV, Kolganova TV, Kublanov IV. *Mobilitalea sibirica* gen. nov., sp. nov., a halotolerant polysaccharide-degrading bacterium. Int J Syst Evol Microbiol. 2014;64(Pt 8):2657–2661.

724. Pocock T, Vetterli A, Falk S. Evidence for phenotypic plasticity in the Antarctic extremophile *Chlamydomonas raudensis* Ettl. UWO 241. J Exp Bot. 2011;62(3):1169–1177.

725. Zabel HP, König H, Winter J. Emended description of *Methanogenium thermophilicum*, Rivard and Smith, and assignment of new isolates to this species. Syst Appl Microbiol. 1985;6(1):72–78.

726. Huber H, Jannasch H, Rachel R, Fuchs T, Stetter KO. *Archaeoglobus veneficus* sp. nov., a novel facultative chemolithoautotrophic hyperthermophilic sulfite reducer, isolated from abyssal black smokers. Syst Appl Microbiol. 1997;20(3):374–380.

727. Giovannelli D, Grosche A, Starovoytov V, Yakimov M, Manini E, Vetriani C. *Galenea microaerophila* gen. nov., sp. nov., a mesophilic, microaerophilic, chemosynthetic, thiosulfate-oxidizing bacterium isolated from a shallow-water hydrothermal vent. Int J Syst Evol Microbiol. 2012;62(Pt 12):3060–3066.

728. Tenreiro S, Nobre MF, Rainey FA, Miguel C, Da Costa MS. *Thermonema rossianum* sp. nov., a new thermophilic and slightly halophilic species from saline hot springs in Naples, Italy. Int J Syst Bacteriol. 1997;47(1):122–126.

729. Ni S, Boone DR. Isolation and characterization of a dimethyl sulfide-degrading methanogen, *Methanolobus siciliae* HI350, from an oil well, characterization of *M. siciliae* T4/MT, and emendation of *M. siciliae*. Int J Syst Bacteriol. 1991;41(3):410–416.

730. Engle M, Li Y, Woese C, Wiegel J. Isolation and characterization of a Novel Alkalitolerant Thermophile, *Anaerobranca horikoshii* gen. nov., sp. nov. Int J Syst Bacteriol. 1995;45(3):454–461.

731. Carreto L, Moore E, Nobre MF, Wait R, Riley PW, Sharp RJ, et al. *Rubrobacter xylanophilus* sp. nov., a new thermophilic species isolated from a thermally polluted effluent. Int J Syst Bacteriol. 1996;46(2):460–465.

732. Miyazaki M, Sakai S, Ritalahti KM, Saito Y, Yamanaka Y, Saito Y, et al. *Sphaerochaeta multiformis* sp. nov., an anaerobic, psychrophilic

bacterium isolated from subseafloor sediment, and emended description of the genus *Sphaerochaeta*. Int J Syst Evol Microbiol. 2014;64(Pt 12):4147–4154.

733. Chrisostomos S, Patel B, Dwivedi PP, Denman SE. *Caloramator indicus* sp. nov., a new thermophilic anaerobic bacterium isolated from the deep-seated nonvolcanically heated waters of an Indian artesian aquifer. Int J Syst Bacteriol. 1996;46(2):497–501.

734. Engle M, Li Y, Rainey F, DeBlois S, Mai V, Reichert A, et al. *Thermobrachium celere* gen. nov., sp. nov., a rapidly growing thermophilic, alkalitolerant, and proteolytic obligate anaerobe. Int J Syst Bacteriol. 1996;46(4):1025–1033.

735. Svetlitshnyi V, Rainey F, Wiegel J. *Thermosyntropha lipolytica* gen. nov., sp. nov., a lipolytic, anaerobic, alkalitolerant, thermophilic bacterium utilizing short-and long-chain fatty acids in syntrophic coculture with a methanogenic archaeum. Int J Syst Bacteriol. 1996;46(4):1131–1137.

736. Stohr R, Waberski A, Völker H, Tindall BJ, Thomm M. *Hydrogenothermus marinus* gen. nov., sp. nov., a novel thermophilic hydrogen-oxidizing bacterium, recognition of *Calderobacterium hydrogenophilum* as a member of the genus *Hydrogenobacter* and proposal of the reclassification of *Hydrogenobacter acidophilus* as *Hydrogenobaculum acidophilum* gen. nov., comb. nov., in the phylum 'Hydrogenobacter/Aquifex'. Int J Syst Evol Microbiol. 2001;51(5):1853–1862.

737. Huber R, Kurr M, Jannasch HW, Stetter KO. A novel group of abyssal methanogenic archaebacteria (*Methanopyrus*) growing at 110 C. Nature. 1989;342(6251):833–834.

738. Darland G, Brock TD, Samsonoff W, Conti SF. A thermophilic, acidophilic mycoplasma isolated from a coal refuse pile. Science. 1970;170(3965):1416–1418.

739. Brannan DK, Caldwell DE. *Thermothrix thiopara*: growth and metabolism of a newly isolated thermophile capable of oxidizing sulfur and sulfur compounds. Appl Environ Microbiol. 1980;40(2):211–216.

740. Huber H, Thomm M, König H, Thies G, Stetter KO. *Methanococcus thermolithotrophicus*, a novel thermophilic lithotrophic methanogen. Arch Microbiol. 1982;132(1):47–50.

741. Zillig W, Yeats S, Holz I, Böck A, Rettenberger M, Gropp F, et al. *Desulfurolobus ambivalens*, gen. nov., sp. nov., an autotrophic archaebacterium facultatively oxidizing or reducing sulfur. Syst Appl Microbiol. 1986;8(3):197–203.

742. Miroshnichenko ML, Bonch-Osmolovskaya EA, Neuner A, Kostrikina NA, Chernych NA, Alekseev VA. *Thermococcus stetteri* sp. nov., a new extremely thermophilic marine sulfur-metabolizing archaebacterium. Syst Appl Microbiol. 1989;12(3):257–262.

743. Kotelnikova S, Obraztsova AY, Gongadze G, Laurinavichius K. *Methanobacterium thermoflexum* sp. nov. and *Methanobacterium defluvii* sp. nov., thermophilic rod-shaped methanogens isolated from anaerobic digestor sludge. Syst Appl Microbiol. 1993;16(3):427–435.

744. Burggraf S, Fricke H, Neuner A, Kristjansson J, Rouvier P, Mandelco L, et al. *Methanococcus igneus* sp. nov., a novel hyperthermophilic methanogen from a shallow submarine hydrothermal system. Syst Appl Microbiol. 1990;13(3):263–269.

745. Burggraf S, Jannasch HW, Nicolaus B, Stetter KO. *Archaeoglobus profundus* sp. nov., represents a new species within the sulfate-reducing archaebacteria. Syst Appl Microbiol. 1990;13(1):24–28.

746. Kobayashi T, Kwak YS, Akiba T, Kudo T, Horikoshi K. *Thermococcus profundus* sp. nov., a new hyperthermophilic archaeon isolated from a deep-sea hydrothermal vent. Syst Appl Microbiol. 1994;17(2):232–236.

747. Plugge CM, Balk M, Zoetendal EG, Stams AJM. *Gelria glutamica* gen. nov., sp. nov., a thermophilic, obligately syntrophic, glutamate-degrading anaerobe. Int J Syst Evol Microbiol. 2002;52(2):401–407.

748. Hernández-Eugenio G, Fardeau ML, Cayol JL, Patel BKC, Thomas P, Macarie H, et al. *Clostridium thiosulfatireducens* sp. nov., a proteolytic, thiosulfate-and sulfur-reducing bacterium isolated from an upflow anaerobic sludge blanket (UASB) reactor. Int J Syst Evol Microbiol. 2002;52(5):1461–1468.

749. Zeikus J, Hegge P, Anderson MA. *Thermoanaerobium brockii* gen. nov. and sp. nov., a new chemoorganotrophic, caldoactive, anaerobic bacterium. Arch Microbiol. 1979;122(1):41–48.

750. Daumas S, Cord-Ruwisch R, Garcia JL. *Desulfotomaculum geothermicum* sp. nov., a thermophilic, fatty acid-degrading, sulfate-reducing bacterium isolated with H2 from geothermal ground water. Antonie van Leeuwenhoek. 1988;54(2):165–178.

751. Alain K, Rolland S, Crassous P, Lesongeur F, Zbinden M, Le Gall C, et al. *Desulfurobacterium crinifex* sp. nov., a novel thermophilic, pinkish-streamer forming, chemolithoautotrophic bacterium isolated from a Juan de Fuca Ridge hydrothermal vent and amendment of the genus *Desulfurobacterium.* Extremophiles. 2003;7(5):361–370.

752. Cha IT, Min UG, Kim SJ, Yim KJ, Roh SW, Rhee SK. *Methanomethylovorans uponensis* sp. nov., a methylotrophic methanogen isolated from wetland sediment. Antonie van Leeuwenhoek. 2013;104(6):1005–1012.

753. Etchebehere C, Pavan ME, Zorzopulos J, Soubes M, Muxi L. *Coprothermobacter platensis* sp. nov., a new anaerobic proteolytic thermophilic bacterium isolated from an anaerobic mesophilic sludge. Int J Syst Bacteriol. 1998;48(4):1297–1304.

754. Méndez BS, Pettinari MJ, Ivanier SE, Ramos CA, Siñeriz F. *Clostridium thermopapyrolyticum* sp. nov., a cellulolytic thermophile. Int J Syst Bacteriol. 1991;41(2):281–283.

755. Miroshnichenko ML, Kostrikina NA, l'Haridon S, Jeanthon C, Hippe H, Stackebrandt E, et al. *Nautilia lithotrophica* gen. nov., sp. nov., a thermophilic sulfur-reducing $\epsilon$-proteobacterium isolated from a deep-sea hydrothermal vent. Int J Syst Evol Microbiol. 2002;52(4):1299–1304.

756. Ravot G, Magot M, Fardeau ML, Patel BK, Thomas P, Garcia JL, et al. *Fusibacter paucivorans* gen. nov., sp. nov., an anaerobic, thiosulfate-reducing bacterium from an oil-producing well. Int J Syst Bacteriol. 1999;49(3):1141–1147.

757. Lyon PF, Beffa T, Blanc M, Auling G, Aragno M. Isolation and characterization of highly thermophilic xylanolytic *Thermus thermophilus* strains from hot composts. Can J Microbiol. 2000;46(11):1029–1035.

758. Foesel BU, Rohde M, Overmann J. *Blastocatella fastidiosa* gen. nov., sp. nov., isolated from semiarid savanna soil–The first described species of Acidobacteria subdivision 4. Syst Appl Microbiol. 2013;36(2):82–89.

759. Dedysh SN, Kulichevskaya IS, Serkebaeva YM, Mityaeva MA, Sorokin VV, Suzina NE, et al. *Bryocella elongata* gen. nov., sp. nov., a member of subdivision 1 of the Acidobacteria isolated from a methanotrophic enrichment culture, and emended description of *Edaphobacter aggregans* Koch et al. 2008. Int J Syst Evol Microbiol. 2012;62(Pt 3):654–664.

760. Yumoto I, Hirota K, Nodasaka Y, Tokiwa Y, Nakajima K. *Alkalibacterium indicireducens* sp. nov., an obligate alkaliphile that reduces indigo dye. Int J Syst Evol Microbiol. 2008;58(4):901–905.

761. Ishikawa M, Nakajima K, Yanagi M, Yamamoto Y, Yamasato K. *Marinilactibacillus psychrotolerans* gen. nov., sp. nov., a halophilic and alkaliphilic marine lactic acid bacterium isolated from marine organisms in temperate and subtropical areas of Japan. Int J Syst Evol Microbiol. 2003;53(3):711–720.

762. Itoh T, Suzuki K, Sanchez PC, Nakase T. *Caldisphaera lagunensis* gen. nov., sp. nov., a novel thermoacidophilic crenarchaeote isolated from a hot spring at Mt Maquiling, Philippines. Int J Syst Evol Microbiol. 2003;53(4):1149–1154.

763. Nakajima K, Hirota K, Nodasaka Y, Yumoto I. *Alkalibacterium iburiense* sp. nov., an obligate alkaliphile that reduces an indigo dye. Int J Syst Evol Microbiol. 2005;55(4):1525–1530.

764. Huber KJ, Wüst PK, Rhode M, Overmann J, Foesel BU. *Aridibacter famidurans* and *Aridibacter kavangonensis*, 2 novel species of Acidobacteria subdivision 4 isolated from semiarid savanna soil. Int J Syst Evol Microbiol. 2014;p. ijs–0.

765. Yumoto I, Hirota K, Nodasaka Y, Yokota Y, Hoshino T, Nakajima K. *Alkalibacterium psychrotolerans* sp. nov., a psychrotolerant obligate alkaliphile that reduces an indigo dye. Int J Syst Evol Microbiol. 2004;54(6):2379–2383.

766. Khelaifia S, Fardeau ML, Pradel N, Aussignargues C, Garel M, Tamburini C, et al. *Desulfovibrio piezophilus* sp. nov., a piezophilic, sulfate-reducing bacterium isolated from wood falls in the Mediterranean Sea. Int J Syst Evol Microbiol. 2011;61(11):2706–2711.

767. Golyshina OV, Yakimov MM, Lünsdorf H, Ferrer M, Nimtz M, Timmis KN, et al. *Acidiplasma aeolicum* gen. nov., sp. nov., a euryarchaeon of the family Ferroplasmaceae isolated from a hydrothermal pool, and transfer of *Ferroplasma cupricumulans* to *Acidiplasma cupricumulans* comb. nov. Int J Syst Evol Microbiol. 2009;59(11):2815–2823.

768. Kulichevskaya IS, Suzina NE, Rijpstra WIC, Damsté JSS, Dedysh SN. *Paludibaculum fermentans* gen. nov., sp. nov., a facultative anaerobe capable of dissimilatory iron reduction from subdivision 3 of the Acidobacteria. Int J Syst Evol Microbiol. 2014;64(Pt 8):2857–2864.

769. Gruber C, Legat A, Pfaffenhuemer M, Radax C, Weidler G, Busse HJ, et al. *Halobacterium noricense* sp. nov., an archaeal isolate from a bore core of an alpine Permian salt deposit, classification of *Halobacterium* sp. NRC-1 as a strain of *H. salinarum* and emended description of *H. salinarum*. Extremophiles. 2004;8(6):431–439.

770. Ntougias S, Russell NJ. *Alkalibacterium olivoapovliticus* gen. nov., sp. nov., a new obligately alkaliphilic bacterium isolated from edible-olive wash-waters. Int J Syst Evol Microbiol. 2001;51(3):1161–1170.

771. Spring S, Ludwig W, Marquez MC, Ventosa A, Schleifer KH. *Halobacillus* gen. nov., with descriptions of *Halobacillus litoralis* sp. nov. and *Halobacillus trueperi* sp. nov., and transfer of *Sporosarcina halophila* to *Halobacillus halophilus* comb. nov. Int J Syst Bacteriol. 1996;46(2):492–496.

772. Pascual J, Wüst PK, Geppert A, Foesel BU, Huber KJ, Overmann J. *Terriglobus albidus* sp. nov., a novel acidobacterial species of the family Acidobacteriaceae isolated from Namibian semiarid savannah soil. Int J Syst Evol Microbiol. 2015;.

773. Toffin L, Zink K, Kato C, Pignet P, Bidault A, Bienvenu N, et al. *Marinilactibacillus piezotolerans* sp. nov., a novel marine lactic acid bacterium isolated from deep sub-seafloor sediment of the Nankai Trough. Int J Syst Evol Microbiol. 2005;55(1):345–351.

774. Soto-Ramírez N, Sánchez-Porro C, Rosas-Padilla S, Almodóvar K, Jiménez G, Machado-Rodríguez M, et al. *Halobacillus mangrovi* sp. nov., a moderately halophilic bacterium isolated from the black mangrove *Avicennia germinans*. Int J Syst Evol Microbiol. 2008;58(1):125–130.

775. Yi H, Oh HM, Lee JH, Kim SJ, Chun J. *Flavobacterium antarcticum* sp. nov., a novel psychrotolerant bacterium isolated from the Antarctic. Int J Syst Evol Microbiol. 2005;55(2):637–641.

776. Pugin B, Blamey JM, Baxter BK, Wiegel J. *Amphibacillus cookii* sp. nov., a facultatively aerobic, spore-forming, moderately halophilic, alkalithermotolerant bacterium. Int J Syst Evol Microbiol. 2012;62(Pt 9):2090–2096.

777. Sun B, Cole JR, Sanford RA, Tiedje JM. Isolation and characterization of *Desulfovibrio dechloracetivorans* sp. nov., a marine dechlorinating bacterium growing by coupling the oxidation of acetate to the reductive dechlorination of 2-chlorophenol. Appl Environ Microbiol. 2000;66(6):2408–2413.

778. Langendijk PS, Kulik EM, Sandmeier H, Meyer J, van der Hoeven JS. Isolation of *Desulfomicrobium orale* sp. nov. and *Desulfovibrio* strain NY682, oral sulfate-reducing bacteria involved in human periodontal disease. Int J Syst Evol Microbiol. 2001;51(3):1035–1044.

779. Widdel F. New types of acetate-oxidizing, sulfate-reducing *Desulfobacter* species, *D. hydrogenophilus* sp. nov., *D. latus* sp. nov., and *D. curvatus* sp. nov. Arch Microbiol. 1987;148(4):286–291.

780. Romano I, Lama L, Orlando P, Nicolaus B, Giordano A, Gambacorta A. *Halomonas sinaiensis* sp. nov., a novel halophilic bacterium isolated from a salt lake inside Ras Muhammad Park, Egypt. Extremophiles. 2007;11(6):789–796.

781. Reichenbecher W, Schink B. *Desulfovibrio inopinatus*, sp. nov., a new sulfate-reducing bacterium that degrades hydroxyhydroquinone (1, 2, 4-trihydroxybenzene). Arch Microbiol. 1997;168(4):338–344.

782. Zavarzina DG, Tourova TP, Kolganova TV, Boulygina ES, Zhilina TN. Description of *Anaerobacillus alkalilacustre* gen. nov., sp. nov.–Strictly anaerobic diazotrophic bacillus isolated from soda lake and transfer of *Bacillus arseniciselenatis*, *Bacillus macyae*, and *Bacillus alkalidiazotrophicus* to *Anaerobacillus* as the new combinations *A. arseniciselenatis* comb. nov., *A. macyae* comb. nov., and *A. alkalidiazotrophicus* comb. nov. Microbiology. 2009;78(6):723–731.

783. Miroshnichenko ML, Rainey FA, Hippe H, Chernyh NA, Kostrikina NA, Bonch-Osmolovskaya EA. *Desulfurella kamchatkensis* sp. nov. and *Desulfurella propionica* sp. nov., new sulfur-respiring thermophilic bacteria from Kamchatka thermal environments. Int J Syst Bacteriol. 1998;48(2):475–479.

784. Fröhlich J, Sass H, Babenzien HD, Kuhnigk T, Varma A, Saxena S, et al. Isolation of *Desulfovibrio intestinalis* sp. nov. from the hindgut of the lower termite *Mastotermes darwiniensis.* Can J Microbiol. 1999;45(2):145–152.
